# Comparison of carbon-sulfur and carbon-amine bond in therapeutic drug: 4β-*S*-aromatic heterocyclic podophyllum derivatives display antitumor activity

**DOI:** 10.1038/srep14814

**Published:** 2015-10-07

**Authors:** Jian-Long Li, Wei Zhao, Chen Zhou, Ya-Xuan Zhang, Hong-Mei Li, Ya-Ling Tang, Xin-Hua Liang, Tao Chen, Ya-Jie Tang

**Affiliations:** 1 Key Laboratory of Fermentation Engineering (Ministry of Education), Hubei Provincial Cooperative Innovation Center of Industrial Fermentation, Hubei University of Technology, Wuhan 430068, China; 2 State Key Laboratory of Oral Diseases West China Hospital of Stomatology (Sichuan University), Chengdu Sichuan 610041,People’s Republic of China; 3 Key Laboratory of Systems Bioengineering (Ministry of Education), School of Chemical Engineering and Technology, Tianjin University, Tianjin 300072, China

## Abstract

Herein is a first effort to systematically study the significance of carbon-sulfur (C-S) and carbon-amine (C-NH) bonds on the antitumor proliferation activity of podophyllum derivatives and their precise mechanism of apoptosis. Compared with the derivative modified by a C-NH bond, the derivative modified by a C-S bond exhibited superior antitumor activity, the inhibition activity of target proteins tubulin or Topo II, cell cycle arrest, and apoptosis induction. Antitumor mechanistic studies showed that the death receptor and the mitochondrial apoptotic pathways were simultaneously activated by the C-S bond modified aromatic heterocyclic podophyllum derivatives with a higher cellular uptake percentage of 60–90% and induction of a higher level of reactive oxygen species (ROS). Only the mitochondrial apoptotic pathway was activated by the C-NH bond modified aromatic heterocyclic podophyllum derivatives, with a lower cellular uptake percentage of 40–50%. This study provided insight into effects of the C-S and C-NH bond modification on the improvement of the antitumor activity of Podophyllum derivatives.

Podophyllotoxin (PTOX) and its analogue 4′-demethylepipodophyllotoxin (DMEP) are the most well-known naturally-occurring aryltetralin lignans exhibiting anti-tumor activity. Numerous reviews emphasized the occurrence, synthesis and applications of PTOX and DMEP derivatives^1^,^2^. Modifications have been made at several positions of its skeleton with the aim to either improve its potency or to overcome drug resistance. In recent years, the structurally modified podophyllotoxins have been investigated for their apoptosis inducing ability, with recent progress systematically reviewed by Kamal *et al.*^1^. The modification at the 4 position of cycloparaffin (C-4 position) has been identified as an effective method to facilitate the druggability^2^,^3^. Structural modifications of the C-4 position on PTOX and DMEP can be achieved through both O- N- and S-linkages. To date, hundreds of derivatives have been designed and synthesized based on podophyllum compounds, such as etoposide (VP-16)^4^ and teniposide (VM-26)^5^, both of which are more potent antitumor agents than DMEP.

A bioisostere is a substituent with similar physical or chemical properties to the parent substituent which produce broadly similar biological properties but potentially leads to improvements in activity or reductions in toxicity^6^,^7^. The oxygen atom, sulfur atom, and amine (-NH-) group are bioisosteres with the same numbers of the outermost electrons and bond angle. So, substitution of the carbon-oxygen (C-O) bond at the C-4 position of PTOX and DMEP with C-S and C-NH bonds may also be a potential modification direction for improving the antitumor activity. Currently, the carbon-amine (C-NH) bond substitution at the C-4 position of PTOX and DMEP has attracted many medicinal chemists’s eyes to develop more podophyllum derivatives potential to be drugs,: perhaps the most celebrated example is the 4β-(4“nitroanilino)-4-deoxy-4′-demethylepipodophyllotoxin (GL331), which has been used for antitumor chemotherapy^8^. Meanwhile, we previously reported on novel carbon-sulfur (C-S) bond substituted podophyllum derivatives with the superior antitumor activity: the IC_50_ value of 4β-*S*-(1, 2, 4-triazole-3)-4-deoxy-podophyllotoxin against cancer cell line human gastric cell line BGC-823 and A549 were significantly improved by 91 and 221 times over those of etoposide, respectively^9^. These results demonstrate that the C-S and C-NH bonds linkage between the substituent group and the C-4 position of PTOX and DMEP make a significant contribution for the antitumor proliferation activity of podophyllum derivatives. In the past several years, multiple C4-NH and S-substituted podophyllum derivatives were synthesized and explored but the comparison between C4-NH and S-substituted podophyllum derivatives on the SAR have received less attention. An effort is underway to use podophyllum compounds as the model for natural lead compounds, and to study the effect of the C-S and C-NH bond modification on the improvement of antitumor activity.

In the present work, by taking podophyllum compound as an excellent research model for the natural lead compound, we systematically study the significance of the C-S and C-NH bonds modification on the antitumor activity of podophyllum derivatives and their precise mechanism. The results provide guidance for modifications of other natural lead compounds.

## Results

### Design and synthesis of 4β-*S*- and 4β-*NH*-aromatic heterocyclic podophyllum derivatives

The process of drug molecular design was performed in the following four steps. Firstly, the steric clash and electrostatic contour plots of the comparative molecular field analysis model^10^ showed that the carbon atom at the 4 position of cycloparaffin (C-4 position) of podophyllotoxin (PTOX) and its analogue 4′-demethylepipodophyllotoxin (DMEP) is more active by comparing other carbon atoms in the tetranap skeleton of PTOX and DMEP. Therefore, structural modifications at the C-4 position of PTOX and DMEP may be effective alterations for improving the antitumor activity. Secondly, from the viewpoint of bioisosteric replacement, the physical or chemical properties of the carbon-sulfur (C-S) and carbon-amine (C-NH) bonds were similar with the C-O bond but potentially provided superior biological properties for bioactivity improvement and the toxicity reduction. Numerous lines of evidence suggested that C-S and C-NH bond modifications were promising for anti-inflammatory and antitumor agent design^1^,^3^,^11^. Therefore, C-S and C-NH bonds were selected as the modification direction for linking podophyllum compounds and substituents group at the C-4 position of PTOX and DMEP. Thirdly, the pharmacophoric groups would be further considered after confirming the effective modification directions of C-4 position and C-S/NH bond. It is well known that functionalisation of drugs through an ester bond can produce compounds with better bioactivity. In particularly, addition of an aromatic heterocyclic with unique chemical properties (i.e., dense electron cloud, rigid frames) and wide-ranging biological activities (i.e., metabolic stability, drug-likeness) can be effective. Five-member ring systems such as thiophene, 1, 3, 4-thiadiazole and 1, 2, 4-triazole have exhibited notable bioactivities, such as antitumor, anticonvulsant, antimicrobial, antitubercular and antiviral properties^12^,^13^. Hu *et al.*^14^ designed and synthesized a series of novel NH-substituted podophyllotoxin derivatives with potent anti-tumor activity and better selectivity toward the normal cell. Sampath Kumar^15^ designed in *silico* and prepared a series of 4β-[(4-alkyl)-1, 2, 3-triazol-1-yl] podophyllotoxin derivatives, which proved to be more potent than etoposide and exhibited significant anti-tumor activity with IC_50_ values in the range of 0.001–1 μM. Purine analogues have been shown to damage the integrity of DNA and to kill cells as effectively as pyrimidine substituents^16^,^17^. Thus, naphthalene or benzothiazole cores might have similar bioactivity. Taking these findings into consideration, aromatic heterocycles including five-member, six-member and condensed rings linked with C-S and C-NH bond at the C-4 position of PTOX and DMEP were selected for the effective structure modifications. And in total, 36 new compounds were designed. All of the designed compounds were named using an abbreviation form of “substituent group -parent structure-bond type”. For example, the number “1” represented 1, 3, 4-trizole. The “1S” and “1′S” represented that 1, 3, 4-trizole was linked at C-4 position of PTOX and DMEP by C-S bond, respectively. The “1N” and “1′N” represented that 1, 3, 4-trizole was linked at the C-4 position of PTOX and DMEP by a C-NH bond, respectively. So, Compound 1S describes 4β-*S*-(1, 3, 4-trizole-2)-4-deoxy-podophyllotoxin.

**Figure 1:**
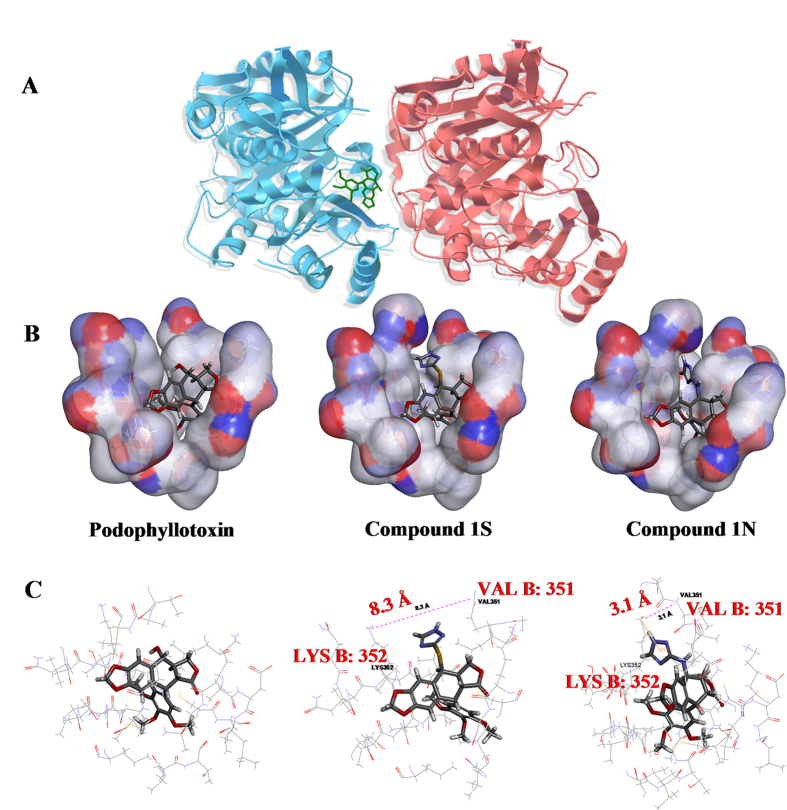
(**A**) Structure of the tubulin cleavage complex stabilized by podophyllum derivatives. (**B**) Podophyllotoxin (PTOX) was used as a positive control. PTOX, 4β-*S*-(1, 3, 4-trizole-2)-4-deoxy-podophyllotoxin (Compound 1S), and Compound 4β-*NH*-(1, 3, 4-trizole-2)-4-deoxy-podophyllotoxin (Compound 1N) were found to bind the active site between α/β interfaces with the surface model treatment in 3D image. The red and blue surfaces were the hydrophobic and hydrophilic regions, respectively. (**C**) Active interaction fragments were found to bind the actsive site between α/β interfaces with the amino acids model treatment. Schematic representation of the interactions between tubulin and Compound 1S/1N interactions were marked with lines: pink dotted lines indicate H-bonds length of LYS352 and VAL351, the orange yellow solid lines were π-π stacking interactions to the tubulin. The red solid lines in amino acids were the unsaturated bonds.

**Figure 2:**
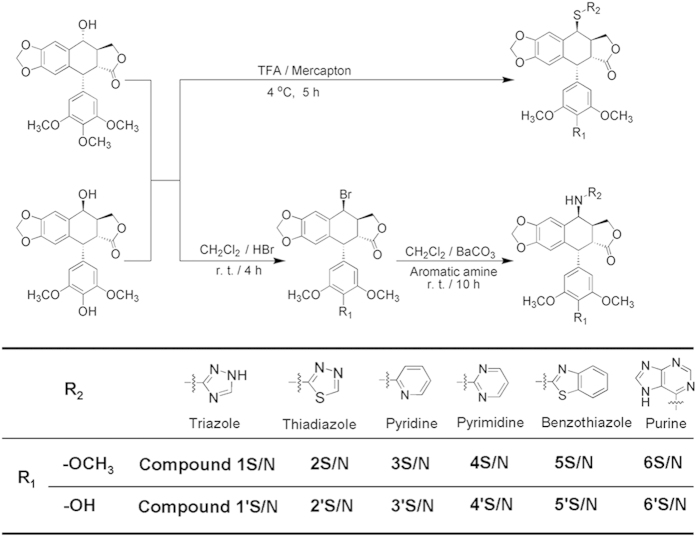
Synthesis of 4β-*S*- and 4β-*NH*-aniline derivatives of podophyllum derivatives from podophyllotoxin and 4′-demethylepipodophyllotoxin.

Finally, in order to improve efficiency of drug design, the tubulin-stathmin complex (PDB code: 1SA1) and topoisomerase II (Topo II) DNA cleavage complex (PDB code: 3QX3) were used as the target proteins for the virtual screening of the designed PTOX and DMEP derivatives, respectively. The structure-based CDOCKING energy (-kcal/mol) (Table S1) was analyzed for 36 designed virtual podophyllum derivatives interacting with residues in the tubulin colchicine domain^18^,^19^ or Topo II etoposide (VP-16) domain^20^. New designed PTOX derivatives Compounds 1S~6S, 1N~6N and DMEP derivatives Compounds 1′S~6′S, 1′N~6′N possessed affinities with tubulin (Energy: 2.11-6.28 -kcal/mol) and Topo II (Energy: 2.69-8.42 -kcal/mol) similar or stronger than PTOX (Energy: 4.11 kcal/mol) and etoposide (Energy: 6.38 kcal/mol) (Table S1), respectively. However, PTOX derivatives Compounds 7S~9S, 7N~9N and DMEP derivatives Compounds 7′S~9′S, 7′N~9′N, could not bond with tubulin (Energy: −2.33~−11.04 -kcal/mol) or Topo II (Energy: −4.23~−7.23 -kcal/mol) (Table S1). So, Compounds 1S-6S, 1N-6N, 1′S~6′S, and 1′N~6′N showed strong binding capacities with target proteins tubulin or Topo II. Contrarily, Compounds 7S-9S and 7N-9N failed to dock at an active site of tubulin. For example, the best docked configurations with the lowest glide scores of the designed 4β-*S*-(1, 3, 4-trizole-2)-4-deoxy-podophyllotoxin (Compound 1S) and Compound 4β-*NH*-(1, 3, 4-trizole-2)-4-deoxy-podophyllotoxin (Compound 1N) were located at the α/β interface of tubulin (Fig. 1A). The lipophilic nature of the tubulin region around the trimethoxyphenyl fragment (β-unit of tubulin) can be observed while the possible hydrogen bond interactions of the pinacol function with the hydroxyl group of threonine (α-unit of tubulin) are also indicated (Fig. 1B). By analyzing the results of the docking studies, the cleavage site-specific Compound 1S insertion physically interferes with the stacking between the amino acid residues LYS352 and VAL351 by 8.3 Å (Fig. 1C), which effectively traps the tubulin by preventing the α/β subunit normal religation reaction. However, the cleavage site-specific Compound 1N insertion physically interferes with the stacking between the amino acid residues LYS352 and VAL351 by 3.1 Å. The degree of separation of the cleaved α/β subunit caused by Compound 1S was more than that of Compound 1N. The Compound-induced separation of the cleaved α/β subunit was accompanied by the two amino acid residues sliding away from each other. In this effort, 24 of the designed novel 4β-*S*- and 4β-*NH*-aromatic heterocyclic podophyllum derivatives were screened out as target compounds (Fig. 2).

### *In vitro* cytotoxicity

Compared with the clinically important podophyllum anticancer drug etoposide, the antitumor activity of aromatic heterocyclic podophyllum derivatives modified by the C-S and C-NH bond was in general improved by 5–450 times (Table 1). Surprisingly, the C-S bond was more effective to enhance antitumor activity than the C-NH bond, such as the 50% inhibitory concentration (IC_50_) of Compound 1S (i.e., IC_50_ value of 0.17 ± 0.02, 0.34 ± 0.02, and 0.79 ± 0.05 μM) was about 29, 3 and 6 times higher than that of Compound 1N (i.e., 3.85 ± 0.21, 1.25 ± 0.13, and 5.33 ± 0.52 μM) against HeLa, BGC-823 and A549 cells, respectively. The IC_50_ of 4β-*S*-(1, 3, 4-trizole-2)-4-deoxy-4′-demethylepipodophyllotoxin (Compound 1′S) (i.e., IC_50_ value of 0.32 ± 0.03 μM) was 16 times higher than that of 4β-*NH*-(1, 3, 4-trizole-2)-4-deoxy-4′-demethylepipodophyllotoxin (Compound 1′N) (i.e., 4.36 ± 0.42 μM) against HeLa cells. The HeLa cell line was the most sensitive cancer cell lines. In terms of the cytotoxicity to the normal cell of human hepatocyte cell line (HL-7702), most of the compounds showed comparable or lower inhibition activity relative to etoposide, especially the cytotoxicity of 4β-*S*-(1,2,4-triazole-3-)-4-deoxy-4′-demethylepipodophyllotoxin (Compound 2′S) was 7 times lower than etoposide. The above results demonstrated that the C-S and C-NH bonds modification were in general effective to enhance the antitumor activity and reduce the cytotoxicity to normal cells. The C-S bond modification was better than the C-NH bond modification. Because of the superior antitumor activity, Compound 1S and 1N were considered as the representative derivatives for the further studies of bioactivity and mechanism. PTOX derivatives have been reported to bind with tubulin and inhibit cellular microtubule polymerization^21^. DMEP derivatives could bind to Topo II-DNA active site and induce the breakage of single- and double-stranded DNA^22^,^23^. Therefore, tubulin and Topo II were used as the target protein for the study on the mechanism of PTOX and DMEP derivatives, respectively.

### Effect of Compounds 1-6S and 1-6N on the inhibition of cellular microtubule polymerizati**on**

**Figure 3:**
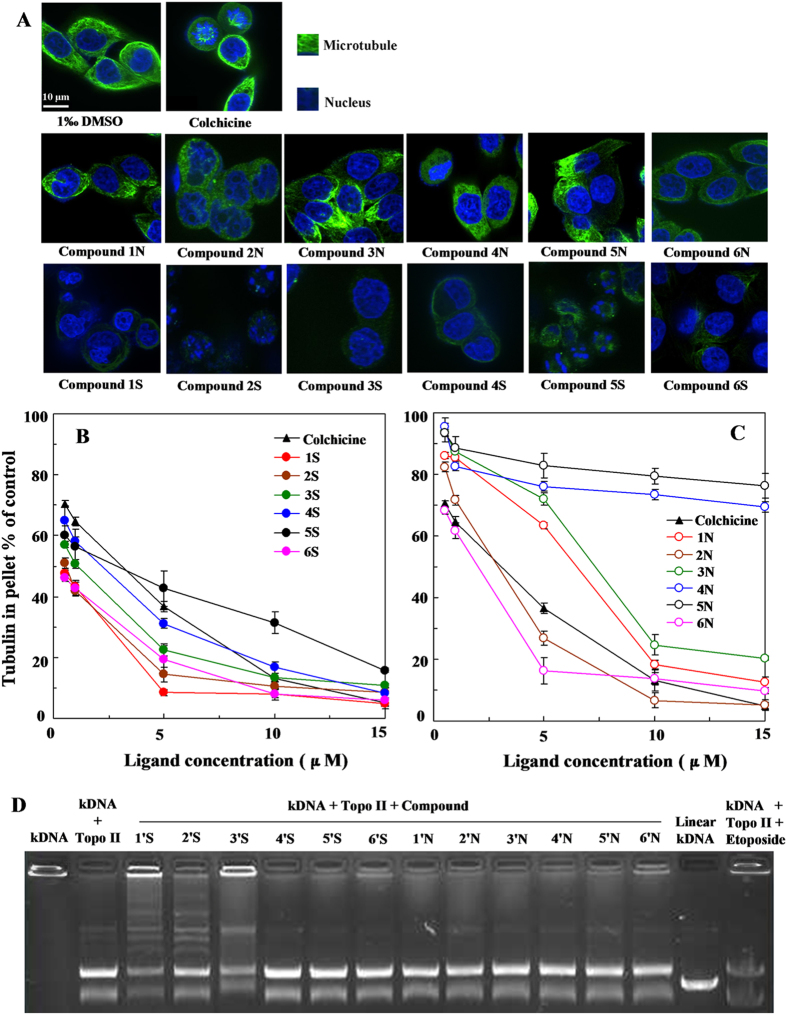
Effect of podophyllum derivatives on the inhibition of cellular microtubule polymerization (A), the scale bar represents 10 μm. Inhibition of tubulin assembly in *vitro* by colchicine, compounds 1-6S and 1-6N (**B,C**) or topoisomerase II catalytic activity (**D**).

Availability of tubulin is essential to mitosis, and therefore PTOX and its derivatives effectively function as a “mitotic poison” or spindle poison^19^. The effect of Compounds 1S and 1N on the inhibition of cellular microtubule polymerization was investigated. Compared with the positive control colchicine and Compound 1-6N, Compound 1-6S (at the high concentration of 10 μM) showed potent depolymerization of the microtubule (Fig. 3A). Furthermore, the degree of tubulin polymerization was evaluated through pellet mass formation in centrifugation assays in the presence of stoichiometric and semi-stoichiometric concentrations of each lignan. Inhibition curves were used to determine GI_50_, which is the concentration that causes 50% growth inhibition. Several of them displayed effects higher than colchicine, used as positive control, especially, compound 1-6S exhibited stronger microtubule depolymerization (GI_50_ < 1 μM) (Fig. 3B,C). Interestingly and in parallel with the cytotoxicity values shown in Table 1 and inhibition of cellular microtubule polymerization shown in Fig. 3B, those compounds of the NH series were less potent than those of the S series (Compound 1-6S vs 1-6N, respectively). These results indicated that Compound 1S was a microtubule depolymerization agent with higher activity than Compound 1N, even better than colchicine.

### Stabilization of Topoisomerase II-DNA cleavage complex

Kinetoplast DNA (kDNA) is a catenated network of mitochondrial DNA seen in trypanosomes^24^,^25^. The kDNA decatenation assay has been utilized to test drug potential to inhibit Topo II activity^26^. The catalytic activity of Topo II was nearly inhibited by Compounds 1′S and 3′S at the high concentration of 100 μM. But a positive control VP-16 had partly inhibition effect on catalytic activity of Topo II. The kDNA decatenation almost did not affected by Compound 1′-6′ N, which were worse than Compounds 1′-6′S (Fig. 3B). These indicated that the Topo II catalytic activity of Compounds 1′-3′S was higher than VP-16 and Compound 1′-6′ N.

### Effect of Compounds 1S/1N and 1′S/1′N on cell cycle arrest and induced apoptosis in HeLa cells

Comparing with HepG2 and A549 cells, tumor cell HeLa exhibited strong drug sensitivity to the 4β-*S* and 4β-*NH*-aromatic heterocyclic podophyllum derivative in the above *in vitro* cytotoxicity experiment. As a cell type in an immortal cell line, HeLa cells were often also used in the mechanism of antitumor drug scientific research. So, HeLa cells were used as a cell model for the following study. Notably, the cell cycle arrest ratio induced by Compound 1S was higher than Compound 1N throughout the 12–48 h (Figure S1A). The primarily G_2_/M arrest noted at 24 and 48 h might be not consistent with the apoptosis. Following the treatment of Compounds 1S, 1N, 1′S and 1′N at the concentration of 0–5 μM for 6–48 h, the highest ratio up to 60% and 50% of cells were detected to be undergoing apoptosis, respectively (Figure S1B). Interestingly, the C-S and C-NH bonds modification aromatic heterocyclic podophyllum derivatives exhibited a similar effect on the G_2_/M phase arrest, but the apoptosis of cells induced by Compound 1S were significantly higher than that induced by Compound 1N, and Compound 1′S showed higher potency than Compound 1′N to induce the cell death through apoptosis. The above results demonstrated that the C-S bond modification aromatic heterocyclic podophyllum derivatives might induce apoptosis via an alternative mechanism, which we subsequently investigated further as described below.

### Compounds 1S, 1N, 1′S, and 1′N induced the caspase-dependent apoptosis pathway

As previously reported, PTOX derivatives induce the apoptosis of cancer cells by damaging the spindle assemble in mitosis. Upon the depolymerization of microtubule, Bim_EL_ sequestered the microtubule by interaction with the light chain of dynein. Bim could further promote the release of the proapoptotic protein Bax, which is in turn responsible for mitochondrial dysfunction and caspases activation^27^,^28^. In the intrinsic or mitochondrial pathway of apoptosis, caspase-9 activation is the marker event. Consecutively, the executioner caspase-3 is recruited to the cleaved caspase-9, where it is activated by the active caspase-9^29^,^30^. In the extrinsic or death receptor pathway of apoptosis, stimulation of death receptors results in the initiator caspase-8 which can propagate the apoptosis signal by direct cleavage of downstream caspase-3^31^,^32^.

**Figure 4:**
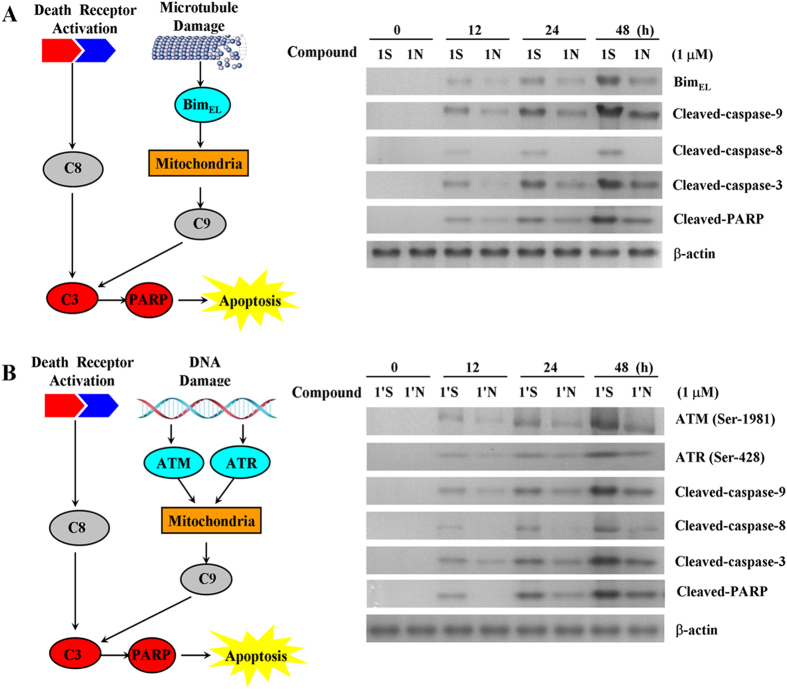
Detection of apoptosis pathway protein in HeLa cells by using Western blot analysis. (**A**) Effects of Compound 4β-*S*-(1, 3, 4-trizole-2)-4-deoxy-podophyllotoxin (Compound 1S) and Compound 4β-*NH*-(1, 3, 4-trizole-2)-4-deoxy-podophyllotoxin (Compound 1N) with an adding concentration of 1 μM on the levels of Bim, caspase-9, caspase-8, caspase-3 for 48 h. The extent of proteins level was then determined by immunoblotting. The gels have been run under the same experimental conditions. Each column represents the mean ± S.E.M. of three independent experiments. The full-length blot is presented in Supplementary Figure 2S. (**B**) Effects of 4β-*S*-(1, 3, 4-trizole-2)-4-deoxy-4′-demethylepipodophyllotoxin (Compound 1′S) and Compound 4β-*NH*-(1, 3, 4-trizole-2)-4-deoxy-4′-demethylepipodophyllotoxin (Compound 1′N) with an adding concentration of 1 μM on the levels of ATM, ATR, caspase-9, caspase-8, caspase-3 for 48 h. The extent of proteins level was then determined by immunoblotting. The gels have been run under the same experimental conditions. Each column represents the mean ± S.E.M. of three independent experiments. The full-length blot is presented in Supplementary Figure 3S.

To understand the molecular events that regulate apoptosis, the active patterns of various intracellular proteins including the pro-apoptosis protein Bim_EL_ and caspase-9, caspase-8, caspase-3, were detected by Western blot with the treatment of Compounds 1S and 1N at 1 μM. Bim_EL_, cleaved-caspase-9, cleaved-caspase-8, cleaved-caspase-3, and cleaved-PARP were detected after the treatment of Compound 1S for 12 h, and as the exposure time prolonged, the signal was further activated until 48 h (Fig. 4A). Except for caspase-8, which was activated in a similar manner corresponding to Compound 1S, Bim, caspase-9 and caspase-3 were also initiated by Compound 1N, but with weaker and delayed signal than Compound 1S. This result revealed that Compound 1S activated the mitochondrial and the death receptor apoptotic pathways, and Compound 1N only stimulated the mitochondrial apoptotic pathway. Obviously, Compound 1S showed superior activity than Compound 1N to strengthen the apoptosis signal, which may be partly because of the activation of the death receptor pathway.

As the cleaved-DNA accumulated to a certain extent, the DNA damage checkpoint signal protein ataxia telangiectasia mutated (ATM) and ataxia telangiectasia-mutated and Rad3-related (ATR) would be phosphorylated to active the following signal pathway including DNA repair, cell cycle arrest and apoptosis^33^. To study the mechanism of DMEP derivatives, the activation of ATM, ATR, and caspases including caspase-8, 9, and 3 were detected after the treatment of Compounds 1′S and 1′N for 0-48 h. As shown in Fig. 4B, the activation of all these proteins by Compound 1′S were observed, and these proteins were also activated by Compound 1′N though with significantly weaker signal. It was worth noting that the cleaved-caspase-8 appeared until 48 h after the treatment of Compound 1′N. The above results demonstrated that the death receptor and the mitochondrial apoptotic pathways were activated by the C-S bond modified aromatic heterocyclic podophyllum derivatives, while only the mitochondrial apoptotic pathway was activated by the C-NH bond modified aromatic heterocyclic podophyllum derivatives.

### Effect of Compounds 1S/1N on the reactive oxygen species (ROS) in HeLa cells

**Figure 5:**
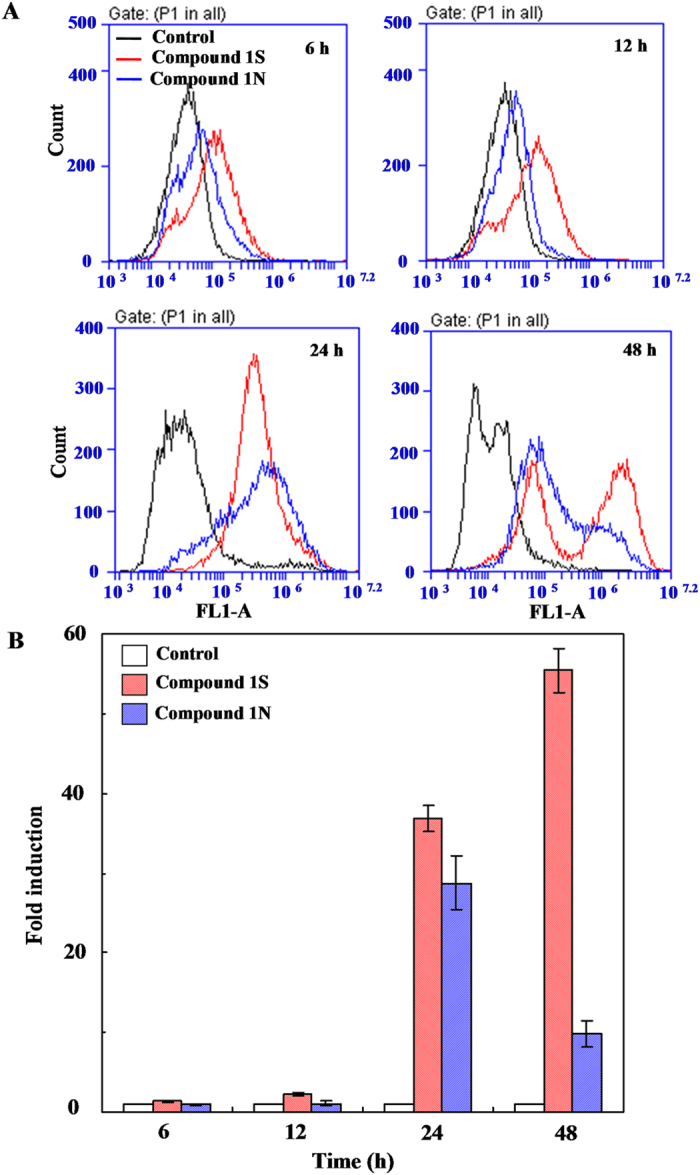
Effects of Compound 4β-*S*-(1, 3, 4-trizole-2)-4-deoxy-podophyllotoxin (Compound 1S) and Compound 4β-*NH*-(1, 3, 4-trizole-2)-4-deoxy-podophyllotoxin (Compound 1N) on the reactive oxygen species (ROS) levels in HeLa cells. (**A**) Flow cytometric analysis was used to determine a concentrations of 10 μM Compound 1S/1N induced ROS generation in a time-dependent manner for 6, 12, 24, and 48 h, respectively. The abscissa of ROS was the fluorescence intensity. The ordinate was the count of cells containing ROS. Symbols: the negative control without adding Compounds (0 μM) (**—**), Compound 4β-*S*-(1, 3, 4-trizole-2)-4-deoxy-podophyllotoxin (Compound 1S) (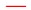); Compound 4β-*NH*-(1, 3, 4-trizole-2)-4-deoxy-podophyllotoxin (Compound 1N) (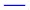). (**B**) Measurement of ROS in HeLa cells compared to empty vector. Mean values + standard deviation (SD) of three independent experiments are shown. h indicates hour here after. Symbols: the negative control without adding Compounds (0 μM) (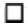), Compound 1S (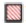), Compound 1N (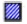).

ROS are formed as a natural byproduct of the normal metabolism of oxygen and have important roles in cell signaling and homeostasis. However, excessive ROS can induce apoptosis through both the extrinsic and intrinsic pathways^34^,^35^,^36^. In this study, compared with the control (the cells incubated with no drug), a significant early increase of ROS in cells were induced by Compound 1S and Compound 1N after 6 and 12 h (Fig. 5A). After 24 h of induction with Compound 1 S and Compound 1N, a rapid increase of the fluorescence intensity of ROS was observed. The fluorescence intensity of ROS further increased from 10^5^–10^6^ to 10^6^–10^7^.^2^ for approximaetly half of the cells after 48 h induction with Compound 1S. In contrast, the fluorescence intensity of ROS decreased from 10^5^–10^6^ to 10^4^–10^5^ for most cells after 48 h induction with Compound 1N (Fig. 5A). Compared with the Compound 1N, the amount of ROS (calculated as: the fluorescence intensity of ROS × the count of cells containing ROS) induction with Compound 1S were improved by 4.8 times after 48 h. The above results demonstrated that the C-S bond modification aromatic heterocyclic podophyllum derivatives showed a higher potency for generating ROS than those of the C-NH bond in HeLa cells. This result is consistent with Compound 1N being less efficient and weaker than Compound 1S on the activated caspase-9 in the mitochondrial apoptotic pathways.

## Discussion

**Figure 6:**
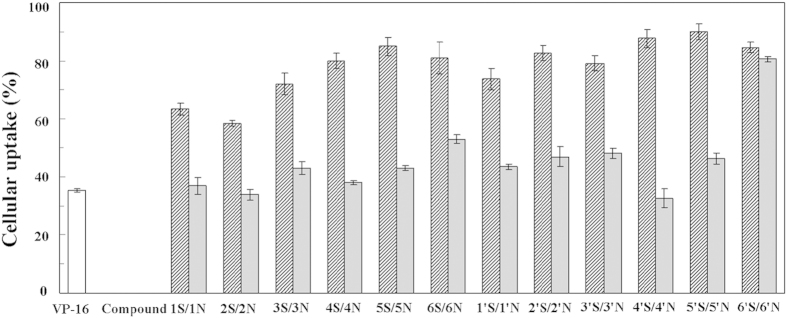
Cellular uptake analysis of the podophyllum derivatives in HeLa cells. Symbols: etoposide (VP-16) (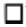); 4β-*S*-aromatic heterocyclic podophyllotoxin derivatives (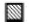), 4β-*NH*-aromatic heterocyclic 4′-demethylepipodophyllotoxin derivatives (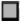).

**Figure 7:**
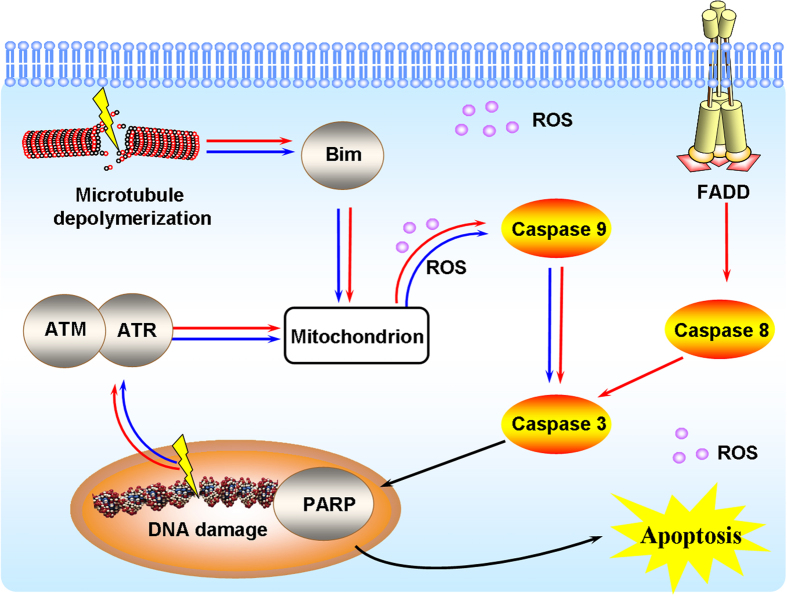
The integrated apoptotic pathways, a schematic diagram showing some of the known components of the intrinsic and the death receptor apoptotic programs and the mitochondrial apoptotic pathways. The carbon-sulfur and carbon-amine bonds modification podophyllum compounds showed that the death receptor and the mitochondrial apoptotic pathways were activated by the carbon-sulfur bond modified aromatic heterocyclic podophyllum derivatives. Only the mitochondrial apoptotic pathway was activated by the carbon-amine bond modified aromatic heterocyclic podophyllum derivatives. Symbols: the pathways were activated by Compound 4β-*S*-(1, 3, 4-trizole-2)-4-deoxy-podophyllotoxin (Compound 1S) (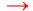); the pathways were activated Compound 4β-*NH*-(1, 3, 4-trizole-2)-4-deoxy-podophyllotoxin (Compound 1N) (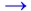).

Most of the currently available antitumor drugs have been discovered empirically by screening of large numbers of compounds for efficacy against tumor models. The rational design of a drug is usually based on biochemical and physiological differences between tumors and normal tissue. Some of the most striking differences between tumor cells and their protein are found in proliferation pathways. Therefore, tubulin and Topo II constitute excellent potential targets for the rational design of antitumor chemotherapeutic regimens. Sulfur and nitrogen atoms exist in natural products and clinical drugs widely, making a significant contribution for the balance of heteroatom substituent in drugs molecules. This bioisosteric replacement may dramatically change the biological activity of the natural products. Herein, the difference between the carbon-sulfur (C-S) and carbon-amine (C-NH) bond modification on the antitumor activity of natural product podophyllum derivatives and their precise mechanism were systematically studied for the first time. Most of the designed C-S bond modified aromatic heterocyclic podophyllum derivatives exhibited much higher cytotoxic potency than that of the C-NH bond modified aromatic heterocyclic podophyllum derivatives against cancer cell lines. Because the electronegativity of the sulfur atom was lower than that of the nitrogen atom, the hydrophobic thioether bond (-S-) is better than imino bond (-NH-) to improve the cellular uptake of the compounds which may enhance their biological activity. Measurement of the cellular uptake of the compounds indicated that all the C-S bond modified aromatic heterocyclic podophyllum derivatives showed significantly higher cellular uptake percentage (60–90%) than the C-NH bond modified aromatic heterocyclic podophyllum derivatives (35–80%) (Fig. 6). Furthermore, while the cellular uptake results generally demonstrated consistently higher uptake of (S) derivatives, the purine derivatives of both (S) and (N) showed similar cellular uptake. It is well known that purines are indispensable to all life, performing many vital functions for cells. Unlike triazole, thiadiazole, pyridine, pyrimidine, and benzothiazole heterocycles, purine is a precursor for activated forms of both carbohydrates and lipids, and nucleotide derivatives of vitamins are essential cofactors in metabolic processes^37^. Purine bases pass the cell membrane by facilitated diffusion^38^, and most proliferating neoplastic cells show enhanced uptake of purine bases when compared to quiescent cells^39^. Therefore, active transport for drug targeting has been the focus of considerable scientific investigation^40^. This rapid absorption might facilitate higher concentration of the compounds around the active site of the target protein. So, the C-S bond modified compounds induced an earlier stress response caused by the dysfunction of the targets such as microtubule and Topo II-DNA complex, which lead to the earlier activation of the caspases-dependent apoptosis pathway. On the other hand, the earlier and stronger activation or expression of apoptosis related signal proteins revealed the higher potent activity of the C-S bond modified aromatic heterocyclic podophyllum derivatives. Unlike Compound 1N, except for the mitochondrial apoptosis pathway, Compound 1S demonstrated higher potency to activate the death receptor pathway, as observed by the significant activation of caspase-8 (Fig. 7). Corresponding to this, Compound 1′S also activated caspase-8 significantly, but Compound 1′N did not induce the cleaved-caspase-8 until 48 h after the treatment of Compound 1′N against HeLa cells. This may be caused by the higher affinity of Compounds 1S and 1′S to the cytomembrane, leading to further activation of the death receptor which attached on the membrane. Furthermore, the C-S bond modification aromatic heterocyclic podophyllum derivatives showed higher potency for generating ROS in HeLa cells than of the corresponding analogues with a C-NH bond in HeLa. The result may partly explained the phenomena that the activity of Compounds 1S and 1′S to arrest the cell cycle and induce apoptosis is more potent than Compounds 1N and 1′N. These results indicated the death receptor and the mitochondrial apoptotic pathways were activated by the C-S bond modified aromatic heterocyclic podophyllum derivatives, but only the mitochondrial apoptotic pathway was activated by the C-NH bond modified aromatic heterocyclic podophyllum derivatives (Fig. 7).

In conclusion, by systematically studing the significances of C-S and C-NH bonds on the antitumor proliferation activity of podophyllum derivatives and their precise mechanism of apoptosis, the C-S bond aromatic heterocycles at the C-4 position of PTOX and DMEP were found to be an effective modification to enhance antitumor activity and reduce toxicity. This study revealed that the C-S bond substitution was an effective direction for the rational modification of natural products.

## Methods

### Chemicals

The starting materials podophyllotoxin and 4′-demethylepipodophyllotoxin were purchased from Xi’an Helin Bio-technique Co., Ltd; the 1,2,4-triazole-3-thiol, triazol-3-amine, 2-mercapto-1,3,4-thiadiazol, 2-amino-1, 3, 4-thiadiazole, 2-mercaptopyridine, 2-aminopyridine, 2-mercaptopyrimidine, 2-aminopyrimidine, 2-mercaptobenzothiazole, 2-benzothiazolamine, 6-mercaptopurine and adenine were purchased from J&K Chemical; trifluoroacetic acid were purchased from Aladdin; hydrogen bromide, dichloromethane, methanol, acetonitrile, trichloromethane and acetone were (from Sinopharm Chemical Reagent Co., Ltd) used without purification; methanol and acetonitrile (HPLC/Spectro grade) were purchased from Tedia Company, Inc., all the deionized water was filtered with 0.22 μm membrane.

Cell lines used in this work including cervical epithelioid carcinoma (HeLa), human gastric carcinoma (BGC823) and adenocarcinomic human alveolar basal epithelial carcinoma (A549) were purchased from American-Type Culture Collection (ATCC). Cells were grown in media supplemented with fetal bovine serum (FBS) and antibiotics (100 μg/mL penicillin and 100 U/mL streptomycin). Specifically, experiments were performed using the following cell lines and media compositions: HeLa and A549 (RPMI-1640 and 10% FBS), and BGC823 (DMEM and 10% FBS). Cells were incubated at 37 °C in a 5% CO_2_, 95% humidity atmosphere. Phosphate-buffered saline (PBS) were purchased from Thermo Fisher Scientific. For biological assays, propidium iodide (PI) and fluorescein isothiocyanate (FITC) were purchased from Multi-Sciences Biotech Co., Ltd. The 3-(4, 5-dimethylthiazol-2-yl)-5-(3-carboxymethoxyphenyl)-2-(4-sulfophenyl)-2H-tetrazolium salt (MTT) was obtained from Aladdin. Immunofluorescence agents including immune staining fix solution, sealing fluid, Hoechst 33258, tubulin primary antibody and immunol fluorescence staining kit with FITC-labeled secondary antibody (goat anti-mouse) were purchased from Beyotime Institute of Biotechnology. Caspase-8, caspase-9, caspase-3, β-actin and phosphate-Bcl-2 (Ser70) (rabbit, dilution 1:1000, Thermo Fisher Scientific) were used as primary antibodies and ECL rabbit IgG (dilution 1:2000, Beijing CoWin Bioscience Co., Ltd.) were used as secondary antibodies. The chemiluminescent reagent was purchased from Merck Millipore.

### Instrumentation

All Proton nuclear magnetic resonance (^1^H NMR) spectra, Carbon-13 nuclear magnetic resonance (^13^C NMR) spectra and ^1^H-^1^H COSY, HSQC, and HMBC were recorded with a Varian Mercury VX-300 spectrometer or a Agilent DD2 400-MR NMR spectrometer. Chemical shifts (δ) are reported in ppm relative to the TMS internal standard. Abbreviations are as follows: s (singlet), d (doublet), dd (doublet of doublets), t (triplet), q (quartet) and m (multiple). HPLC analysis was performed on a Dionex system with the Ultimate 3000 Binary HPLC Pump, Ultimate 3000 diode array Detector, using a Promisil C18 (Bonna-Agela Technologies Inc.), 150 mm × 4.6 mm column with gradient H_2_O, 0.1% TFA/CH_3_OH and 0.1% TFA from 20% to 80% organic in 45 min, a flow rate of 0.8 mL/min, and UV detection at 210 nm. Mass spectra were recorded on a Agilent 6420 LC-MS system using an electrospray (ESI) ionization source. Precoated silica gel plates (Merck, Kieselgel 60 F254, 0.25 mm) were used for TLC analysis. Analytical thin layer chromatography (TLC) was performed using precoated silica gel plates (Merck, Kieselgel 60 F254, 0.25 mm). Flash column chromatography was performed using granular silica gel (60-Å pore size, 40–60 μm, Qingdao Haiyang Chemical Co. Ltd.). The separation was carried on a reversed-phase column with dimensions 250 × 10 mm and a particle size of 5 μm.

Cytotoxicity of the podophyllum derivatives were determined by reading the absorbance at 492 nm on a multiplate reader (Thermo Fisher Scientific, Multiskan MK3). Cell cycle analysis and apoptosis analysis were analyzed by flow cytometry (Accuri C6, BD). Immunofluorescence images were recorded using laser scanning microscope (Perkin-Elmer & Olympus, UltraView VoX & Ix81). The gel for the decatenation of kDNA by Topoisomerase II study was photographed with UV illumination, Tanon 1600 (Tanon Science & Technology Co., Ltd., Shanghai). The nitrocellulose blotting membrane for western blot analysis was visualized using X-ray film processor and X-ray film, Fujifilm SUPER RX-N-C.

### General procedure for the preparation of compound 1S-6S, 1′S-6′S

PTOX (414 mg, 1 mmol) or DMEP (400 mg, 1 mmol), and function module (1 mmol) were mixed in 5 mL trifluoroacetic acid (TFA). All reactions were stirred under nitrogen at 0 °C for 48 h and monitored by TLC and then the liquid was dropped into 100 mL cold deionized water. Collect and dry the solid at 45 °C after the mix was filtered and washed by deionized water (100 mL × 3). The solid was separated by silica gel column chromatography to give pure product (purity > 95%).

### 4β-*S*-(pyridine-2)-4-deoxy-podophyllotoxin (3S)

yield 75%, white needle, purity 96% (by HPLC); ^1^H NMR (400 MHz, CDCl_3_, δ): 8.40 (d, *J* = 4.0 Hz, 1H), 7.52 (t, *J* = 4.0 Hz, 1H), 7.18 (d, *J* = 8.0 Hz, 1H), 7.05 (t, *J* = 4.0 Hz, 1H), 6.95 (s, 1H), 6.46 (s, 1H), 6.33 (s, 2H), 5.95 (d, *J* = 8.0 Hz, 2H), 5.57 (d, *J* = 4.0 Hz, 1H), 4.60 (d, *J* = 4.0 Hz, 1H), 4.34 (t, *J* = 8.0 Hz, 1H), 3.86 (t, *J* = 8.0 Hz, 1H), 3.80 (s, 3H), 3.76 (s, 6H), 3.25-3.21 (m, 2H); ^13^C NMR (101 MHz, CDCl_3_, δ): 174.83, 157.95, 152.50 (2C), 147.99, 147.89, 147.25, 137.05, 136.37, 135.70, 132.37, 128.41, 121.51, 120.25, 110.09, 109.84, 108.23 (2C), 101.46, 71.01, 60.76, 56.21 (2C), 45.63, 43.77, 42.50, 37.12; ESI-MS: calc’d for C_27_H_25_NO_7_S [M + H]^+^: 508.13. FOUND 508.14. Elemental analysis calcd (%): C 63.89, H 4.94, O 22.07, N 2.76, S 6.32. FOUND C 63.85, H 4.91, O 22.12, N 2.78, S 6.34.

### 4β-*S*-(pyrimidine-2)-4-deoxy-podophyllotoxin (4S)

yield 51%, yellow amorphous solid, purity 96% (by HPLC); ^1^H NMR (400 MHz, CDCl_3_, δ): 8.57 (d, *J* = 4.0 Hz, 2H), 7.08 (t, *J* = 4.0 Hz, 1H), 6.97 (s, 1H), 6.48 (s, 1H), 6.34 (s, 2H), 5.97 (d, *J* = 8.0 Hz, 2H), 5.45 (d, *J* = 4.0 Hz, 1H), 4.62 (d, *J* = 4.0 Hz, 1H), 4.39 (t, *J* = 8.0 Hz, 1H), 3.88 (t, *J* = 8.0 Hz, 1H), 3.81 (s, 3H), 3.77 (s, 6H), 3.30-3.23 (m, 2H); ^13^C NMR (101 MHz, CDCl_3_, δ): 174.45, 171.37, 157.45 (2C), 152.54 (2C), 148.10, 147.38, 137.22, 135.57, 132.48, 127.54, 117.38, 110.15, 109.92, 108.38 (2C), 101.52, 70.68, 60.73, 56.26 (2C), 47.13, 43.75, 42.40, 37.01; ESI-MS: calc’d for C_26_H_24_N_2_O_7_S [M + H]^+^: 508.13 FOUND 508.31. Elemental analysis calcd (%): C 61.41, H 4.76, O 22.02, N 5.51, S 6.31. FOUND C 61.01, H 4.26, O 22.31, N 5.80, S 6.66.

### 4β-*S*-(benzothiazole-2)-4-deoxy-podophyllotoxin (5S)

yield 67%, white power, purity 97% (by HPLC); ^1^H NMR (400 MHz, CDCl_3_, δ): 7.86 (d, *J* = 8.0 Hz, 1H), 7.80 (d, *J* = 8.0 Hz, 1H), 7.45 (t, *J* = 8.0 Hz, 1H), 7.35 (t, *J* = 8.0 Hz, 1H), 7.01 (s, 1H), 6.50 (s, 1H), 6.33 (s, 2H), 6.99 (d, *J* = 8.0 Hz, 2H), 5.77 (d, *J* = 4.0 Hz, 1H), 4.63 (d, *J* = 4.0 Hz, 1H), 4.47 (t, *J* = 8.0 Hz, 1H), 3.99 (t, *J* = 8.0 Hz, 1H), 3.82 (s, 3H), 3.78 (s, 6H), 3.43-3.34 (m, 1H), 3.24-3.19 (m, 1H); ^13^C NMR (101 MHz, CDCl_3_, δ): 174.29, 165.32, 152.60 (2C), 148.43 (2C), 147.49, 135.28, 135.18, 132.73, 126.95, 126.31, 124.85 (2C), 121.40, 121.30, 110.15, 110.01, 108.31 (2C), 101.65, 70.79, 60.76, 56.28 (2C), 49.64, 43.74, 42.58, 37.14; ESI-MS: calc’d for C_29_H_25_NO_7_S_2_ [M + H]^+^: 563.82. FOUND 564.02. Elemental analysis calcd (%): C 61.80, H 4.47, O 19.87, N 2.49, S 11.38. FOUND C 60.97, H 4.88, O 20.07, N 2.21, S 11.67.

### 4β-*S*-(purine-6)-4-deoxy-podophyllotoxin (6S)

yield 60%, white power, purity 95% (by HPLC); ^1^H NMR (300 MHz, CDCl_3_, δ): 6.87 (s, 1H), 6.54 (s, 1H), 6.27 (s, 2H), 5.99 (d, *J* = 9.0 Hz, 2H), 5.29 (s, 1H), 4.86 (d, *J* = 9.0 Hz, 1H), 4.59 (d, *J* = 9.0 Hz, 1H), 4.37 - 4.32 (m, 1H), 3.79 (s, 3H), 3.73 (s, 6H), 3.28 (dd, *J*_1_ = 16.0 Hz, *J*_2_ = 6.0 Hz, 1H), 3.212-3.177 (m, 1H); ^13^C NMR (75 MHz, CDCl_3_, δ): 175.03 (2C), 152.55 (2C), 148.53, 147.47 (2C), 137.14, 135.05 (2C), 131.94, 131.85, 110.48 (2C), 108.97, 108.16 (2C), 67.61, 66.72, 60.74, 56.23 (2C), 53.42, 47.258, 43.89, 40.47, 38.27; ESI-MS: calc’d for C_27_H_24_N_4_O_7_S [M + H]^+^: 549.13. FOUND 549.14. Elemental analysis calcd (%): C 59.78, H 4.66, O 19.87, N 9.96, S 5.70. FOUND C 59.01, H 4.15, O 20.79, N 9.24, S 5.33.

### 4β-*S*-(pyridine-2)-4-deoxy-4′-demethyl-podophyllotoxin (3′S)

yield 80%, white power, purity 97% (by HPLC); ^1^H NMR (400 MHz, CDCl_3_, δ): 8.40 (d, *J* = 4.0 Hz, 1H), 7.52 (t, *J* = 8.0 Hz, 1H), 7.18 (d, *J* = 8.0 Hz, 1H), 7.05 (t, *J* = 8.0 Hz, 1H), 6.94 (s, 1H), 6.46 (s, 1H), 6.34 (s, 2H), 5.96 (d, *J* = 12.0 Hz, 2H), 5.56 (d, *J* = 4.0 Hz, 1H), 5.35 (s, 1H), 4.59 (d, *J* = 4.0 Hz, 1H), 4.33 (t, *J* = 8.0 Hz, 1H), 3.85 (t, *J* = 8.0 Hz, 1H), 3.79 (s, 6H), 3.33-3.20 (m, 2H); ^13^C NMR (101 MHz, CDCl_3_, δ): 174.89, 157.98, 149.29, 147.89, 147.21, 146.34 (2C), 136.37, 133.92, 132.55, 131.18, 128.40, 121.52, 120.24, 110.04, 109.85, 107.90 (2C), 101.44, 70.98, 56.43 (2C), 45.64, 43.61, 42.61, 37.05; ESI-MS: calc’d for C_26_H_23_NO_7_S [M + H]^+^: 494.21. FOUND 494.00 Elemental analysis calcd (%): C 63.27, H 4.70, O 22.69, N 2.84, S 6.50. FOUND C 63.00, H 4.54, O 22.17, N 2.34, S 6.99.

### 4β-*S*-(pyrimidine-2)-4-deoxy-4′-demethyl-podophyllotoxin (4′S)

yield 34%, white power, purity 95% (by HPLC); ^1^H NMR (300 MHz, CDCl_3_, δ): 8.46 (d, *J* = 7.2 Hz, 1H), 7.59 (t, *J* = 6.9 Hz, 1H), 7.25 (d, *J* = 7.8 Hz, 1H), 7.12 (t, *J* = 6.0 Hz, 1H), 6.99 (s, 1H), 6.51 (s, 1H), 6.38 (s, 2H), 6.00 (d, *J* = 7.2 Hz, 2H), 5.64 (t, *J* = 6.0 Hz, 1H), 4.64 (d, *J* = 4.2 Hz, 1H), 4.39 (t, *J* = 8.1 Hz, 1H), 3.89 (t, *J* = 9.0 Hz, 1H), 3.83 (s, 6H), 3.35-3.24 (m, 2H); ^13^C NMR (75 MHz, CDCl_3_, δ): 175.17, 158.16, 149.39, 148.17, 147.46, 146.61, 136.85, 134.15, 132.82, 131.40, 128.56, 121.89, 120.56, 110.31, 110.11, 108.13 (2C), 101.71, 71.23, 56.67 (2C), 46.03, 43.84, 42.85, 37.31; ESI-MS: calc’d for C_25_H_22_N_2_O_7_S [M + H]^+^: 496.15. FOUND 496.00. Elemental analysis calcd (%): C 60.72, H 4.48, O 22.65, N 5.66, S 6.48. FOUND C 61.46, H 4.87, O 22.15, N 5.95, S 6.72.

### 4β-*S*-(benzothiazole-2)-4-deoxy-4′-demethyl-podophyllotoxin (5′S)

yield 41%, white power, purity 95% (by HPLC); ^1^H NMR (400 MHz, CDCl_3_, δ): 7.88 (d, *J* = 8.0 Hz, 1H), 7.80 (d, *J* = 8.0 Hz, 1H), 7.46 (t, *J* = 8.0 Hz, 1H), 7.35 (t, *J* = 8.0 Hz, 1H), 7.27 (s, 1H), 6.56 (s, 1H), 6.40 (s, 2H), 6.00 (d, *J* = 8.0 Hz, 2H), 5.59 (d, *J* = 4.0 Hz, 1H), 4.65 (d, *J* = 4.0 Hz, 1H), 4.34-4.25 (m, 2H), 3.80 (s, 6H), 3.13-3.02 (m, 1H), 2.96-2.91 (m, 1H); ^13^C NMR(101 MHz, CDCl_3_, δ): 174.01, 164.61, 152.58, 147.91, 147.52, 146.51 (2C), 135.59, 133.82, 132.90, 130.43, 128.05, 126.40, 124.91, 121.66, 121.22, 110.06, 109.02, 107.43 (2C), 101.64, 71.55, 56.34 (2C), 49.75, 47.69, 43.90, 41.11; ESI-MS: calc’d for C_28_H_23_NO_7_S_2_ [M + H]^+^: 550.07; FOUND 550.10. Elemental analysis calcd (%): C 61.19, H 4.22, O 20.38, N 2.55, S 11.67. FOUND C 61.27, H 4.55, O 20.72, N 2.16, S 11.43.

### 4β-*S*-(purine-6)-4-deoxy-4′-demethyl-podophyllotoxin (6′S)

yield 55%, white power, purity 98% (by HPLC); ^1^H NMR (400 MHz, DMSO): δ 8.24 (s, 1H), 6.91 (s, 1H), 6.48 (s, 1H), 6.19 (s, 2H), 5.98 (d, *J* = 8.0 Hz, 2H), 5.42 (d, *J* = 8.0 Hz, 1H), 4.71 (dd, *J* = 4.0 Hz, 1H), 4.47 (d, *J* = 8.0 Hz, 1H), 4.31 (t, J = 8.0 Hz, 1H), 4.15 (t, *J* = 8.0 Hz, 1H), 3.59 (s, 6H), 3.24-3.19 (m, 1H), 2.79 - 2.70 (m, 1H); ^13^C NMR (101 MHz, DMSO): δ 175.46 (2C), 147.54, 147.51 (2C), 146.63 (2C), 135.08, 133.69 (2C), 132.04, 130.72 (2C), 110.20, 109.81, 108.82 (2C), 101.51, 68.11, 65.41, 56.42 (2C), 55.36, 43.41, 38.65; ESI-MS: calc’d for C_26_H_22_N_4_O_7_S [M + H]^+^: 535.08. FOUND 535.12. Elemental analysis calcd (%): C 58.48, H 4.15, O 20.95, N 10.48, S 6.00. FOUND C 58.08, H 4.55, O 20.84, N 10.93, S 6.60.

### General procedure for the preparation of Compound 1N-6N, 1′N -6′N

PTOX (2.07 g, 5 mmol) or DMEP (2.00 g, 5 mmol), aqueous hydrogen bromide (30%, 3 mmol) were mixed in 10 mL dichloromethane. All reactions were stirred at room temperature for 24 h and monitored by TLC. The resulting mixture was washed with saturated aqueous sodium bicarbonate (20 mL × 2), and water (20 mL × 2). The organic layer was dried over anhydrous Na_2_SO_4_, and then the solution was removed by rotary evaporation to get crude 4-Br-4-deoxypodophyllotoxin or 4-Br-4-deoxy-4′-demethylepipodophyllotoxin (yield > 85%). The above products (2 mmol) were dissolved into 15 ml dichloromethane respectively, followed by 3 mmol aromatic amines and 5 g BaCO_3_. The mixture was stirred for overnight. The resulting turbid liquid was filtered, and the filtrate was collected and dried by rotary evaporation. Silica gel column chromatography was used to purify the crude product, recrystallization was done by slow diffusion of diethyl ether into a methanol solution of Series N compounds (yield: 30%–50%).

### 4β-*NH*-(1,2,4-trizole-3)-4-deoxy-podophyllotoxin (1N)

yield 86%, white power, purity 97% (by HPLC); ^1^H NMR (400 MHz, CDCl_3_): δ 6.83 (s, 1H), 6.52 (s, 1H), 6.27 (s, 2H), 5.99 (d, *J* = 16 Hz, 2H), 4.60 (dd, *J* = 4.0 Hz, 2H), 4.37 (t, *J* = 4.0 Hz, 1H), 4.24 (t, *J* = 4.0 Hz, 1H), 3.80 (s, 3H), 3.74 (s, 6H), 3.71 (t, *J* = 4.0 Hz, 1H), 3.39-3.34 (dd, *J* = 8.0 Hz, 1H), 2.89-2.80 (m, 1H); ^13^C NMR (101 MHz, CDCl_3_): δ 174.84, 158.60, 152.72 (3C), 148.61, 147.68, 137.26, 135.70, 132.54, 127.55, 110.42, 110.09, 108.46 (2C), 101.88, 70.75, 61.02, 56.46 (2C), 49.10, 43.89, 42.42, 37.48; ESI-MS: calc’d for C_24_H_24_N_4_O_7_ [M+H]^+^: 481.00. FOUND 481.00 Elemental analysis calcd (%): C 59.99, H 2.03, O 23.31, N 11.66. FOUND C 59.32, H 2.71, O 23.65, N 11.26.

### 4β-*NH*-(1,3,4-thiodiazole-2)-4-deoxy-podophyllotoxin (2N)

yield 54%, white power, purity 96% (by HPLC); ^1^H NMR (400 MHz, CDCl_3_): δ 6.82 (s, 1H), 6.52 (s, 1H), 6.27 (s, 2H), 5.98 (d, *J* = 3.6 Hz, 2H), 5.30 (s, 1H), 5.46 (dd, 2H, *J* = 4.0 Hz), 4.36 (t, 1H, *J* = 8.0 Hz), 4.24 (t, 1H, *J* = 8.0 Hz), 3.80 (s, 3H), 3.74 (s, 6H), 3.72 (t, *J* = 8.0 Hz, 1H), 3.38 (m, *J* = 4.0 Hz, 1H), 2.88-2.81 (m, 1H); ^13^C NMR(101 MHz, CDCl_3_): δ 175.06, 152.51 (2C), 148.13, 147.03, 137.14, 135.33, 132.13, 130.43, 110.41, 109.18, 108.24 (2C), 104.89, 101.39, 71.31, 71.25, 67.76, 60.73, 56.23 (2C), 43.86, 41.19, 38.43; ESI-MS: calc’d for C_24_H_23_N_3_O_7_S: 498.00. FOUND 498.00. Elemental analysis calcd (%): C 57.94, H 4.66, O 22.51, N 8.45. FOUND C 57.31, H 4.24, O 22.72, N 8.61.

### 4β-*NH*-(pyridine-2)-4-deoxy-podophyllotoxin (3N)

yield 47%, white power, purity 98% (by HPLC); ^1^H NMR (300 MHz, CD_3_OD): δ 8.82 (d, *J* = 6.3 Hz, 2H), 7.04-7.00 (m, 2H), 6.83 (t, *J* = 7.8 Hz,1H), 6.51 (s, 2H), 6.28 (s, 2H), 5.98 (s, 2H), 5.07 (s, 1H), 4.63 (d, *J* = 4.5 Hz, 3H), 3.73 (s, 6H), 3.71 (s, 3H), 3.04 (m, 2H); ^13^C NMR(75 MHz, CD_3_OD): δ 153.06, 151.12, 149.07, 147.71, 141.95, 138.81 (2C), 137.02, 131.29, 125.62, 114.58, 113.09, 109.58, 108.27, 107.02 (2C), 101.85, 59.86, 55.37 (2C), 52.83, 51.70, 42.16, 29.57, 25.74; ESI-MS: calc’d for C_27_H_26_N_2_O_7_ [M + H]^+^: 491.04. FOUND 491.13 Elemental analysis calcd (%): C 66.11, H 5.34, O 22.83, N 5.17. FOUND C 66.20, H 5.17, O 22.07, N 5.98.

### 4β-*NH*-(pyrimidine-2)-4-deoxy-podophyllotoxin (4N)

yield 52%, white power, purity 95% (by HPLC); ^1^H NMR (300 MHz, CDCl_3_, δ): 8.15 (s, 2H), 6.81 (s, 1H), 6.87 (s, 1H), 6.60 (s, 1H), 6.50 (s, 1H), 6.31 (s, 2H), 5.95 (d, *J* = 8.4 Hz, 2H), 5.33 (s, 1H), 4.60 (s, 1H), 4.39 (t, *J* = 0.6 Hz, 1H), 3.80 (s, 3H), 3.74 (s, 6H), 3.04 (s, 3H); ^13^C NMR(75 MHz, CDCl_3_, δ): 174.94, 161.65, 158.20 (2C), 152.83 (2C), 148.51, 147.79, 135.27, 132.39, 130.01, 111.93, 110.14, 109.72, 108.62, 108.48 (2C), 101.77, 69.63, 60.98, 56.45 (2C), 50.27, 44.03, 42.13, 38.29; ESI-MS: calc’d for C_26_H_25_N_3_O_7_ [M + H]^+^: 492.20. FOUND 492.17 Elemental analysis calcd (%): C 63.54, H 5.13, O 22.79, N 8.55. FOUND C 63.11, H 5.83, O 22.51, N 8.60.

### 4β-*NH*-(pyrimidine-2)-4-deoxy-podophyllotoxin (5N)

yield 39%, white power, purity 95% (by HPLC); ^1^H NMR (400 MHz, CDCl_3_, δ): 7.62 (d, *J* = 8.0 Hz, 1H), 7.57 (d, *J* = 8.0 Hz, 1H), 7.36 (t, *J* = 8.0 Hz, 1H), 7.19 (t, *J* = 4.0 Hz, 1H), 6.88 (s, 1H), 6.51 (s, 1H), 6.30 (s, 2H), 5.97 (d, *J* = 8.0 Hz, 2H), 5.39 (s, 1H), 4.61 (d, *J* = 4.0 Hz, 1H), 4.50 (t, *J* = 4.0 Hz, 1H), 4.02 (t, *J* = 8.0 Hz, H), 3.80 (s, 3H), 3.75 (s, 6H), 3.11-3.07 (m, 2H); ^13^C NMR(101 MHz, CDCl_3_, δ): 174.25, 165.66, 152.63 (2C), 148.68, 147.72 (2C), 137.27, 134.70, 132.48, 128.27, 126.61, 123.00, 121.15, 118.83 (2C), 110.12, 109.16, 108.19 (2C), 101.69, 69.00, 60.75, 56.24 (2C), 53.91, 43.69, 41.92, 37.84; ESI-MS: calc’d for C_29_H_26_N_2_O_7_S [M + H]^+^: 547.00. FOUND 547.12 Elemental analysis calcd (%): C 63.72, H 4.79, O 20.49, N 5.13, S 5.87. FOUND C 63.33, H 4.18, O 20.03, N 5.87, S 5.96.

### 4β-*NH*-(pyrimidine-2)-4-deoxy-podophyllotoxin (6N)

yield 34%, white power, purity 95% (by HPLC); ^1^H NMR (400 MHz, CDCl_3_, δ): 6.87 (s, 1H), 6.54 (s, 1H), 6.27 (s, 2H), 5.99 (d, *J* = 12.0 Hz, 2H), 5.29 (s, 1H), 4.86 (d, *J* = 4.0 Hz, 1H), 4.60 (d, *J* = 4.0 Hz, 1H), 4.40-4.32 (m, 2H), 3.79 (s, 3H), 3.73 (s, 6H), 3.29-3.24 (m, 1H), 2.87-2.78 (m, 1H); ^13^C NMR (101 MHz, CDCl_3_, δ): 175.03 (2C), 152.55 (2C), 148.53 (2C), 147.47, 137.15, 135.05 (2C), 131.94, 131.85, 110.48 (2C), 108.97, 108.16 (2C), 101.57, 67.61, 66.72, 60.74, 56.23 (2C), 53.42, 43.89, 40.47, 38.27; ESI-MS: calc’d for C_27_H_25_N_5_O_7_ [M + H]^+^: 532.00. FOUND 532.00 Elemental analysis calcd (%): C 61.01, H 4.74, O 21.07, N 13.18. FOUND C 61.42, H 4.34, O 21.55, N 13.61.

### 4β-*NH*-(1,2,4-trizole-3)-4-deoxy-4′-demethyl-podophyllotoxin (1′N)

yield 27%, white power, purity 95% (by HPLC); ^1^H NMR(400 MHz, CD_3_OD, CD_3_Cl): δ 6.87 (s, 1H), 6.50 (s, 1H), 6.28 (s, 2H), 5.98 (d, 2H, *J* = 8.0 Hz), 5.805 (d, 1H, *J* = 1.8 Hz), 5.439 (s, 1H), 4.691 (d, 1H), 4.618 (d, 1H, *J* = 2.4 Hz, 4.522 (t, 1H *J* = 4.5 Hz,), 3.896 (t, 1H, *J* = 4.8 Hz), 3.796 (s, 6H), 3.379 (t, 1H, *J* = 2.1Hz), 3.190 (dd, 1H, *J* = 2.4Hz); ^13^C NMR (101 MHz, CD_3_OD, CD_3_Cl): δ 180.22, 152.11, 151.00, 150.94, 136.15, 134.58, 134.43, 134.38, 113.90, 112.97, 111.85, 111.83, 105.30, 75.14, 75.04, 72.12, 67.42, 59.65 (2C), 57.26, 47.52, 45.18, 42.40; ESI-MS: calc’d for C_23_H_22_N_4_O_7_ [M + H]^+^: 467.00. FOUND 467.00 Elemental analysis calcd (%): C 59.22, H 4.75, O 24.01, N 12.01. FOUND C 59.10, H 4.21, O 24.64, N 12.89.

### 4β-*NH*-(1,3,4-thiodizole-2)-4-deoxy-4′-demethyl-podophyllotoxin (2′N)

yield 30%, white power, purity 96% (by HPLC); ^1^H NMR(400 MHz, CDCl_3_): δ 6.82 (s, 1H), 6.51 (s, 1H), 6.27 (s, 2H), 5.98 (d, *J* = 16.0 Hz, 2H), 5.38 (s, 1H), 4.59 (dd, *J* = 4.0 Hz, 2H), 4.34 (t, *J* = 4.0 Hz, 1H), 4.23 (t, *J* = 8.0 Hz, 1H), 3.76(s, 6H), 3.71 (t, *J* = 8.0 Hz, 1H), 3.36-3.32 (m, 1H), 2.87-2.78 (m, 1H); ^13^C NMR (101 MHz, CDCl_3_): δ 175.14, 148.13, 146.99 (2C), 146.35, 133.96, 132.29, 130.82, 130.43, 110.41, 109.14, 107.89 (2C), 101.37, 71.32, 71.24, 67.76, 56.44 (2C), 43.69, 41.29, 38.37, 29.67; ESI-MS: calc’d for C_23_H_21_N_3_O_7_S [M + H]^+^: 484.00. FOUND 484.00. Elemental analysis calcd (%): C 57.14, H 4.38, O 23.16, N 8.69, S 6.63. FOUND C 57.25, H 4.09, O 23.67, N 8.40, S 6.84.

### 4β-*NH*-(pyridine-2)-4-deoxy-4′-demethyl-podophyllotoxin (3′N)

yield 23%, white power, purity 95% (by HPLC); ^1^H NMR (300 MHz, CD_3_OD): δ 7.81 (d, *J* = 6.3 Hz, 2H), 6.98 (s, 2H), 6.82 (t, *J* = 6.6 Hz, 1H), 6.36 (s, 1H), 6.24 (s, 2H), 5.97 (s, 2H), 4.59 (d, *J* = 3.0 Hz, 3H), 3.78 (t, *J* = 9.0 Hz, 1H), 3.73 (s, 6H), 3.03 (s, 2H); ^13^C NMR (75 MHz, CD_3_OD): δ 171.74, 149.56, 147.46, 146.24 (2C), 140.36, 137.25, 133.29, 130.14, 130.03, 128.13, 123.96, 112.99, 111.49, 108.06, 106.58, 105.36 (2C), 100.24, 59.10, 53.96 (2C), 51.31, 50.21, 41.83, 40.71; ESI-MS: calc’d for C_26_H_24_N_2_O_7_ [M + H]^+^: 491.39. FOUND 491.16 Elemental analysis calcd (%): C 65.54, H 5.08, O 23.50, N 5.88. FOUND C 65.54, H 5.08, O 23.50, N 5.88.

### 4β-*NH*-(pyrimidine-2)-4-deoxy-4′-demethyl-podophyllotoxin (4′N)

yield 36%, white power, purity 97% (by HPLC); ^1^H NMR (300 MHz, CDCl_3_): δ 8.09 (s, 2H), 6.74 (s, 1H), 6.55 (s, 1H), 6.44 (s, 1H), 6.25 (s, 2H), 5.89 (d, *J* = 9.6 Hz, 2H), 5.27 (d, *J* = 2.7 Hz, 1H), 5.22 (s, 1H), 4.28 (t, *J* = 7.8 Hz, 1H), 4.53 (s, 1H), 4.31 (s, 1H), 3.75 (t, *J* = 5.4 Hz, 1H), 3.69 (s, 6H), 2.96 (s, 2H); ^13^C NMR (75 MHz, CDCl_3_): δ 174.98, 161.40, 158.16, 148.50, 147.73, 146.71 (2C), 134.30, 132.56, 130.73, 129.89, 111.82, 110.16, 109.64, 108.18 (2C), 101.75, 69.57, 56.65 (2C), 50.24, 43.80, 42.18, 38.17, 29.91; ESI-MS: calc’d for C_25_H_23_N_3_O_7_ [M + H]^+^: 478.10. FOUND 478.15 Elemental analysis calcd (%): C 62.89, H 4.86, O 23.46, N 8.80. FOUND C 62.23, H 4.16, O 23.33, N 8.94.

### 4β-*NH*-(benzothiazole-2)-4-deoxy-4′-demethyl-podophyllotoxin (5′N)

yield 33%, white power, purity 96% (by HPLC); ^1^H NMR (400 MHz, CDCl_3_): δ 7.61 (d, *J* = 8.0 Hz, 1H), 7.56 (d, *J* = 8.0 Hz, 1H), 7.34 (t, *J* = 8.0 Hz, 1H), 7.15 (t, *J* = 8.0 Hz, 1H), 6.88 (s, 1H), 6.51 (s, 1H), 6.31 (s, 2H), 5.9 7(d, *J* = 12.0 Hz, 2H), 5.42 (d, *J* = 4.0 Hz, 1H), 4.58 (d, *J* = 4.0 Hz, 1H), 4.49 (t, *J* = 8.0 Hz, 1H), 4.00 (t, *J* = 8.0 Hz, 1H), 3.77 (s, 6H), 3.12-2.98 (m, 2H); ^13^C NMR (101 MHz, CDCl_3_): δ 174.52 (2C), 165.41, 151.61, 148.53, 147.64, 146.46 (2C), 134.07, 132.52, 130.23, 128.76, 126.26, 122.60 (2C), 121.00, 119.36, 110.03, 109.16, 107.81 (2C), 101.63, 69.25, 56.42 (2C), 53.40, 43.52, 42.02, 37.82; ESI-MS: calc’d for C_28_H_24_N_2_O_7_S [M + H]^+^: 533.00. FOUND 533.00 Elemental analysis calcd (%): C 63.15, H 4.54, O 21.03, N 5.26, S 6.02. FOUND C 63.43, H 4.94, O 21.81, N 5.77, S 6.64.

### 4β-*NH*-(purine-6)-4-deoxy-4′-demethylepipodophyllotoxin (6′N)

yield 26%, white power, purity 97% (by HPLC); ^1^H NMR (400 MHz, DMSO): δ 8.24 (s, 1H), 6.91 (s, 1H), 6.48 (s, 1H), 6.19 (s, 2H), 5.98 (d, *J* = 8.0 Hz, 2H), 5.42 (d, *J* = 8.0 Hz, 1H), 4.71 (dd, *J* = 4.0 Hz, 1H), 4.47 (d, *J* = 8.0 Hz, 1H), 4.31 (t, J = 8.0 Hz, 1H), 4.15 (t, *J* = 8.0 Hz, 1H), 3.59 (s, 6H), 3.24-3.19 (m, 1H), 2.79 - 2.70 (m, 1H); ^13^C NMR (101 MHz, DMSO): δ 175.46 (2C), 147.54, 147.51 (2C), 146.63 (2C), 135.08, 133.69 (2C), 132.04, 130.72 (2C), 110.20, 109.81, 108.82 (2C), 101.51, 68.11, 65.41, 56.42 (2C), 55.36, 43.41, 38.65; ESI-MS: calc’d for C_26_H_23_N_5_O_7_ [M + H]^+^: 518.06. FOUND 518.00 Elemental analysis calcd (%): C 60.34, H 4.48, O 21.64, N 13.53. FOUND C 60.48, H 4.36, O 21.18, N 13.64.

### Cytotoxicity Assay

Cytotoxicity assay was performed on human lung adenocarcinoma epithelial cell line (A549), henrietta lacks strain of cancer cells (HeLa), human gastric carcinoma cell line (BGC 823), and normal human hepatocyte cell line (HL-7702). Cells (3500–13,000) in a 100 μL culture medium per well were seeded into 96-well microtest plates (Falcon, CA). A549 cell lines, HeLa cell lines and HL-7702 cell lines were cultured in DMEM supplemented with 10% CS, 100 mg/L penicillin G, 100 mg/L streptomycin. BGC 823 cell lines were cultured in RPMI medium 1640 supplemented with 10% CS, 100 mg/L penicillin G, and 100 mg/L streptomycin. The cells were incubated at 37 °C, in a humidified atmosphere with 5% CO_2_ for 48 h. For all cell lines, the microculture tetrazolium [3-(4, 5-dimethylthiazol-2-yl)-2, 5-diphenyltetrazolium bromide, MTT; Sigma, St. Louis, MO] assay was performed to measure the cytotoxic effects. Drug stock solutions (the each drug concentration was 0.5, 1, 10, 20, 50, 100, 200, and 500 μM, respectively) were prepared in DMSO and the final solvent concentration was ≤ 2% DMSO (v/v), a concentration without effect on cell replication. Initial seeding densities varied among the cell lines to ensure a final absorbance reading in control (untreated) cultures in the range 0.6–0.8 *A*_492_ units. Drug exposure was for 2 days, and the IC_50_ value, the drug concentration that reduced the absorbance by 50%, was interpolated from dose-response data. Each test was performed in triplicate, and absorbance readings varied no more than 5%. Data presented as means ± standard deviation (SD). Statistical analyses were performed by the analysis of variance (ANOVA). All statistical analyses were performed using Origin version 8.0 (GraphPad Software, OriginLab Corp., Northampton, MA, USA). Sigmoidal dose responses and non-linear regression analyses were undertaken to identify half-maximal concentrations for each of the drugs. To evaluate differences in IC_50_ concentrations, analysis of variance combined with Tukey’s multiple range test was used.

### Observation of microtubule in HeLa cells treated with Compounds 1-6S and 1-6N by immunofluorescence

HeLa cells were plated at a density of 150 000 cells/ml into 6-well plates containing 24 mm round coverslips, cultured overnight, and then treated with 1S, 1N and colchicine at different concentrations or drug vehicle (DMSO) for 12 h. Residual DMSO was less than 1.0%. Attached cells were fixed with immune staining fix solution. Cytoskeletons were incubated with primary antibody reacting with α-tubulin, washed twice, and incubated with FITC goat anti-mouse immunoglobulins. The coverslips were washed, and Hoechst 33258 to stain chromatin was added. The mixture was incubated for 30 min. After the samples were washed, they were examined and photographed using a laser scanning microscope, the images were exported by the volocity software (PerkinElmer).

### Tubulin Assembly

Purification of calf brain tubulin and chemicals were followed as previously described by Andreu^41^. Ligands were dissolved in DMSO at 20mM and kept at −80 °C. Work solutions were done in DMSO and kept at −20 °C. The 50% inhibitory ligand concentration of tubulin assembly was determined with a centrifugation assay. Tubulin was equilibrated prior to use in 3.4 M glycerol, 1 mM EGTA, 0.1 mM GTP, pH 7.0, buffer through a 25 cm × 0.9 cm Sephadex G-25 column. Aggregates were removed by a centrifugation at 90000 g × 10 min in a TLA 120 rotor at 4 °C in an Optima TLX centrifuge. Tubulin concentration was determined as described by Andreu^42^. Tubulin was kept at 4 °C, and 0.9 mM GTP and 6 mM MgCl_2_ were added to the sample. The solution was distributed in 200 μL polycarbonate tubes for the TL100 rotor. Growing concentrations of the ligands ranging from 0 to 25 μM were added to the samples (DMSO content of the samples, 2.5%), which were incubated for 30 min at 37 °C. Microtubules were separated from unassembled tubulin by a centrifugation at 90000 g × 10 min in a TLA100 rotor at 37 °C in an Optima TLX centrifuge. The supernatant containing unassembled tubulin was carefully collected and the microtubule pellet resuspended in 10 mM sodium phosphate buffer, pH 7.0, containing 1% SDS. Both supernatants and pellets were diluted 1:5 in the same buffer, and tubulin concentrations were measured fluorometrically (λexc = 280; λems = 323) using tubulin standards calibrated spectrophotometrically. The 50% inhibitory ligand concentration of tubulin assembly was determined with a centrifugation assay that measured the decrease in the concentrations of microtubules assembled in the presence of different concentrations of the compound.

### Topoisomerase II inhibition

Topoisomerase II activity was determined using kit (TopoGEN Inc., USA, Cat no. 2000H). The substrate kDNA (134 ng) and 10 μM drug were combined in assay buffer (5 × Top II assay buffer A and B) and incubated for 10 min on ice. Next, 4 U of topoisomerase II was added and the reaction was allowed to proceed for 60 min at 37 °C. The reaction was stopped by the addition of 1/10 volume of 1 mg/mL proteinase K and incubated for 15 min at 37 °C. The reaction was quenched via the addition of loading buffer (350 mM Tris, pH 6.8, 12% SDS, 0.012% bromophenol blue, 47% glycerol) and was then analyzed by electrophoresis on a 1% agarose gel in TBE buffer (89 mM Tris, 89 mM borate, and 2 mM Na-EDTA, pH 8.3) for 0.5 h at 140 V. The gel was visualized under UV illumination and photographed on an Alpha Imager.

### Flow cytometry analysis of cell cycle, apoptosis, and the reactive oxygen species (ROS)

For the cell cycle analysis, HeLa cells were treated with test compounds for 6, 12, 24 and 48 h at 37 °C in 6-well plates. Appropriate controls, in which cells were mock-treated by DMSO (<1%) for the same durations, were also set up. After being treated, all cells were trypsinized and collected, washed twice with PBS, added with 4 °C 70% ethanol (10 mL) and stored overnight at 4 °C. After this, cells were spun down at 1500 rpm for 5 min, washed once with PBS, and then treated with the mixture of 10 μL RNase (10 mg/mL), 50 μL propidium iodide (1 mg/mL) and 940 μL 1 × PBS, incubated at room temperature for 30 min in the dark. The stained cells were analyzed by flow cytometry. The result was analyzed with ModFit LT^TM^ 3.2 version software. For the apoptosis determination, after 6, 12, 24 and 48 h treatment with test compounds, HeLa cells were harvested, re-suspend in 500 μL annexin-binding buffer (10mM HEPES, 140 mM NaCl, 2.5 mM CaCl_2_, pH 7.4) at the cell density of about 10^6^ cells/mL and stained with 5 μL annexin V and 5 μL propidium iodide for 15 min at room temperature. Changes in intracellular ROS levels were determined by measuring the oxidative conversion of cell permeable 2′,7′-dichlorofluorescein diacetate (DCFH-DA) to fluorescent di-chlorofluorescein (DCF) in flow cytometry (BD Accuri C6). Cells in 6-well culture dishes were incubated with DMEM for 6, 12, 24 and 48 h in the absence or presence of test compounds. The cells were washed with DMEM and incubated with DCFH-DA (10 μM) at 37 °C for 30 min. Then DCF fluorescence of 10,000 cells was detected by flow cytometry.

### Apoptosis in HeLa Cells by Western Blot Analysis

After treatment of 1S (5 μM) and 1N (5 μM), HeLa cells were washed with PBS and collected at different intervals. Cells were lysed by adding 100 μL of RIPA buffer (50 mM Tris, pH 8.0, 150 mM NaCl, 1% TX-100, 0.5% sodium deoxycholate, 0.1% SDS) with 1% Protease Inhibitor PMSF. Each sample was then vigorously vortexed twice for 15 seconds, with a 30-minute incubation on ice following each agitation. The cellular debris was pelleted (12,000 rpm, 10 min), and then 100 μL of the protein suspension was transferred to fresh 0.5 mL tubes. The protein levels were quantified using a standard BCA (Thermo Scientific), after which the samples were diluted with deionized water to achieve equal protein concentrations for all samples. Prior to analyzing the samples, 5 × Loading buffer (350 mM Tris, pH 6.8, 12% SDS, 0.012% bromophenol blue, 47% glycerol) with 5% β-mercaptoethanol was added to each sample to achieve a final 1 × concentration, after which the samples were incubated at 95 °C for 10 minutes to denature the protein samples. 20–30 μg of protein was added to and run on a 15-well 4–20% Tris-HCl gel. The gel was equilibrated buffer (39 mM glycine, 48 mM, 0.037 g/L SDS, 200 mL/L methanol) for 5 minutes, then the protein were transferred to a PVDF membrane, which was incubated with 5% fetal bovine serum in TBST (20 mM Tris-HCl, pH 8.0, 150 mM, 0.5 mL/L Tween-20) for 1 h to block non-specific binding, and then probed for the primary antibody at a 1:1000 dilution with a blocking agent in TBST for 1 h at room temperature. The blot was washed with TBST, and then probed with a secondary antibody in TBST for 1 hour at room temperature. The blot was washed with TBST, and then The ECL detection kit (Millipore) was added to catalyze oxidation of luminol to emit chemiluminescence, and the membrane was visualized using X-ray film processor and X-ray film (Fujifilm SUPER RX-N-C).

### Cellular uptake analysis

Cellular uptake of Compound 1S-6S, 1N-6N, 1′S-6′S, and 1′N-6′N in HeLa cells was measured as follow. The cells were seeded in 6-well tissue culture plates. After overnight incubation, the cells were treated with the corresponding compounds (10 μM) for 6 h in the growing medium. Both the cell culture broth and the PBS that washed attached cells were collected at 15 mL tube. This sample was centrifuged at 12000 rpm for 30 min and the supernatant was collected. The samples was filtered with a 0.45 μm micropore filter and transferred to a sampling vial for HPLC analysis. HPLC analysis was carried out on a Waters 600 Series HPLC system, equipped with 2487 ultraviolet detector. An Akasil C18 column (5 μm, 4.6 mm × 150 mm) was used. Mobile phase was methanol per water (50:50 v per v) and the pH was adjusted to 3.00 with formic acid. The HPLC oven temperature was maintained at 45 °C, and the detection wavelength was 230 nm. The flow rate was 0.8 mL per min. Methanol and acetonitrile were high-performance liquid chromatography (HPLC) grade, and all other chemicals used for extraction and isolation were analysis grade and commercially available. The cellular uptake ratio was calculated with following equation: the cellular uptake ratio = (C _in total_ − C _extracellular_)/C _in total_ × 100%. C _extracellular_ is the compound concentration in extracellular. The reported the cellular uptake ratios are an average of three measurements. Data is the mean of three experiments and reported as mean ± SD.

## Additional Information

**How to cite this article**: Li, J.-L. *et al.* Comparison of carbon-sulfur and carbon-amine bond in therapeutic drug: 4β-*S*-aromatic heterocyclic podophyllum derivatives display antitumor activity. *Sci. Rep.* **5**, 14814; doi: 10.1038/srep14814 (2015).

## Supplementary Material

Supplementary Information

## Figures and Tables

**Table 1:**
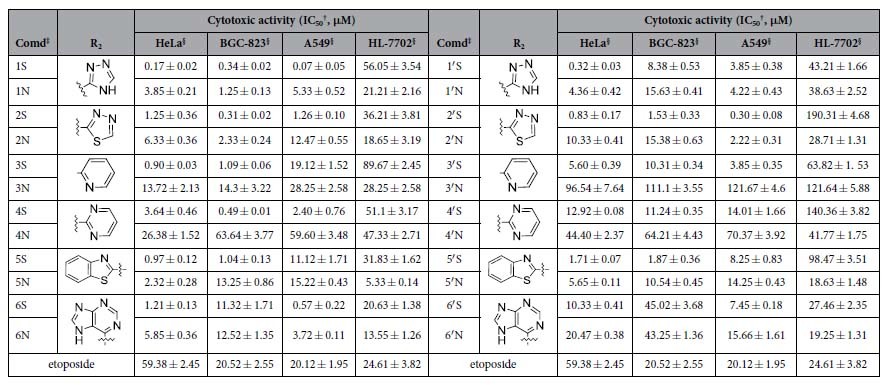
The IC_50_ values of podophyllum derivatives against tumor 16cells and human normal cells.

^†^MTT methods, drugs exposure was for 48 h.

^‡^The abbreviation of Compound.

^§^Standard deviation (SD) of triplicate samples was calculated from three independent samples, mean ± SD (n = 3).
