# Supplementary material for: Comparison of carbon-sulfur and carbon-amine bond in therapeutic drug: 4β-S-aromatic heterocyclic podophyllum derivatives display antitumor activity
Source: Sci Rep. 2015 Oct 7;5:14814. doi: 10.1038/srep14814 (PMC4595834; doi:10.1038/srep14814)
Supplement: Supplementary Information [file srep14814-s1.pdf]

Comparison of carbon-sulfur and carbon-amine bond in therapeutic drug: 4β-*S*-aromatic heterocyclic podophyllum derivatives display antitumor activity

Jian-Long Li [1, a], Wei Zhao [1, a], Chen Zhou [1, a], Ya-Xuan Zhang [a], Hong-Mei Li [a], Ya-Ling Tang [b], Xin-Hua Liang [b], Tao Chen [c], and Ya-Jie Tang [a] *,

[a] *Key Laboratory of Fermentation Engineering (Ministry of Education), Hubei Provincial Cooperative Innovation Center of Industrial Fermentation, Hubei University of Technology, Wuhan 430068, China*

[b] *State Key Laboratory of Oral Diseases West China Hospital of Stomatology (Sichuan University), Chengdu Sichuan 610041,People's Republic of China*

[c] *Key Laboratory of Systems Bioengineering (Ministry of Education), School of Chemical Engineering and Technology, Tianjin University, Tianjin 300072, China*

*Corresponding author. Tel. & Fax: +86-27-5975.0491 Email: yajietang@hotmail.com

[1] Equally contributed to this work

Supporting Information

Table S1. Calculated docking of the complex of podophyllum derivatives with tubulin/Topo II.

Compound	Energy (-kcal/mol)	H-bond	π–π packing	Compound	Energy (-kcal/mol)	H-bond	π–π packing
1S	2.11	-	Ser A178	1'S	6.51	Asp B479	Arg B503, DG D13
1N	4.03	Asn B258	Lys B352	1'N	3.04	Asp B479	Arg B503, DT F9 , DA D12, DG D13
2S	4.99	Lys B254	Leu B248	2'S	10.26	Asp B479	Arg B503, DT F9, DG D13
2N	5.72	Lys B352	Leu B255, Lys B352	2'N	6.15	Asp B479	Arg B503, DA D12, DG D13
3S	4.37	Ser A178	Ala B316	3'S	3.86	Asp B479	Arg B503, DT F9, DG D13
3N	3.87	Asn B258, Thr B353	Leu B248	3'N	5.58	Asp B479	Arg B503, DT F9, DG D13
4S	2.90	Thr A179	Lys B254	4'S	4.52	Asp B479	Arg B503, DT F9, DG D13
4N	2.45	Asn B258	Lys B352	4'N	2.69	Asp B479	Arg B503, DT F9, DG D13
5S	6.45	Lys B254	Lys B254	5'S	5.10	Asp B479	Arg B503, DT F9, DG D13
5N	6.13	-	Leu B248	5'N	4.12	Asp B479	Arg B503, DA D12, DG D13
6S	6.28	Lys B352	-	6'S	8.42	Asp B479	Arg B503, DT F9, DG D13
6N	3.14	Leu B255	-	6'N	8.10	Asp B479, DG D13	Arg B503, DA D12, DG D13
7S	-2.03	-	-	7'S	-7.23	-	-
7N	-2.37	Thr B353	-	7'N	-6.25	DC E8	DT F9, DA D12
8S	-1.49	-	-	8'S	-4.23	Lys B456	Arg B503
8N	-5.37	-	Leu B248	8'N	-6.43	-	Arg B503, DT F9
9S	-10.00	-	-	9'S	-7.26	Lys B456	Arg B503
9N	-11.04	Leu B255	-	9'N	-5.32	-	Arg B503
PTOX	4.11	Leu B248	Ala B316	VP-16	6.38	Asp B479	Arg B503, DC E8, DG D13

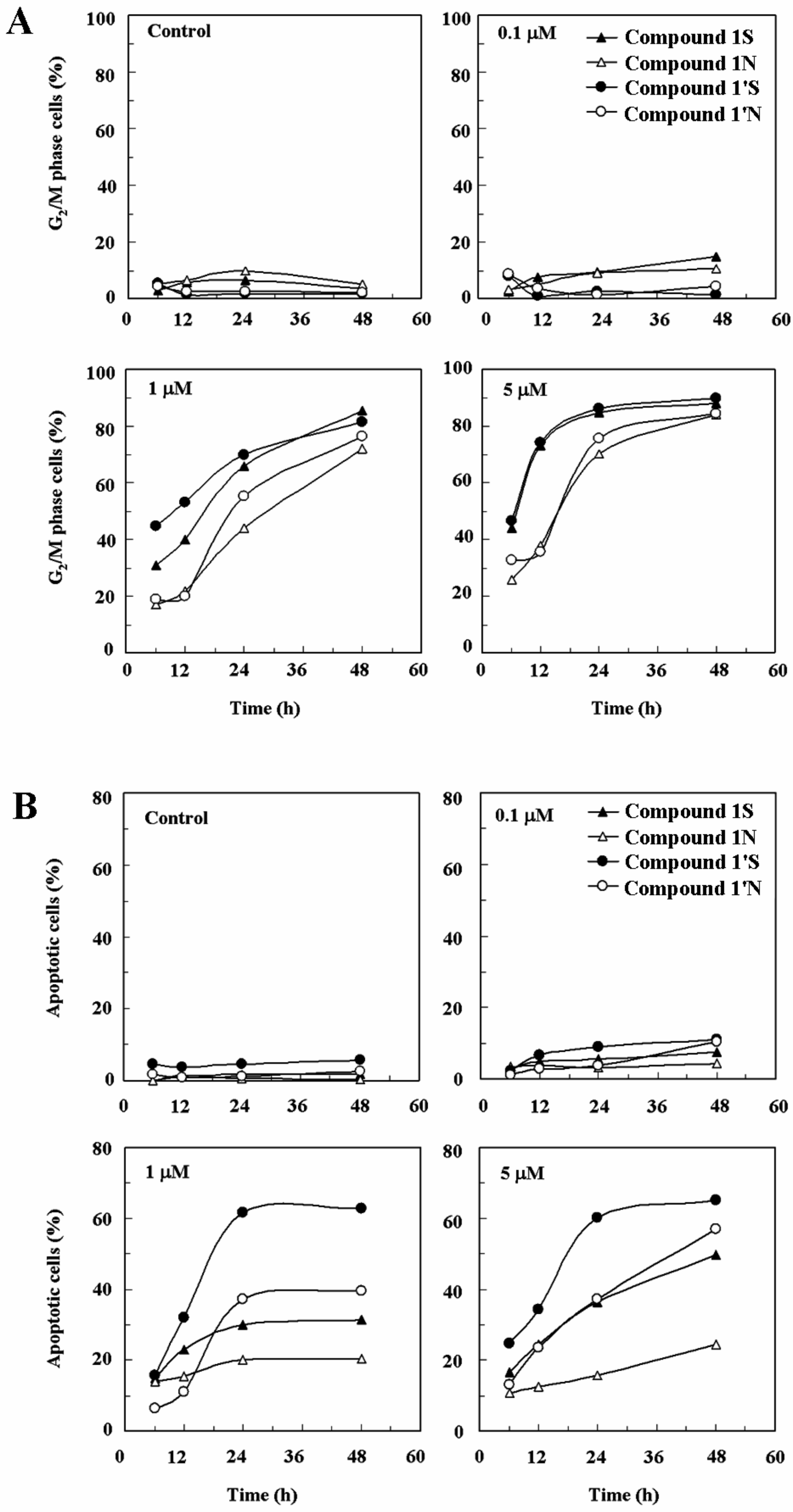
A
Control
0.1 μM
Compound 1S
Compound 1N
Compound 1'S
Compound 1'N
1 μM
5 μM
G_2/M phase cells (%)
Time (h)
B
Control
0.1 μM
Compound 1S
Compound 1N
Compound 1'S
Compound 1'N
1 μM
5 μM
Apoptotic cells (%)
Time (h)

Figure S1. Effects of podophyllum derivatives on the HeLa cell cycle arrest and apoptosis induce. (A) Compound 4β-*S*-(1, 3, 4-trizole-2)-4-deoxy-podophyllotoxin (Compound 1S), Compound 4β-*NH*-(1, 3, 4-trizole-2)-4-deoxy-podophyllotoxin (Compound 1N), 4β-*S*-(1, 3, 4-trizole-2)-4-deoxy-4'-demethylepipodophyllotoxin (Compound 1'S) and Compound 4β-*NH*-(1, 3, 4-trizole-2)-4-deoxy-4'-demethylepipodophyllotoxin (Compound 1'N) arrested cell cycle in HeLa cells in a dose- and time-dependent manner at the concentration of 0, 0.1, 1, and 5 μM for 6, 12, 24, and 48 h, respectively. Compared with the cells incubated with no drug, the treatment of Compounds 1S and 1N did not induce the G_2/M phase arrest at a lower concentration of 0.1 μM. However, the percentage of G_2/M phase cells was accumulated significantly to about 80% at 48 h when the concentration was increased to 1 μM. And then the percentage of G_2/M phase cells continuously increased to the maximum about 80-90% at the higher concentration of 5 μM after the incubation of 48 h. Corresponding to Compounds 1S and 1N, the comparison between Compound 1'S and 1'N showed the similar trend that Compound 1'S was superior than Compound 1'N to arrest the cell cycle. (B) Compound 1S, 1N, 1'S, and 1'N induced cell apoptosis in HeLa cells in a dose- and time-dependent manner at the concentration of 0, 0.1, 1, and 5 μM for 6, 12, 24, and 48 h, respectively. Symbols: the negative control without adding Compounds 1S, 1N, 1'S and 1'N (0 μM), Compound 1S (black triangle, ▲), Compound 1N (open triangle, △), Compound 1'S (black circle, ●), and Compound 1'N (open circle, ○).

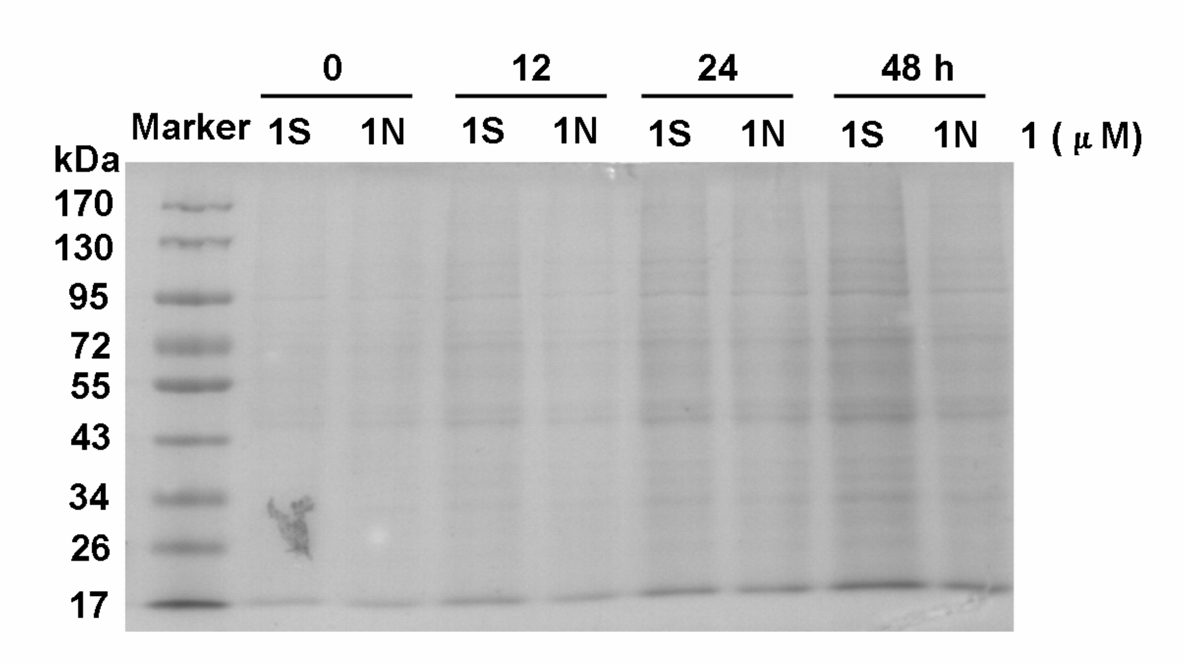

Figure S2

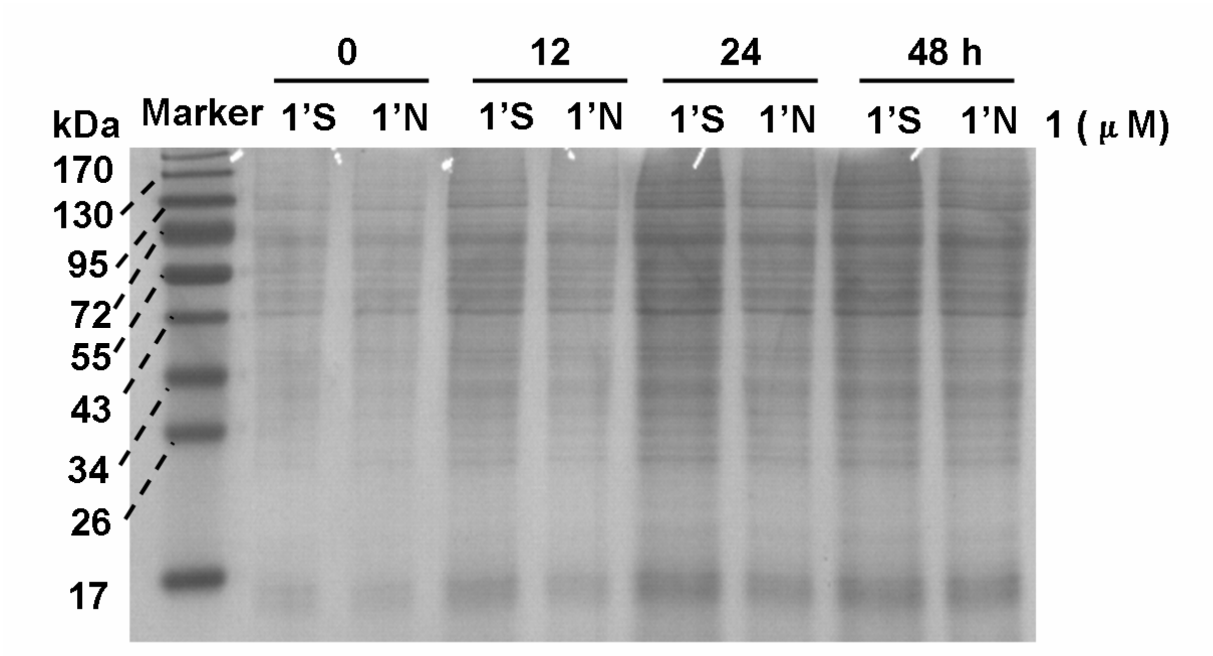

Figure S3

Calculated docking of complexes

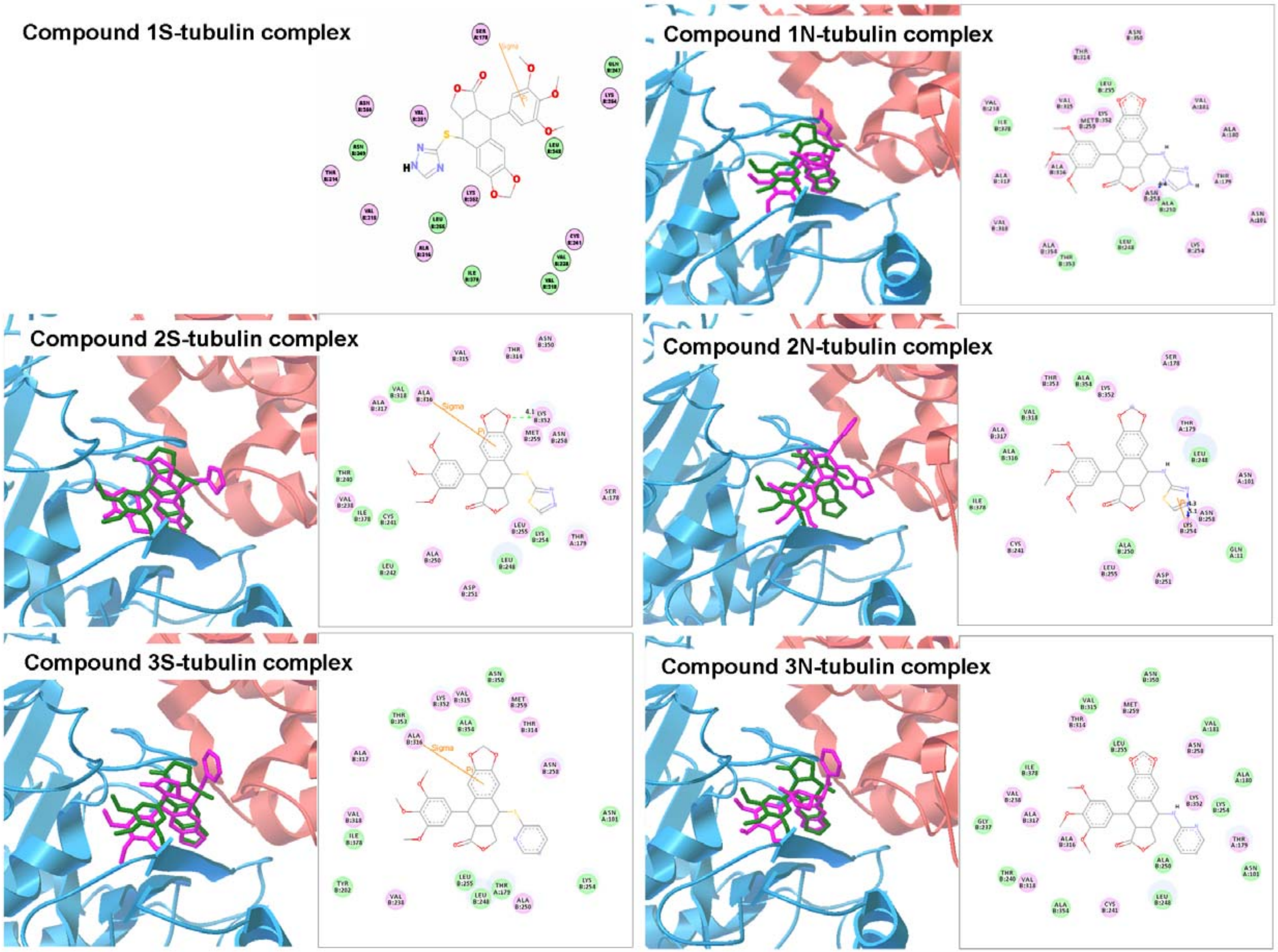

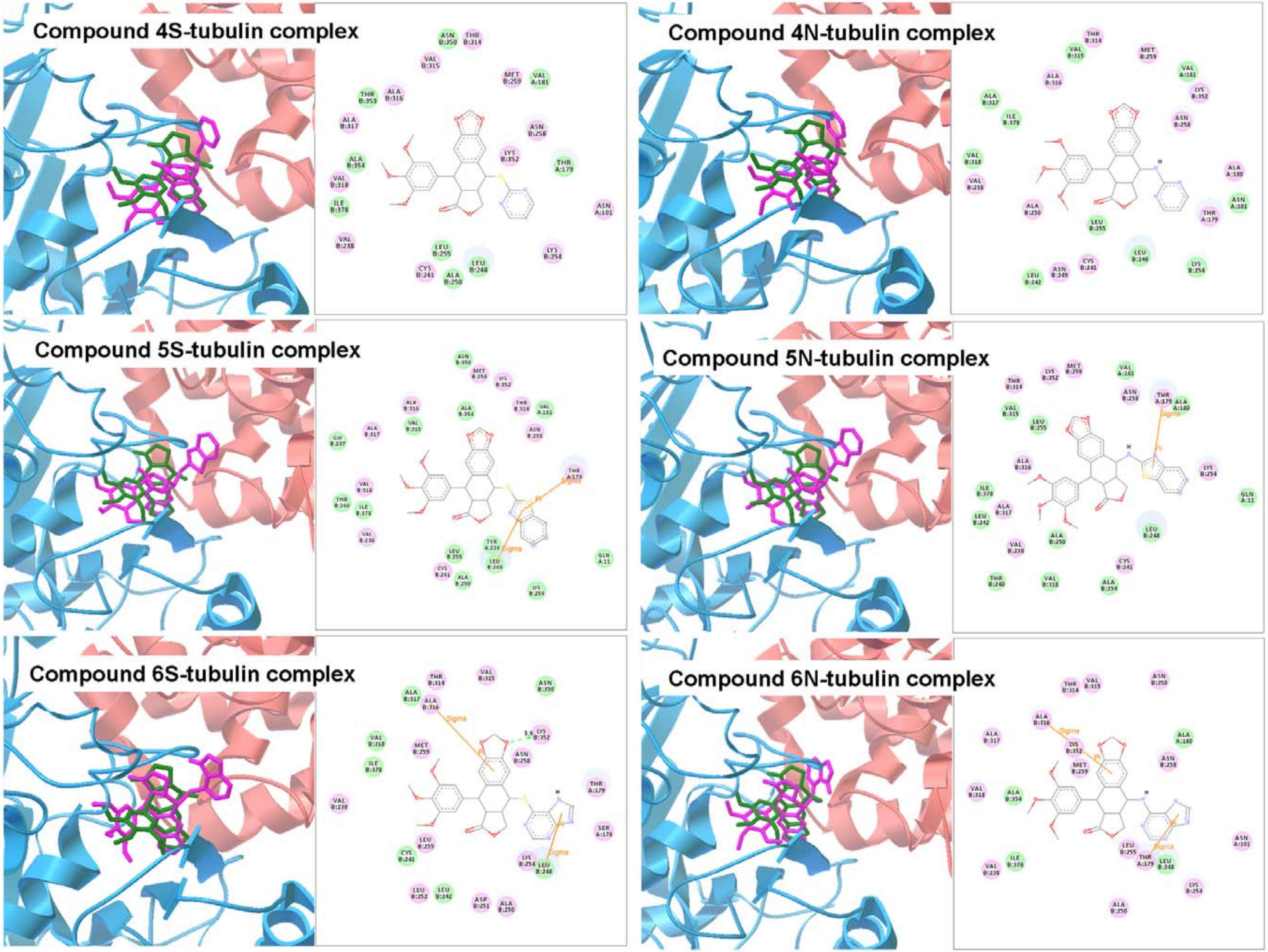

Compound 4S-tubulin complex
Compound 4N-tubulin complex
Compound 5S-tubulin complex
Compound 5N-tubulin complex
Compound 6S-tubulin complex
Compound 6N-tubulin complex

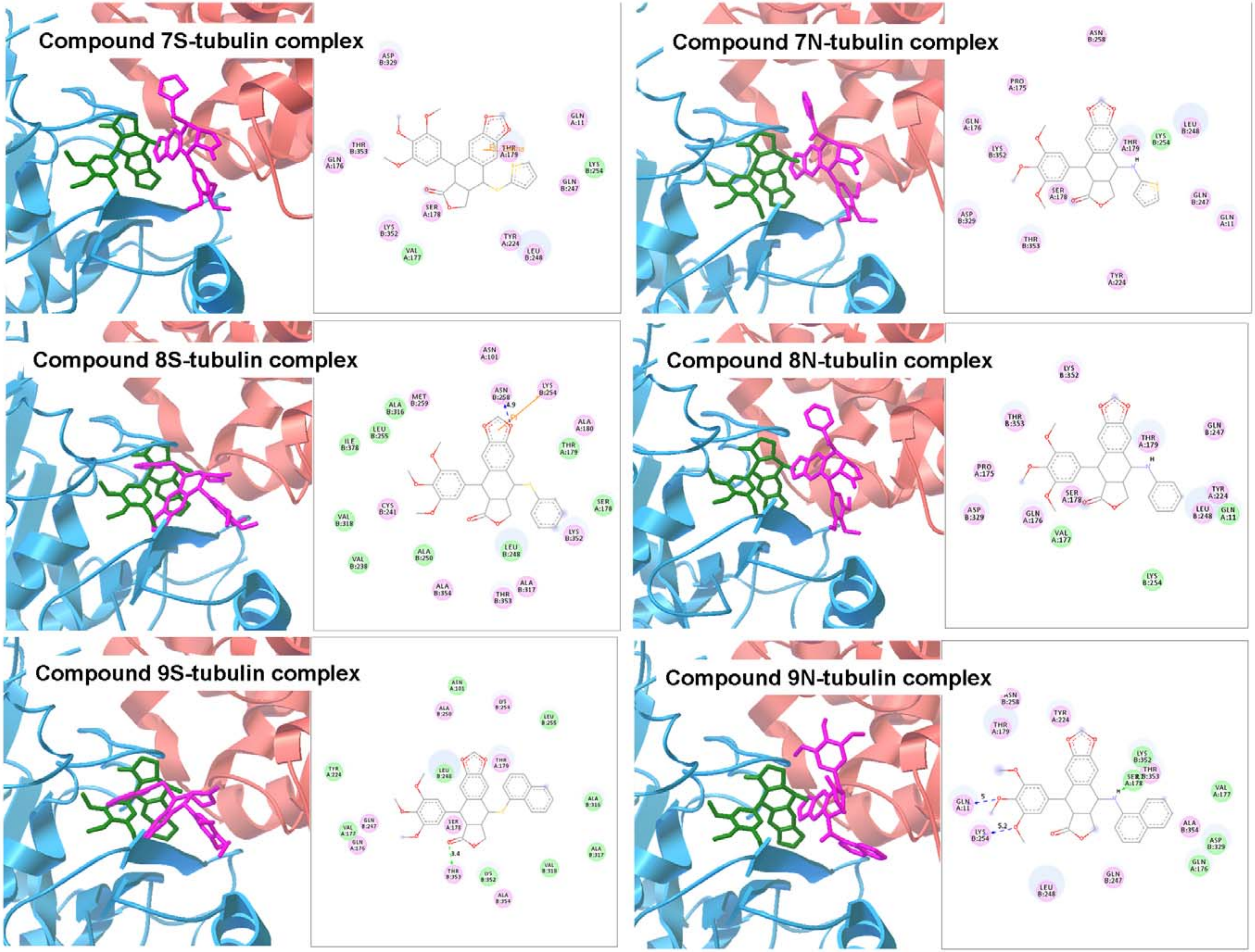
Compound 7S-tubulin complex
Compound 7N-tubulin complex
Compound 8S-tubulin complex
Compound 8N-tubulin complex
Compound 9S-tubulin complex
Compound 9N-tubulin complex

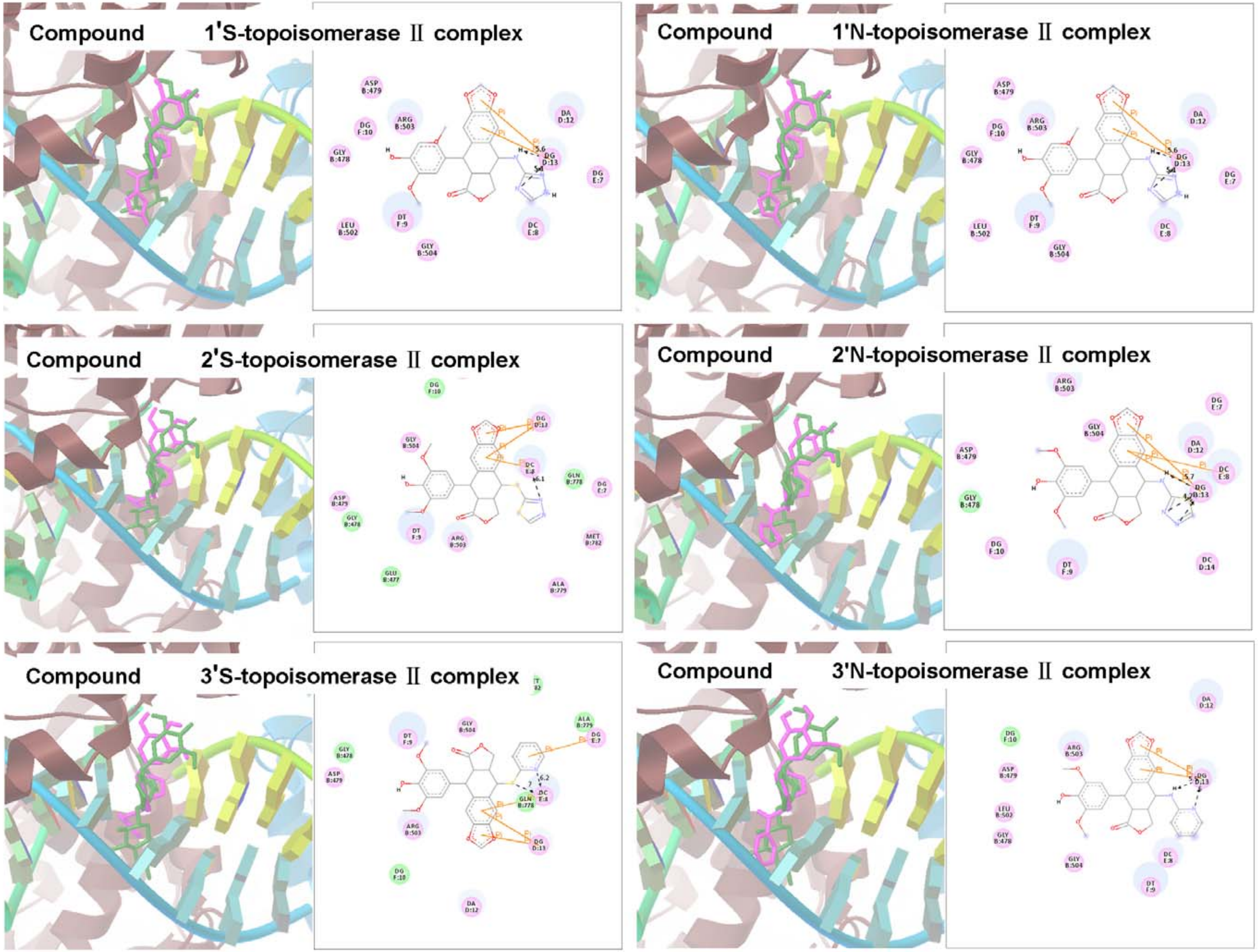
Compound 1'S-topoisomerase Ⅱ complex
Compound 1'N-topoisomerase Ⅱ complex
Compound 2'S-topoisomerase Ⅱ complex
Compound 2'N-topoisomerase Ⅱ complex
Compound 3'S-topoisomerase Ⅱ complex
Compound 3'N-topoisomerase Ⅱ complex

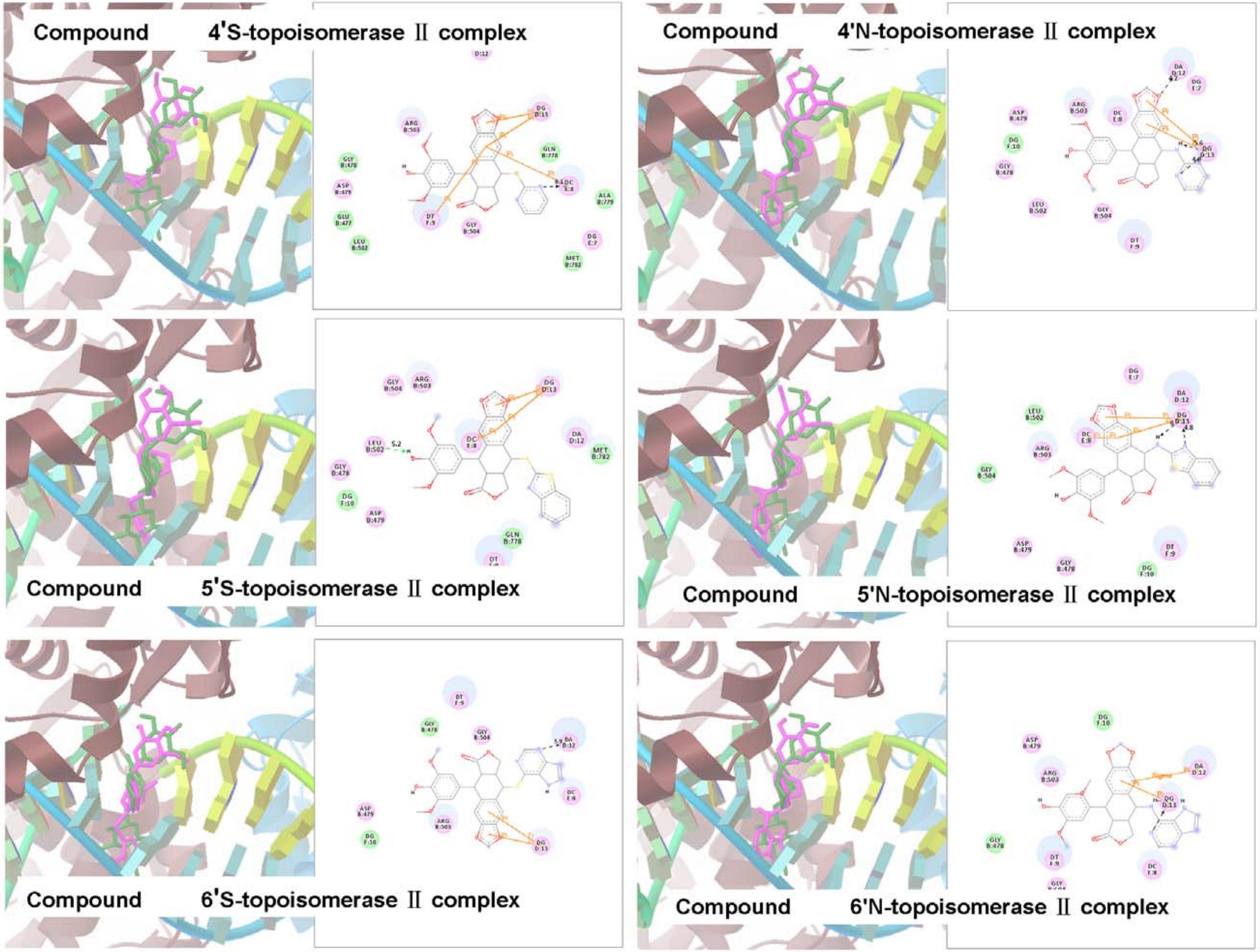
Compound 4'S-topoisomerase Ⅱ complex
Compound 4'N-topoisomerase Ⅱ complex
Compound 5'S-topoisomerase Ⅱ complex
Compound 5'N-topoisomerase Ⅱ complex
Compound 6'S-topoisomerase Ⅱ complex
Compound 6'N-topoisomerase Ⅱ complex

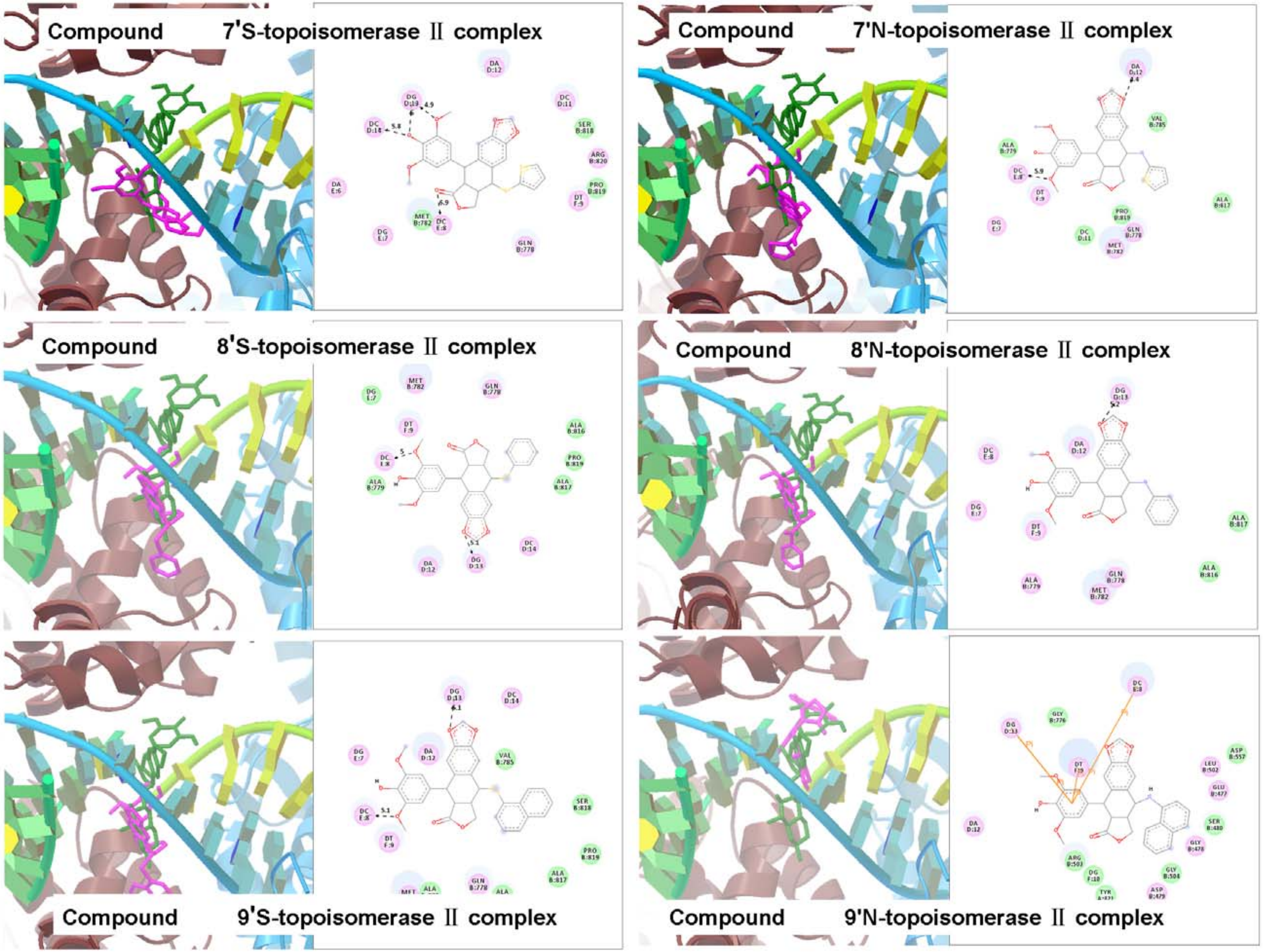
Compound 7'S-topoisomerase Ⅱ complex
Compound 7'N-topoisomerase Ⅱ complex
Compound 8'S-topoisomerase Ⅱ complex
Compound 8'N-topoisomerase Ⅱ complex
Compound 9'S-topoisomerase Ⅱ complex
Compound 9'N-topoisomerase Ⅱ complex

Copies of ^{1}H, ^{13}C NMR and 2D NMR (1H-1H COSY, HMBC, HSQC) Spectra

1. NMR and MS spectrum of compound 3S.

1.1. ^{1}H NMR spectrum of compound 3S.

1.2. ^{13}C NMR spectrum of compound 3S.

1.3. ^{1}H-^{1}H COSY spectrums for compound 3S.

1.4. HMBC spectrums for compound 3S.

1.5. HSQC spectrums for compound 3S.

1.6. MS diagram for compound 3S.

2. NMR spectrum of compound 4S.

2.1. ^{1}H NMR spectrum of compound 4S.

2.2. ^{13}C NMR spectrum of compound 4S.

2.3. ^{1}H-^{1}H COSY spectrums for compound 4S.

2.4. HMBC spectrums for compound 4S.

2.5. HSQC spectrums for compound 4S.

2.6. MS diagram for compound 4S.

3. NMR and MS spectrum of compound 5S.

3.1. ^{1}H NMR spectrum of compound 5S.

3.2. ^{13}C NMR spectrum of compound 5S.

3.3. ^{1}H-^{1}H COSY spectrums for compound 5S.

3.4. HMBC spectrums for compound 5S.

3.5. HSQC spectrums for compound 5S.

3.6. MS diagram for compound 5S.

4. NMR and MS spectrum of compound 6S.

4.1. ^{1}H NMR spectrum of compound 6S.

4.2. ^{13}C NMR spectrum of compound 6S.

4.3. ^{1}H-^{1}H COSY spectrums for compound 6S.

4.4. HMBC spectrums for compound 6S.

4.5. HSQC spectrums for compound 6S.

4.6. MS diagram for compound 6S.

5. NMR and MS spectrum of compound 3'S.

5.1. ^{1}H NMR spectrum of compound 3'S.

5.2. ^{13}C NMR spectrum of compound 3'S.

5.3. ^{1}H-^{1}H COSY spectrums for compound 3'S.

5.4. HMBC spectrums for compound 3'S.

5.5. HSQC spectrums for compound 3'S.

5.6. MS diagram for compound 3'S.

6. NMR and MS spectrum of compound 4'S.

6.1. ^{1}H NMR spectrum of compound 4'S.

6.2. ^{13}C NMR spectrum of compound 4'S.

6.3. ^{1}H-^{1}H COSY spectrums for compound 4'S.

6.4. HMBC spectrums for compound 4'S.

6.5. HSQC spectrums for compound 4'S.

6.6. MS diagram for compound 4'S.

7. NMR and MS spectrum of compound 5'S.

7.1. ^{1}H NMR spectrum of compound 5'S.

7.2. ^{13}C NMR spectrum of compound 5'S.

7.3. ^{1}H-^{1}H COSY spectrums for compound 5'S.

7.4. HMBC spectrums for compound 5'S.

7.5. HSQC spectrums for compound 5'S.

7.6. MS diagram for compound 5'S.

8. NMR spectrum of compound 6'S.

8.1. ^{1}H NMR spectrum of compound 6'S.

8.2. ^{13}C NMR spectrum of compound 6'S.

8.3. ^{1}H-^{1}H COSY spectrums for compound 6'S.

8.4. HMBC spectrums for compound 6'S.

8.5. HSQC spectrums for compound 6'S.

8.6. MS diagram for compound 6'S.

9. NMR and MS spectrum of compound 1N.

9.1. ^{1}H NMR spectrum of compound 1N.

9.2. ^{13}C NMR spectrum of compound 1N.

9.3. ^{1}H-^{1}H COSY spectrums for compound 1N.

9.4. HMBC spectrums for compound 1N.

9.5. HSQC spectrums for compound 1N.

9.6. MS diagram for compound 1N.

10. NMR spectrum of compound 2N.

10.1. ^{1}H NMR spectrum of compound 2N.

10.2. ^{13}C NMR spectrum of compound 2N.

10.3. ^{1}H-^{1}H COSY spectrums for compound 2N.

10.4. HMBC spectrums for compound 2N.

10.5. HSQC spectrums for compound 2N.

10.6. MS diagram for compound 2N.

11. NMR spectrum of compound 3N.

11.1. ^{1}H NMR spectrum of compound 3N.

11.2. ^{13}C NMR spectrum of compound 3N.

11.3. ^{1}H-^{1}H COSY spectrums for compound 3N.

11.4. HMBC spectrums for compound 3N.

11.5. HSQC spectrums for compound 3N.

11.6. MS diagram for compound 3N.

12. NMR spectrum of compound 4N.

12.1.^{1}H NMR spectrum of compound 4N.

12.2. ^{13}C NMR spectrum of compound 4N.

12.3. COSY spectrums for compound 4N.

12.4. HMBC spectrums for compound 4N.

12.5. HSQC spectrums for compound 4N.

12.6. MS diagram for compound 4N.

13. NMR and MS spectrum of compound 5N.

13.1.^{1}H NMR spectrum of compound 5N.

13.2. ^{13}C NMR spectrum of compound 5N.

13.3. COSY spectrums for compound 5N.

13.4. HMBC spectrums for compound 5N.

13.5. HSQC spectrums for compound 5N.

13.6. MS diagram for compound 5N.

14. NMR and MS spectrum of compound 6N.

14.1.^{1}H NMR spectrum of compound 6N.

14.2. ^{13}C NMR spectrum of compound 6N.

14.3. COSY spectrums for compound 6N.

14.4. HMBC spectrums for compound 6N.

14.5. HSQC spectrums for compound 6N.

13.6. MS diagram for compound 6N.

15. NMR and MS spectrum of compound 1'N.

15.1. ^{1}H NMR spectrum of compound 1'N.

15.2. ^{13}C NMR spectrum of compound 1'N.

15.3. ^{1}H-^{1}H COSY spectrums for compound 1'N.

15.4. HMBC spectrums for compound 1'N.

15.5. HSQC spectrums for compound 1'N.

15.6. MS diagram for compound 1'N.

16. NMR spectrum of compound 2'N.

16.1. ^{1}H NMR spectrum of compound 2'N.

16.2. ^{13}C NMR spectrum of compound 2'N.

16.3. ^{1}H-^{1}H COSY spectrums for compound 2'N.

16.4. HMBC spectrums for compound 2'N.

16.5. HSQC spectrums for compound 2'N.

16.6. MS diagram for compound 2'N.

17. NMR spectrum of compound 3'N.

17.1. ^{1}H NMR spectrum of compound 3'N.

17.2. ^{13}C NMR spectrum of compound 3'N.

17.3. ^{1}H-^{1}H COSY spectrums for compound 3'N.

17.4. HMBC spectrums for compound 3'N.

17.5. HSQC spectrums for compound 3'N.

17.6. MS diagram for compound 3'N.

18. NMR spectrum of compound 4'N.

18.1.^{1}H NMR spectrum of compound 4'N.

18.2. ^{13}C NMR spectrum of compound 4'N.

18.3. COSY spectrums for compound 4'N.

18.4. HMBC spectrums for compound 4'N.

18.5. HSQC spectrums for compound 4'N.

18.6. MS diagram for compound 4'N.

19. NMR and MS spectrum of compound 5'N.

19.1.^{1}H NMR spectrum of compound 5'N.

19.2. ^{13}C NMR spectrum of compound 5'N.

19.3. COSY spectrums for compound 5'N.

19.4. HMBC spectrums for compound 5'N.

19.5. HSQC spectrums for compound 5'N.

19.6. MS diagram for compound 5'N.

20. NMR and MS spectrum of compound 6'N.

20.1.^{1}H NMR spectrum of compound 6'N.

20.2. ^{13}C NMR spectrum of compound 6'N.

20.3. COSY spectrums for compound 6'N.

20.4. HMBC spectrums for compound 6'N.

20.5. HSQC spectrums for compound 6'N.

20.6. MS diagram for compound 6'N.

Copies of ^{1}H, ^{13}C NMR and 2D NMR (^{1}H-^{1}H COSY, HMBC, HSQC) Spectra

1.1. ^{1}H NMR spectrum of compound 3S.

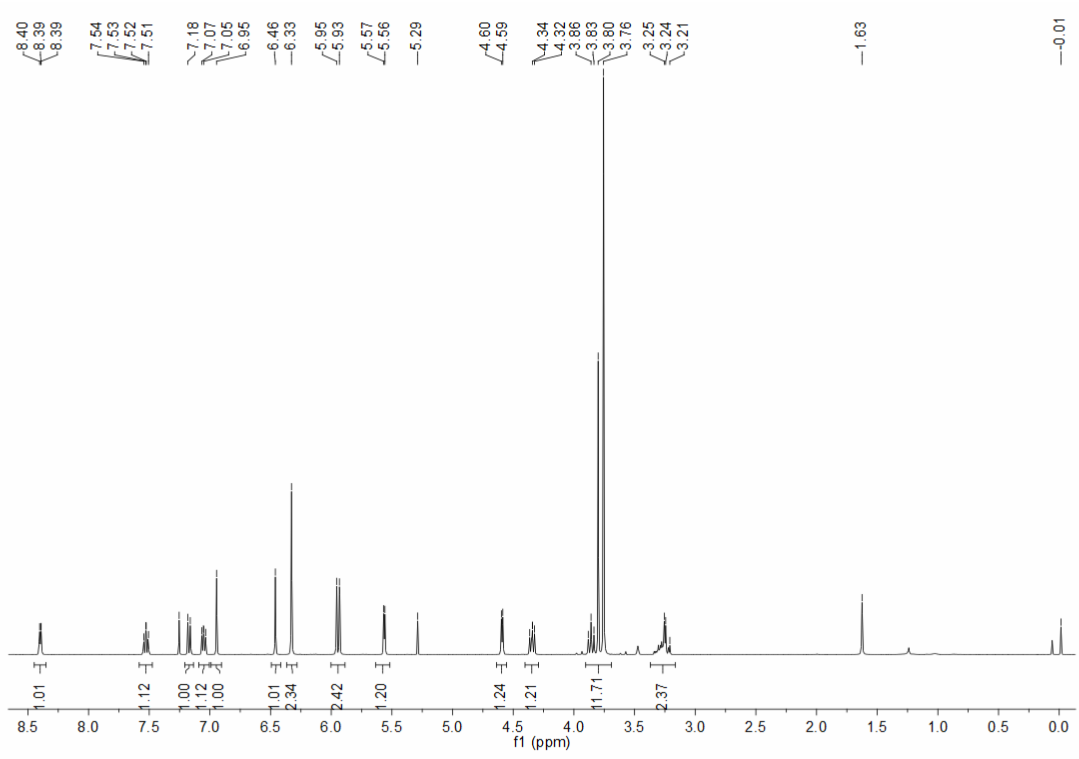

1.2. ^{13}C NMR spectrum of compound 3S.

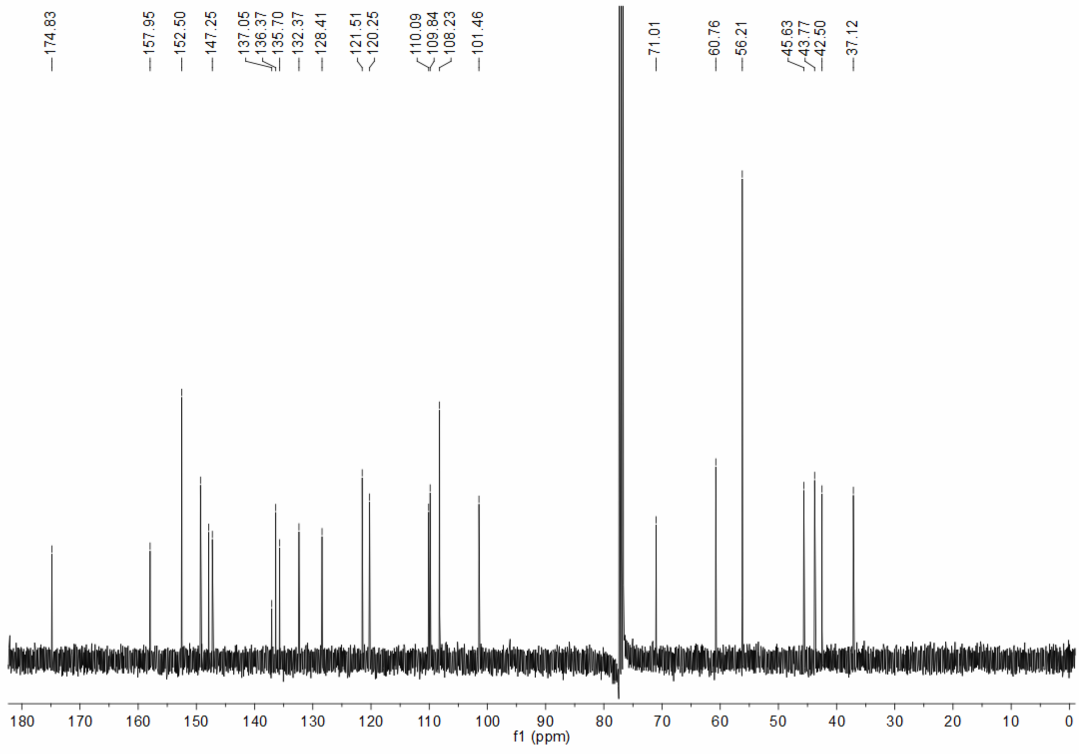

1.3. ^{1}H-^{1}H COSY diagram of compound 3S.

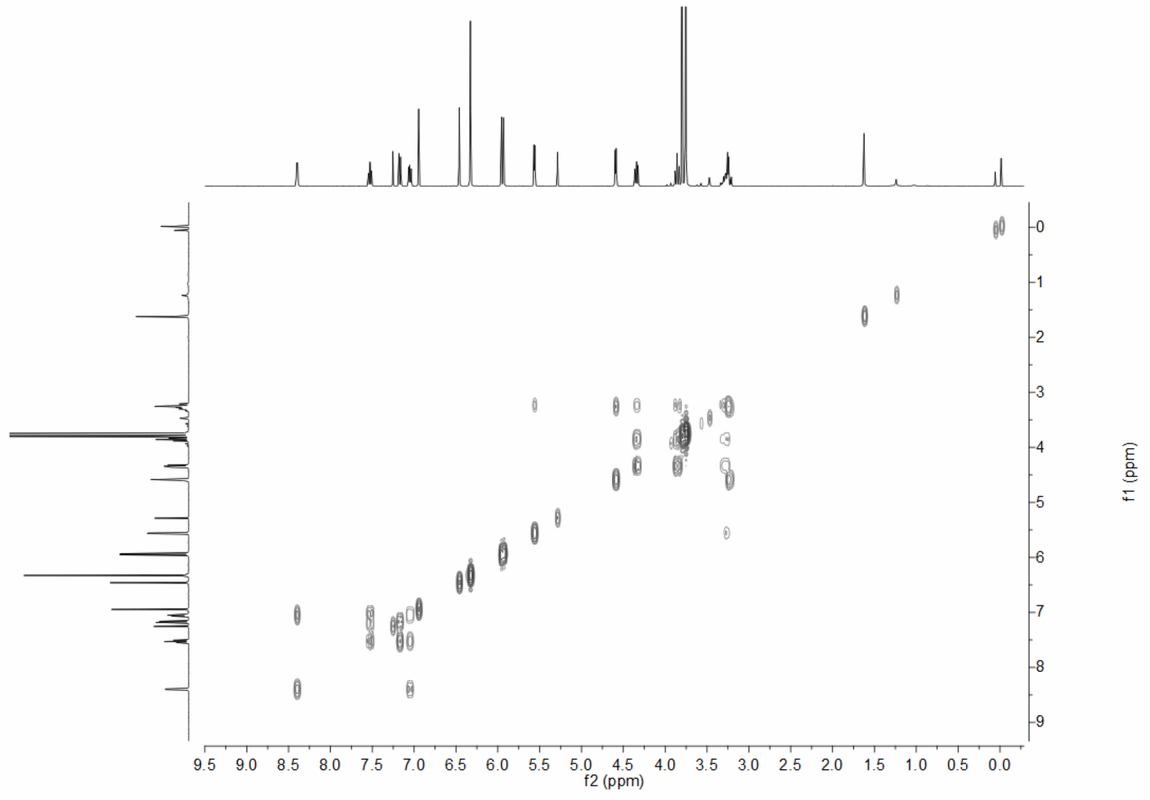

1.4. HMBC diagram of compound 3S.

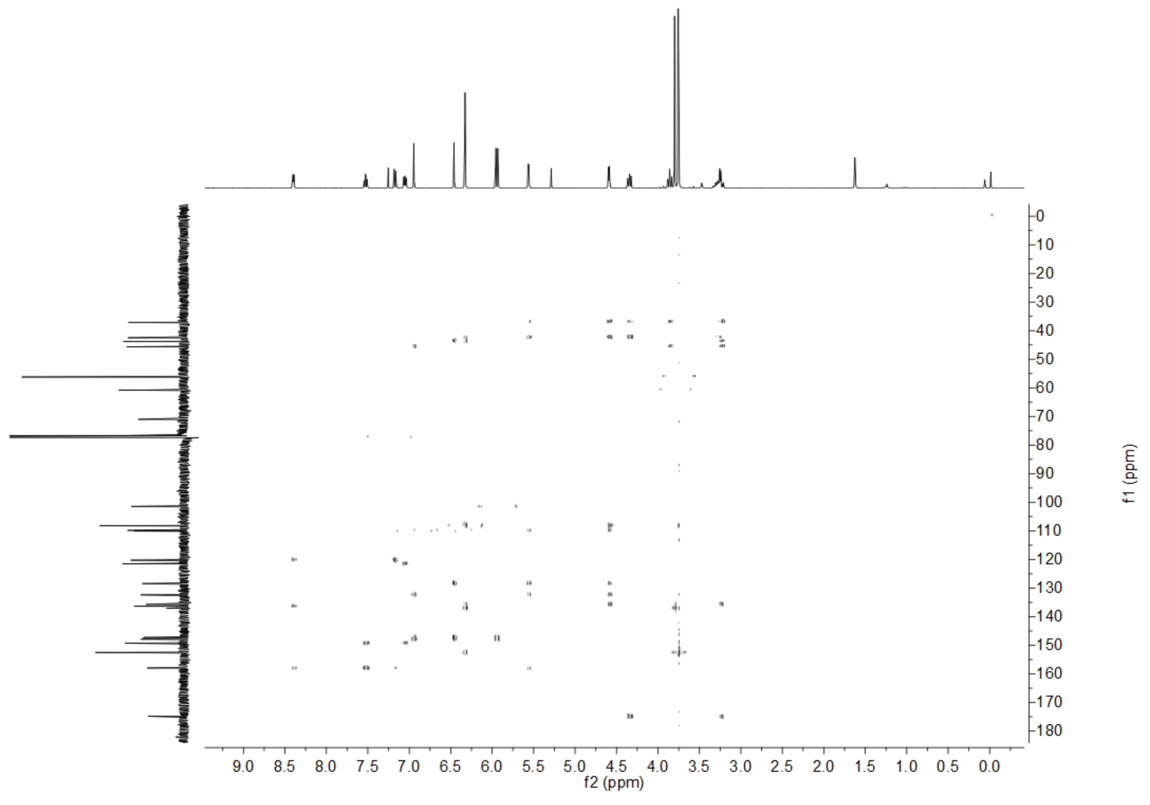

1.5. HSQC diagram of compound 3S.

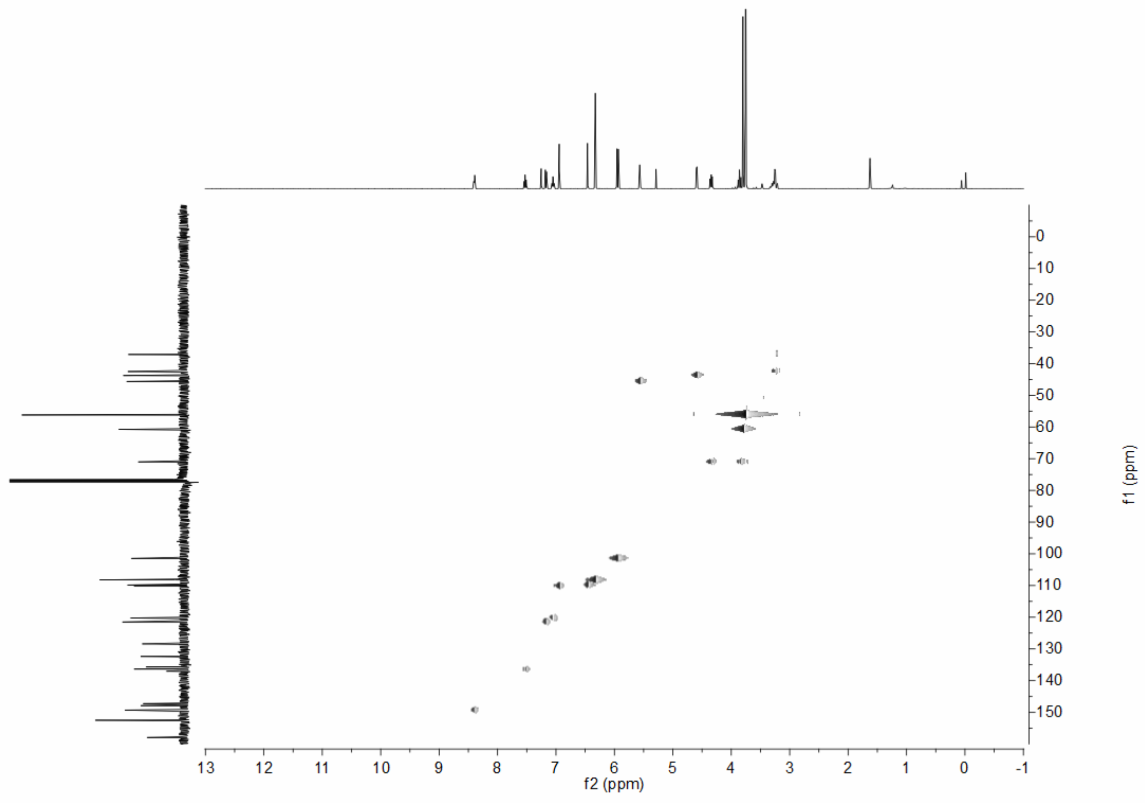

1.6. MS spectrum of compound 3S.

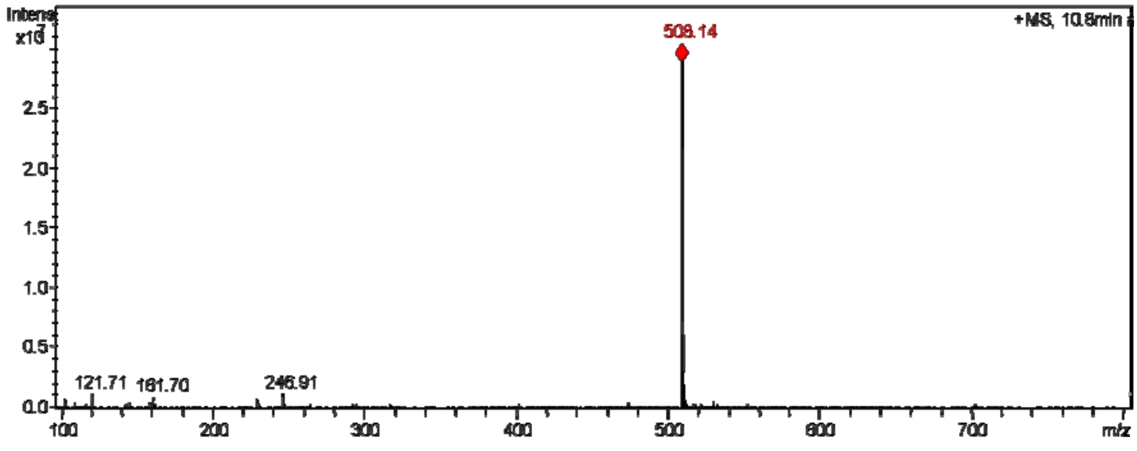

4β-S-(pyridine-2)-4-deoxy-podophyllotoxin (3S).

^{1}H NMR (400 MHz, CDCl$_3$, δ): 8.40 (d, *J* = 4.0 Hz, 1H), 7.52 (t, *J* = 4.0 Hz, 1H), 7.18 (d, *J* = 8.0 Hz, 1H), 7.05 (t, *J* = 4.0 Hz, 1H), 6.95 (s, 1H), 6.46 (s, 1H), 6.33 (s, 2H), 5.95 (d, *J* = 8.0 Hz, 2H), 5.57 (d, *J* = 4.0 Hz, 1H), 4.60 (d, *J* = 4.0 Hz, 1H), 4.34 (t, *J* = 8.0 Hz, 1H), 3.86 (t, *J* = 8.0 Hz, 1H), 3.80 (s, 3H), 3.76 (s, 6H), 3.25-3.21 (m, 2H); ^{13}C NMR (101 MHz, CDCl$_3$, δ): 174.83, 157.95, 152.50 (2C), 147.99, 147.89, 147.25, 137.05, 136.37, 135.70, 132.37, 128.41, 121.51, 120.25, 110.09, 109.84, 108.23 (2C), 101.46, 71.01, 60.76, 56.21 (2C), 45.63, 43.77, 42.50, 37.12.

ESI-MS: calc'd for $C_{27}H_{25}NO_7S$ [M+H]$^+$: 508.14, found 508.14 [M+H]$^+$.

2.1. ^{1}H NMR spectrum of compound 4S.

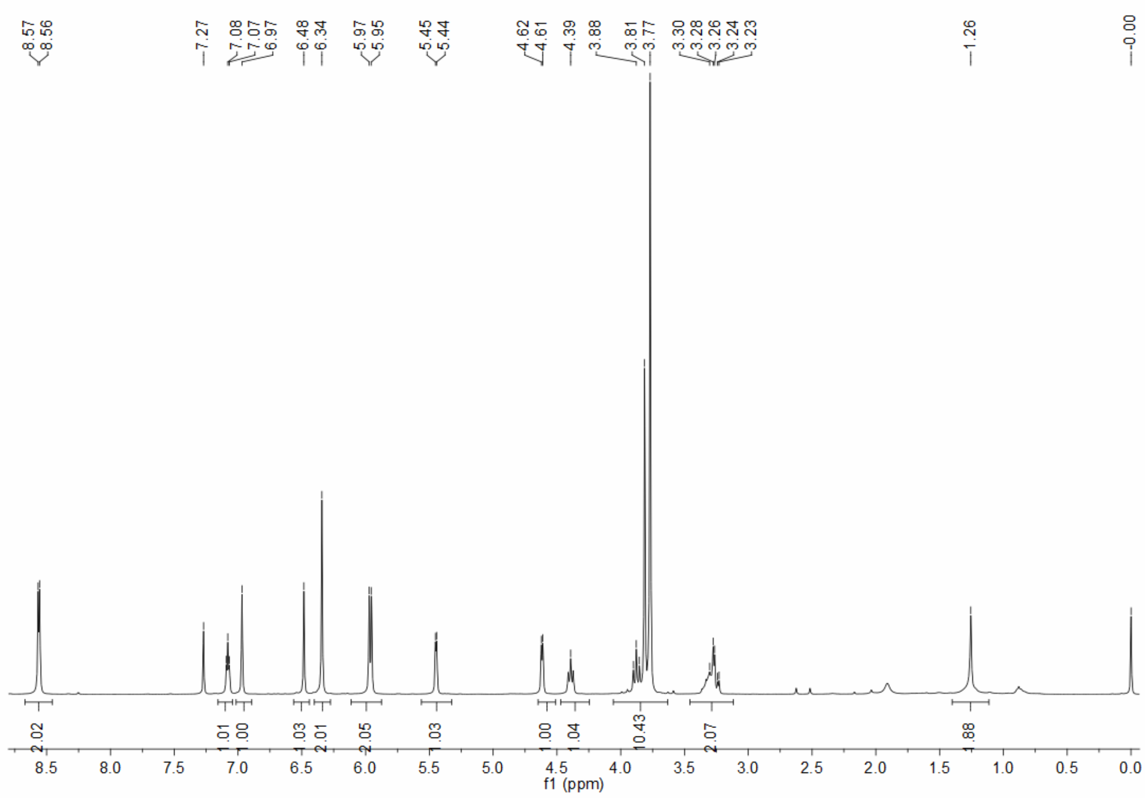

2.2. ^{13}C NMR spectrum of compound 4S.

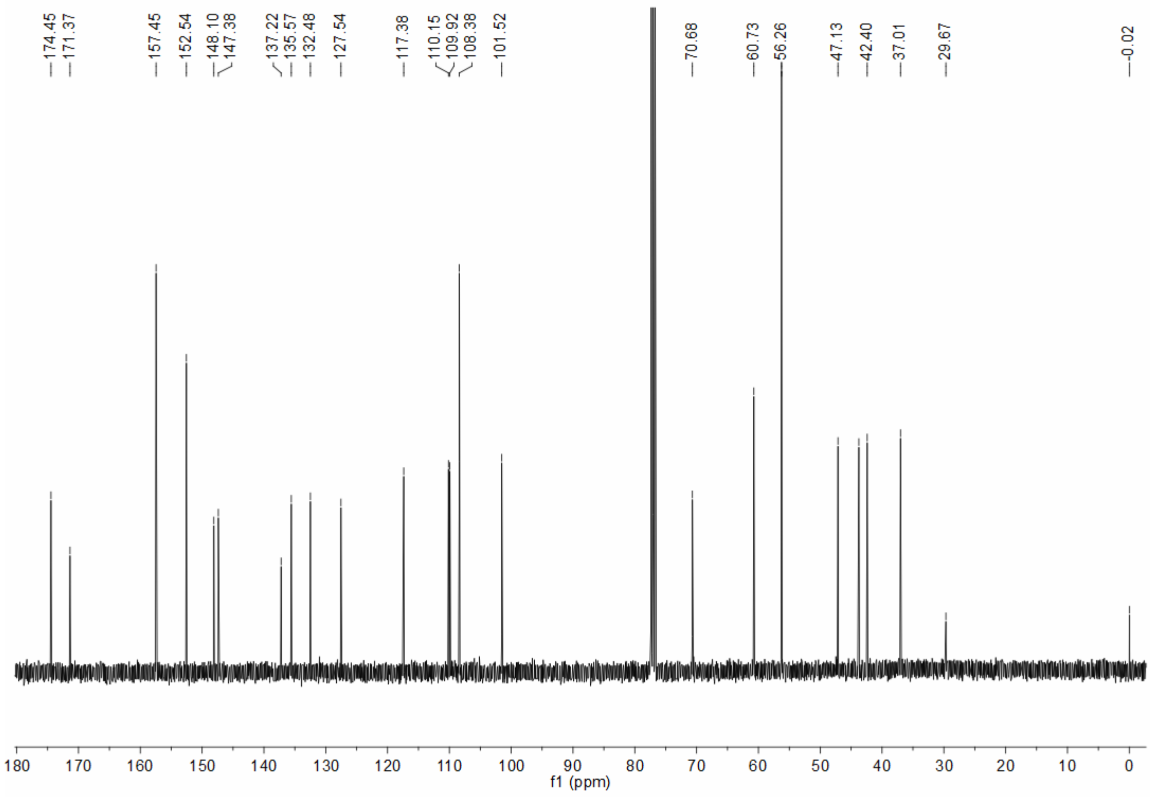

2.3. ¹H-¹H COSY diagram of compound 4S.

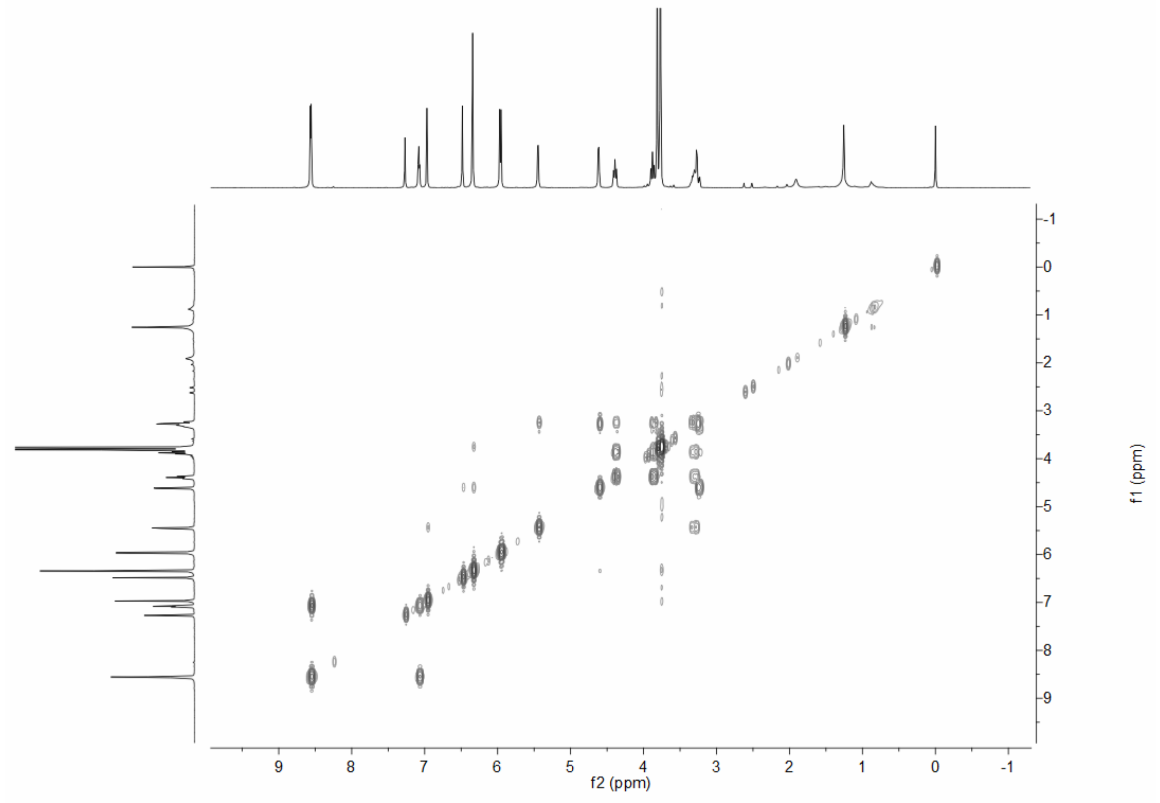

2.4. HMBC diagram of compound 4S.

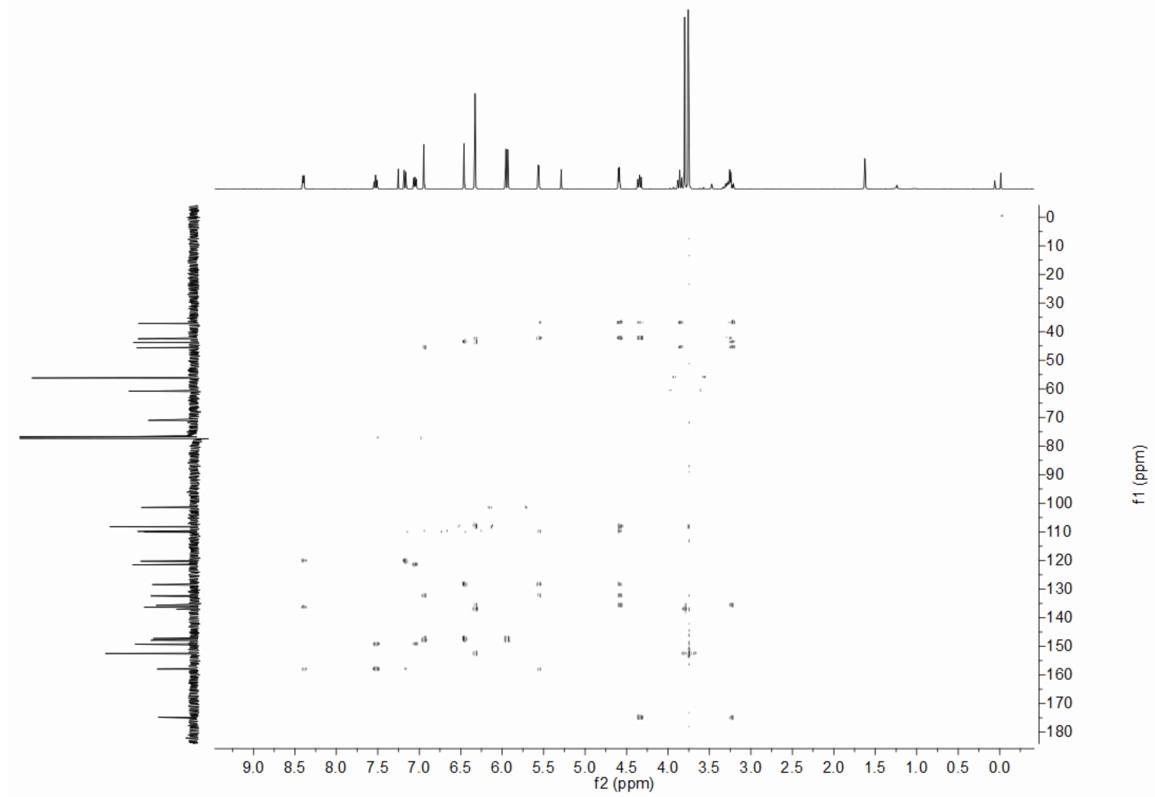

2.5. HSQC diagram of compound 4S.

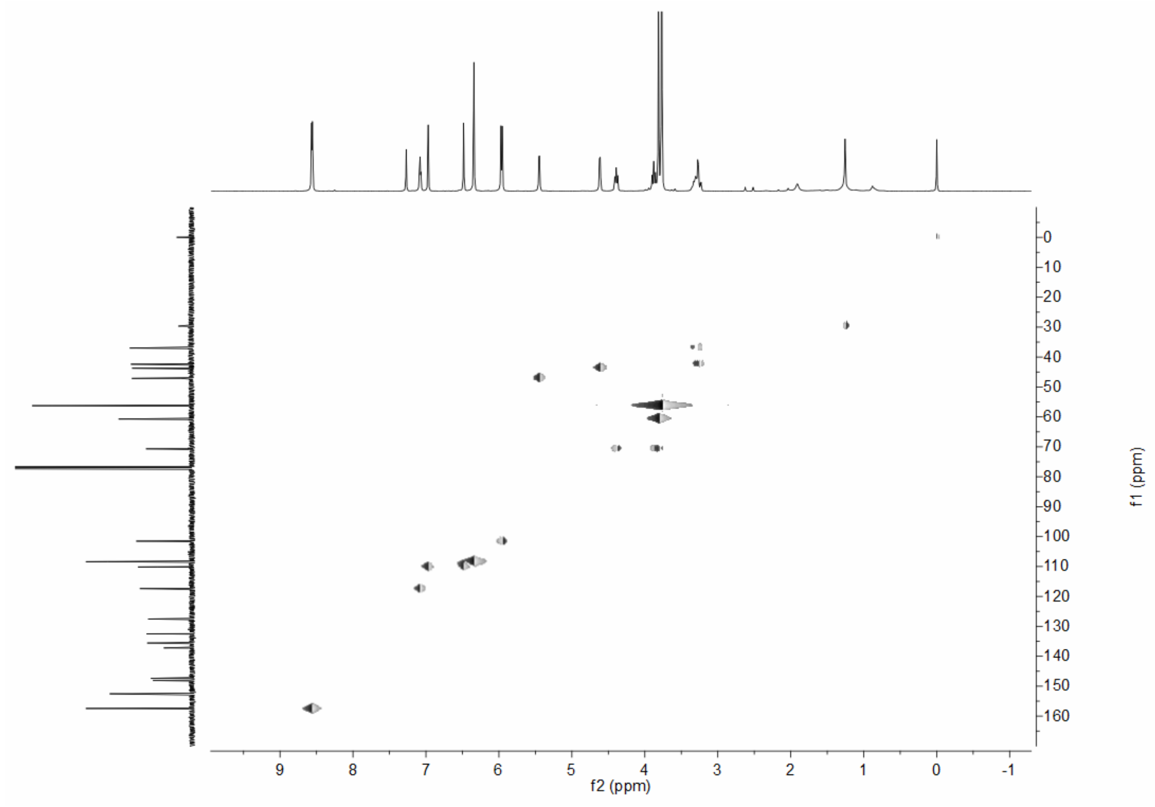

2.6. MS spectrum of compound 4S.

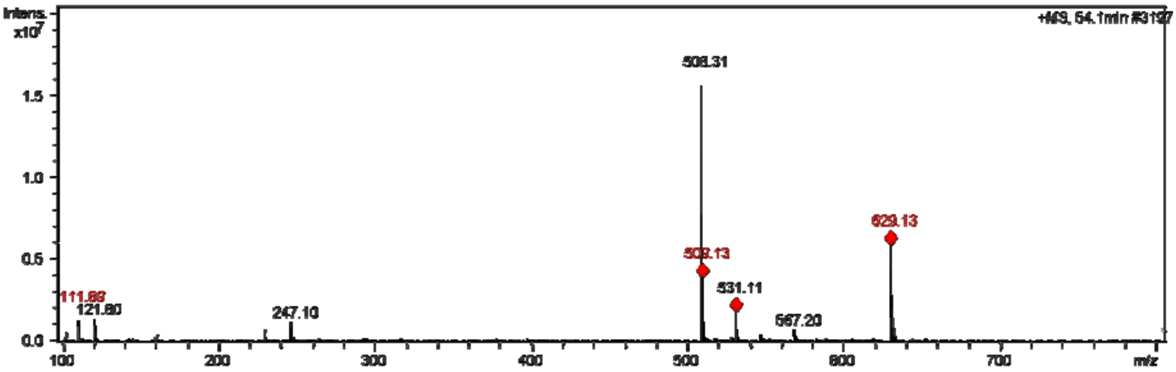

4β-S-(pyrimidine-2)-4-deoxy-podophyllotoxin (4S)

^{1}H NMR (400 MHz, $CDCl_3$, δ): 8.57 (d, *J* = 4.0 Hz, 2H), 7.08 (t, *J* = 4.0 Hz, 1H), 6.97 (s, 1H), 6.48 (s, 1H), 6.34 (s, 2H), 5.97 (d, *J* = 8.0 Hz, 2H), 5.45 (d, *J* = 4.0 Hz, 1H), 4.62 (d, *J* = 4.0 Hz, 1H), 4.39 (t, *J* = 8.0 Hz, 1H), 3.88 (t, *J* = 8.0 Hz, 1H), 3.81 (s, 3H), 3.77 (s, 6H), 3.30-3.23 (m, 2H); ^{13}C NMR (101 MHz, $CDCl_3$, δ): 174.45, 171.37, 157.45 (2C), 152.54 (2C), 148.10, 147.38, 137.22, 135.57, 132.48, 127.54, 117.38, 110.15, 109.92, 108.38 (2C), 101.52, 70.68, 60.73, 56.26 (2C), 47.13, 43.75, 42.40, 37.01.

ESI-MS: calc'd for $C_{26}H_{24}N_2O_7S$ $[M+H]^+$: 508.13, found 508.31 $[M+H]^+$; calc'd for $C_{26}H_{24}N_2O_7S$ $[M+H]^+$: 509.54, found 509.13 $[M+2H]^+$; calc'd for $C_{26}H_{24}N_2O_7S$ $[M+Na]^+$: 531.53, found 531.11 $[M+Na]^+$; calc'd for $C_{26}H_{24}N_2O_7S$ $[M+2Na+2K-2H]^+$: 629.16, found 629.13 $[M+2Na+2K-2H]^+$.

3.1. ^{1}H NMR spectrum of compound 5S.

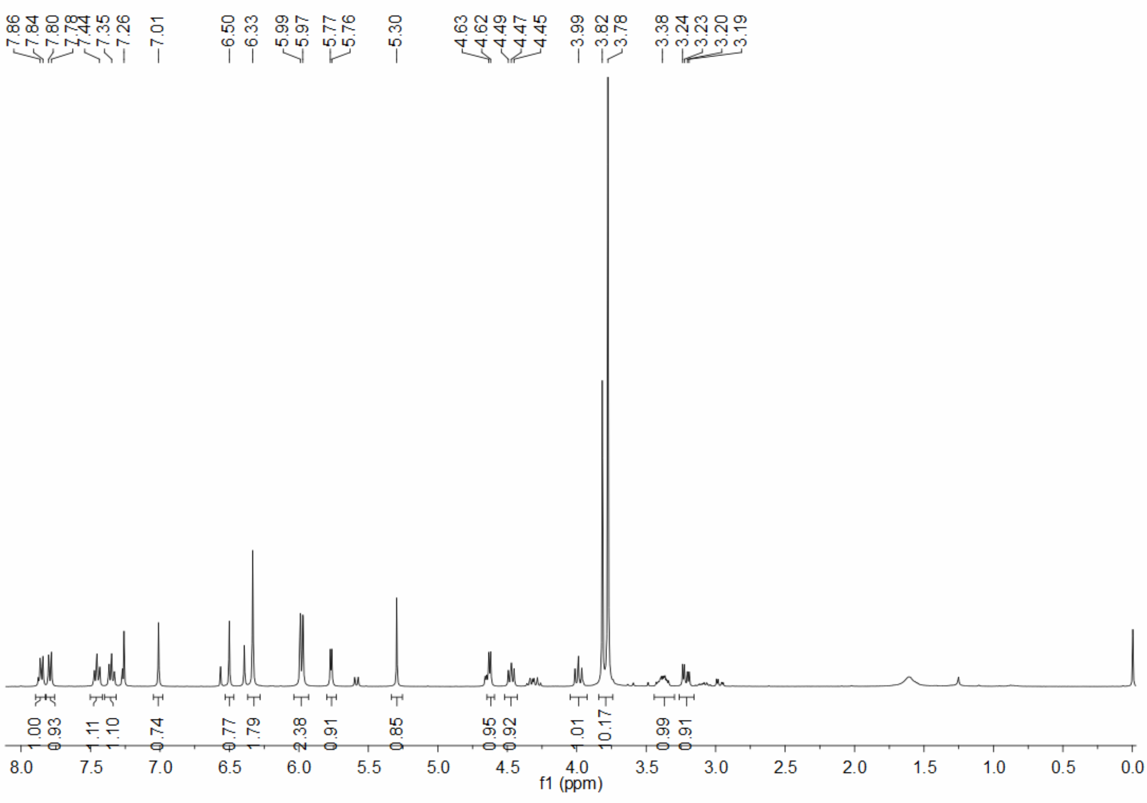

3.2. ^{13}C NMR spectrum of compound 5S.

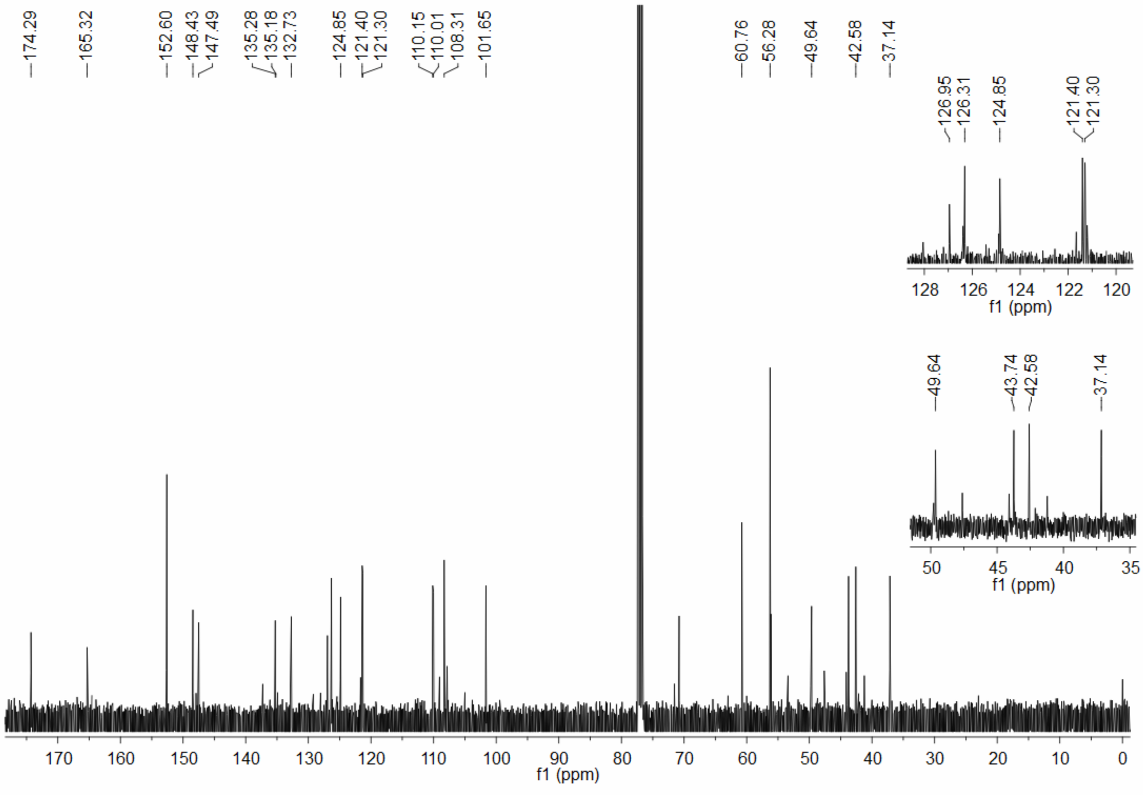

3.3. ^{1}H-^{1}H COSY spectrums for compound 5S.

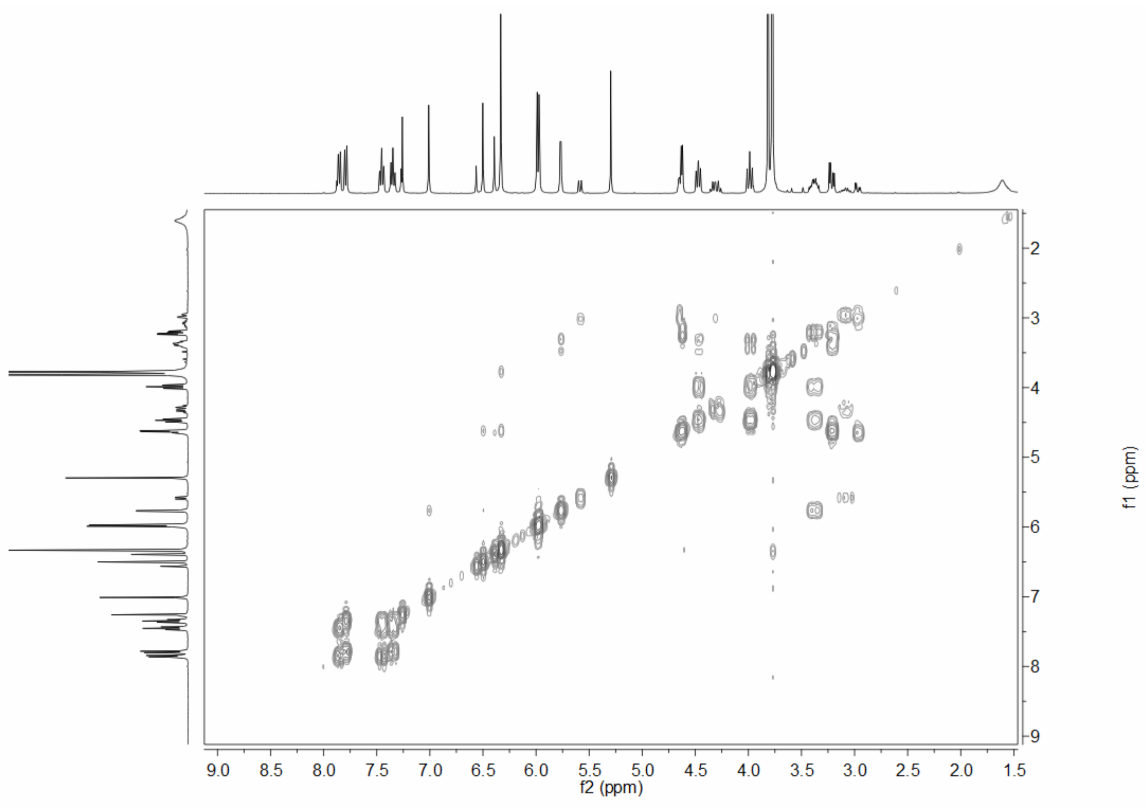

3.4. HMBC spectrums for compound 5S.

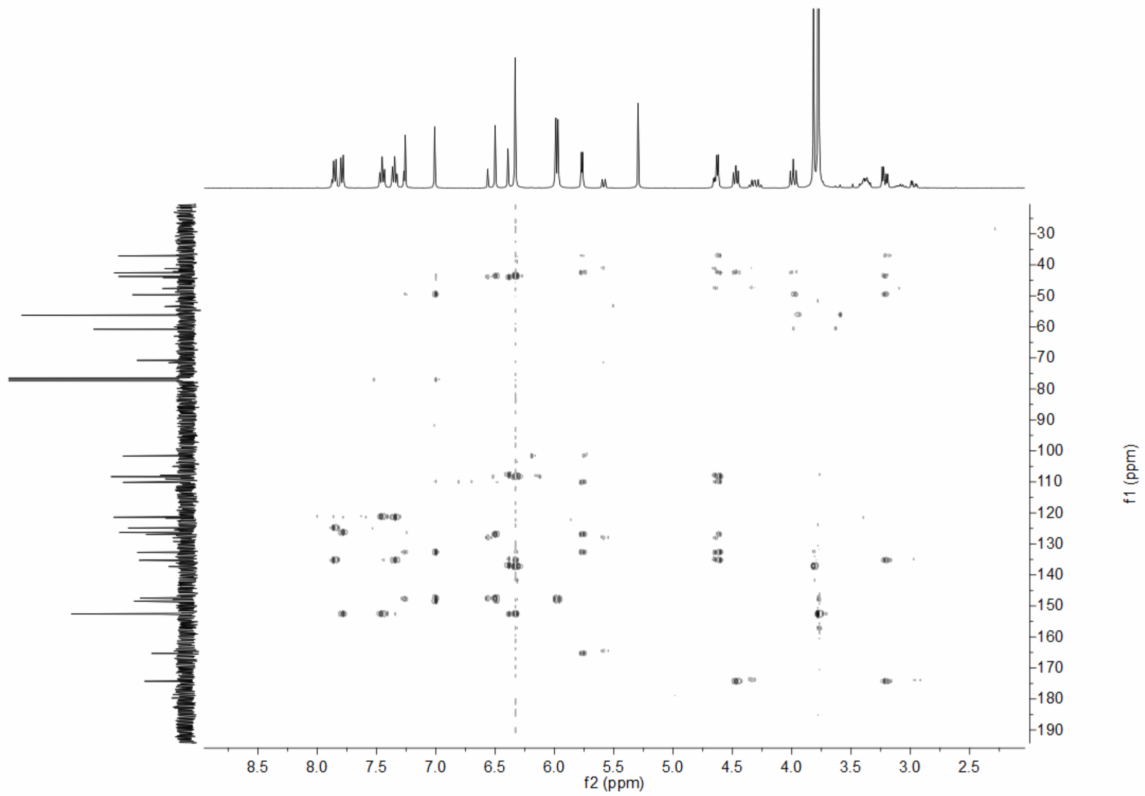

3.5. HSQC spectrums for compound 5S.

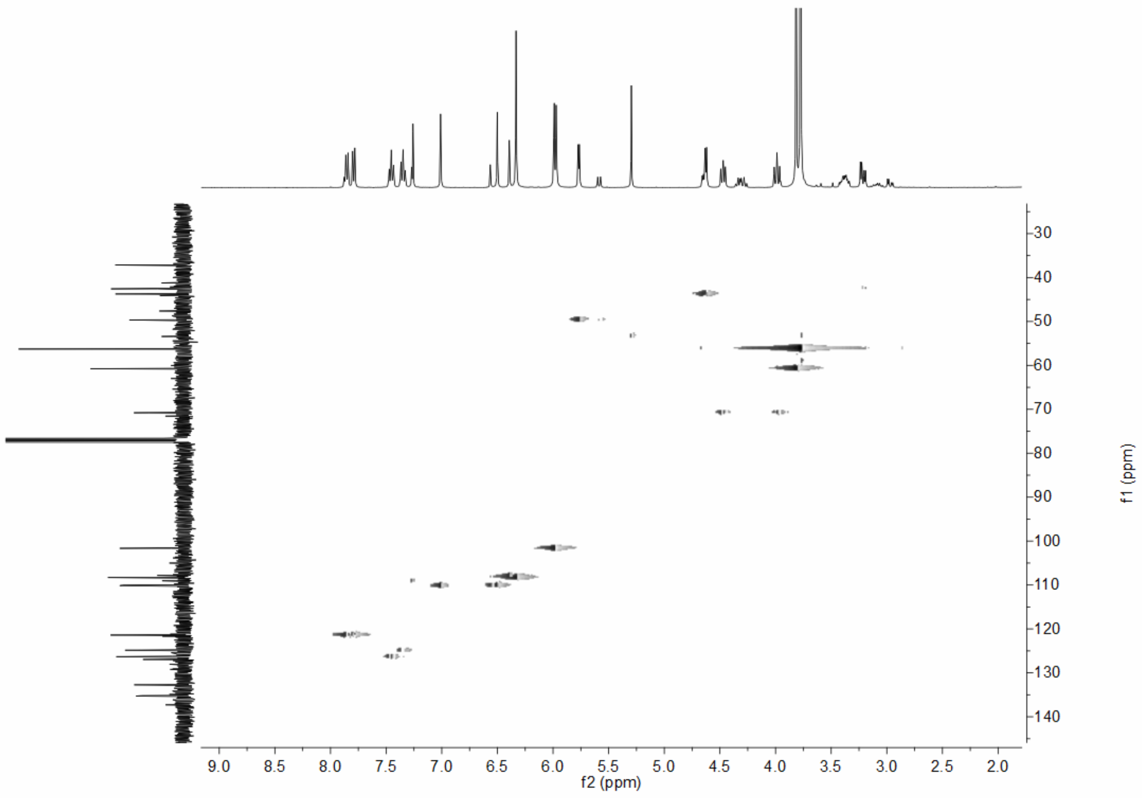

3.6. MS spectrum of compound 5S.

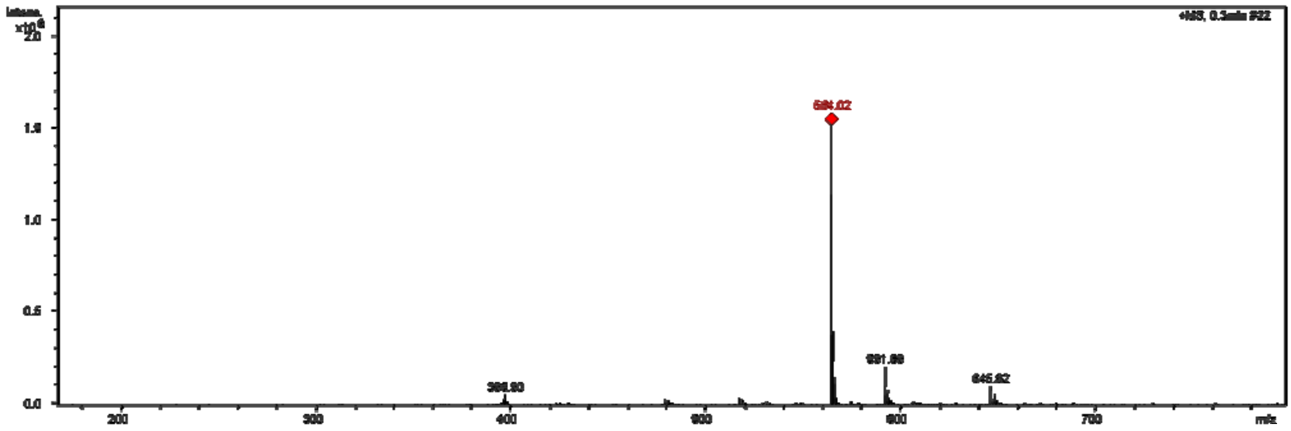

4β-S-(benzothiazole-2)-4-deoxy-podophyllotoxin (5S)

^{1}H NMR (400 MHz, $CDCl_3$, δ): 7.86 (d, *J* = 8.0 Hz, 1H), 7.80 (d, *J* = 8.0 Hz, 1H), 7.45 (t, *J* = 8.0 Hz, 1H), 7.35 (t, *J* = 8.0 Hz, 1H), 7.01 (s, 1H), 6.50 (s, 1H), 6.33 (s, 2H), 6.99 (d, *J* = 8.0 Hz, 2H), 5.77 (d, *J* = 4.0 Hz, 1H), 4.63 (d, *J* = 4.0 Hz, 1H), 4.47 (t, *J* = 8.0 Hz, 1H), 3.99 (t, *J* = 8.0 Hz, 1H), 3.82 (s, 3H), 3.78 (s, 6H), 3.43-3.34 (m, 1H), 3.24-3.19 (m, 1H); ^{13}C NMR (101 MHz, $CDCl_3$, δ): 174.29, 165.32, 152.60 (2C), 148.43 (2C), 147.49, 135.28, 135.18, 132.73, 126.95, 126.31, 124.85 (2C), 121.40, 121.30, 110.15, 110.01, 108.31 (2C), 101.65, 70.79, 60.76, 56.28 (2C), 49.64, 43.74, 42.58, 37.14.

ESI-MS: calc'd for $C_{29}H_{25}NO_7S_2$ $[M+H]^+$: 564.11, found 564.02 $[M+H]^+$.

4.1. ^{1}H NMR spectrum of compound 6S.

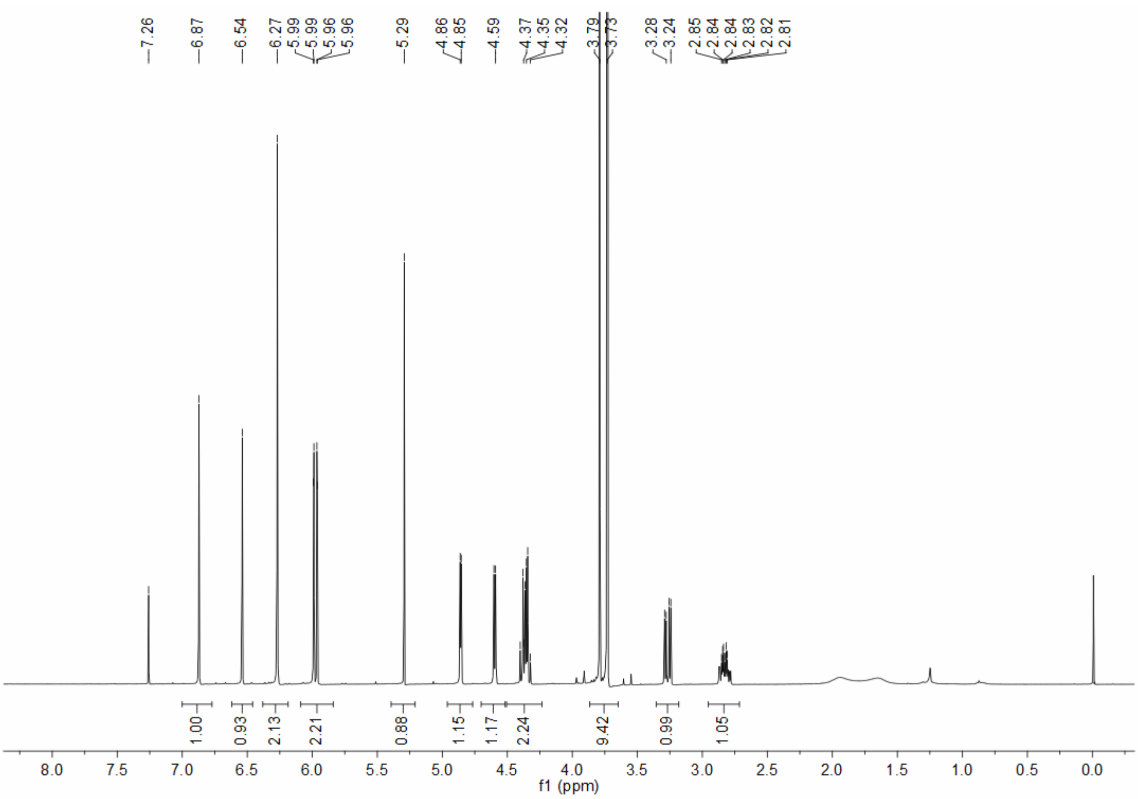

4.2. ^{13}C NMR spectrum of compound 6S.

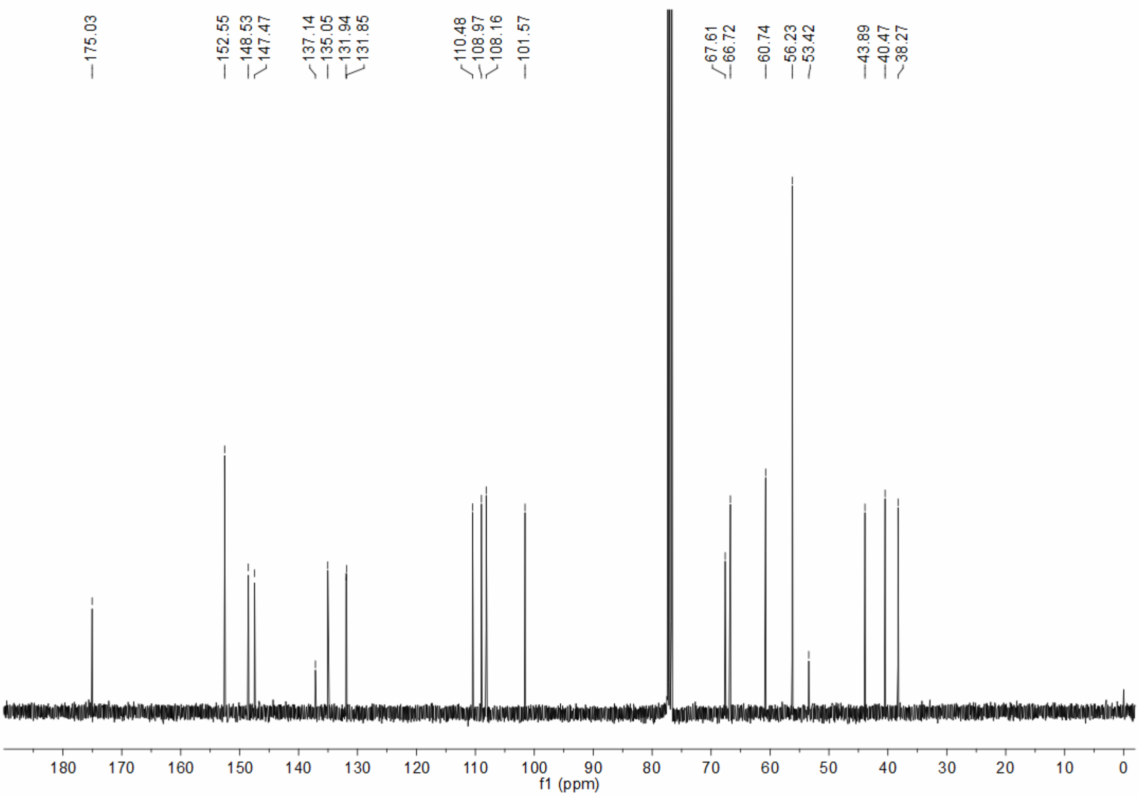

4.3. ^{1}H-^{1}H COSY spectrums for compound 6S.

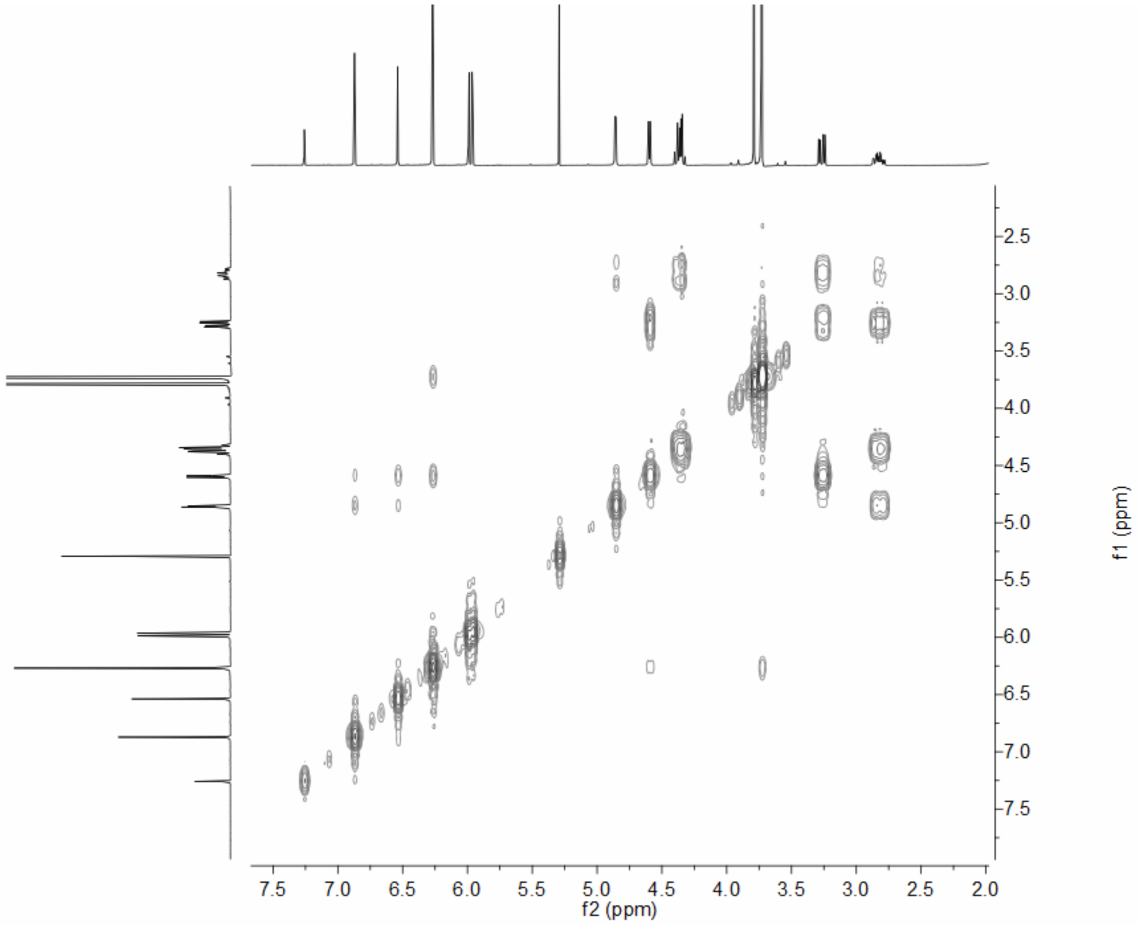

4.4. HMBC spectrums for compound 6S.

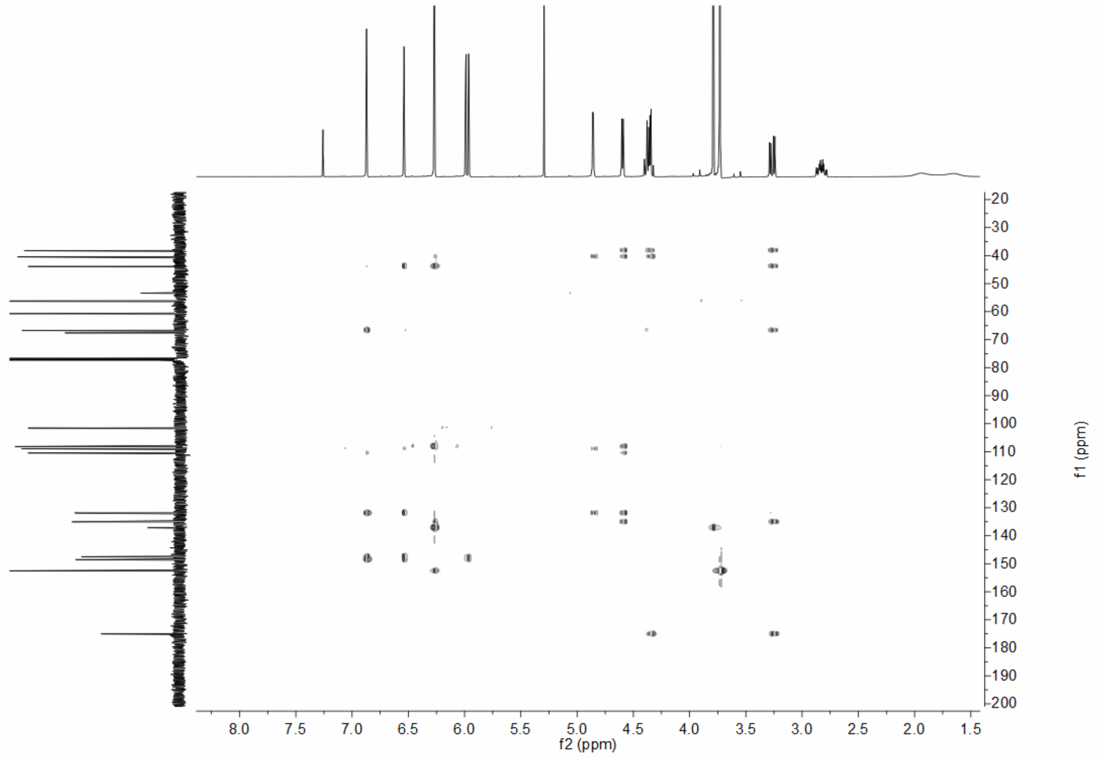

4.5. HSQC spectrums for compound 6S.

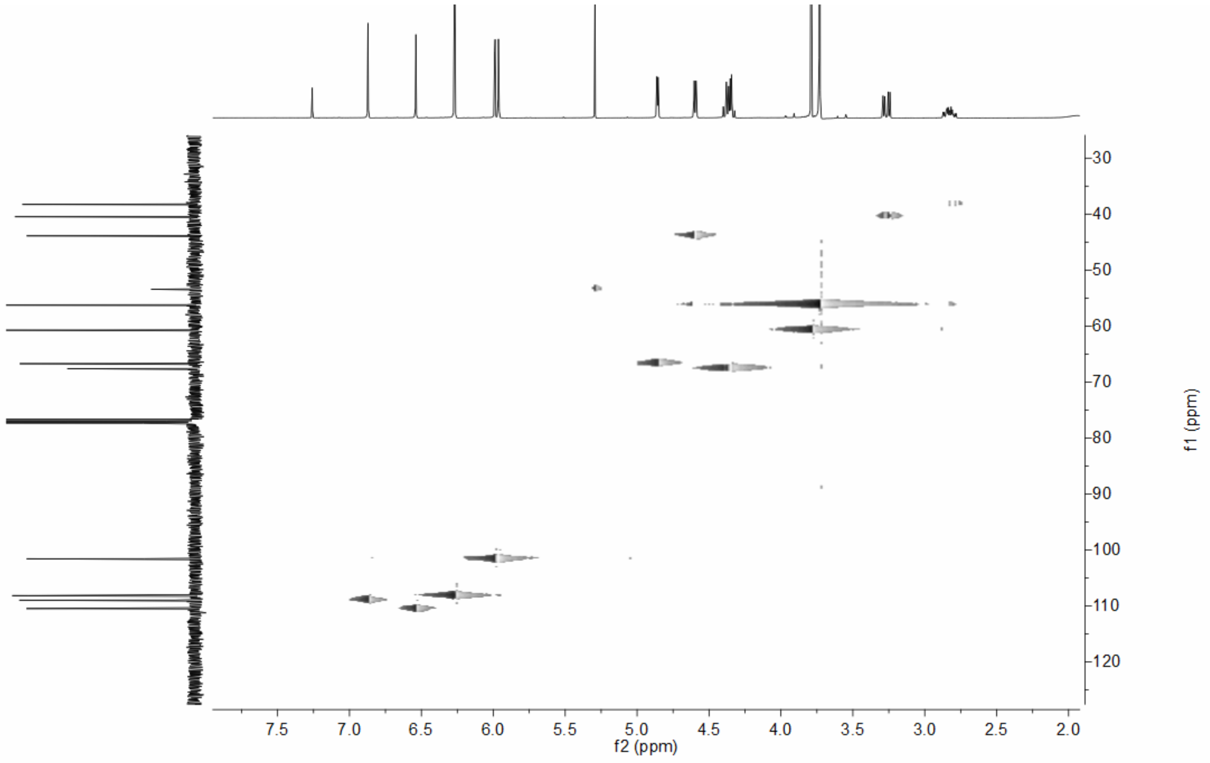

4.6. MS spectrum of compound 6S.

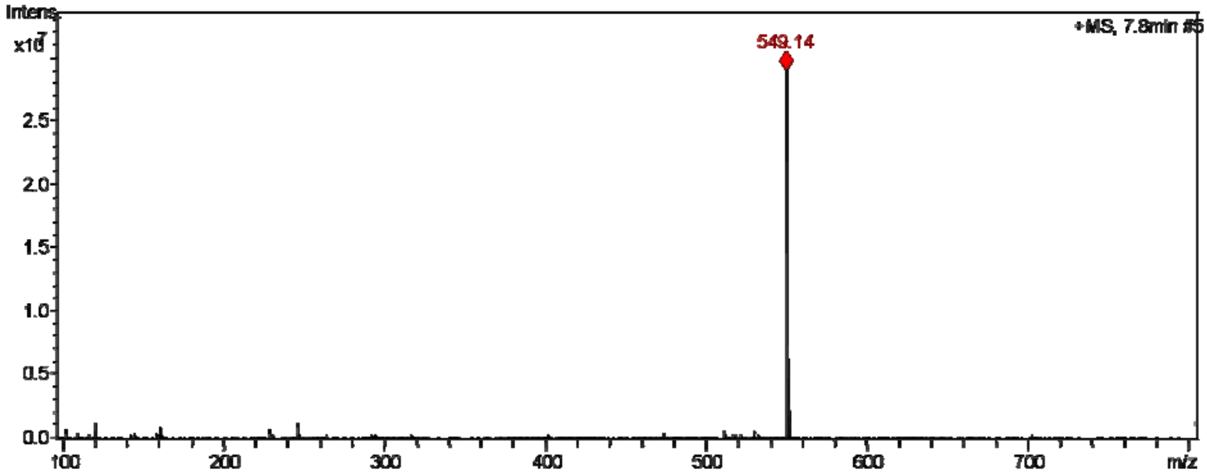

4β-S-(purine-6)-4-deoxy-podophyllotoxin (6S)

^{1}H NMR (300 MHz, $CDCl_3$, δ): 6.87 (s, 1H), 6.54 (s, 1H), 6.27 (s, 2H), 5.99 (d, *J* = 9.0 Hz, 2H), 5.29 (s, 1H), 4.86 (d, *J* = 9.0 Hz, 1H), 4.59 (d, *J* = 9.0 Hz, 1H), 4.37 - 4.32 (m, 1H), 3.79 (s, 3H), 3.73 (s, 6H), 3.28 (dd, J_1 = 16.0 Hz , J_2 = 6.0 Hz, 1H), 3.212-3.177 (m, 1H); ^{13}C NMR (75 MHz, $CDCl_3$, δ): 175.03 (2C), 152.55 (2C), 148.53, 147.47 (2C), 137.14, 135.05 (2C), 131.94, 131.85, 110.48 (2C), 108.97, 108.16 (2C), 67.61, 66.72, 60.74, 56.23 (2C), 53.42, 47.258, 43.89, 40.47, 38.27.

ESI-MS: calc'd for $C_{27}H_{24}N_4O_7S$ $[M+H]^+$: 549.14, found 549.14 $[M+H]^+$.

5.1. ^{1}H NMR spectrum of compound 3'S.

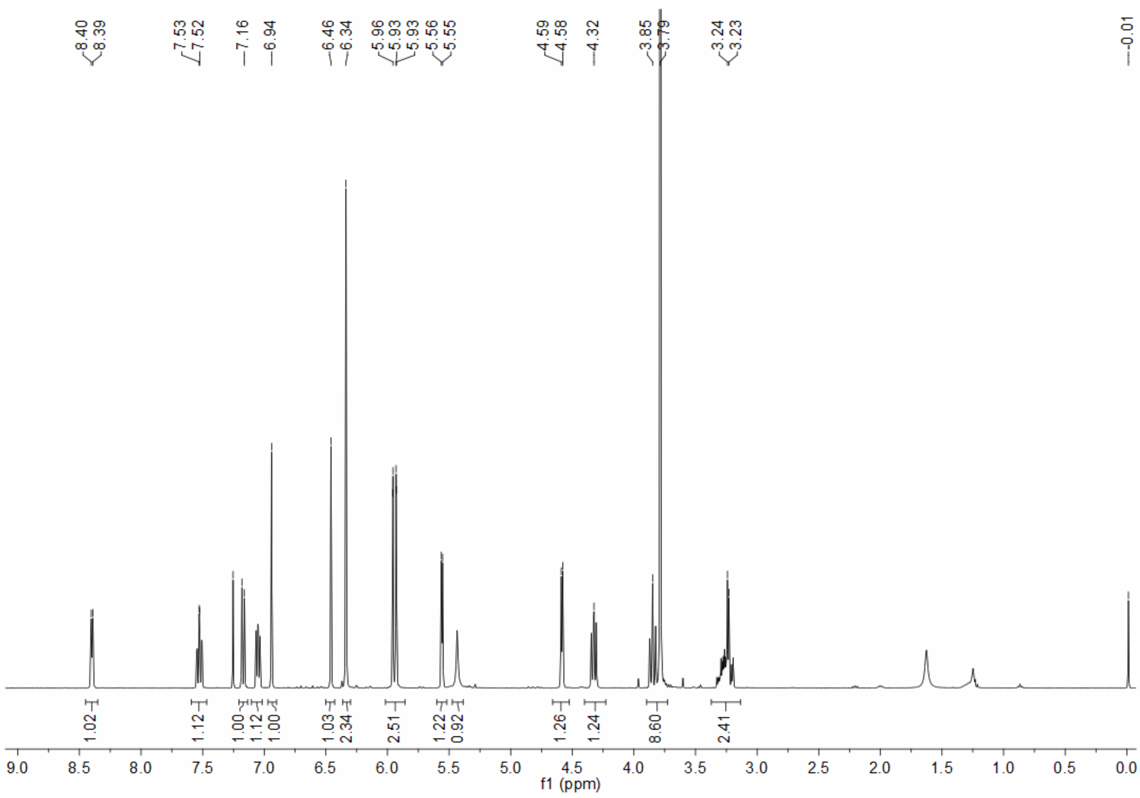

5.2. ^{13}C NMR spectrum of compound 3'S.

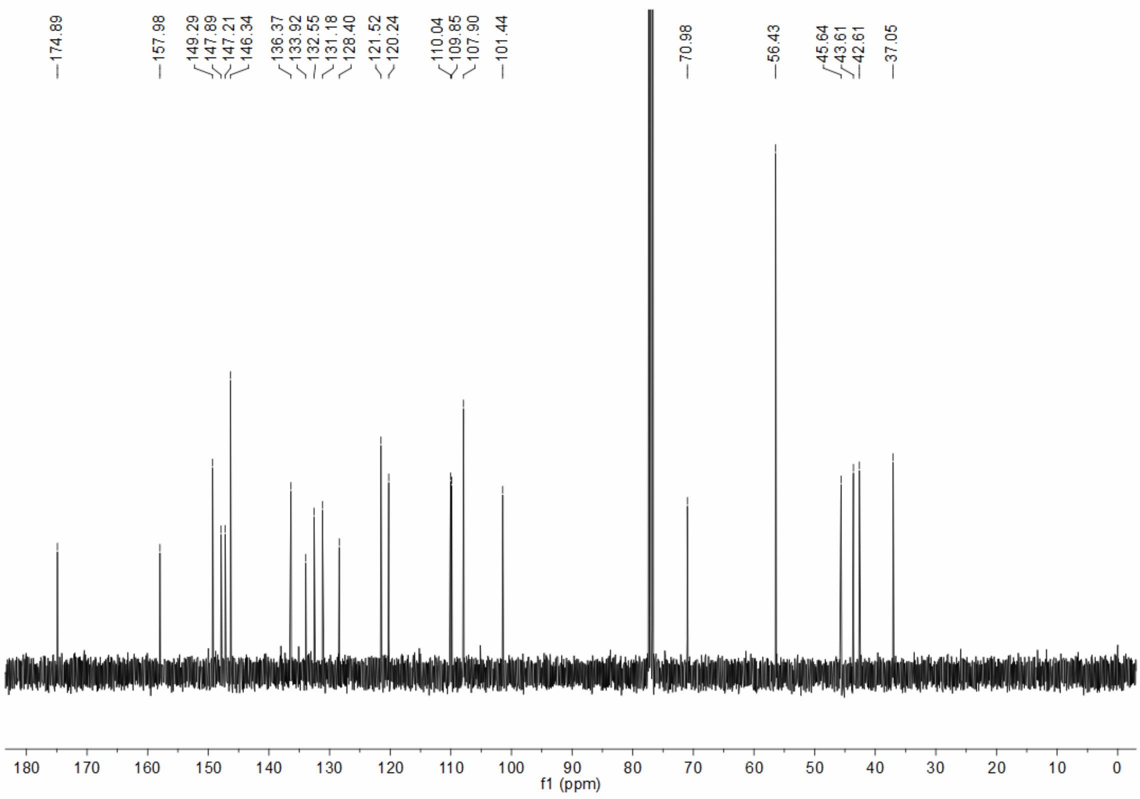

5.3. ^{1}H-^{1}H COSY spectrums for compound 3'S.

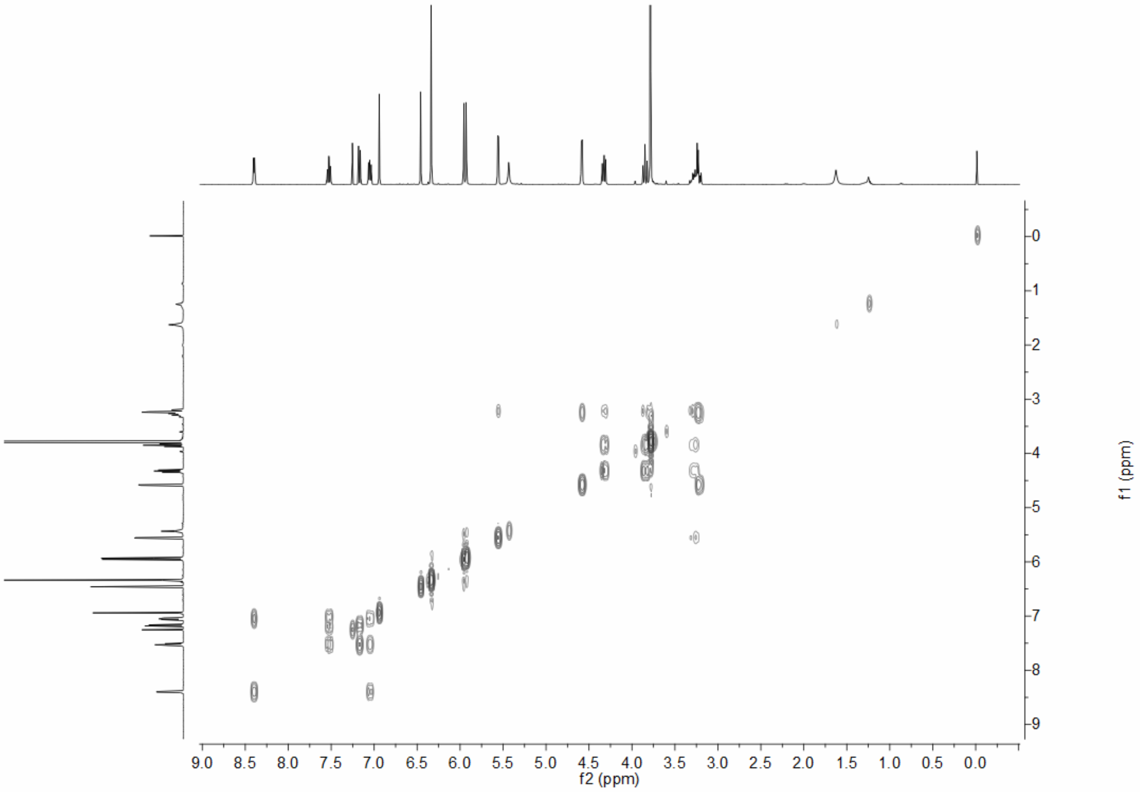

5.4. HMBC spectrums for compound 3'S.

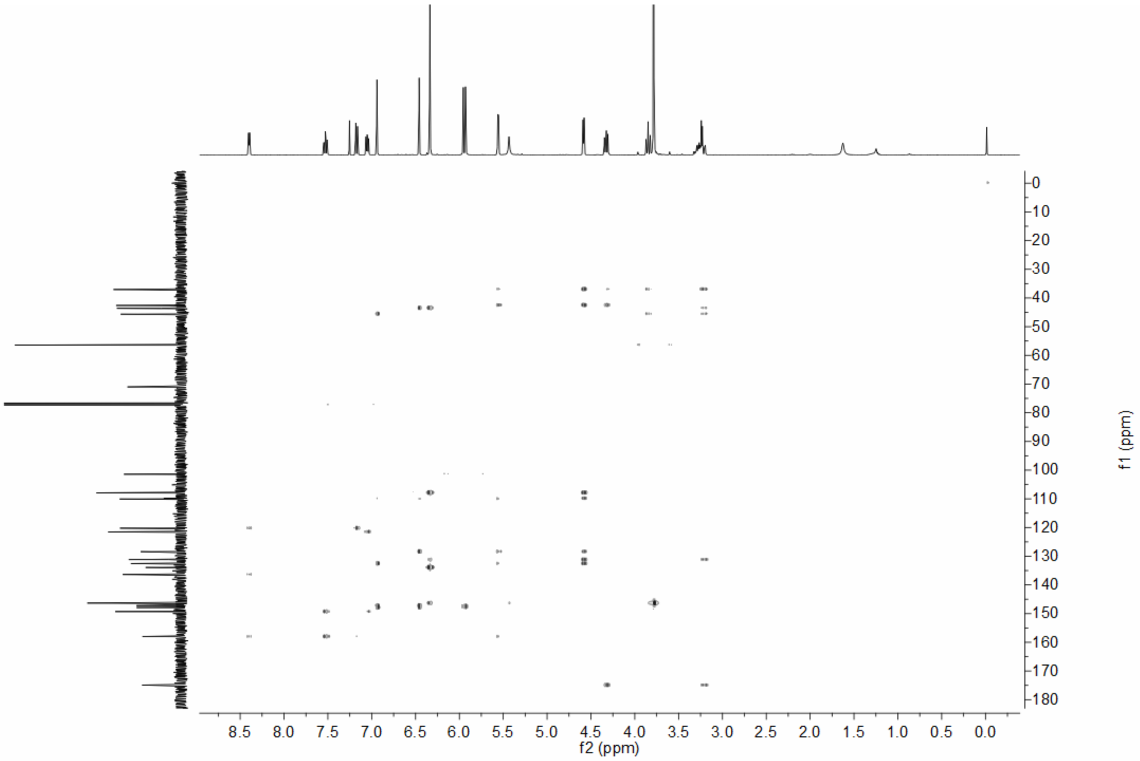

5.5. HSQC spectrums for compound 3'S.

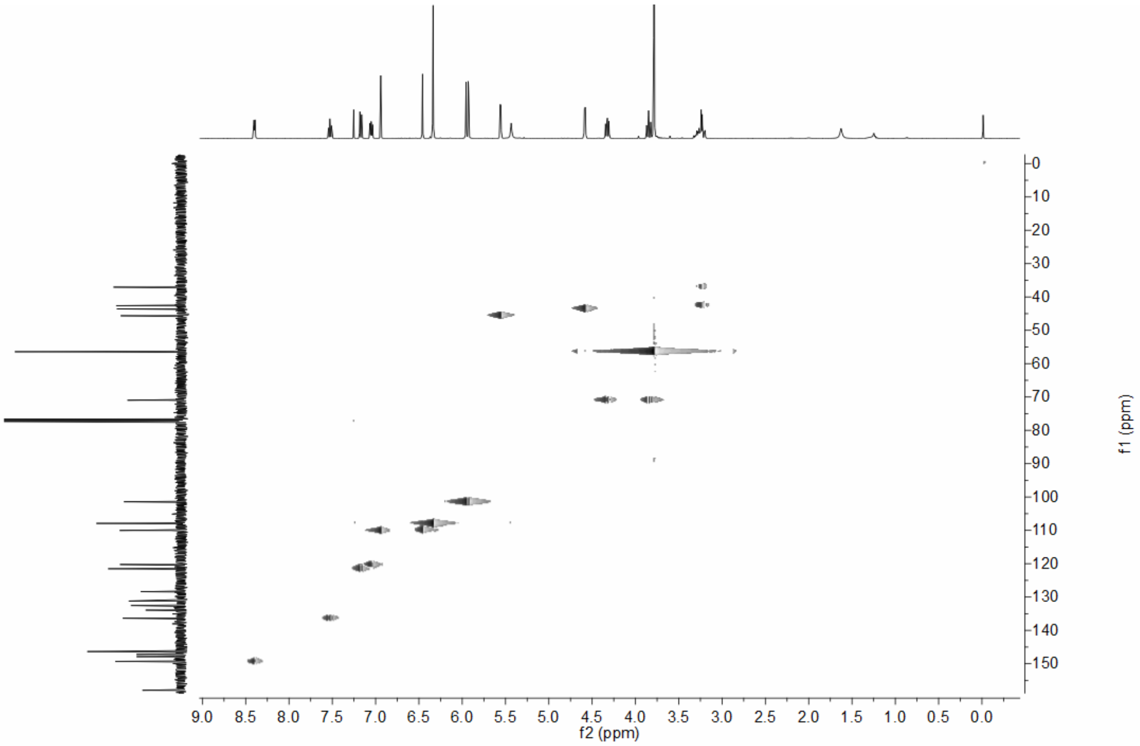

5.6. MS spectrum of compound 3'S.

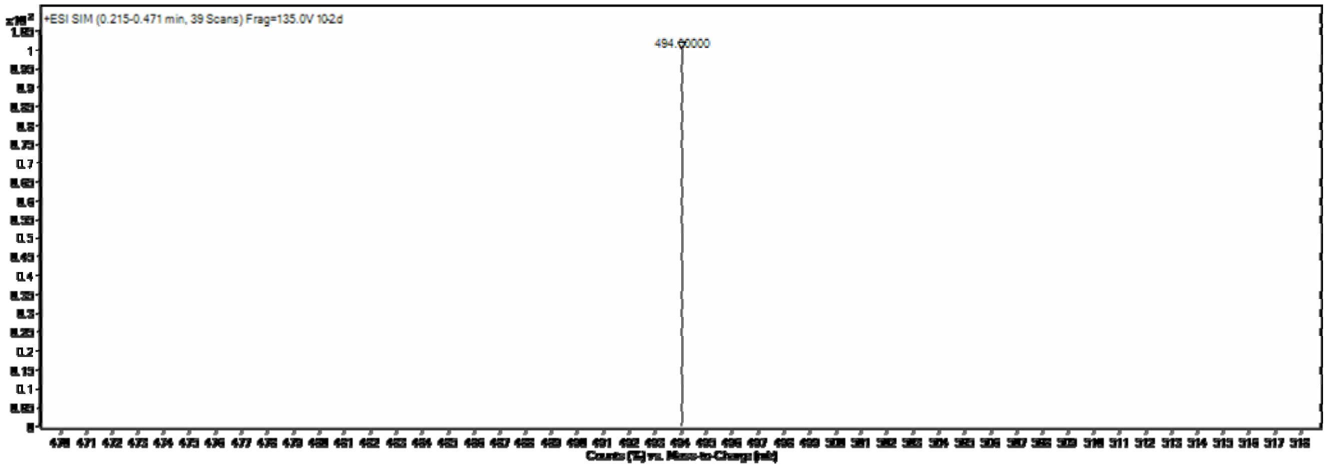

4β-S-(pyridine-2)-4-deoxy-4'-demethyl-podophyllotoxin (3'S)

^{1}H NMR (400 MHz, $CDCl_3$, δ): 8.40 (d, *J* = 4.0 Hz, 1H), 7.52 (t, *J* = 8.0 Hz, 1H), 7.18 (d, *J* = 8.0 Hz, 1H), 7.05 (t, *J* = 8.0 Hz, 1H), 6.94 (s, 1H), 6.46 (s, 1H), 6.34 (s, 2H), 5.96 (d, *J* = 12.0 Hz, 2H), 5.56 (d, *J* = 4.0 Hz, 1H), 5.35 (s, 1H), 4.59 (d, *J* = 4.0 Hz, 1H), 4.33 (t, *J* = 8.0 Hz, 1H), 3.85 (t, *J* = 8.0 Hz, 1H), 3.79 (s, 6H), 3.33-3.20 (m, 2H); ^{13}C NMR (101 MHz, $CDCl_3$, δ): 174.89, 157.98, 149.29, 147.89, 147.21, 146.34 (2C), 136.37, 133.92, 132.55, 131.18, 128.40, 121.52, 120.24, 110.04, 109.85, 107.90 (2C), 101.44, 70.98, 56.43 (2C), 45.64, 43.61, 42.61, 37.05.

ESI-MS: calc'd for $C_{26}H_{23}NO_7S$ $[M+H]^+$: 494.12, found 494.00 $[M+H]^+$.

6.1. ^{1}H NMR spectrum of compound 4'S

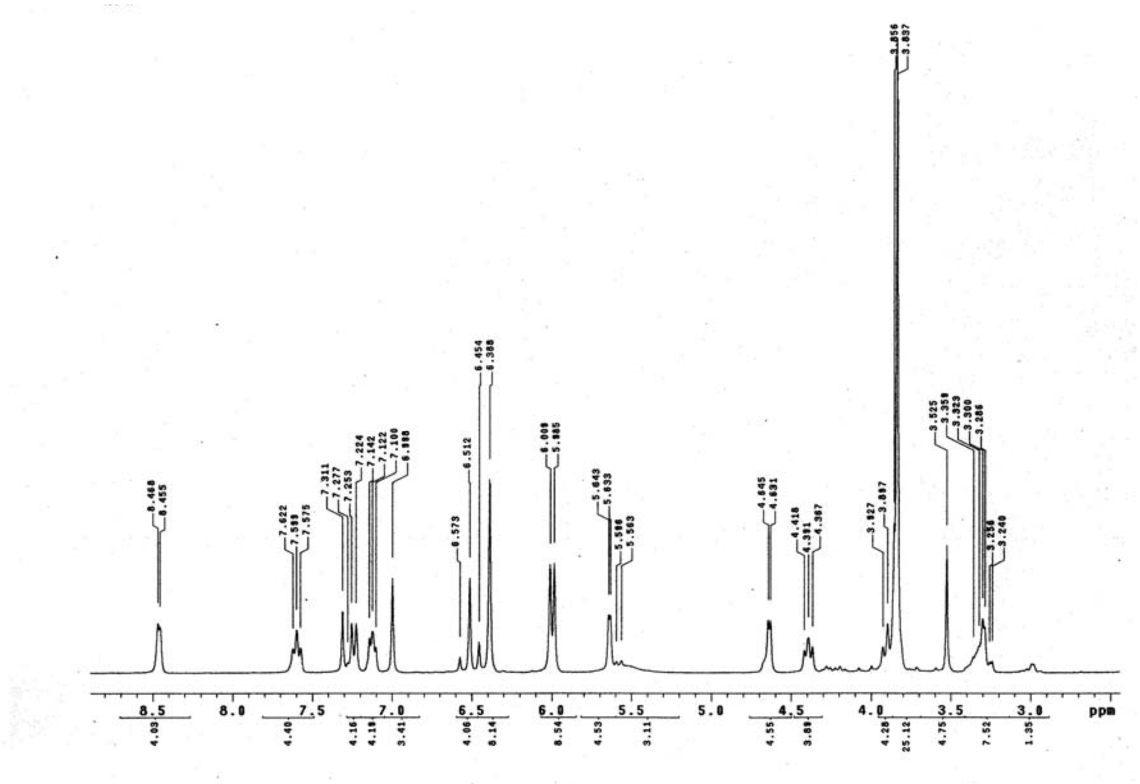

.

6.2. ^{13}C NMR spectrum of compound 4'S.

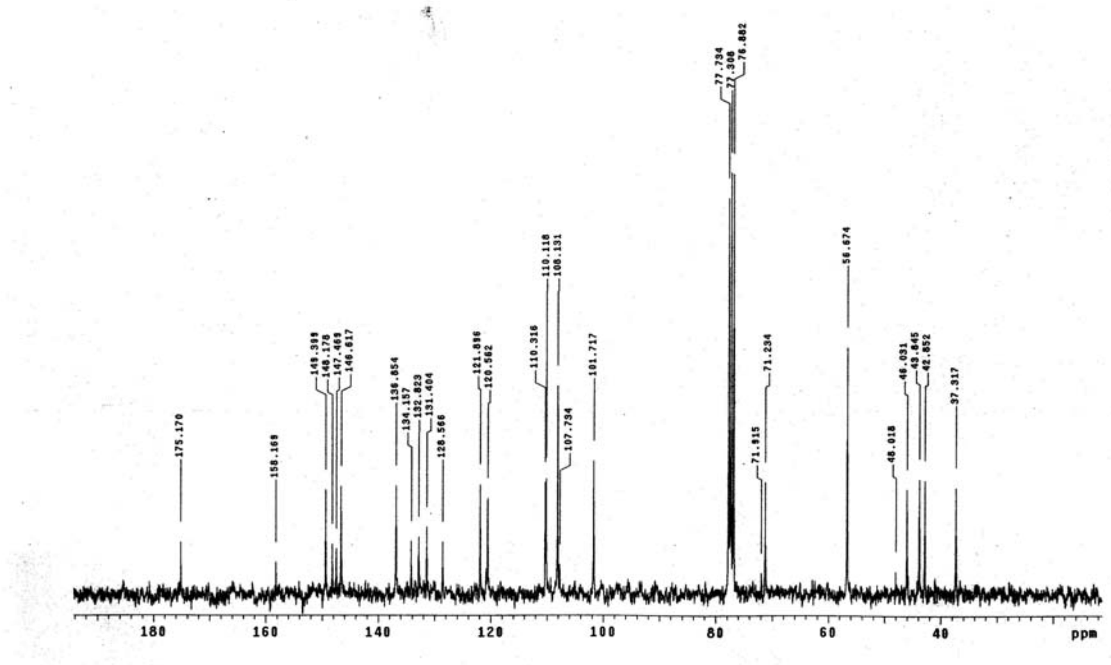

6.3. ^{1}H-^{1}H COSY spectrums for compound 4'S.

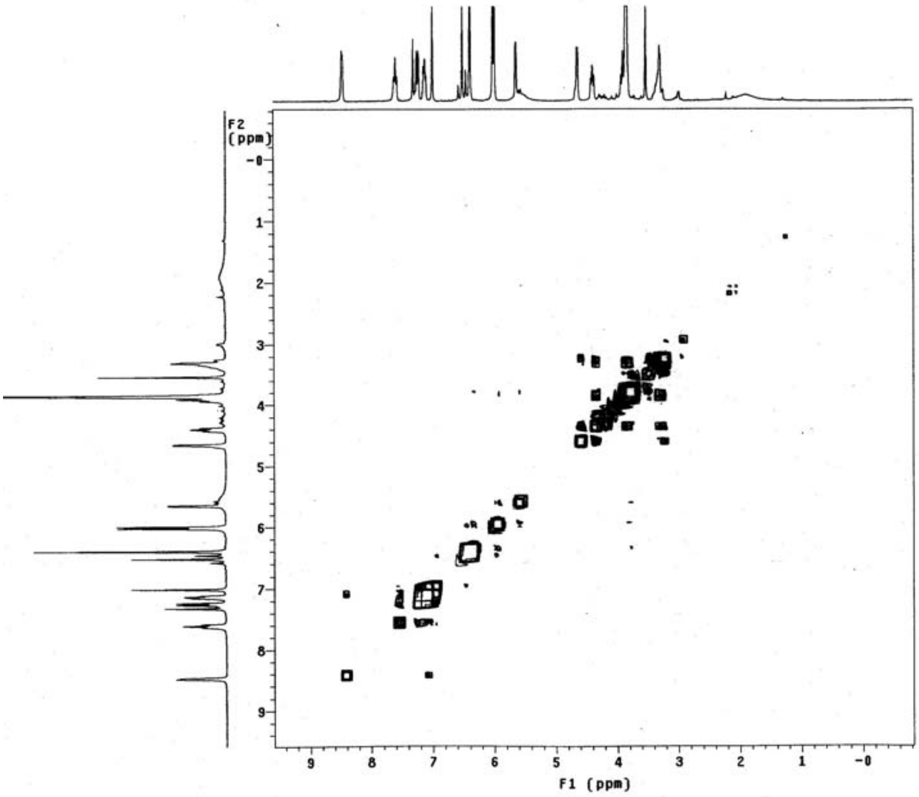

6.4. HMBC spectrums for compound 4'S.

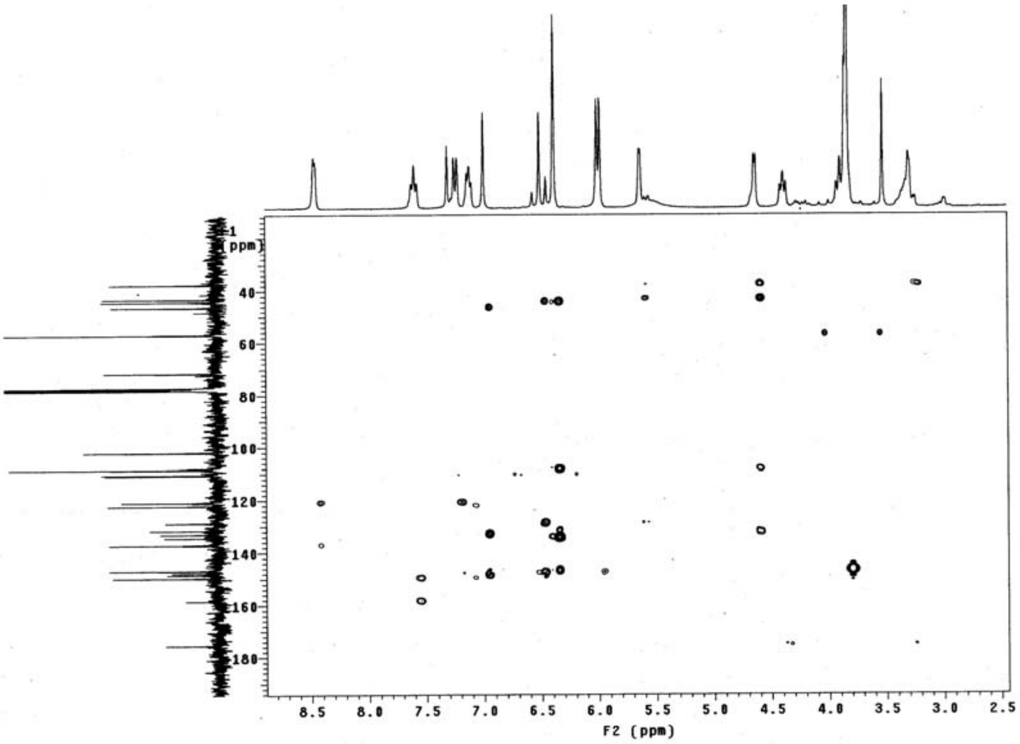

6.5. HSQC spectrums for compound 4'S.

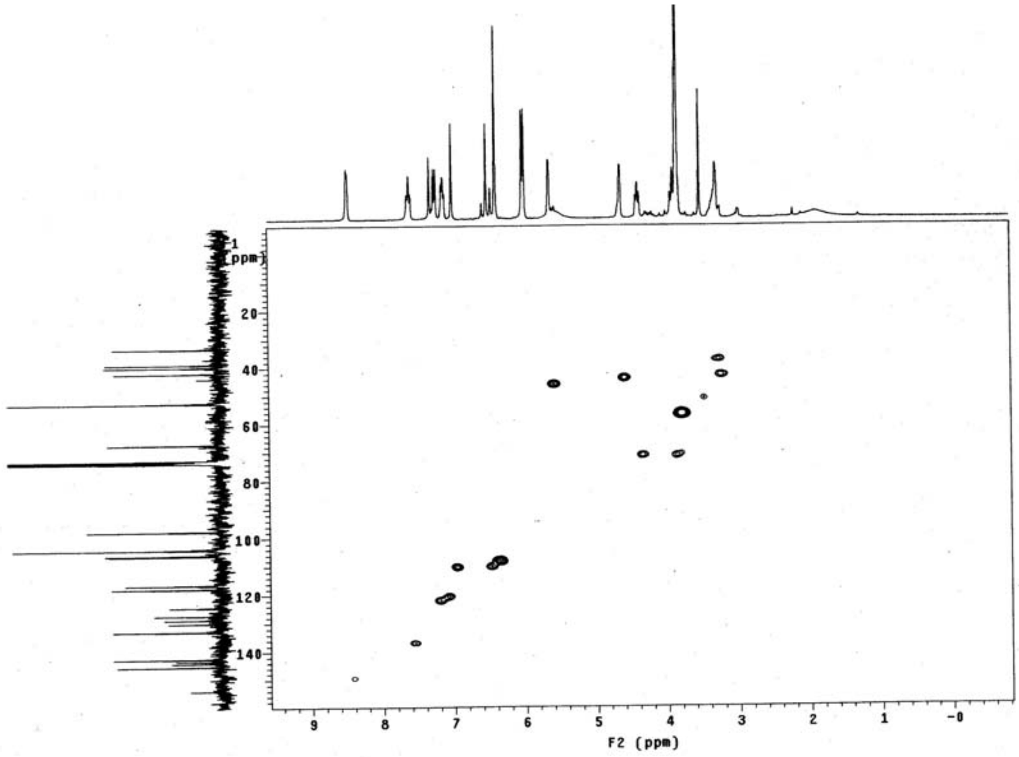

6.6. MS spectrum of compound 4'S.

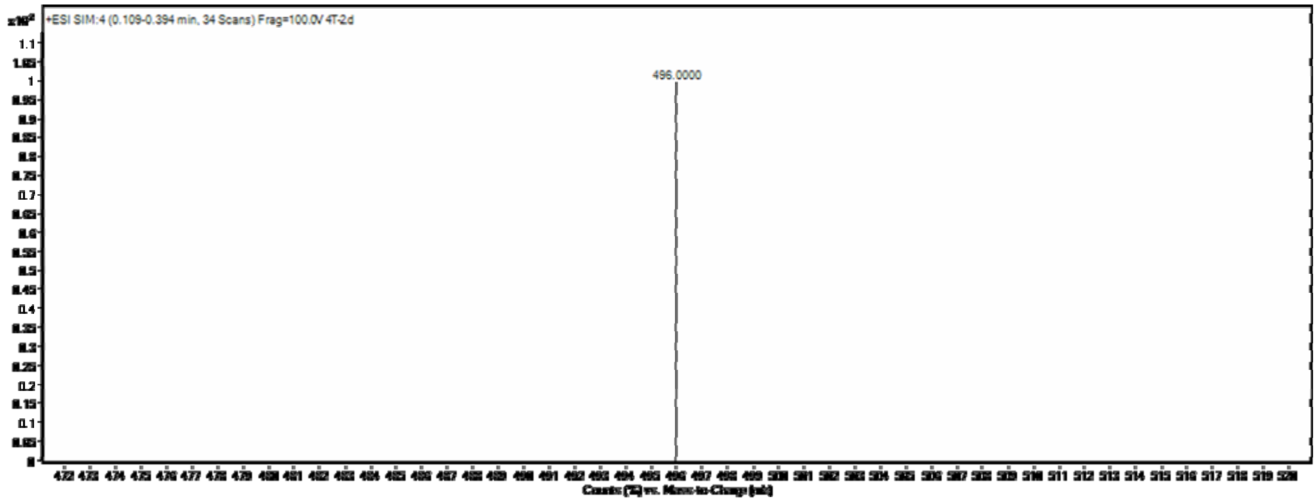

4β-S-(pyrimidine-2)-4-deoxy-4'-demethyl-podophyllotoxin (4'S)

^{1}H NMR (300 MHz, $CDCl_3$, δ): 8.468 (d, *J* = 7.2 Hz, 1H), 7.599 (t, *J* = 6.9 Hz, 1H), 7.253 (d, *J* = 7.8 Hz, 1H), 7.122 (t, *J* = 6.0 Hz, 1H), 6.998 (s, 1H), 6.512 (s, 1H), 6.388 (s, 2H), 6.009 (d, *J* = 7.2 Hz, 2H), 5.643 (t, *J* = 6.0 Hz, 1H), 4.645 (d, *J* = 4.2 Hz, 1H), 4.391 (t, *J* = 8.1 Hz, 1H), 3.897 (t, *J* = 9.0 Hz, 1H), 3.837 (s, 6H), 3.359-3.240 (m, 2H); ^{13}C NMR (75 MHz, $CDCl_3$, δ): 175.170, 158.169, 149.399, 148.178, 147.469, 146.617, 136.854, 134.157, 132.823, 131.404, 128.566, 121.896, 120.562, 110.316, 110.118, 108.131 (2C), 101.717, 71.234, 56.674 (2C), 46.031, 43.845, 42.852, 37.317.

ESI-MS: calc'd for $C_{25}H_{22}N_2O_7S$ $[M+H]^+$: 496.11, found 496.00 $[M+H]^+$.

7.1. ^{1}H NMR spectrum of compound 5'S.

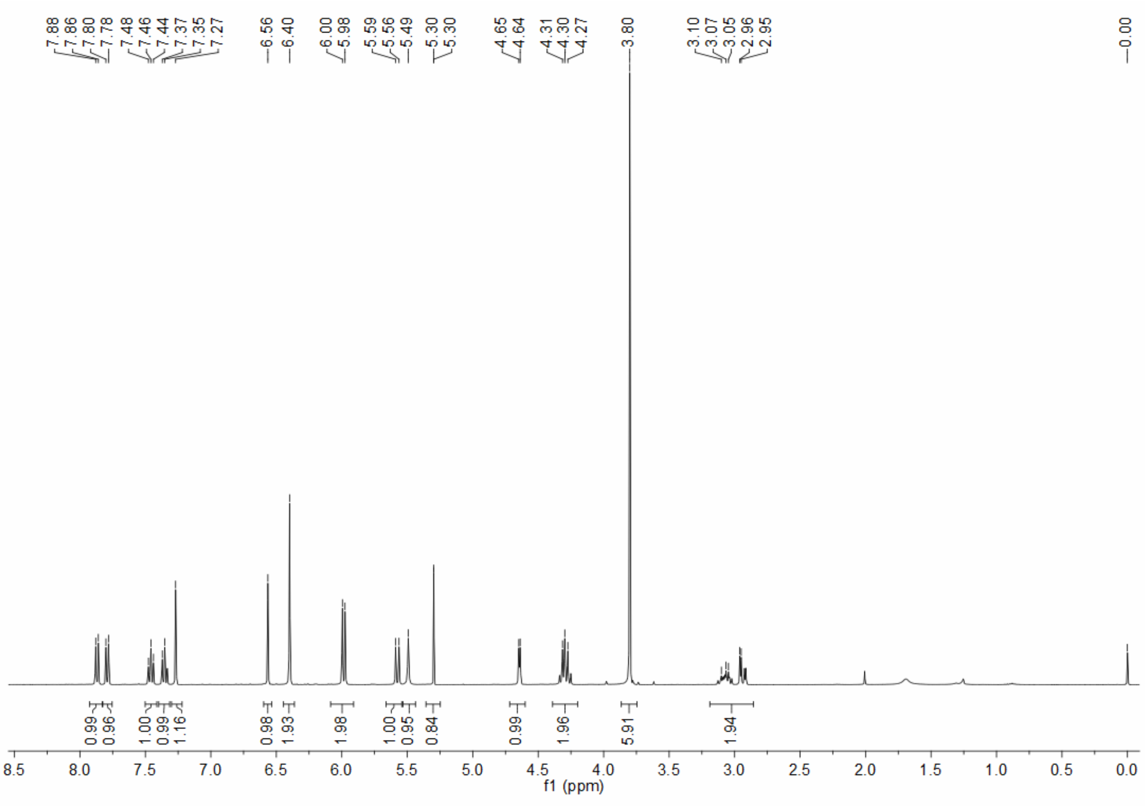

7.2. ^{13}C NMR spectrum of compound 5'S.

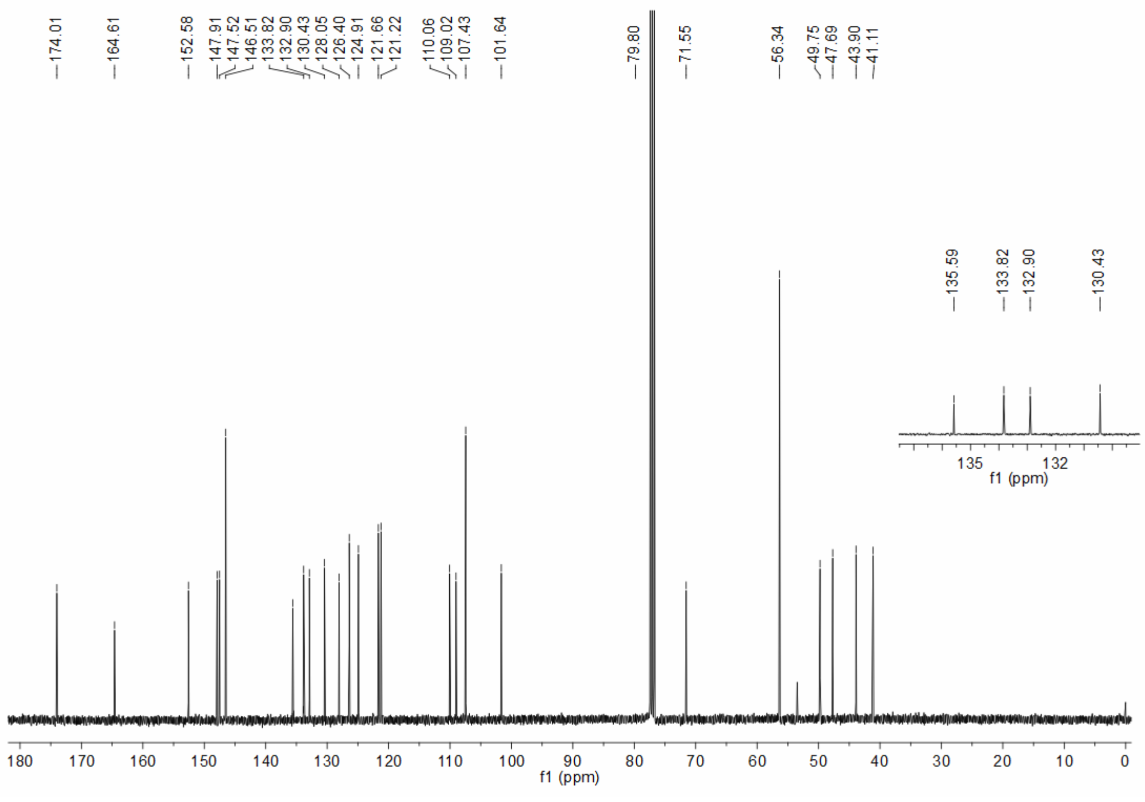

7.3. ^{1}H-^{1}H COSY spectrums for compound 5'S.

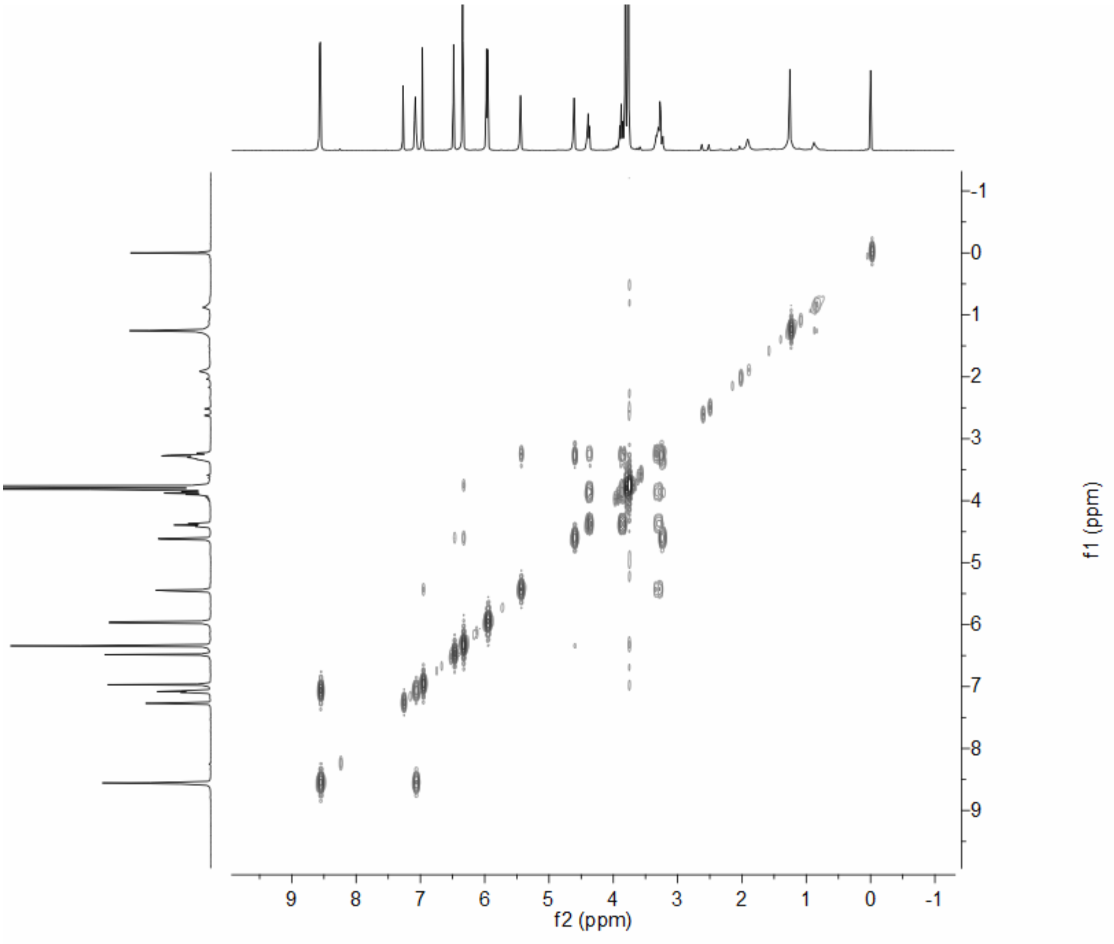

7.4. HMBC spectrums for compound 5'S.

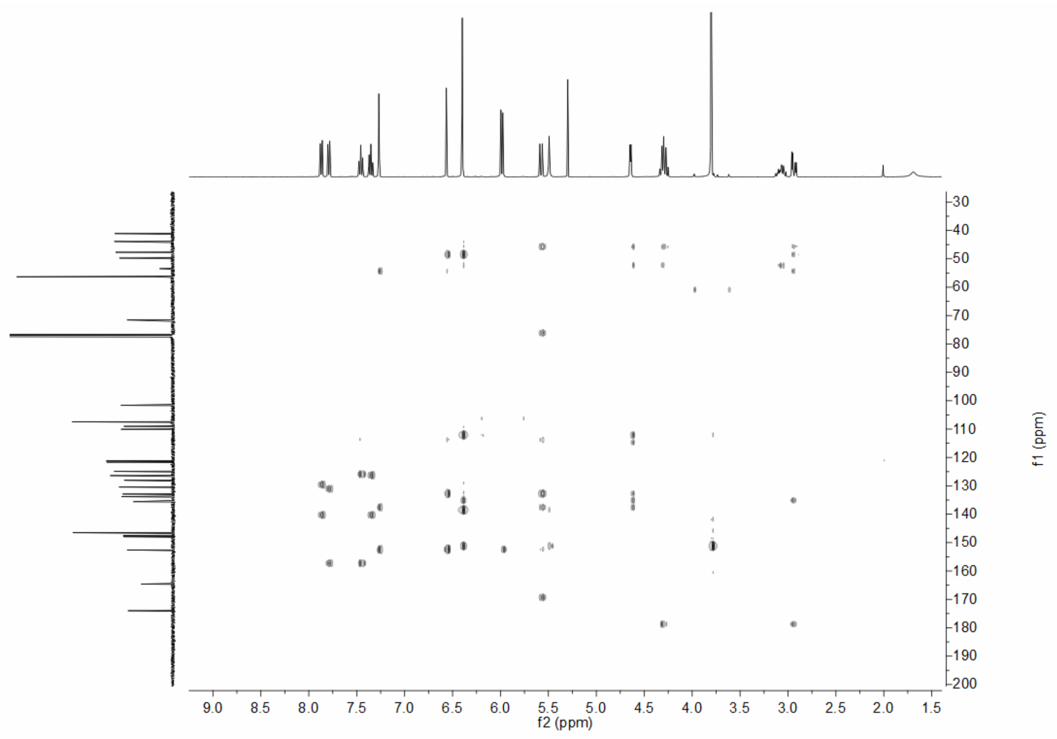

7.5. HSQC spectrums for compound 5'S.

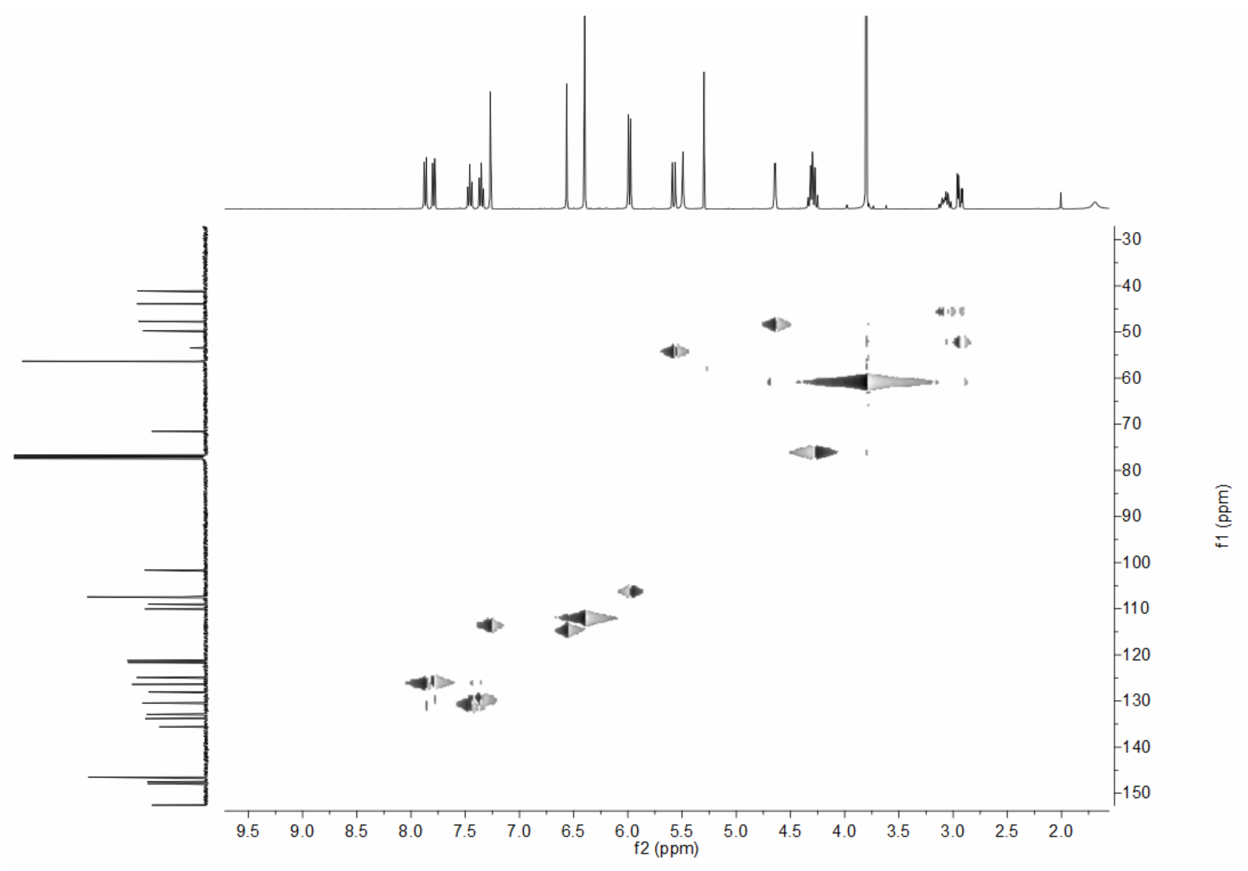

7.6. MS spectrum of compound 5'S.

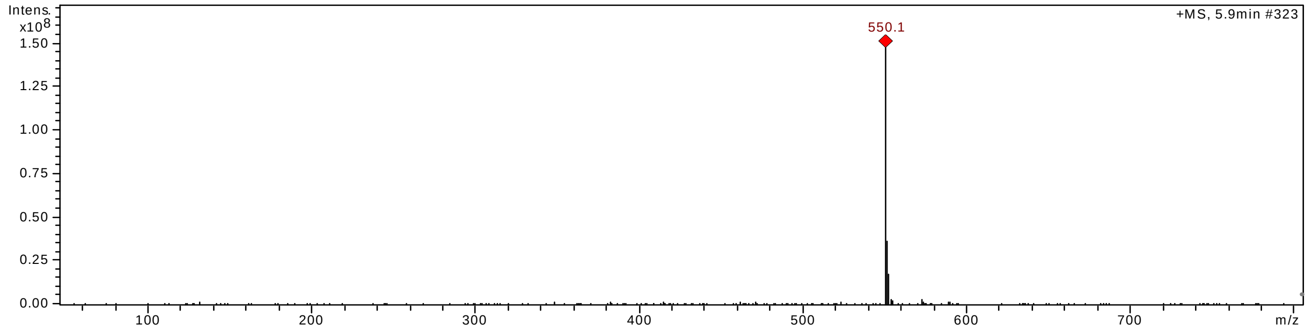

4β-S-(benzothiazole-2)-4-deoxy-4'-demethyl-podophyllotoxin (5'S)

^{1}H NMR (400 MHz, $CDCl_3$, δ): 7.88 (d, *J* = 8.0 Hz, 1H), 7.80 (d, *J* = 8.0 Hz, 1H), 7.46 (t, *J* = 8.0 Hz, 1H), 7.35 (t, *J* = 8.0 Hz, 1H), 7.27 (s, 1H), 6.56 (s, 1H), 6.40 (s, 2H), 6.00 (d, *J* = 8.0 Hz, 2H), 5.59 (d, *J* = 4.0 Hz, 1H), 4.65 (d, *J* = 4.0 Hz, 1H), 4.34-4.25 (m, 2H), 3.80 (s, 6H), 3.13-3.02 (m, 1H), 2.96-2.91 (m, 1H); ^{13}C NMR(101 MHz, $CDCl_3$, δ): 174.01, 164.61, 152.58, 147.91, 147.52, 146.51 (2C), 135.59, 133.82, 132.90, 130.43, 128.05, 126.40, 124.91, 121.66, 121.22, 110.06, 109.02, 107.43 (2C), 101.64, 71.55, 56.34 (2C), 49.75, 47.69, 43.90, 41.11.

ESI-MS: calc'd for $C_{28}H_{23}NO_7S_2$ $[M+H]^+$: 550.09, found 550.1 $[M+H]^+$.

8.1. ^{1}H NMR spectrum of compound 6'S.

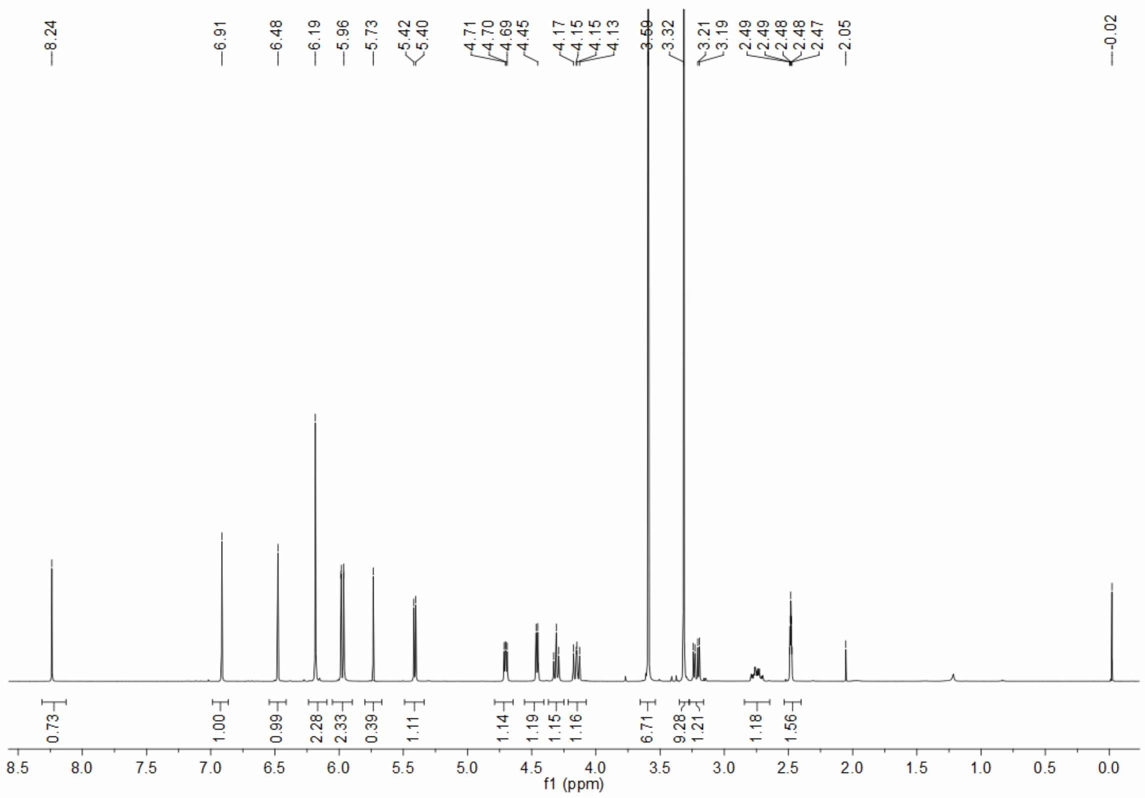

8.2. ^{13}C NMR spectrum of compound 6'S.

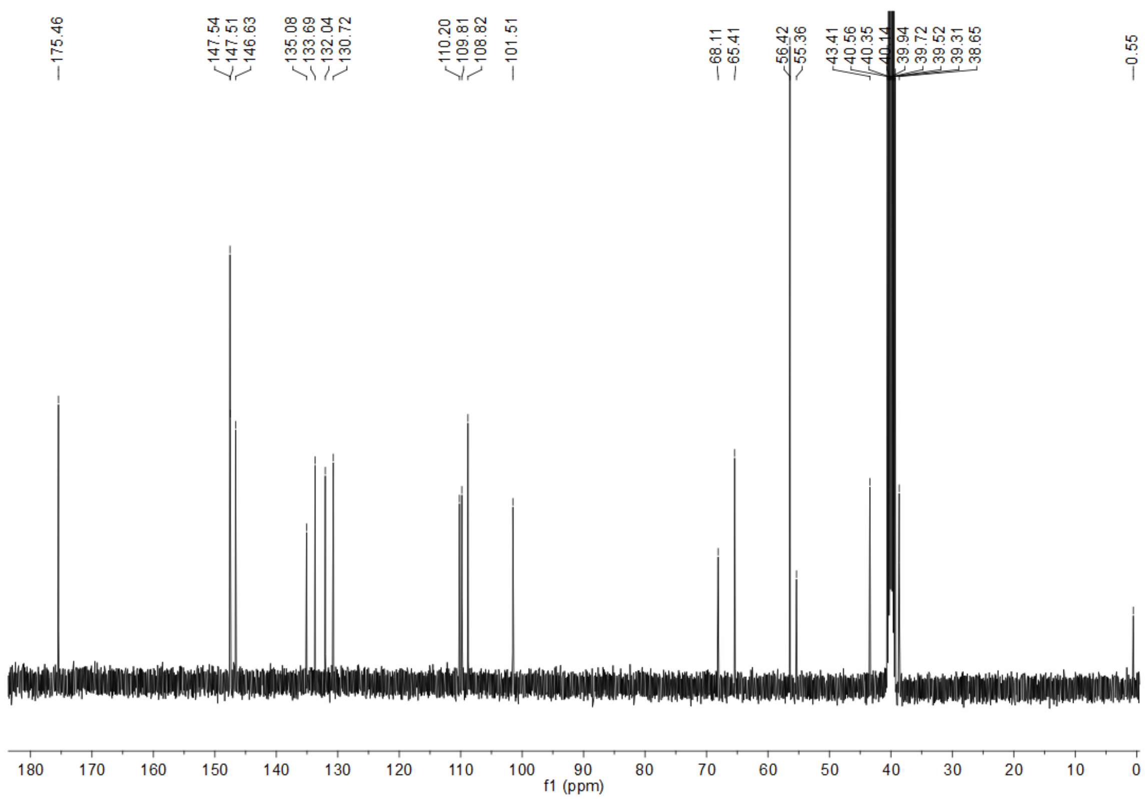

8.3. ^{1}H-^{1}H COSY spectrums for compound 6'S.

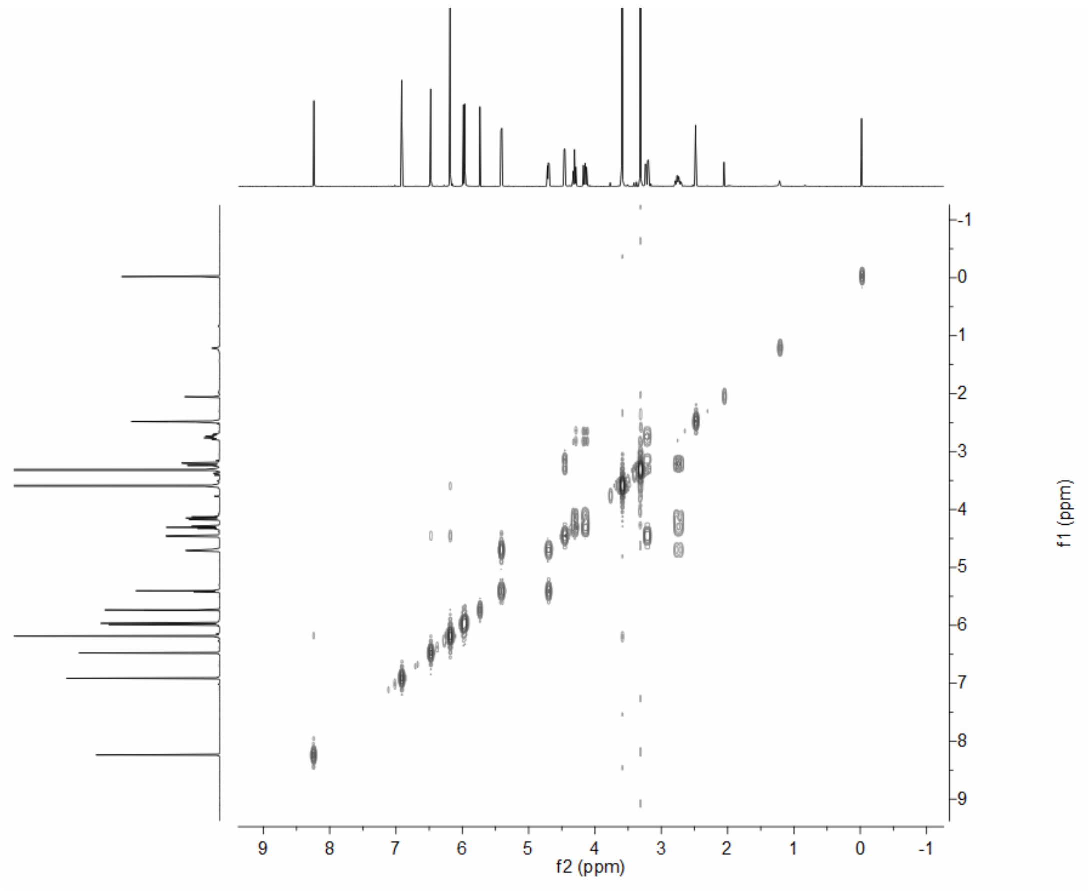

8.4. HMBC spectrums for compound 6'S.

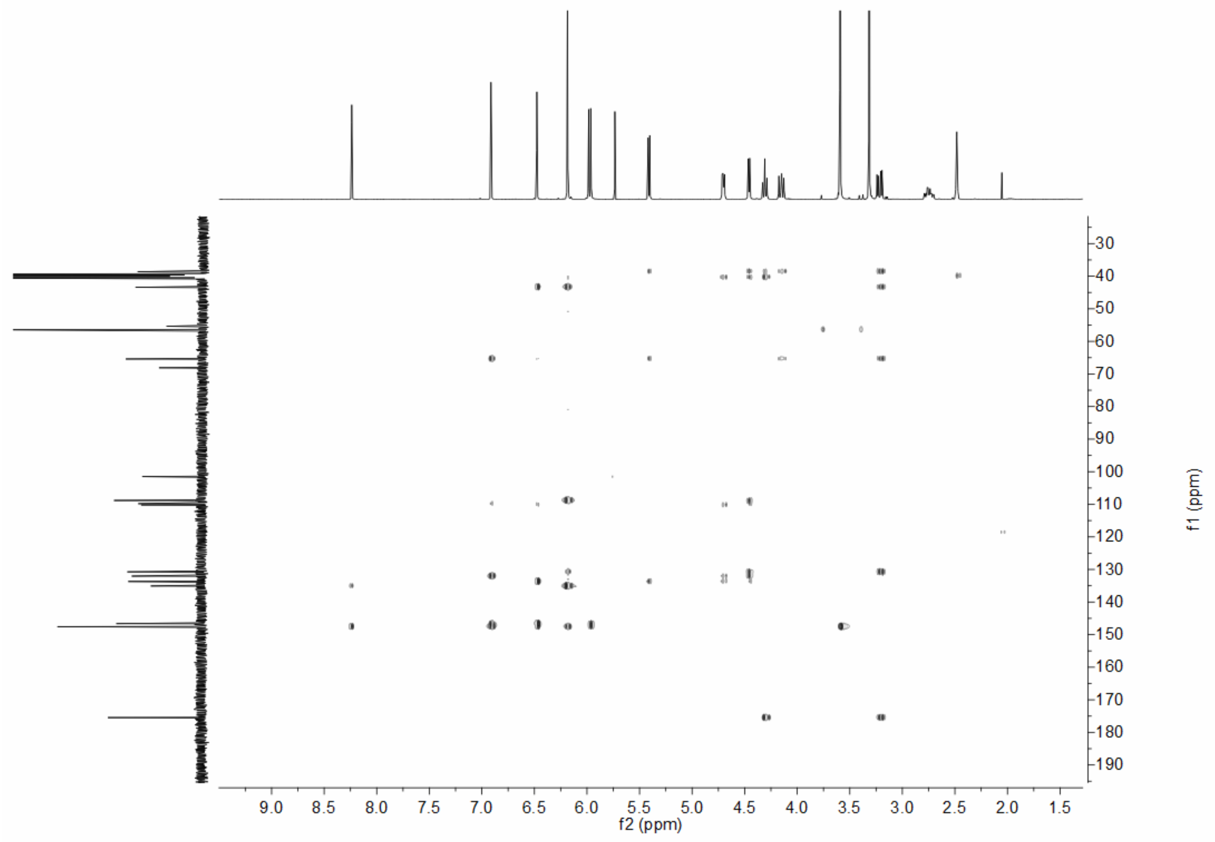

8.5. HSQC spectrums for compound 6'S.

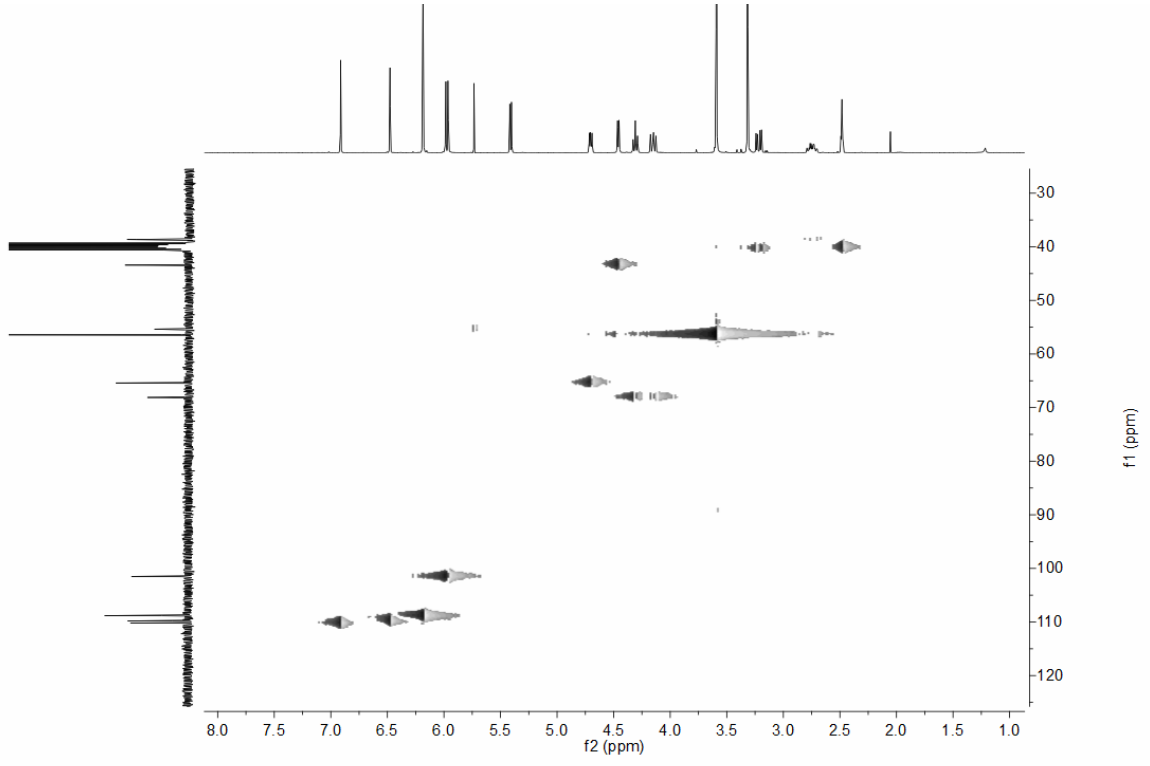

8.6. MS spectrum of compound 6'S.

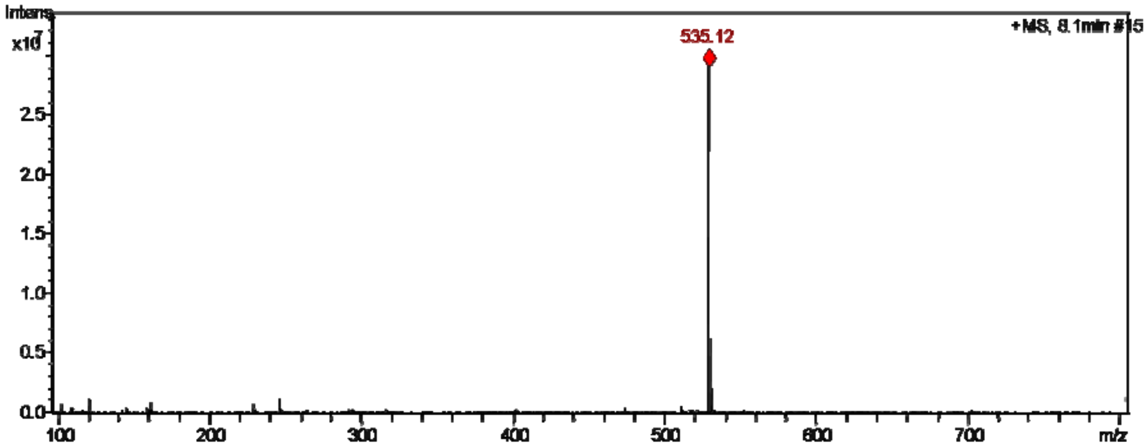

4β-S-(purine-6)-4-deoxy-4'-demethyl-podophyllotoxin (6'S)

^{1}H NMR (400 MHz, DMSO): δ 8.24 (s, 1H), 6.91 (s, 1H), 6.48 (s, 1H), 6.19 (s, 2H), 5.98 (d, *J* = 8.0 Hz, 2H), 5.42 (d, *J* = 8.0 Hz, 1H), 4.71 (dd, *J* = 4.0 Hz, 1H), 4.47 (d, *J* = 8.0 Hz, 1H), 4.31 (t, J = 8.0 Hz, 1H), 4.15 (t, *J* = 8.0 Hz, 1H), 3.59 (s, 6H), 3.24-3.19 (m, 1H), 2.79 - 2.70 (m, 1H); ^{13}C NMR (101 MHz, DMSO): δ 175.46 (2C), 147.54, 147.51 (2C), 146.63 (2C), 135.08, 133.69 (2C), 132.04, 130.72 (2C), 110.20, 109.81, 108.82 (2C), 101.51, 68.11, 65.41, 56.42 (2C), 55.36, 43.41, 38.65.

ESI-MS: calc'd for $C_{27}H_{25}NO_7S$ $[M+H]^+$: 534.12, found 535.12 $[M+H]^+$.

9.1. ^{1}H NMR spectrum of compound 1N.

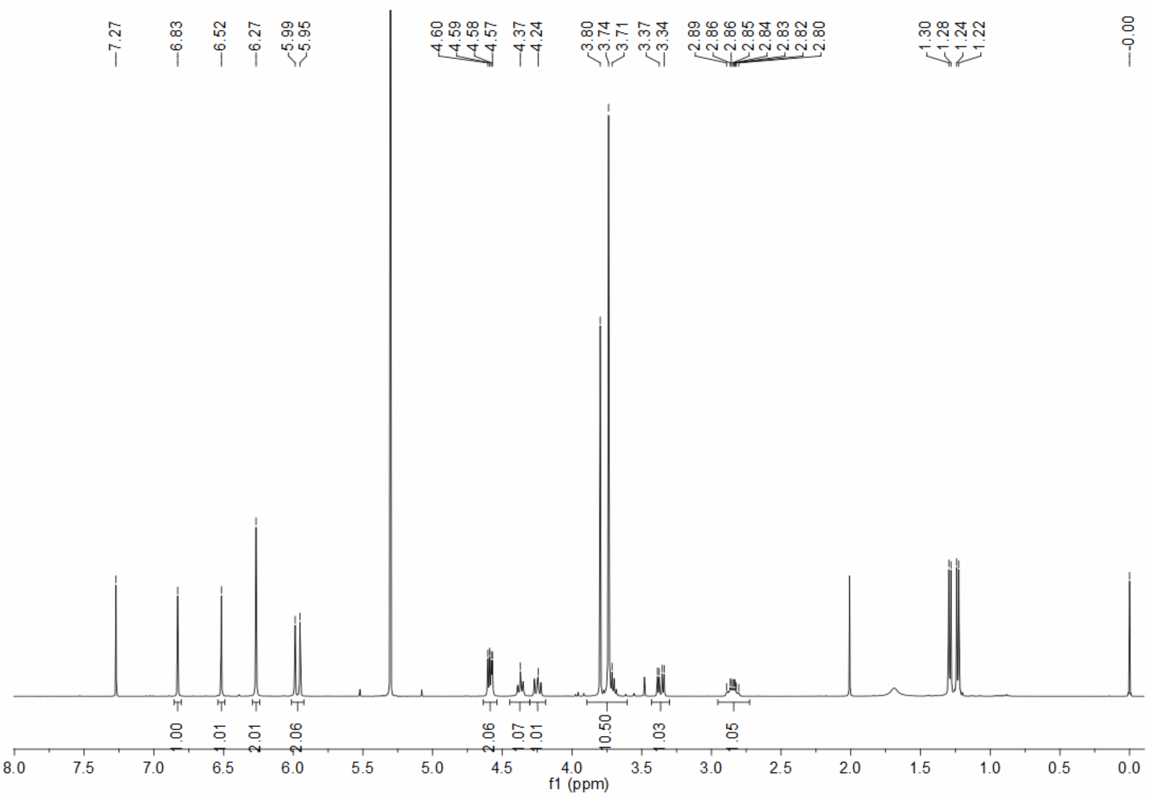

9.2. ^{13}C NMR spectrum of compound 1N.

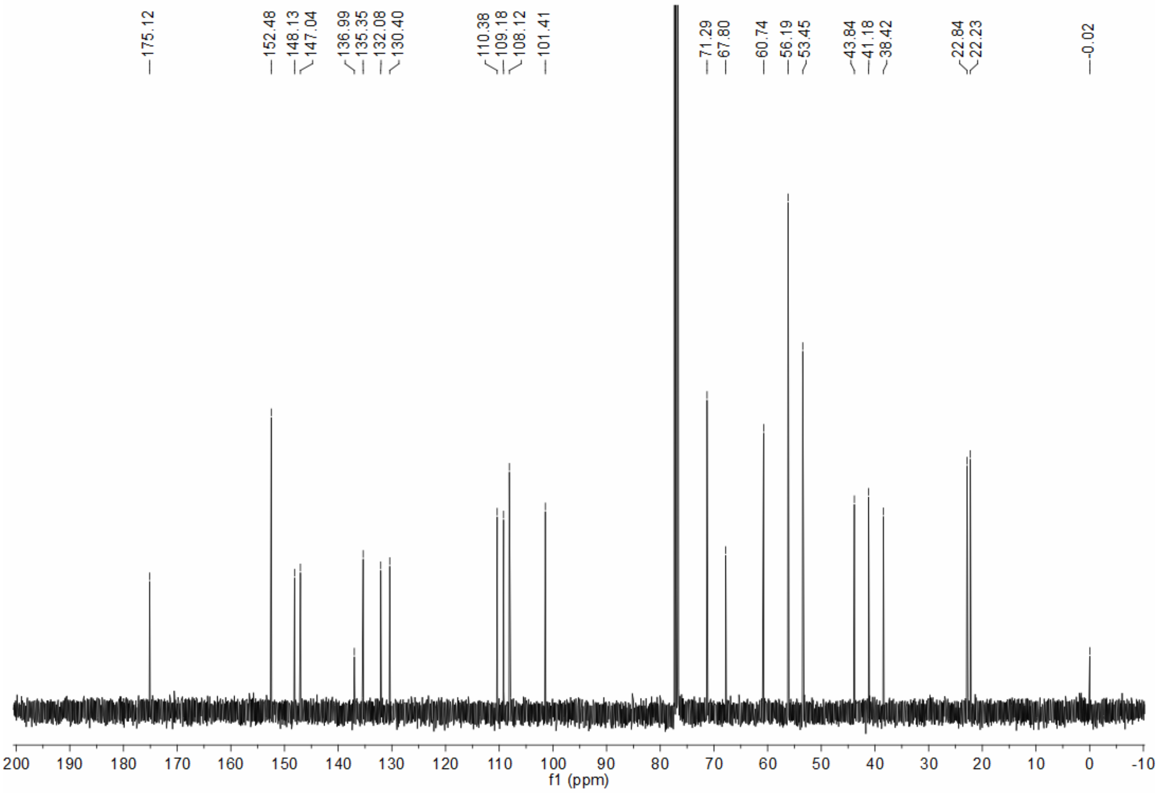

9.3. ^{1}H-^{1}H COSY spectrums for compound 1N.

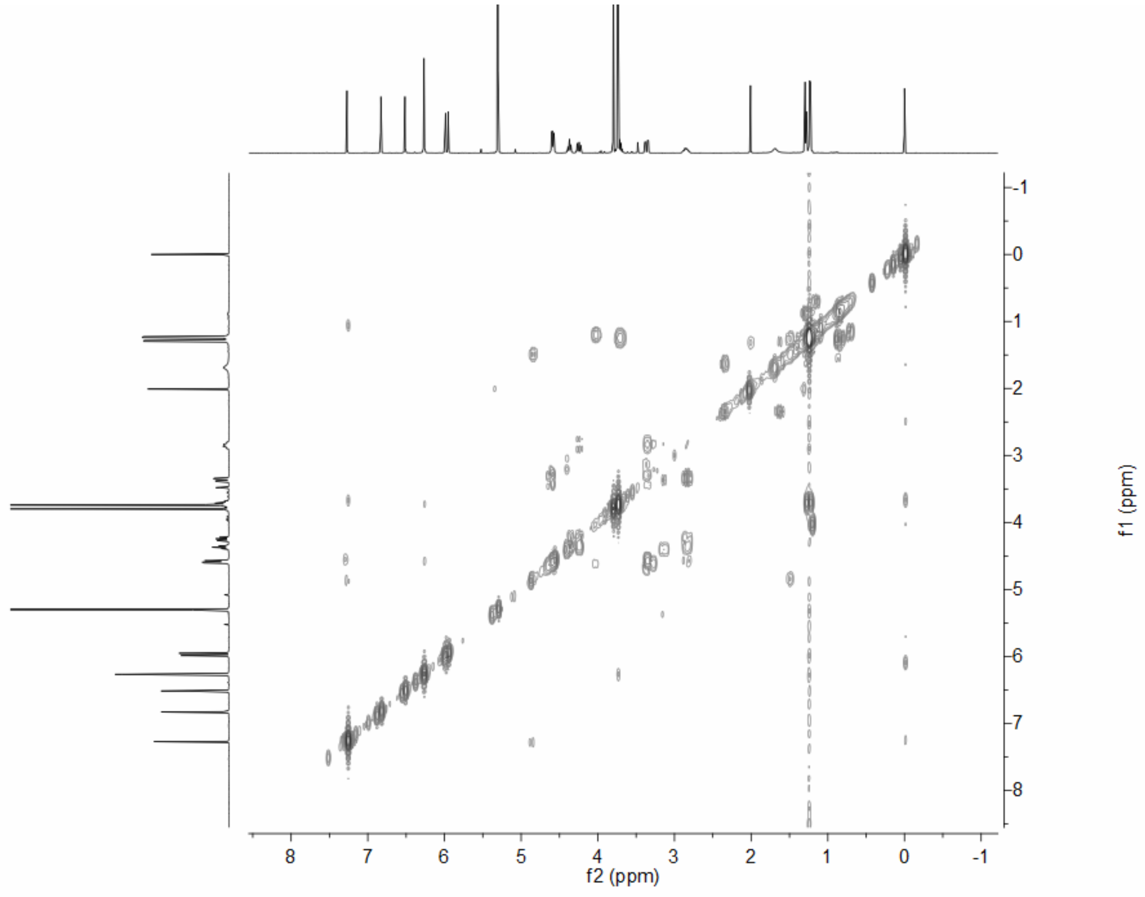

9.4. HMBC spectrums for compound 1N.

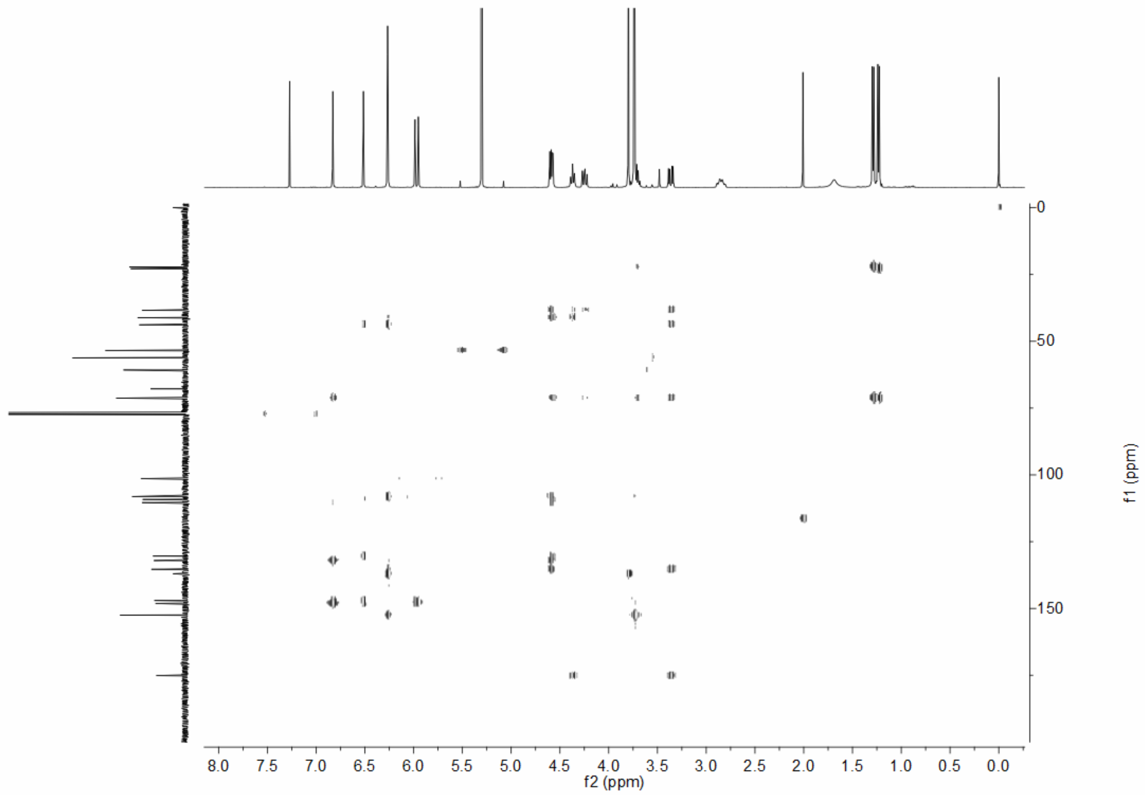

9.5. HSQC spectrums for compound 1N.

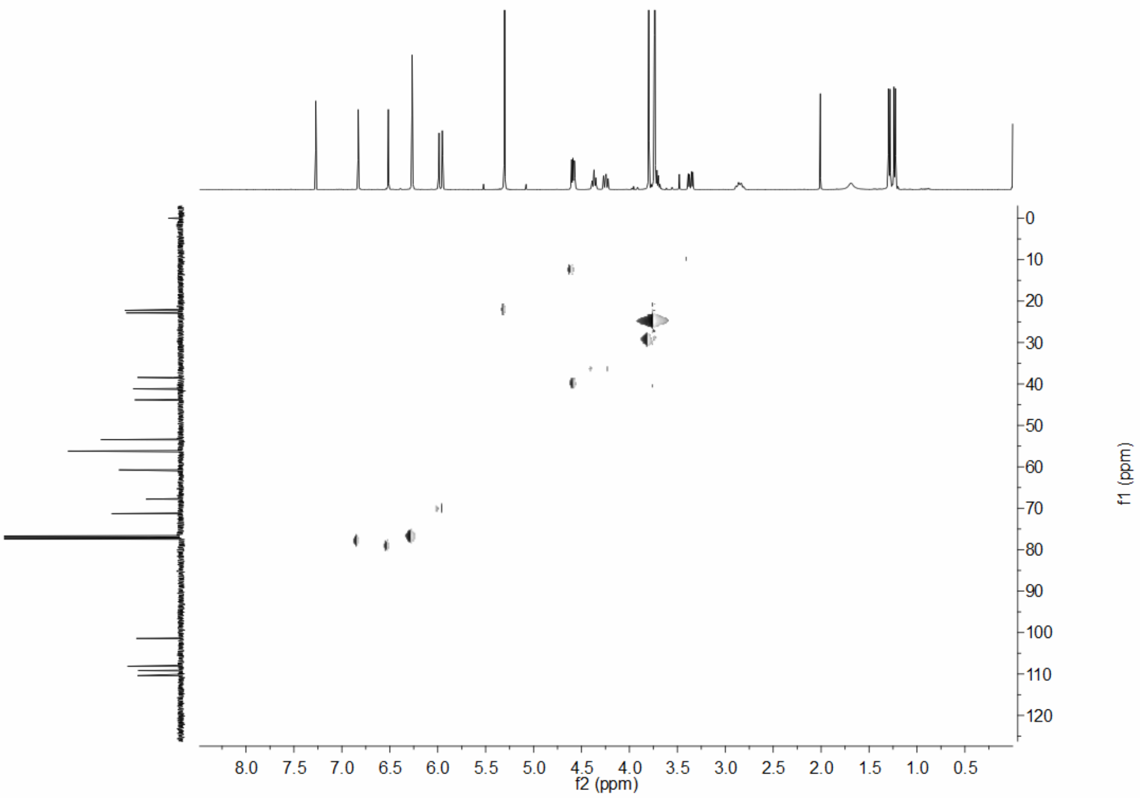

9.6. MS spectrum of compound 1N.

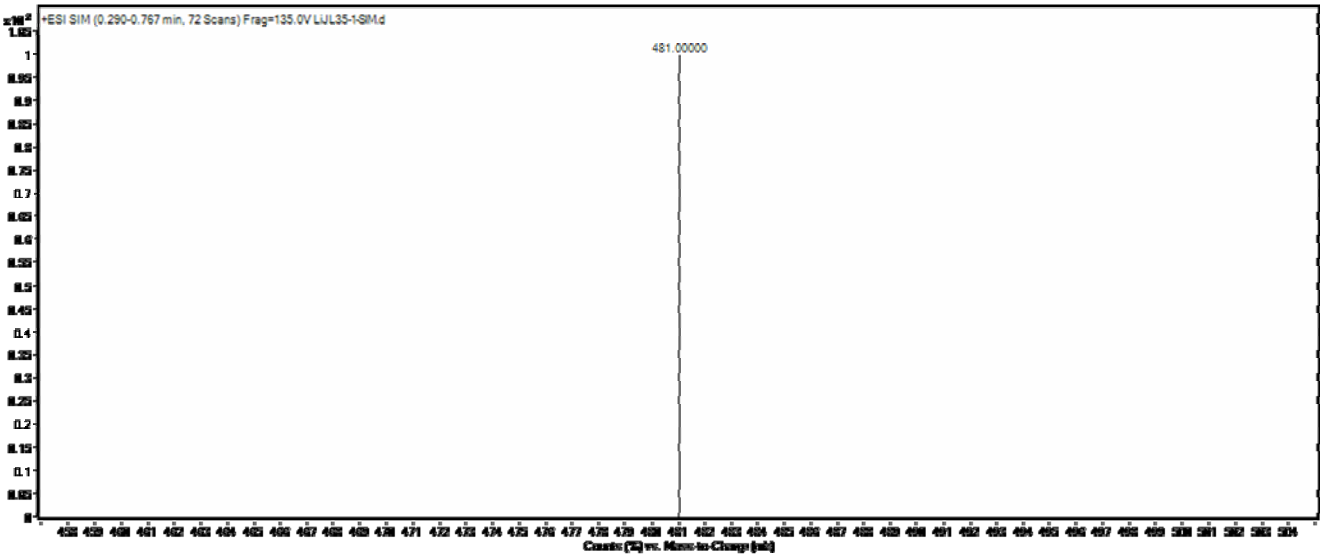

4β-N-(1,2,4-trizole-3)-4-deoxy-podophyllotoxin (1N).

^{1}H NMR (400 MHz, $CDCl_3$): δ 6.83 (s, 1H), 6.52 (s, 1H), 6.27 (s, 2H), 5.99 (d, *J* = 16 Hz, 2H), 4.60 (dd, *J* = 4.0 Hz, 2H), 4.37 (t, *J* = 4.0 Hz, 1H), 4.24 (t, *J* = 4.0 Hz, 1H), 3.80 (s, 3H), 3.74 (s, 6H), 3.71 (t, *J* = 4.0 Hz, 1H), 3.39-3.34 (dd, *J* = 8.0 Hz, 1H), 2.89-2.80 (m, 1H); ^{13}C NMR (101 MHz, $CDCl_3$): δ 174.842, 158.605, 152.729 (3C), 148.617, 147.685, 137.263, 135.708, 132.541, 127.554, 110.428, 110.099, 108.460 (2C), 101.882, 70.754, 61.025, 56.465 (2C), 49.103, 43.899, 42.421, 37.489;

ESI-MS: calc'd for $C_{24}H_{24}N_4O_7$ $[M+H]^+$: 481.16, found 481.00 $[M+H]^+$.

10.1. ^{1}H NMR spectrum of compound 2N.

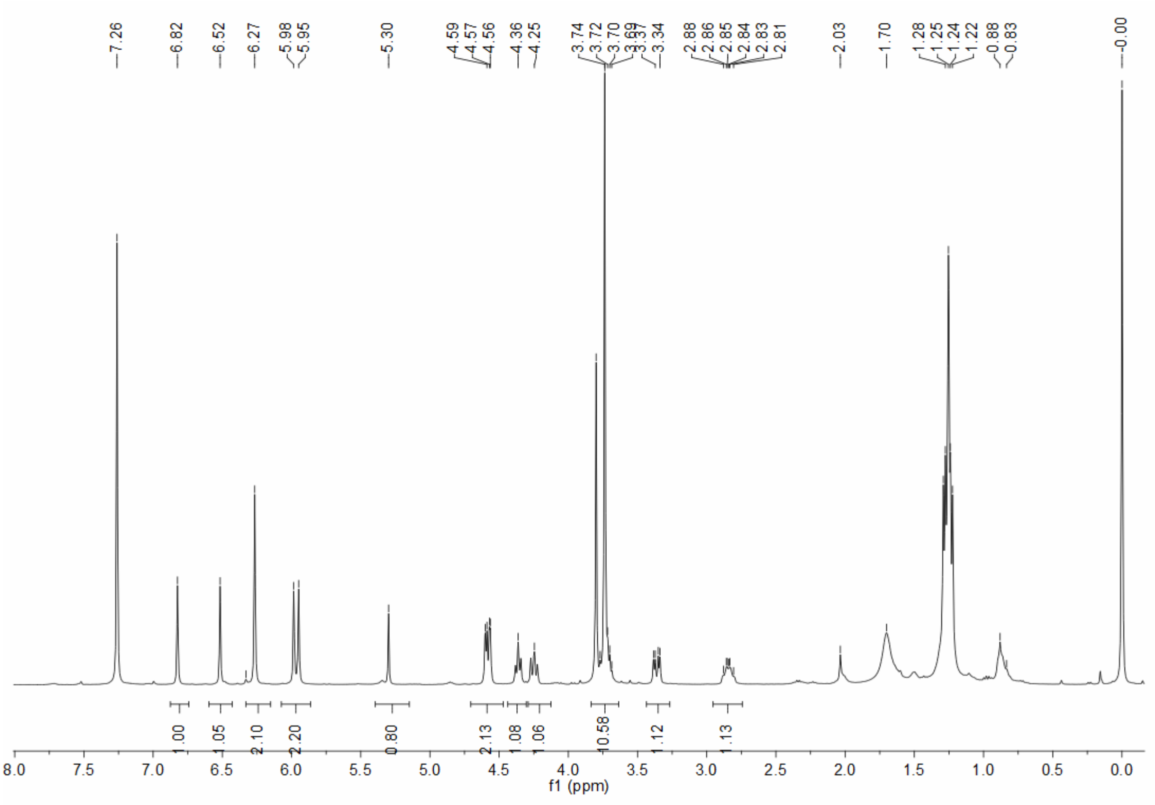

10.2. ^{13}C NMR spectrum of compound 2N.

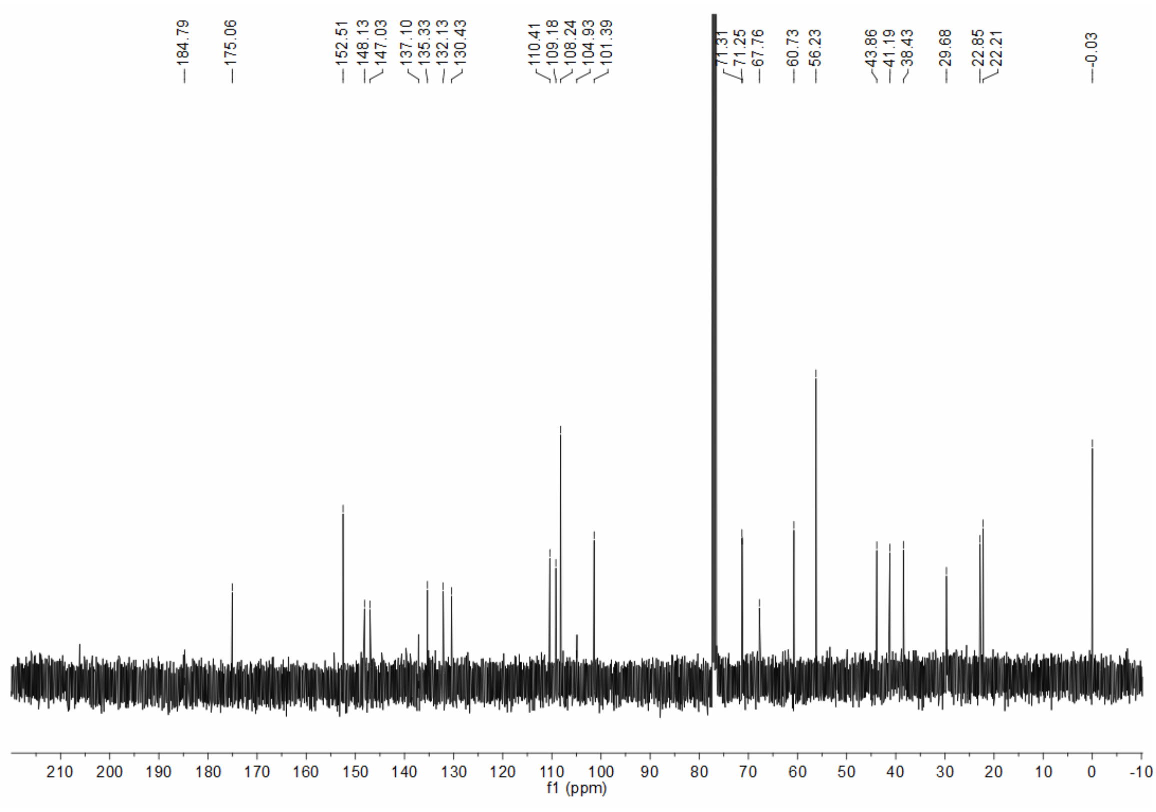

10.3. ^{1}H-^{1}H COSY spectrums for compound 2N.

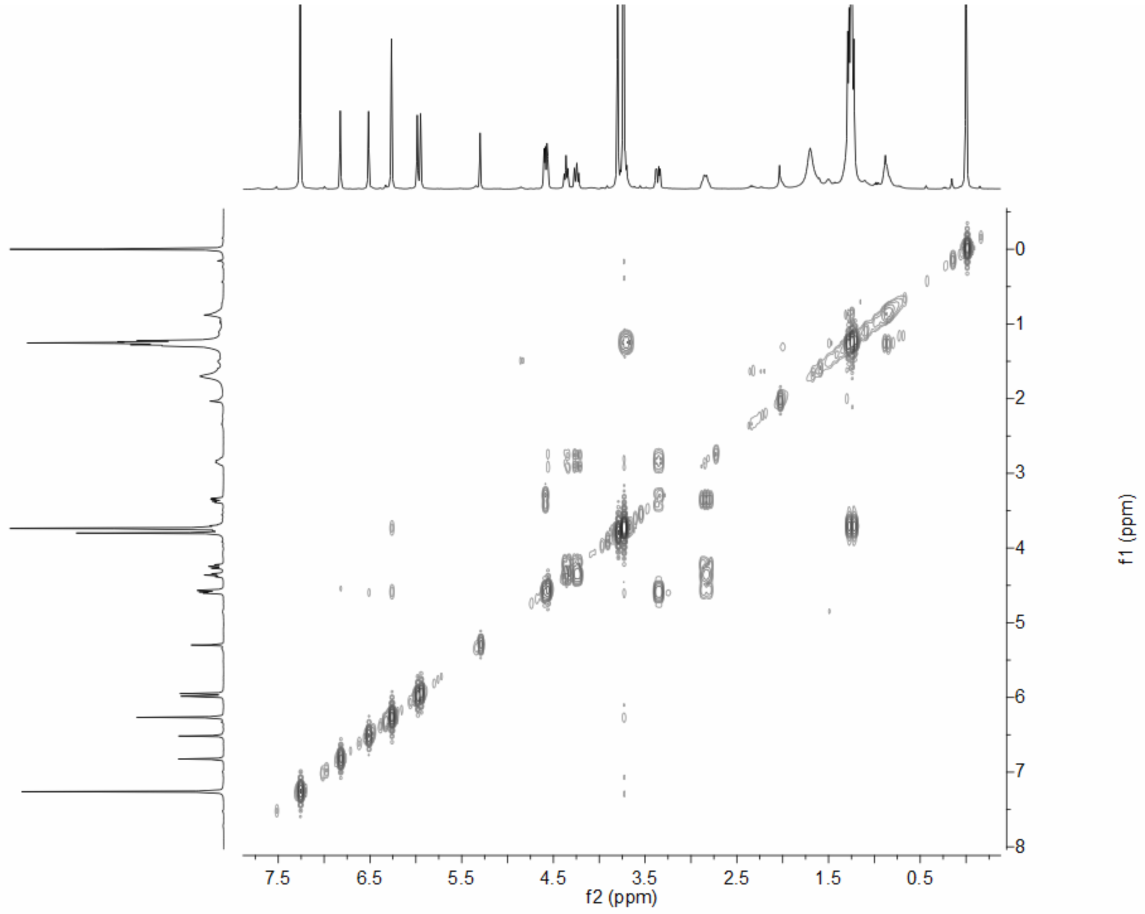

10.4. HMBC spectrums for compound 2N.

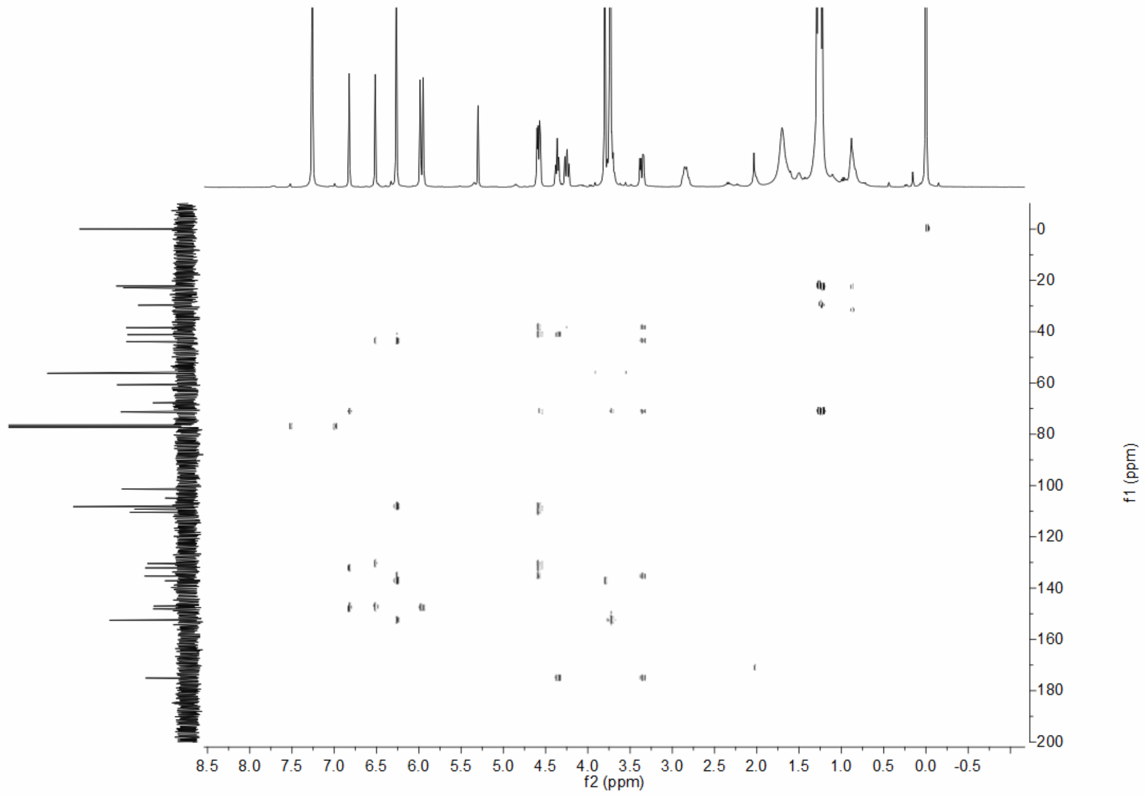

10.5. HSQC spectrums for compound 2N

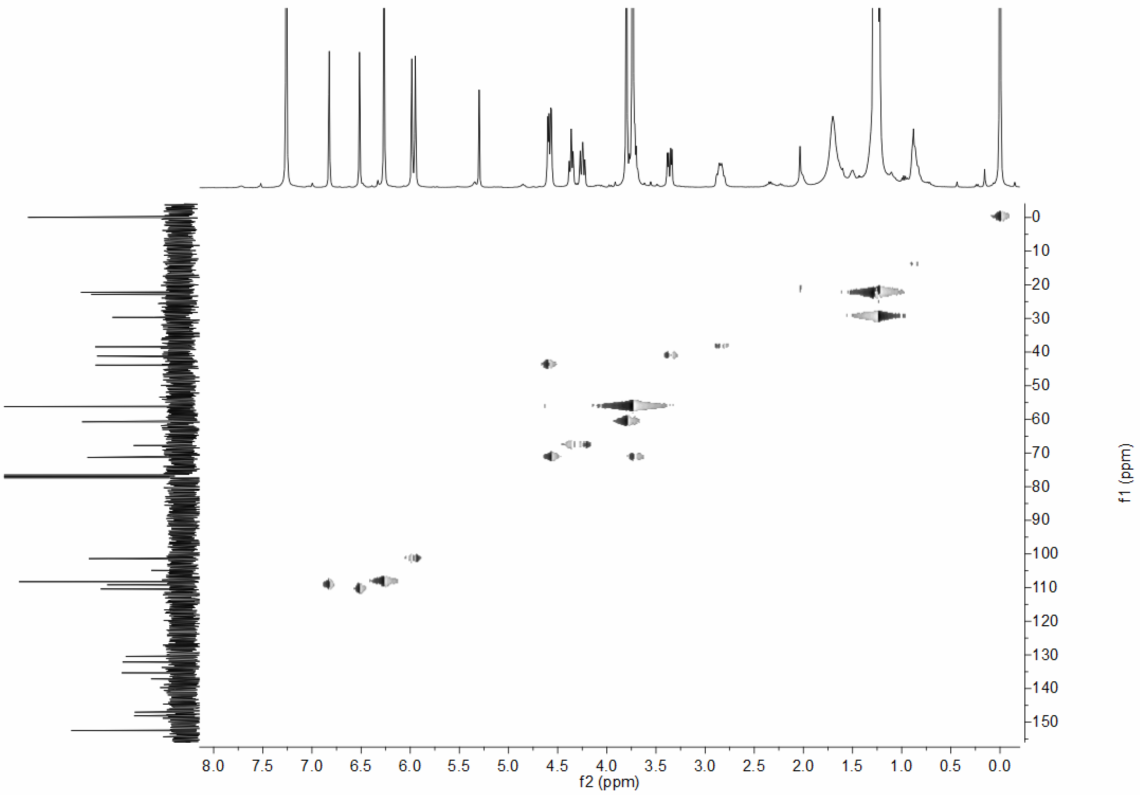

10.6. MS spectrum of compound 2N

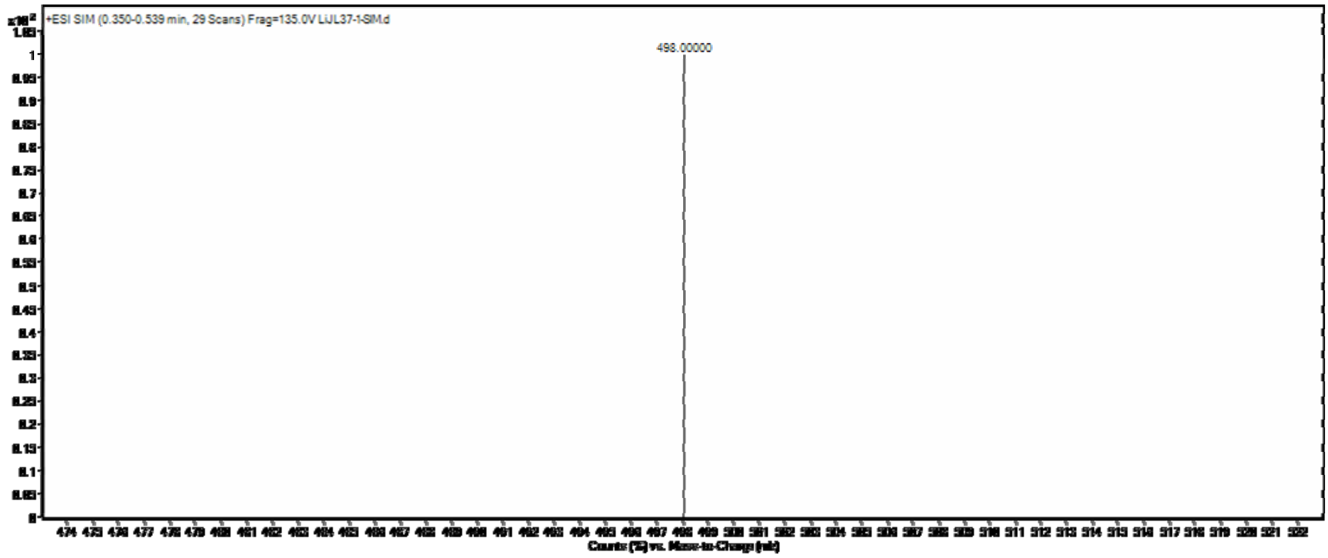

4β-N-(1,3,4-thiodiazole-2)-4-deoxy-podophyllotoxin (2N).

^{1}H NMR (400 MHz, CDCl$_3$): δ 6.82 (s, 1H), 6.52 (s, 1H), 6.27 (s, 2H), 5.98 (d, *J* = 3.6 Hz, 2H), 5.30 (s, 1H), 5.46 (dd, 2H, *J* = 4.0 Hz), 4.36 (t, 1H, *J* = 8.0 Hz), 4.24 (t, 1H, *J* = 8.0 Hz), 3.80 (s, 3H), 3.74 (s, 6H), 3.72 (t, *J* = 8.0 Hz, 1H), 3.38 (m, *J* = 4.0 Hz, 1H), 2.88-2.81 (m, 1H); ^{13}C NMR(101 MHz, CDCl$_3$): δ 175.06, 152.51 (2C), 148.13, 147.03, 137.14, 135.33, 132.13, 130.43, 110.41, 109.18, 108.24 (2C), 104.89, 101.39, 71.31, 71.25, 67.76, 60.73, 56.23 (2C), 43.86, 41.19, 38.43;

ESI-MS: calc'd for $C_{24}H_{23}N_3O_7S$: 498.52, found 498.00 [M+H]$^+$.

11.1. ^{1}H NMR spectrum of compound 3N.

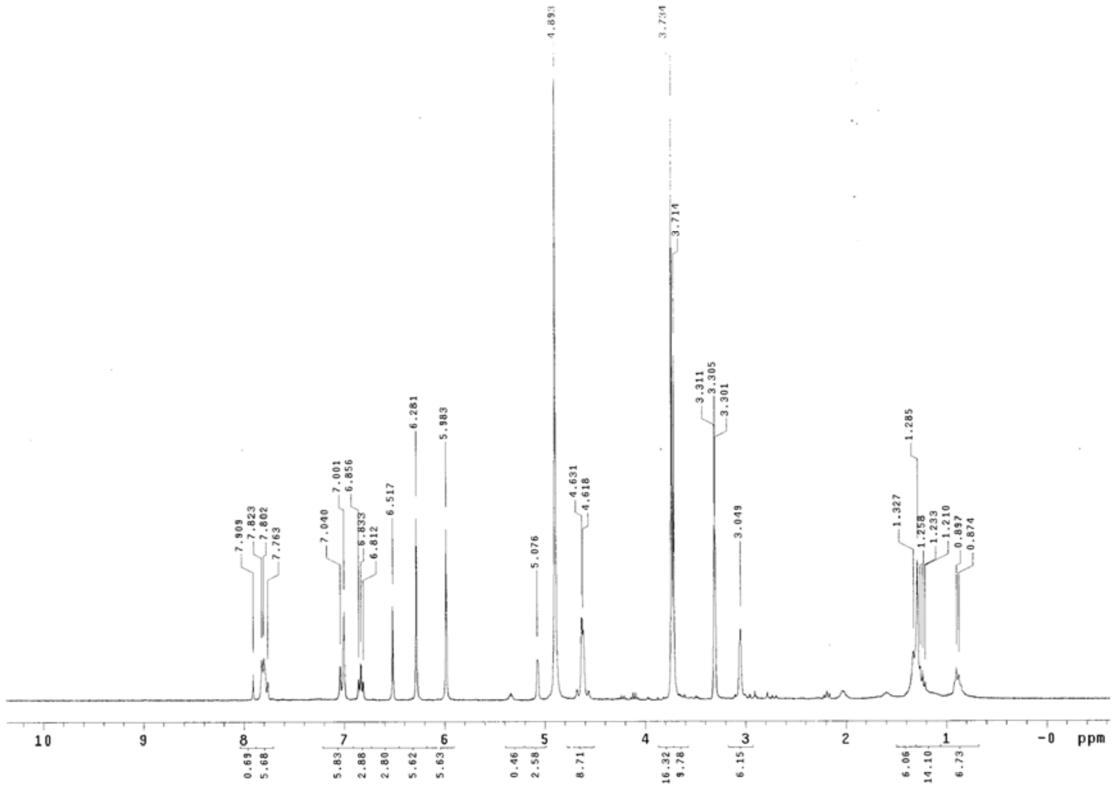

11.2. ^{13}C NMR spectrum of compound 3N.

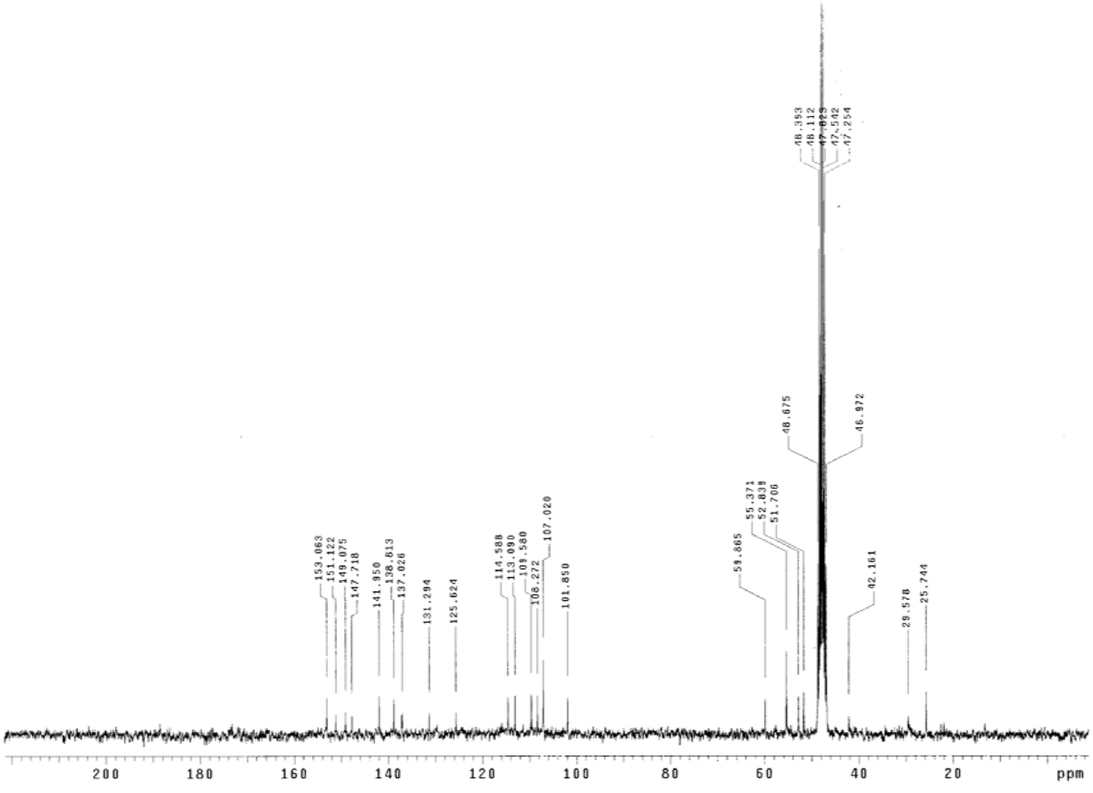

11.3. ^{1}H-^{1}H COSY diagram of compound 3N

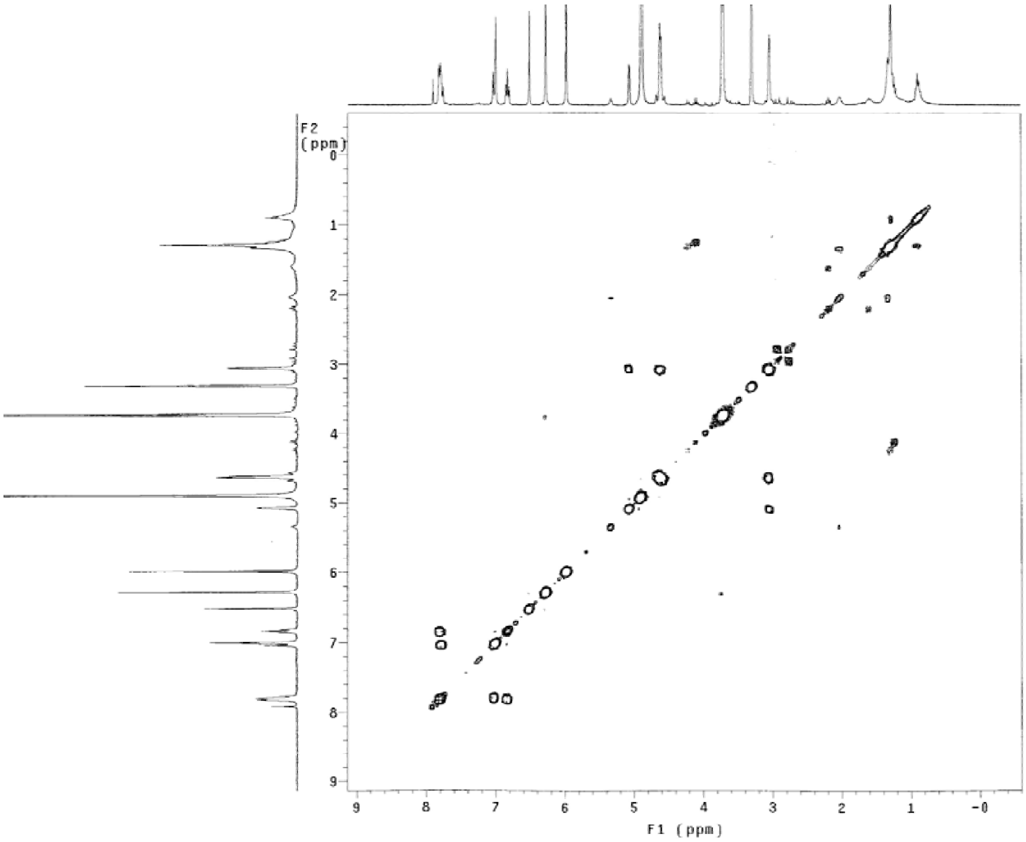

11.4. HMBC diagram of compound 3N.

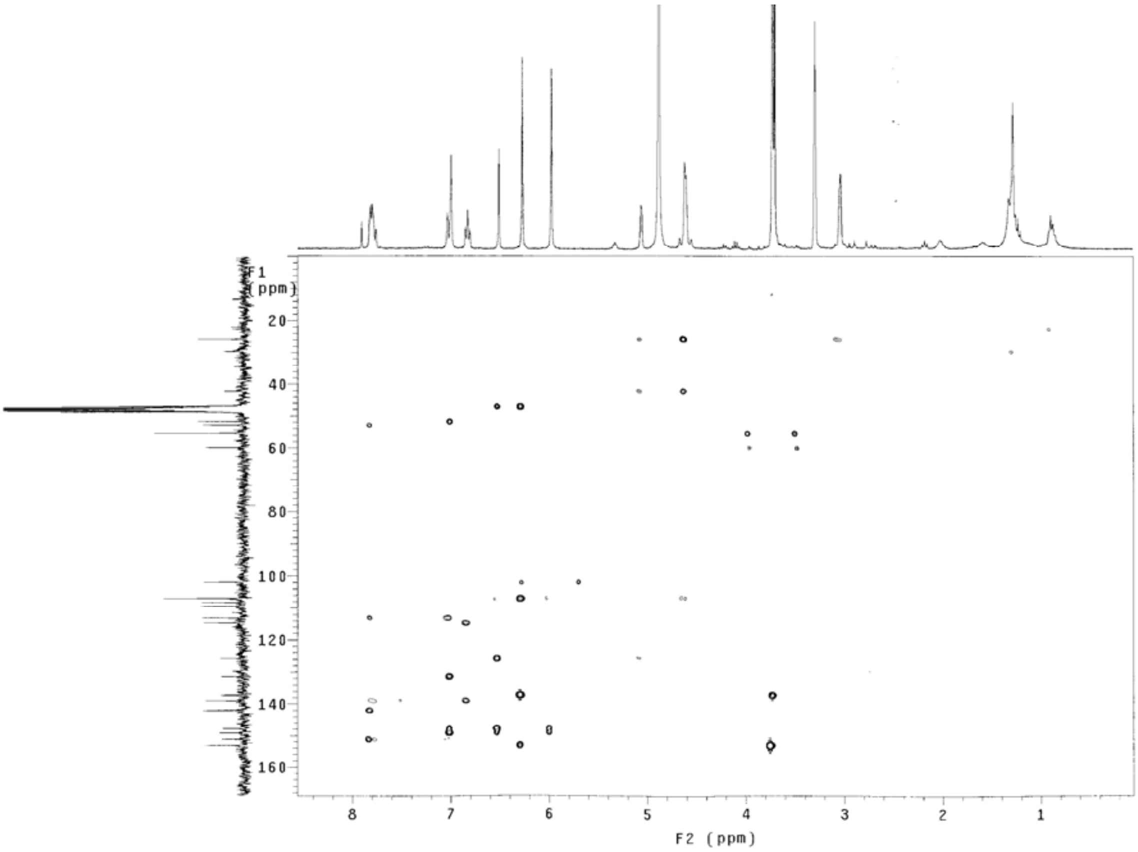

11.5. HSQC diagram of compound 3N.

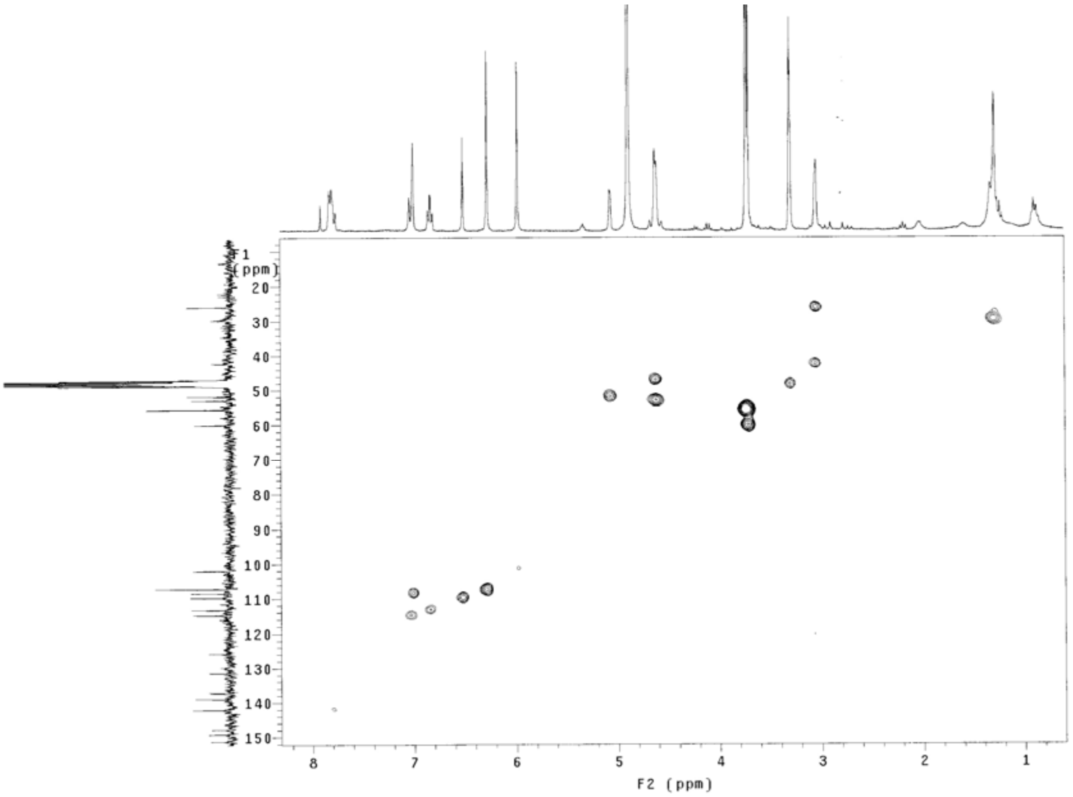

11.6. MS spectrum of compound 3N

4β-N-(pyridine-2)-4-deoxy-podophyllotoxin (3N).

^{1}H NMR (300 MHz, CD_3OD): δ 8.823 (d, *J* = 6.3 Hz, 2H), 7.040-7.001 (m, 2H), 6.833 (t, *J* = 7.8 Hz,1H), 6.517 (s, 2H), 6.281 (s, 2H), 5.983 (s, 2H), 5.076 (s, 1H), 4.631 (d, *J* = 4.5 Hz, 3H), 3.734 (s, 6H), 3.714 (s, 3H), 3.049 (m, 2H); ^{13}C NMR(75 MHz, CD_3OD): δ 153.063 , 151.122, 149.075, 147.718, 141.950, 138.813 (2C), 137.026, 131.294, 125.624, 114.588, 113.090, 109.580, 108.272, 107.020 (2C), 101.850, 59.865, 55.371 (2C), 52.839, 51.706, 42.161, 29.578, 25.744.

ESI-MS: calc'd for $C_{27}H_{26}N_2O_7$ $[M+H]^+$: 491.18, found 491.13 $[M+H]^+$.

12.1. ^{1}H NMR spectrum of compound 4N

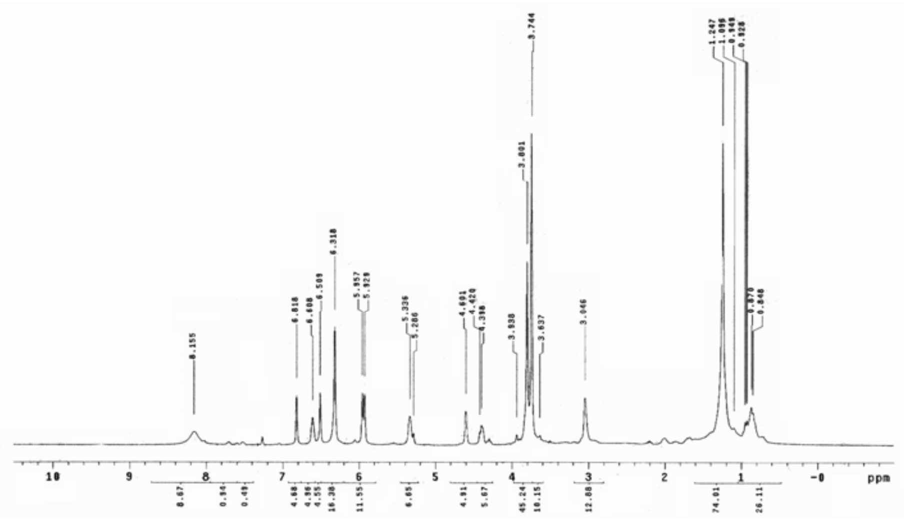

12.2. ^{13}C NMR spectrum of compound 4N

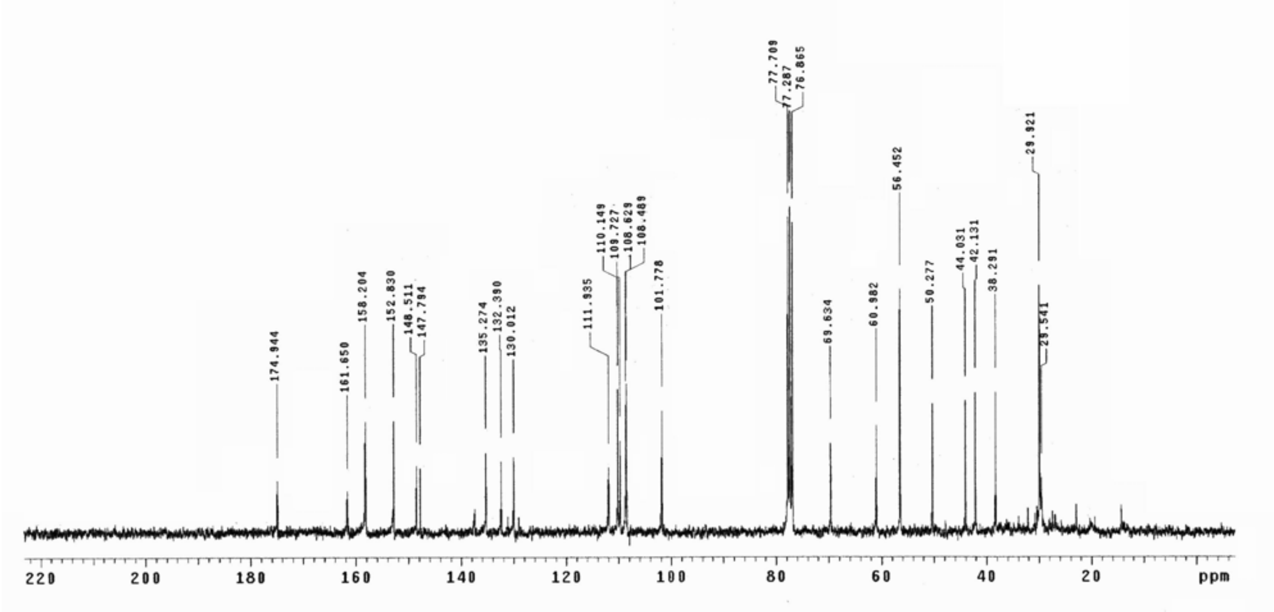

12.3. ^{1}H-^{1}H COSY diagram of compound 4N

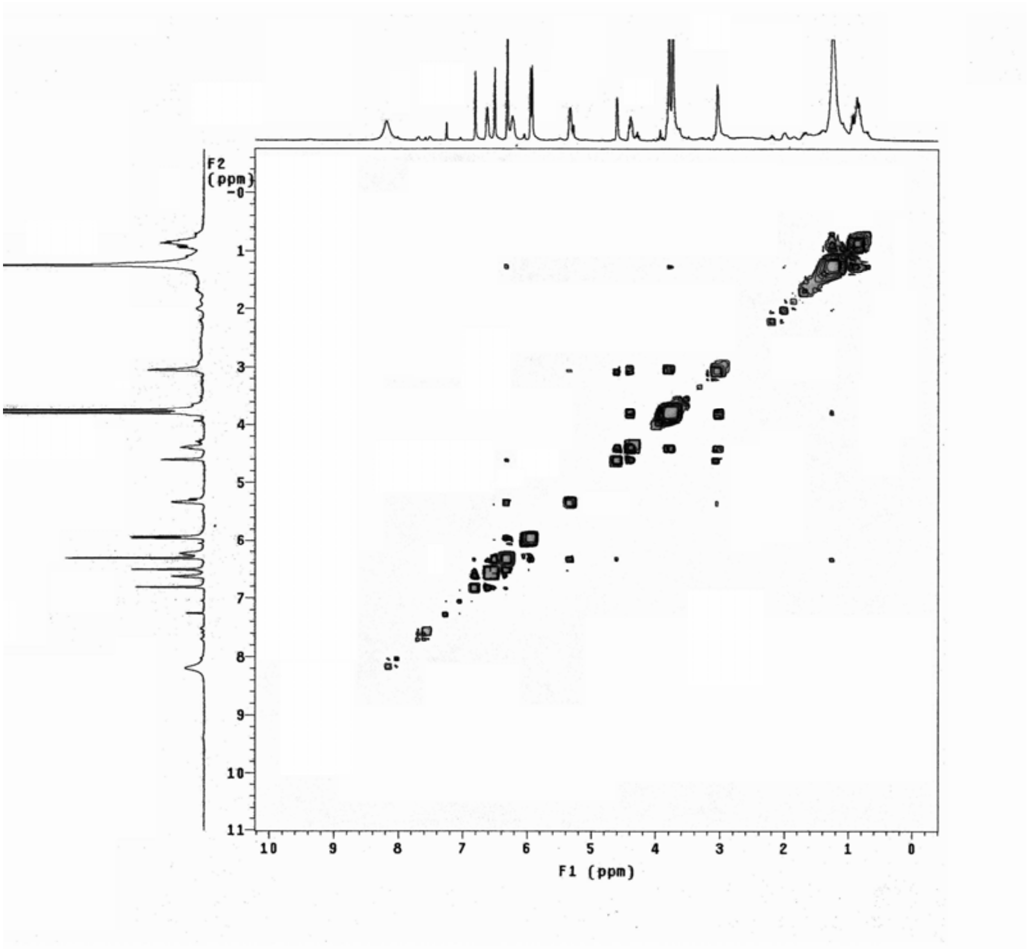

12.4. HMBC diagram of compound 4N

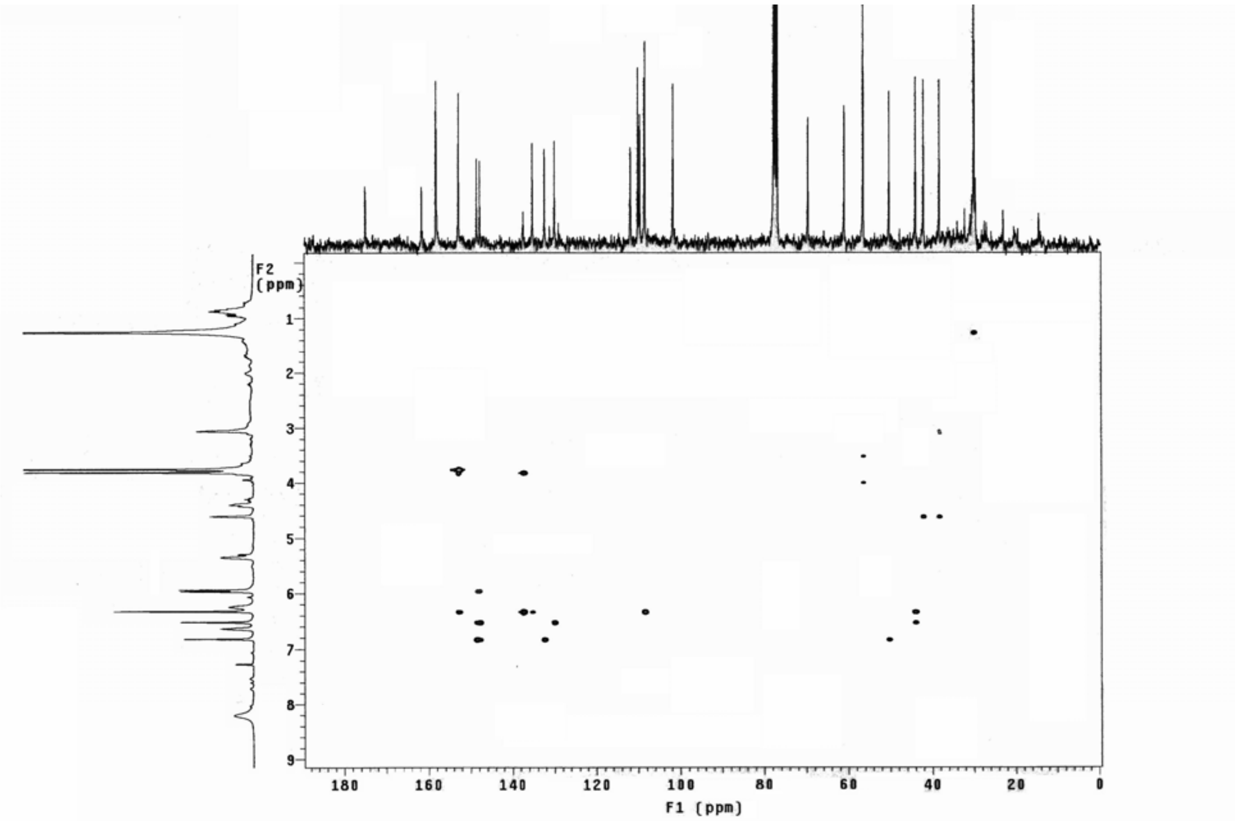

12.5. HSQC diagram of compound 4N

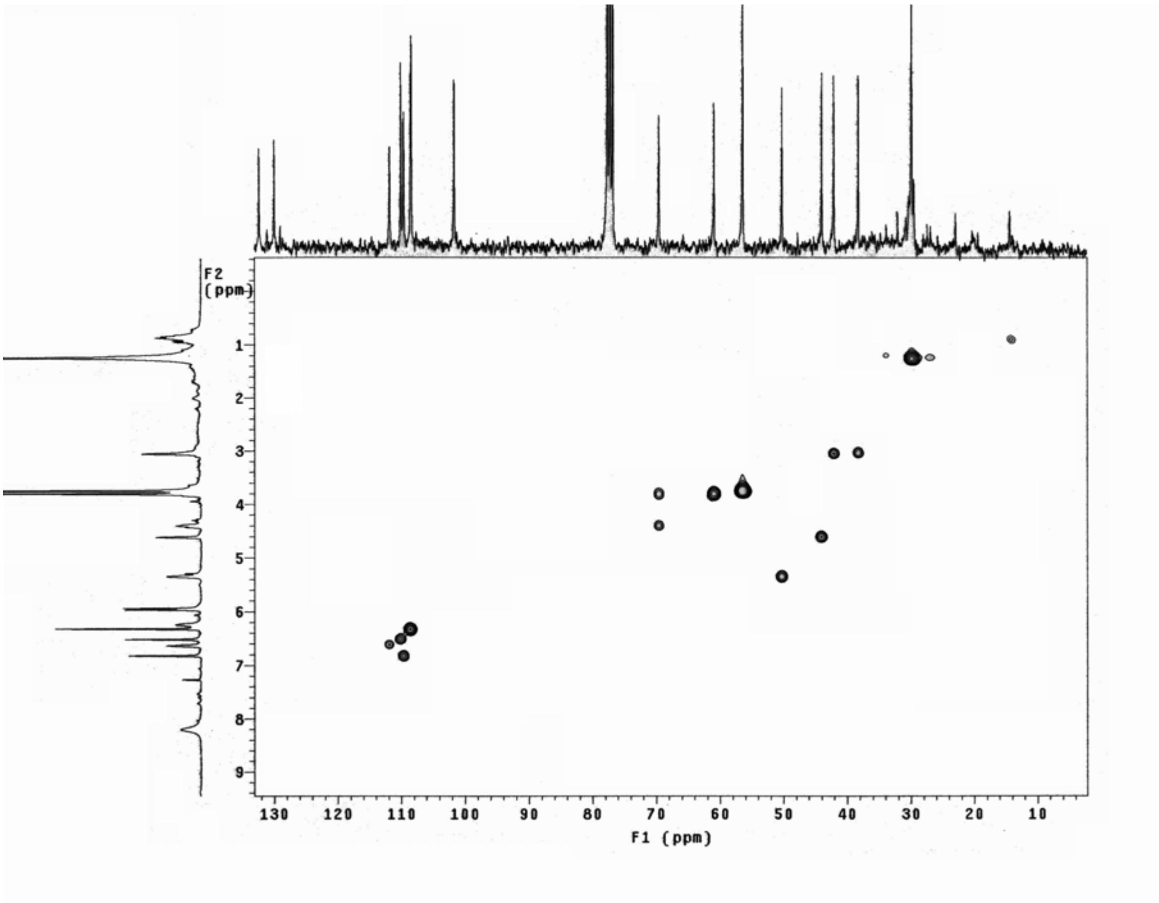

12.6. MS spectrum of compound 4N

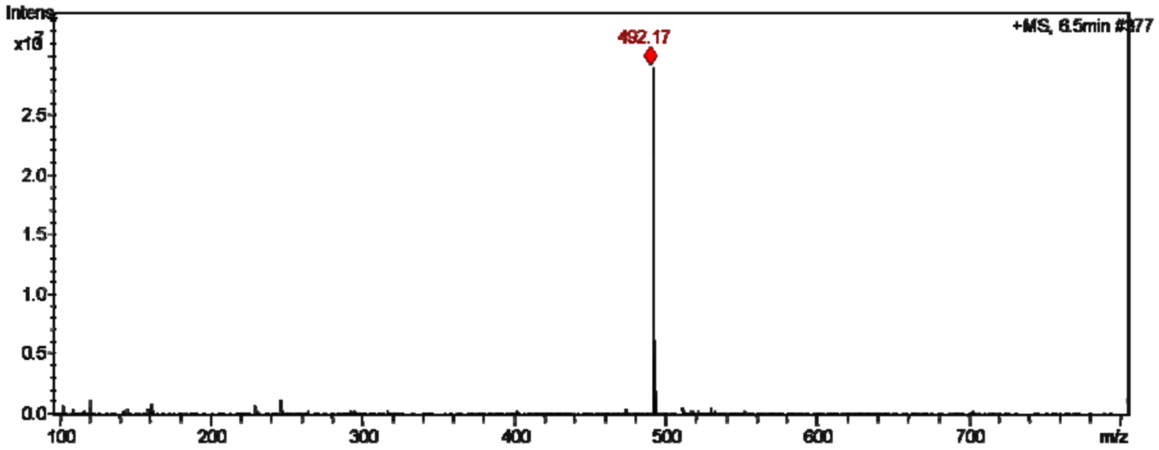

4β-N-(pyrimidine-2)-4-deoxy-podophyllotoxin (4N)

^{1}H NMR (300 MHz, CDCl$_3$, δ): 8.155 (s, 2H), 6.818 (s, 1H), 6.871 (s, 1H), 6.608 (s, 1H), 6.509 (s, 1H), 6.318 (s, 2H), 5.957 (d, *J* = 8.4 Hz, 2H), 5.336 (s, 1H), 4.601 (s, 1H), 4.398 (t, *J* = 0.6 Hz, 1H), 3.801 (s, 3H), 3.744 (s, 6H), 3.046 (s, 3H); ^{13}C NMR(75 MHz, CDCl$_3$, δ): 174.944 , 161.650, 158.204 (2C), 152.830 (2C), 148.511, 147.794, 135.274, 132.390, 130.012, 111.935, 110.149, 109.727, 108.629, 108.489 (2C), 101.778, 69.634, 60.982, 56.452 (2C), 50.277, 44.031, 42.131, 38.291.

ESI-MS: calc'd for $C_{26}H_{25}N_3O_7$ [M+H]$^+$: 492.17, found 492.17 [M+H]$^+$.

13.1. ^{1}H NMR spectrum of compound 5N

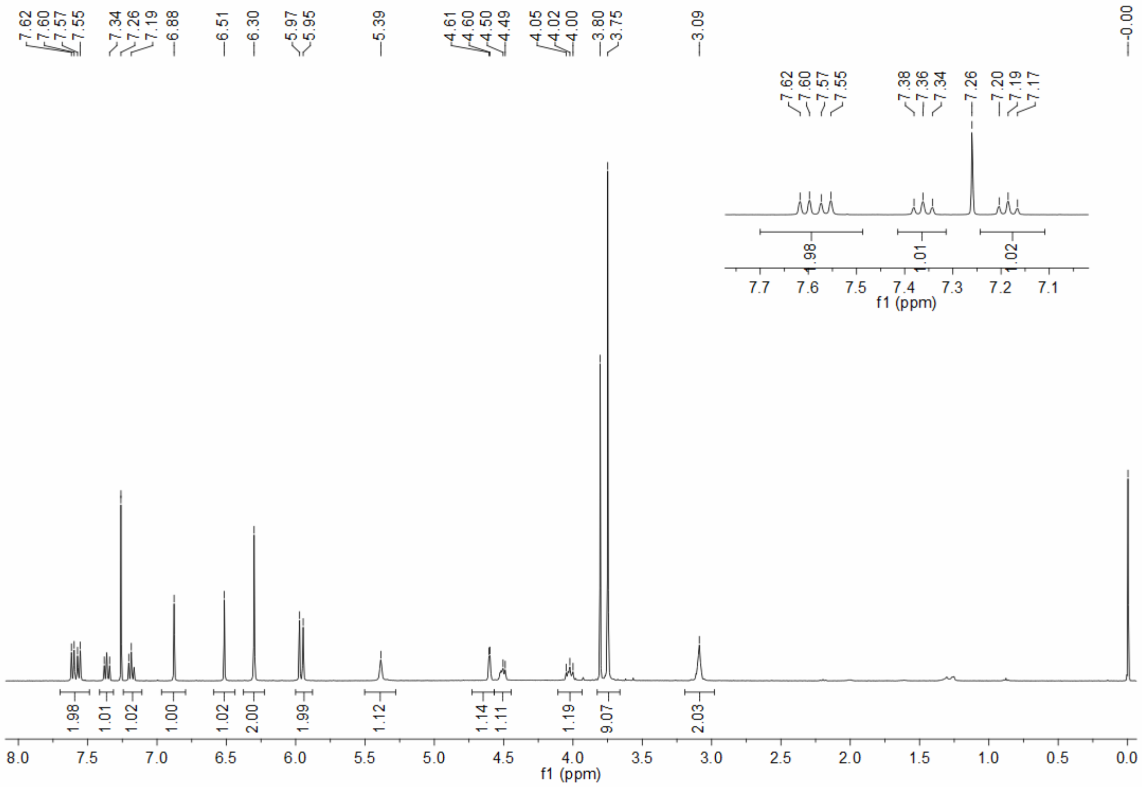

13.3. ^{1}H-^{1}H COSY diagram of compound 5N

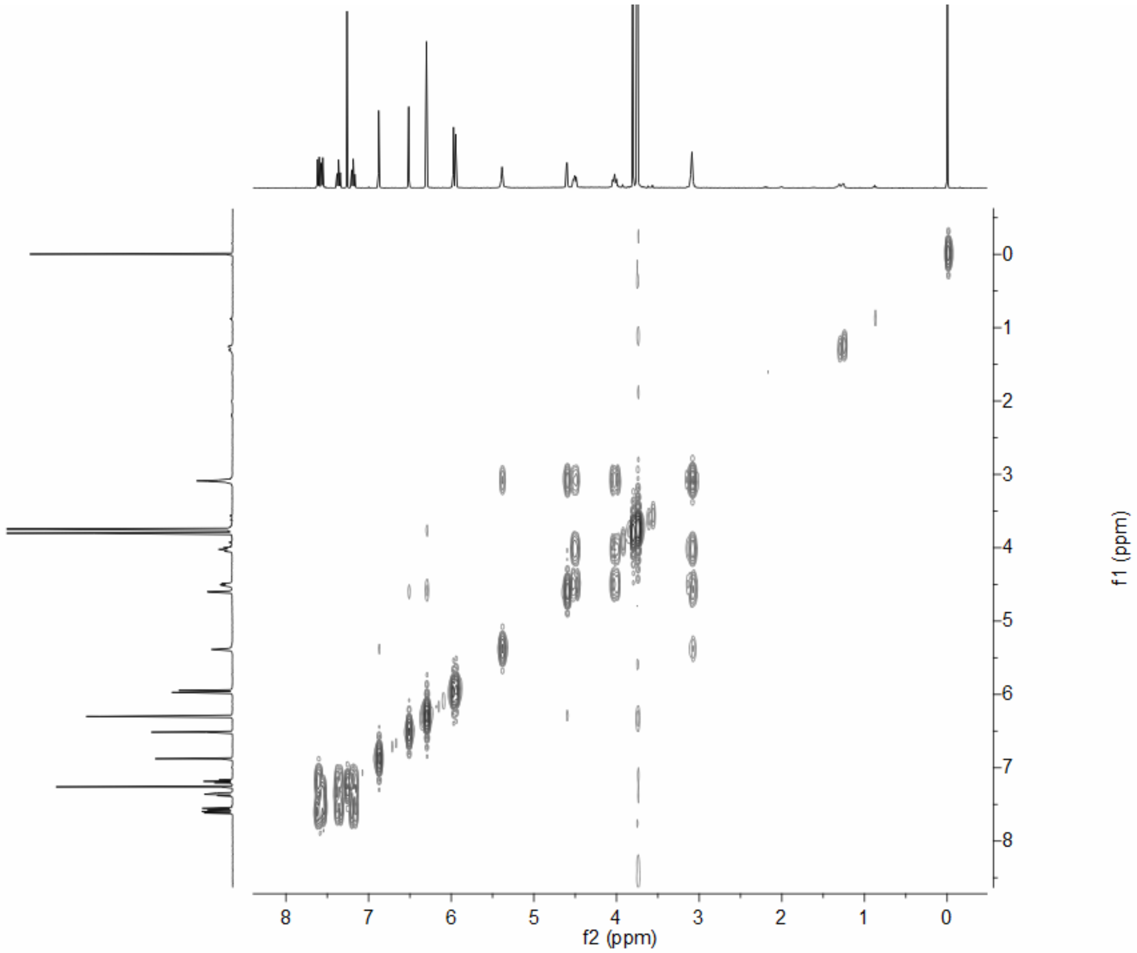

13.4. HMBC diagram of compound 5N

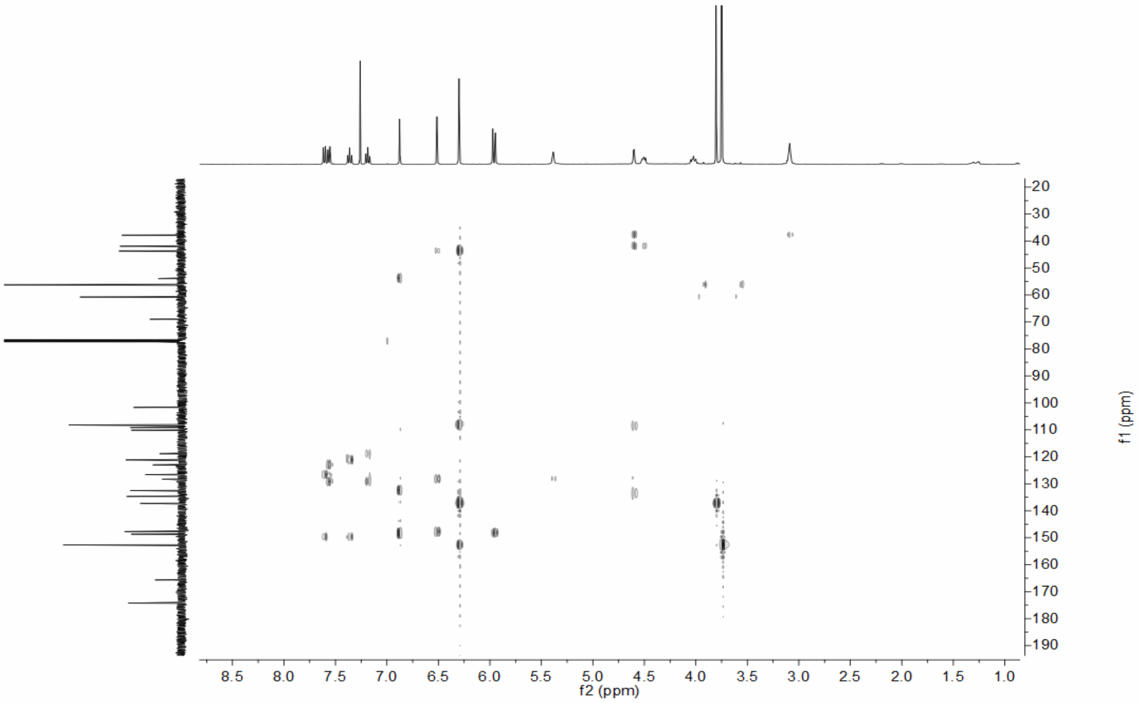

13.5. HSQC diagram of compound 5N

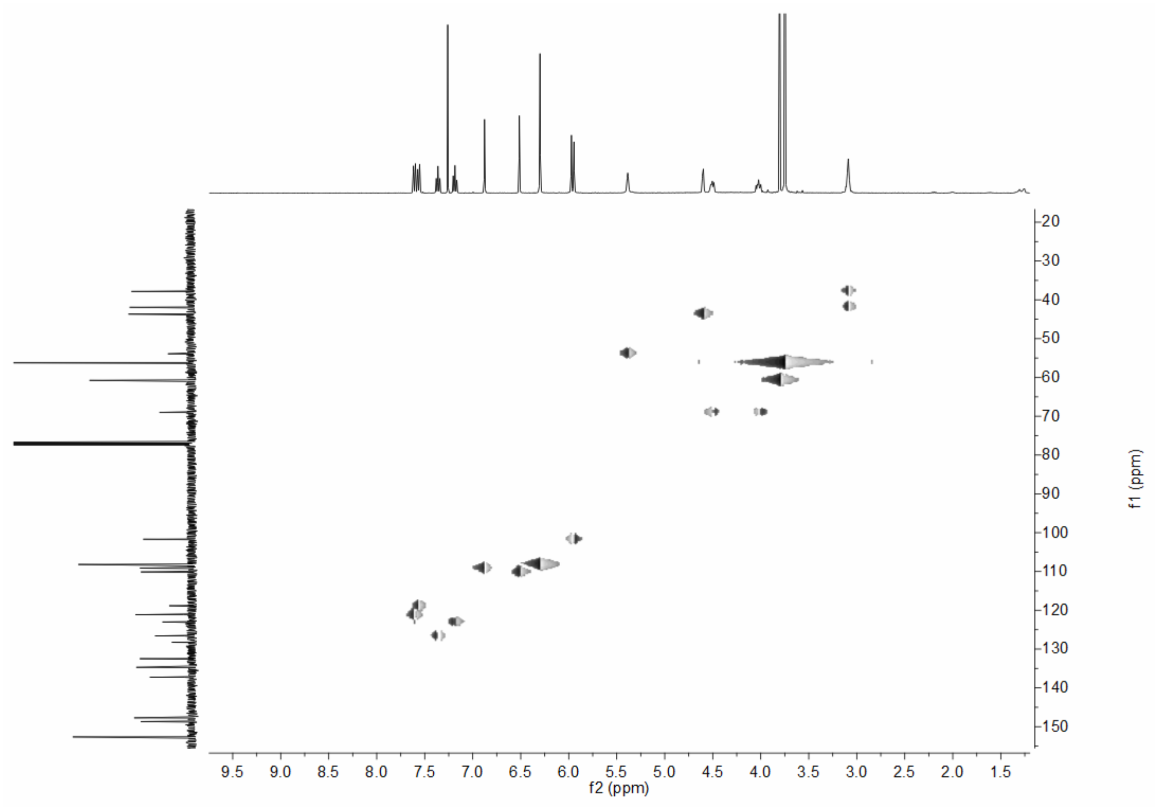

13.6. MS spectrum of compound 5N

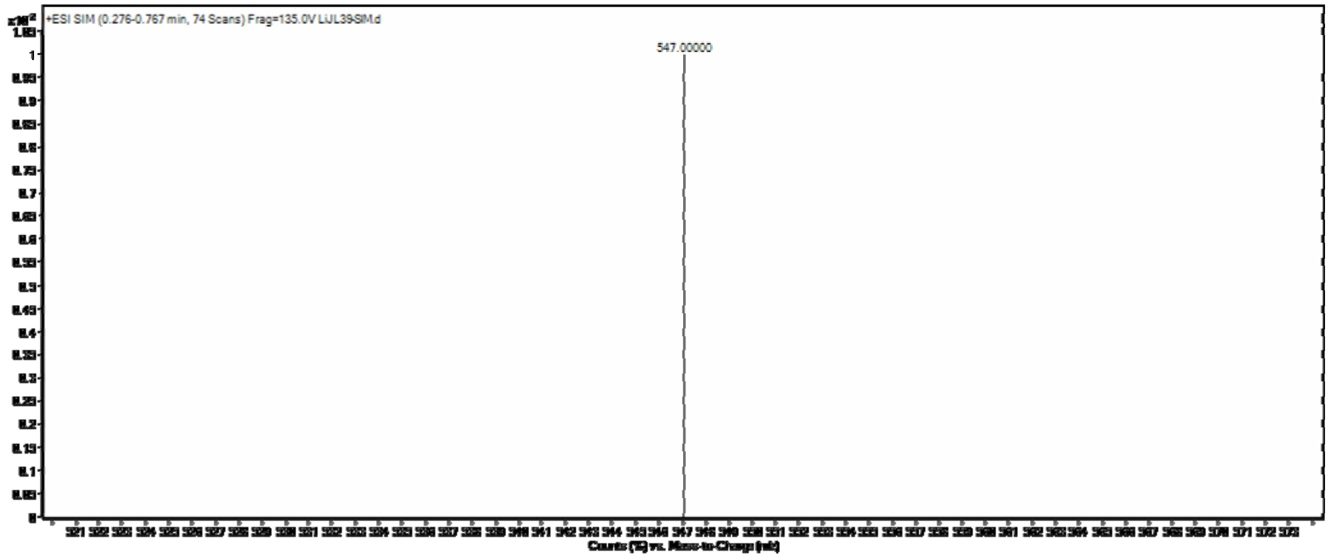

4β-N-(pyrimidine-2)-4-deoxy-podophyllotoxin (5N)

^{1}H NMR (400 MHz, $CDCl_3$, δ): 7.62 (d, *J* = 8.0 Hz, 1H), 7.57 (d, *J* = 8.0 Hz, 1H), 7.36 (t, *J* = 8.0 Hz, 1H), 7.19 (t, *J* = 4.0 Hz, 1H), 6.88 (s, 1H), 6.51 (s, 1H), 6.30 (s, 2H), 5.97 (d, *J* = 8.0 Hz, 2H), 5.39 (s, 1H), 4.61 (d, *J* = 4.0 Hz, 1H), 4.50 (t, *J* = 4.0 Hz, 1H), 4.02 (t, *J* = 8.0 Hz, H), 3.80 (s, 3H), 3.75 (s, 6H), 3.11-3.07 (m, 2H); ^{13}C NMR(101 MHz, $CDCl_3$, δ): 174.25, 165.66, 152.63 (2C), 148.68, 147.72 (2C), 137.27, 134.70, 132.48, 128.27, 126.61, 123.00, 121.15, 118.83 (2C), 110.12, 109.16, 108.19 (2C), 101.69, 69.00, 60.75, 56.24 (2C), 53.91, 43.69, 41.92, 37.84.

ESI-MS: calc'd for $C_{29}H_{26}N_2O_7S$ $[M+H]^+$: 547.14, found 547.00 $[M+H]^+$.

14.1. ^{1}H NMR spectrum of compound 6N

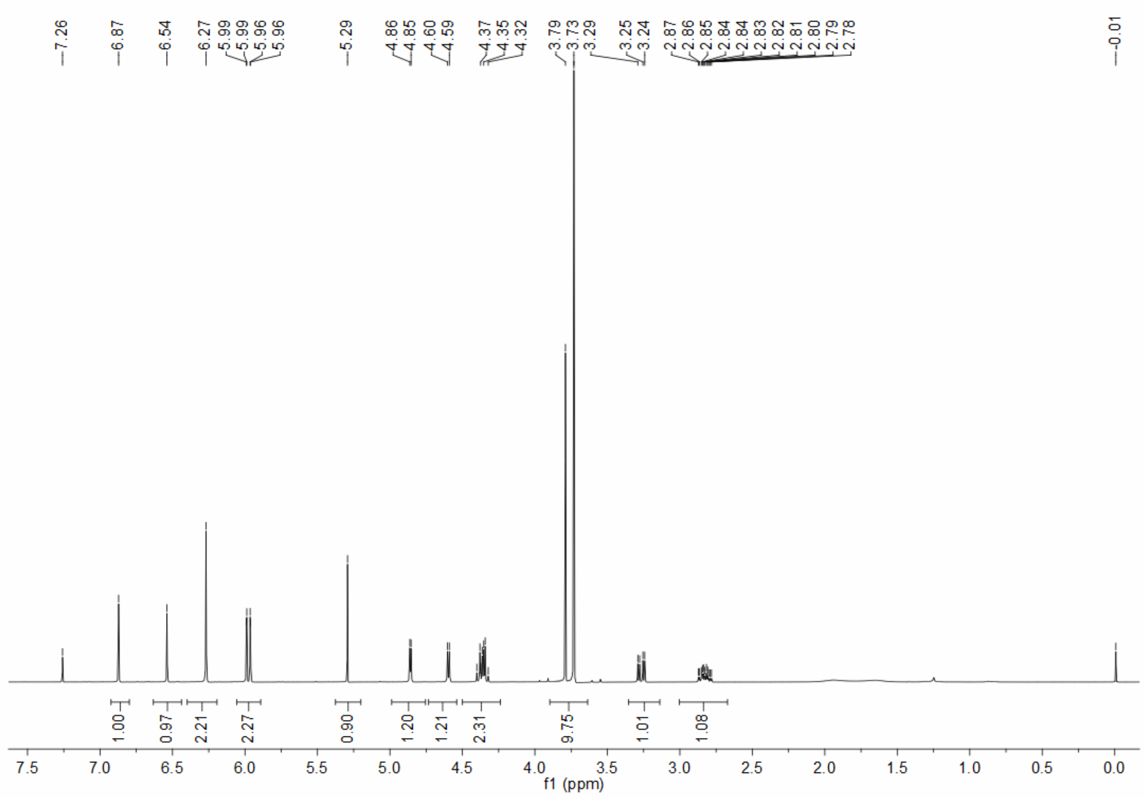

14.2. ^{13}C NMR spectrum of compound 6N

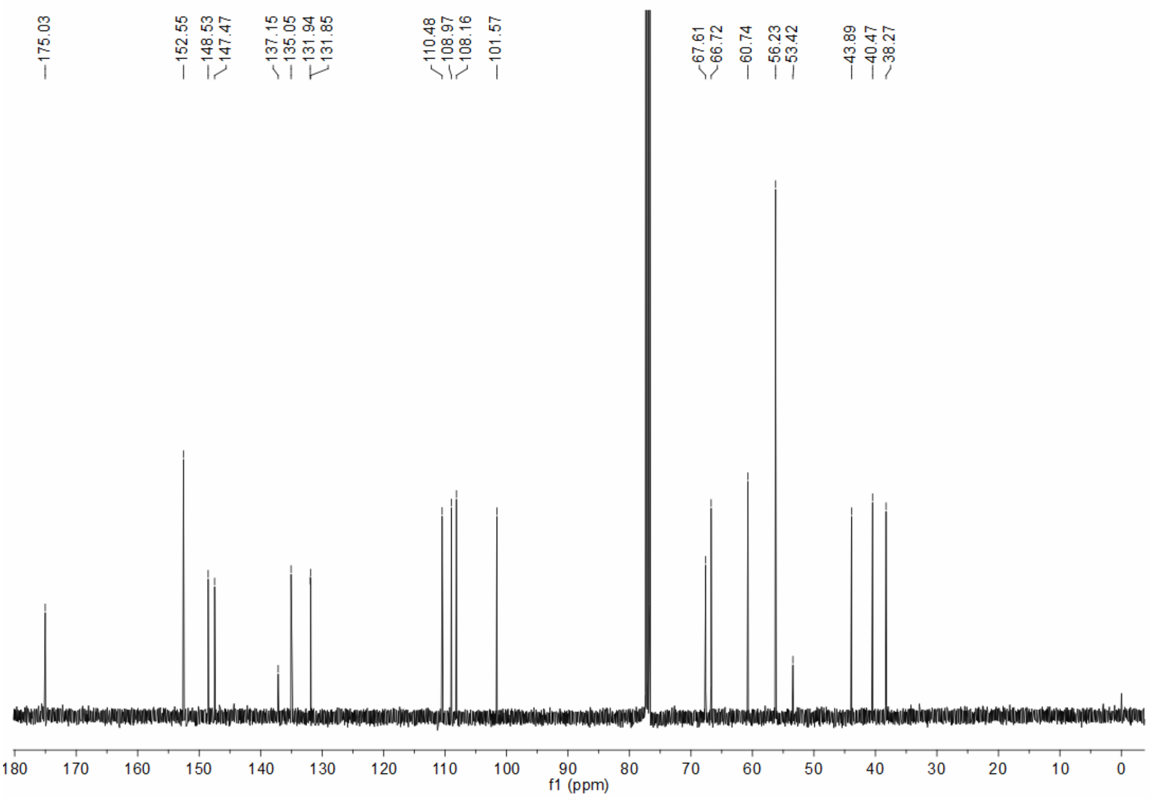

14.3. ^{1}H-^{1}H COSY diagram of compound 6N

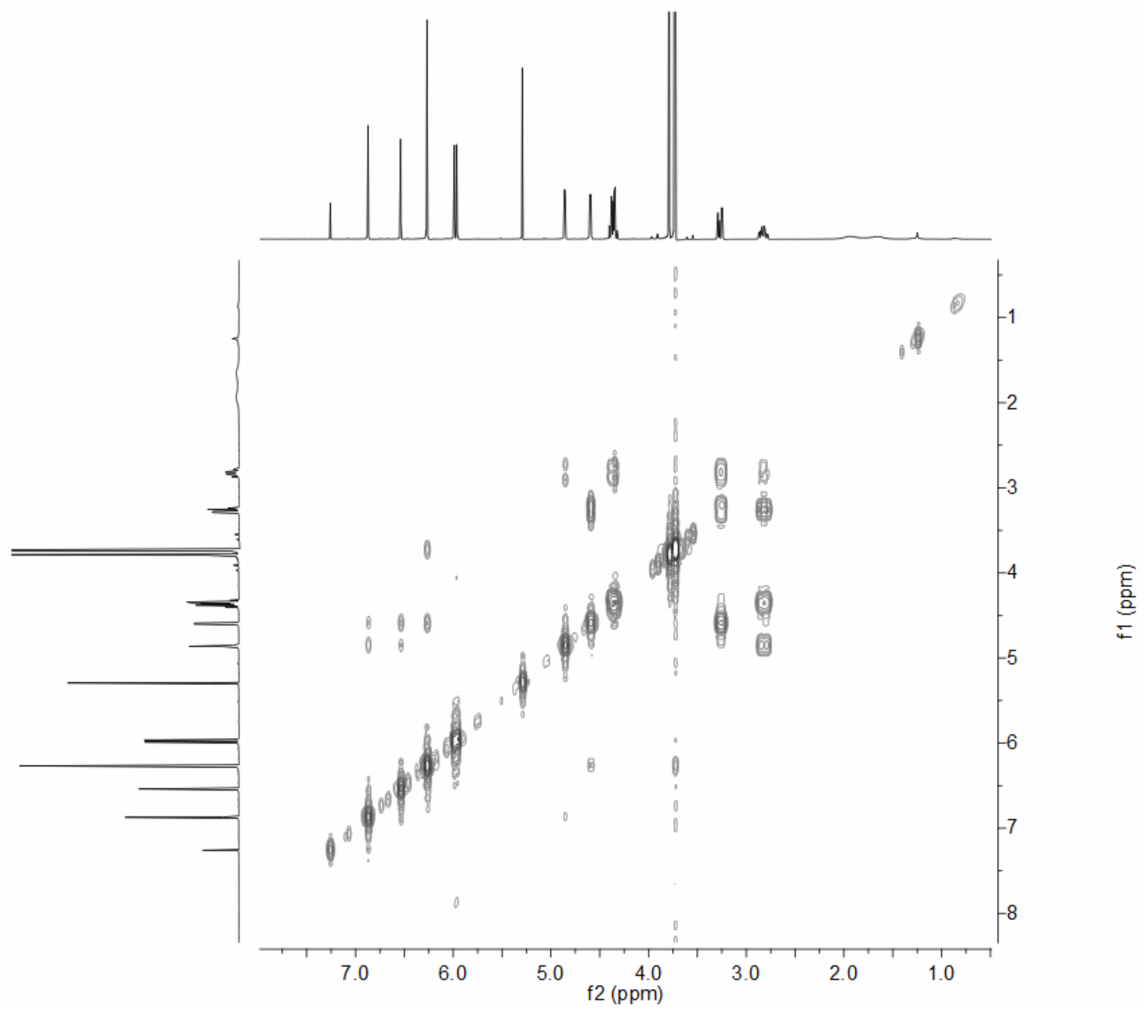

14.3. HMBC diagram of compound 6N

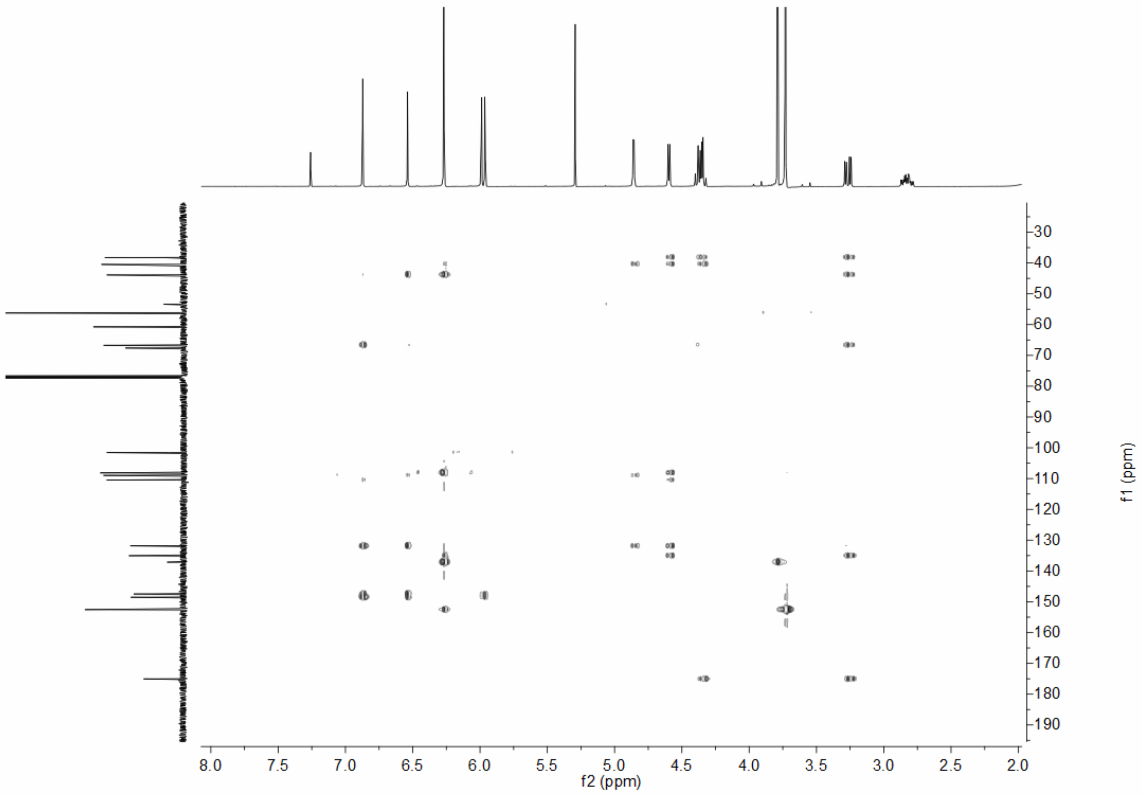

14.5. HSQC diagram of compound 6N

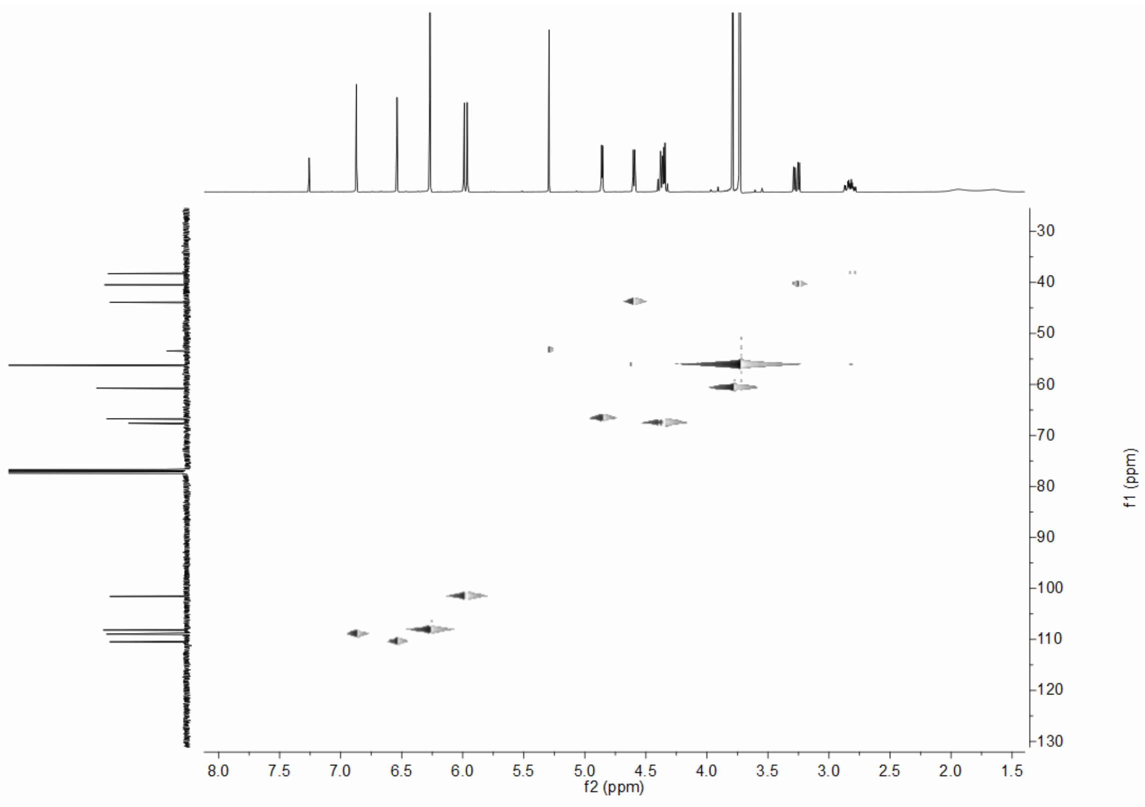

14.6 MS spectrum of compound 6N

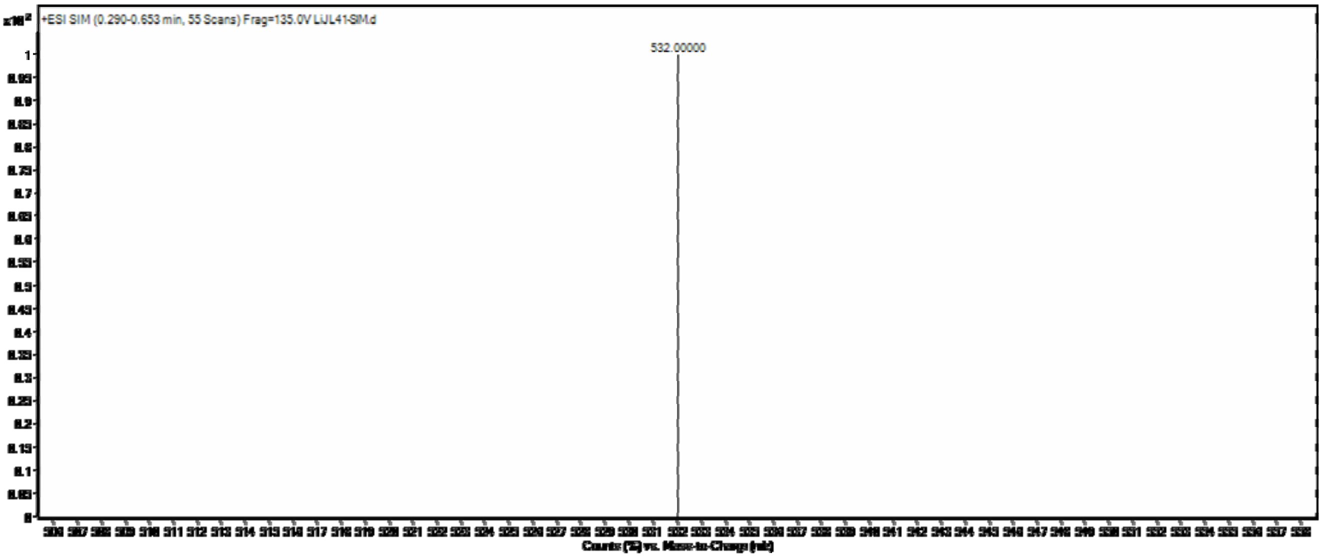

4β-N-(pyrimidine-2)-4-deoxy-podophyllotoxin (6N)

^{1}H NMR (400 MHz, $CDCl_3$, δ): 6.87 (s, 1H), 6.54 (s, 1H), 6.27 (s, 2H), 5.99 (d, *J* = 12.0 Hz, 2H), 5.29 (s, 1H), 4.86 (d, *J* = 4.0 Hz, 1H), 4.60 (d, *J* = 4.0 Hz, 1H), 4.40-4.32 (m, 2H), 3.79 (s, 3H), 3.73 (s, 6H), 3.29-3.24 (m, 1H), 2.87-2.78 (m, 1H); ^{13}C NMR (101 MHz, $CDCl_3$, δ): 175.03 (2C), 152.55 (2C), 148.53 (2C), 147.47, 137.15, 135.05 (2C), 131.94, 131.85, 110.48 (2C), 108.97, 108.16 (2C), 101.57, 67.61, 66.72, 60.74, 56.23 (2C), 53.42, 43.89, 40.47, 38.27.

ESI-MS: calc'd for $C_{27}H_{25}N_5O_7$ $[M+H]^+$: 532.18, found 532.00 $[M+H]^+$.

15.1. ^{1}H NMR spectrum of compound 1'N

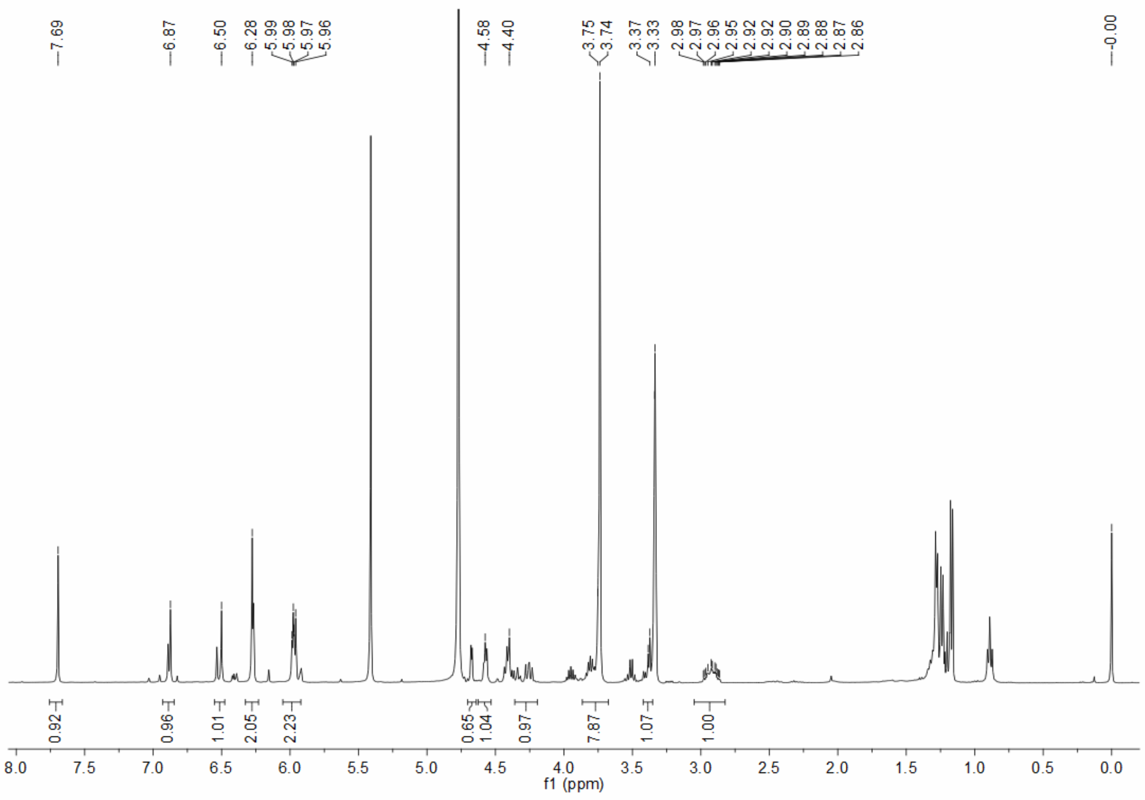

15.2. ^{13}C NMR spectrum of compound 1'N

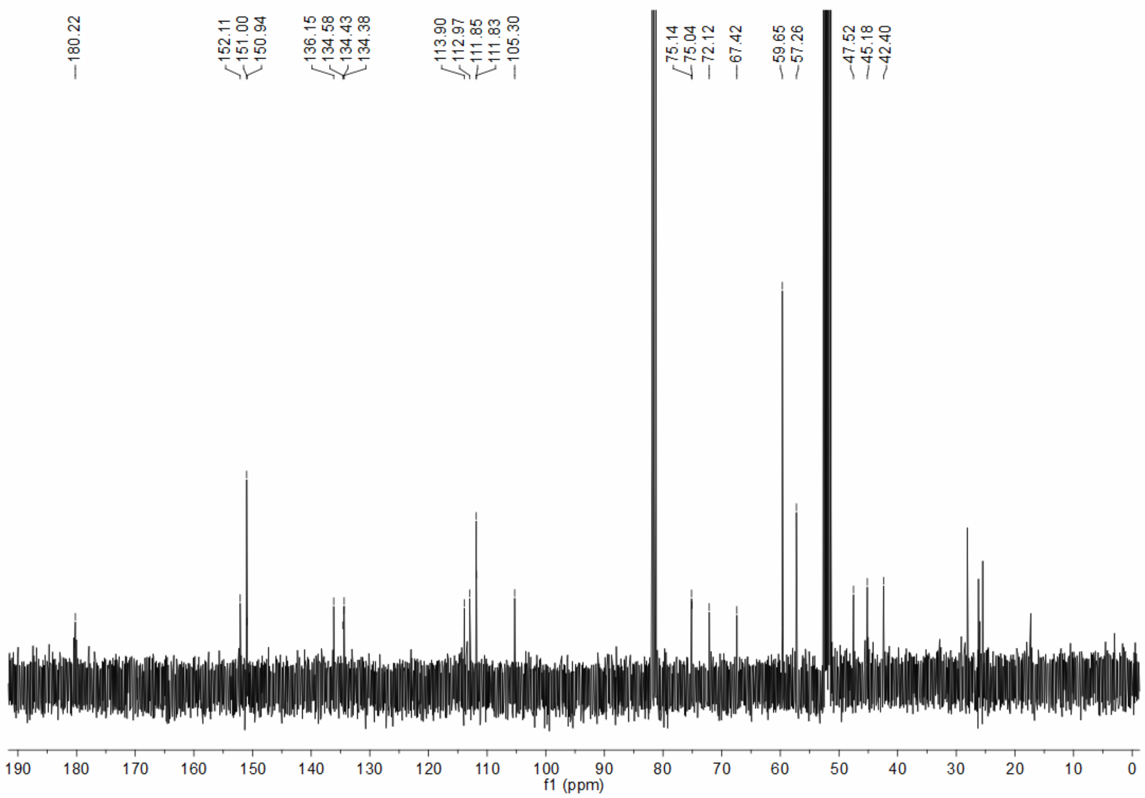

15.3.

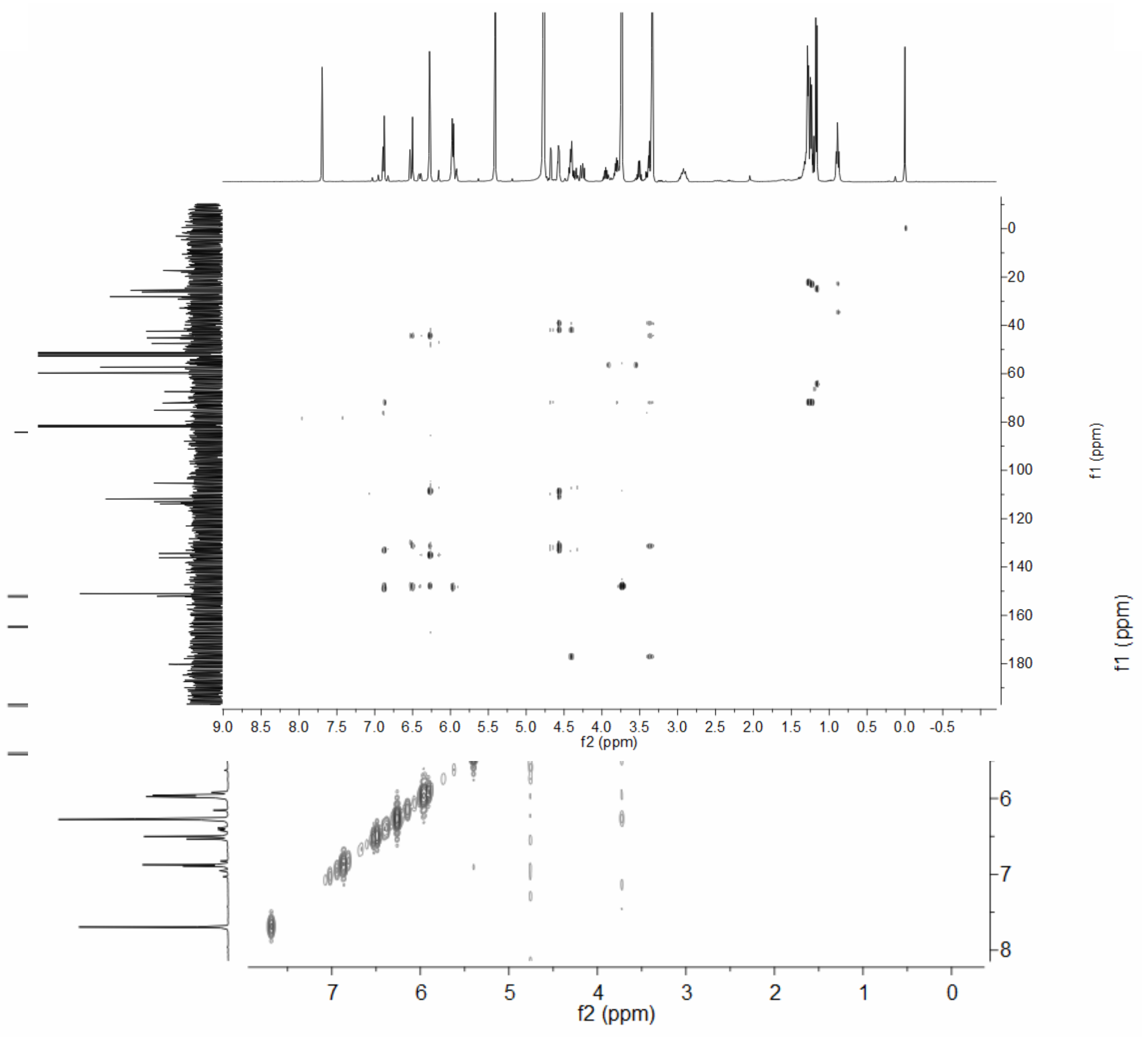

^{1}H-^{1}H COSY diagram of compound 1'N

15.4. HMBC diagram of compound 1'N

15.5. HSQC diagram of compound 1'N

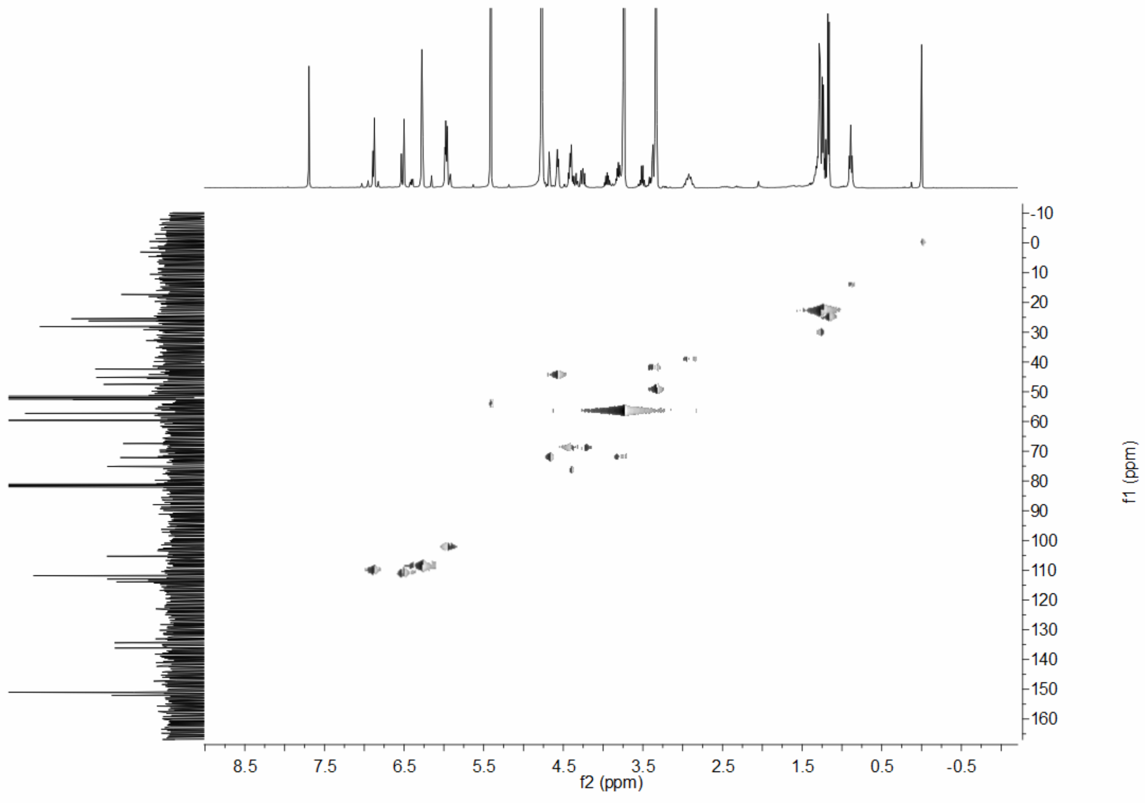

15.6. MS spectrum of compound 1'N

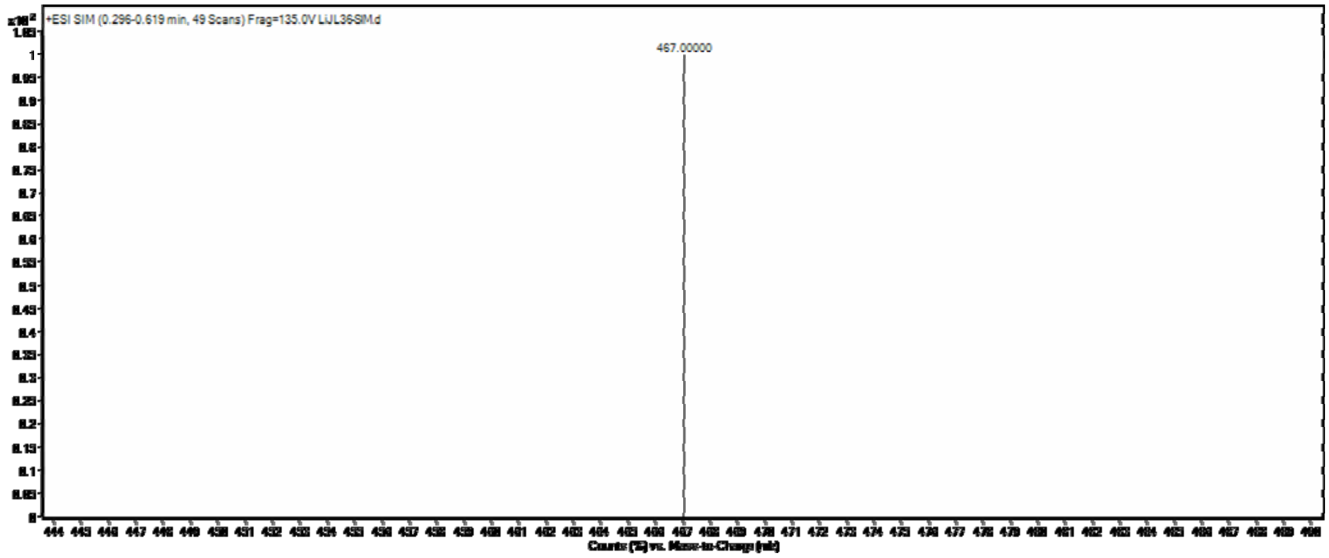

4β-N-(1,2,4-trizole-3)-4-deoxy-4'-demethyl-podophyllotoxin (1'N)

^{1}H NMR(400 MHz, CD_3OD, CD_3Cl): δ 6.87 (s, 1H), 6.50 (s, 1H), 6.28 (s, 2H), 5.98 (d, 2H, *J* = 8.0 Hz), 5.805 (d, 1H, *J* = 1.8 Hz), 5.439 (s, 1H), 4.691 (d, 1H), 4.618 (d, 1H, *J* = 2.4 Hz, 4.522 (t, 1H *J* = 4.5 Hz,), 3.896 (t, 1H, *J* = 4.8 Hz), 3.796 (s, 6H), 3.379 (t, 1H, *J* = 2.1Hz), 3.190 (dd, 1H, *J* = 2.4Hz); ^{13}C NMR (101 MHz, CD_3OD, CD_3Cl): δ 180.22, 152.11, 151.00, 150.94, 136.15, 134.58, 134.43, 134.38, 113.90, 112.97, 111.85, 111.83, 105.30, 75.14, 75.04, 72.12, 67.42, 59.65 (2C), 57.26, 47.52, 45.18, 42.40.

ESI-MS: calc'd for $C_{23}H_{22}N_4O_7$ $[M+H]^+$: 467.15, found 467.00 $[M+H]^+$.

16.1. ^{1}H NMR spectrum of compound 2'N

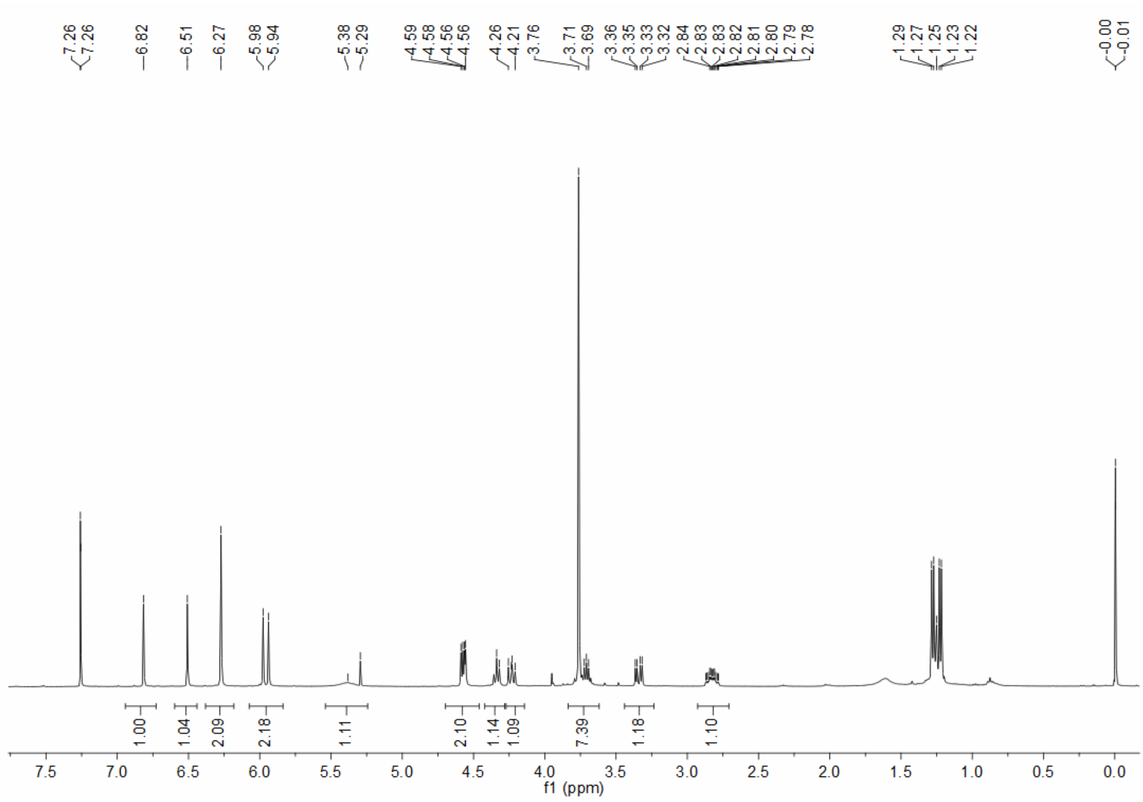

16.2. ^{13}C NMR spectrum of compound 2'N

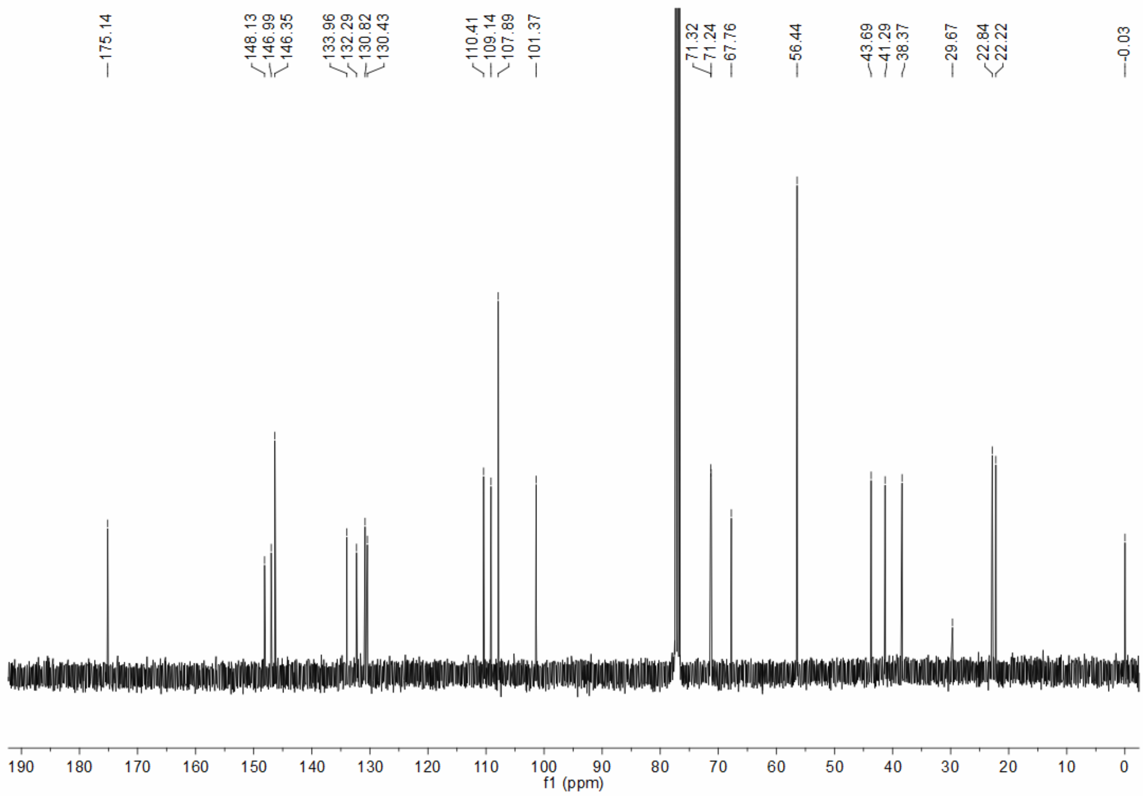

16.3. ^{1}H-^{1}H COSY diagram of compound 2'N

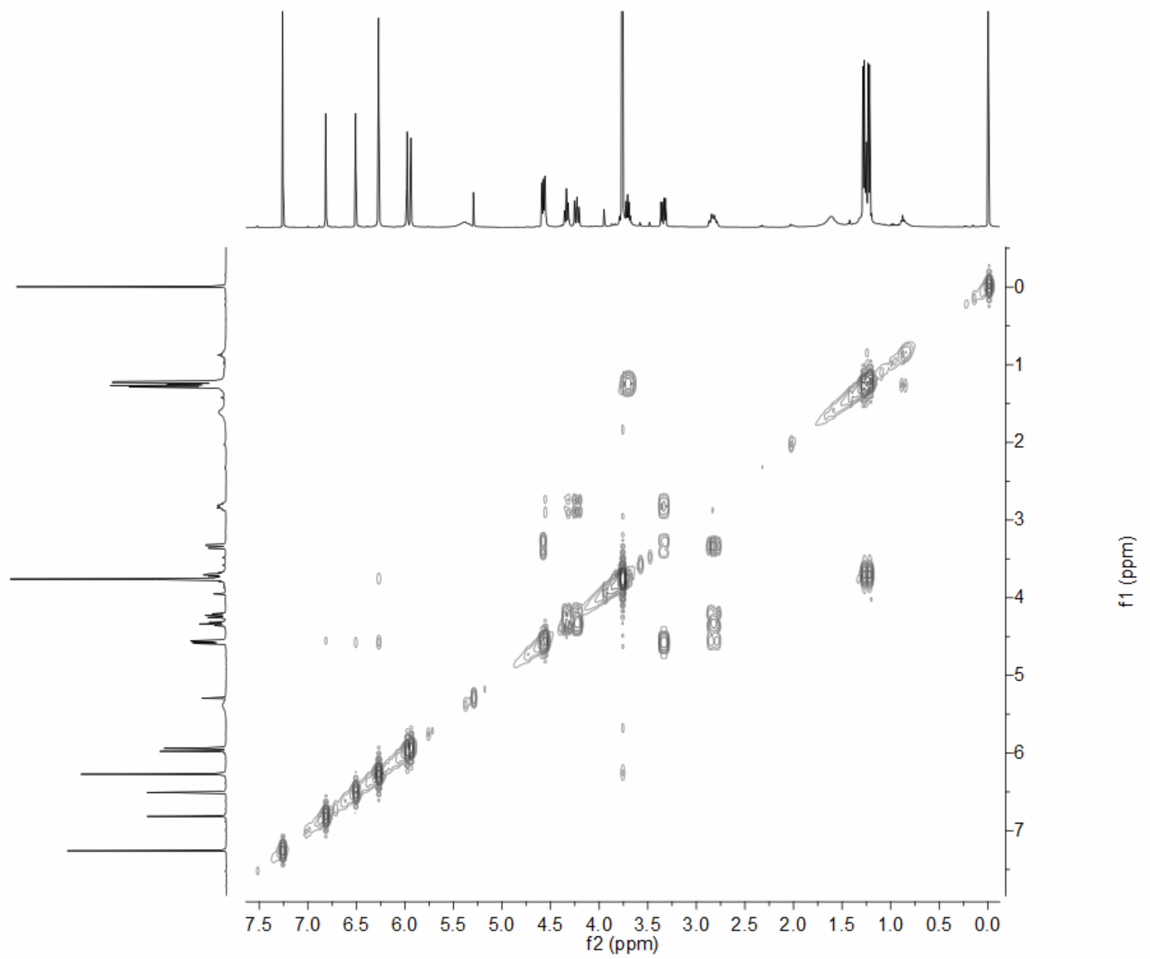

16.4. HMBC diagram of compound 2'N

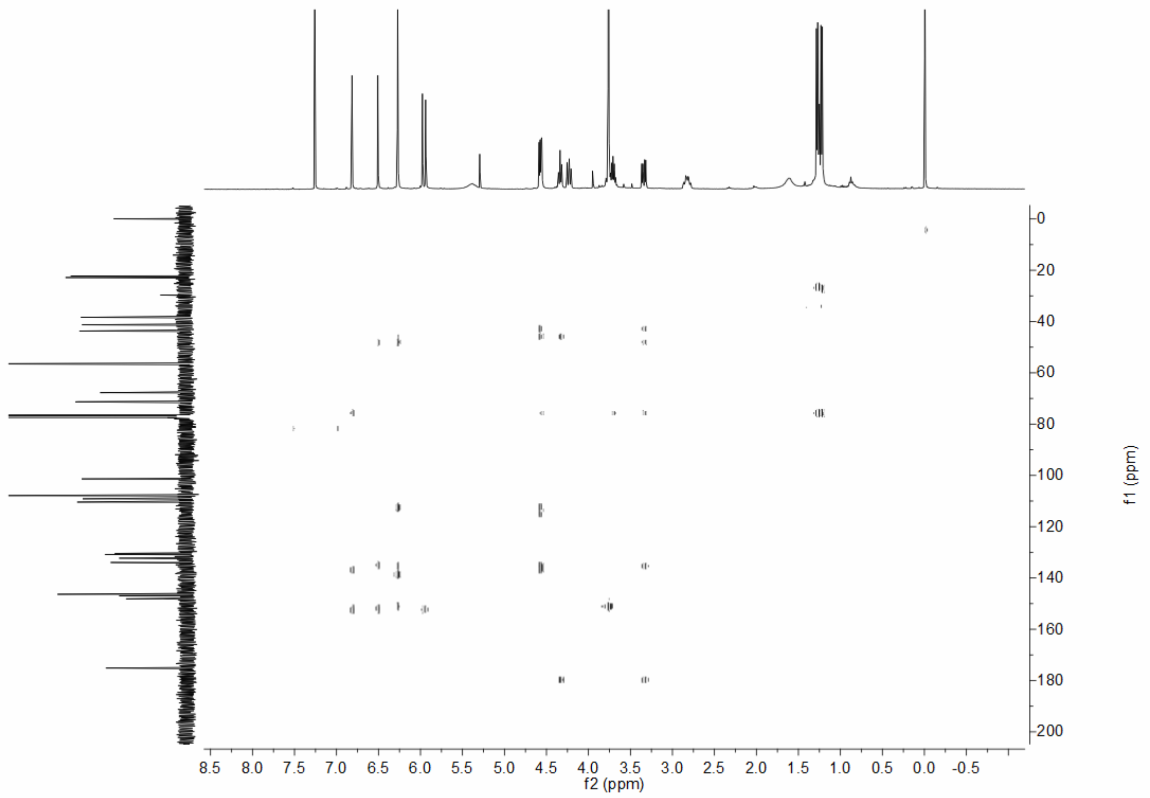

16.5. HSQC diagram of compound 2'N

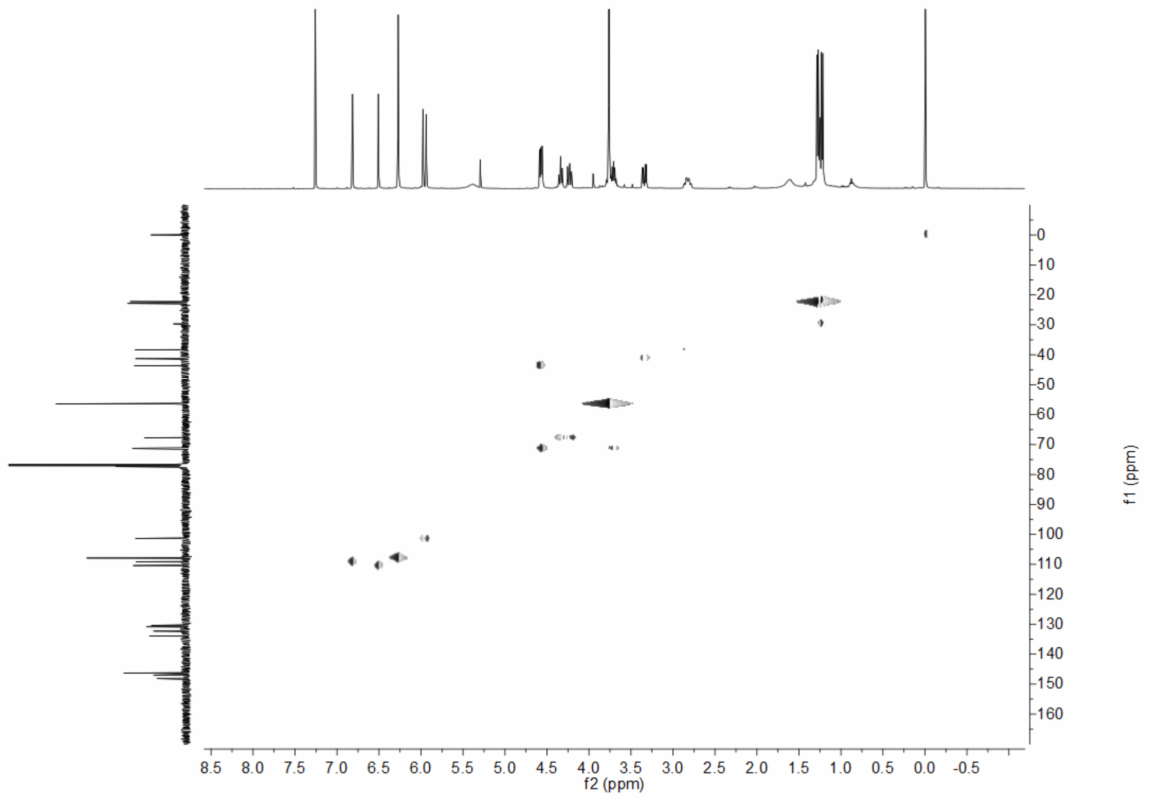

16.6. MS spectrum of compound 2'N

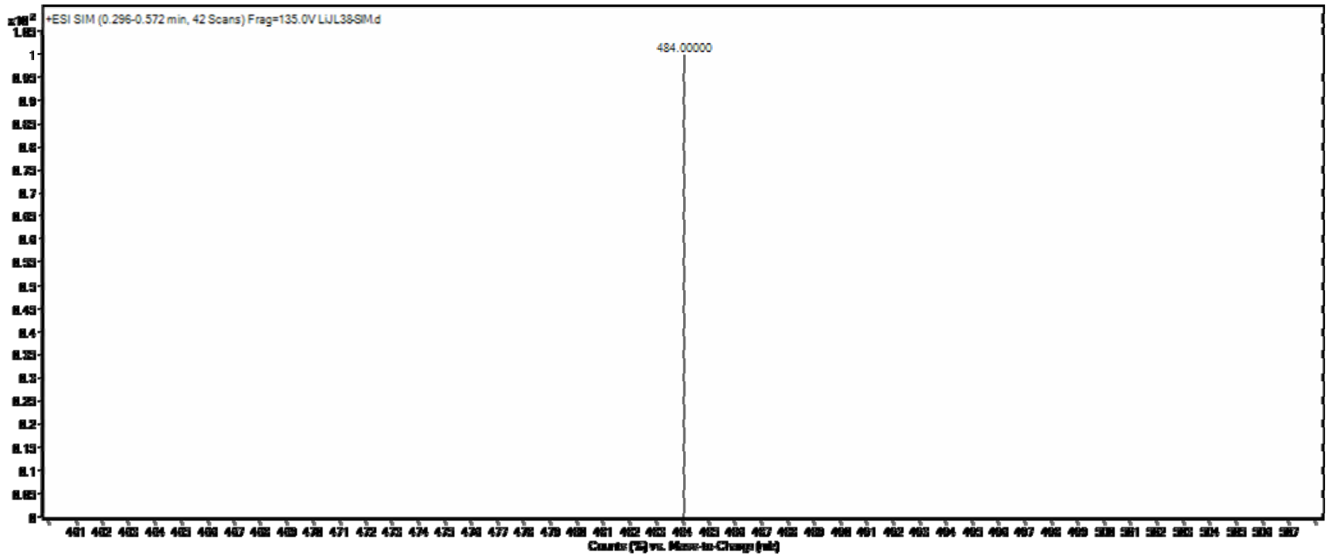

4β-N-(1,3,4-thiodizole-2)-4-deoxy-4'-demethyl-podophyllotoxin (2'N)

^{1}H NMR(400 MHz, $CDCl_3$): δ 6.82 (s, 1H), 6.51 (s, 1H), 6.27 (s, 2H), 5.98 (d, *J* = 16.0 Hz, 2H), 5.38 (s, 1H), 4.59 (dd, *J* = 4.0 Hz, 2H), 4.34 (t, *J* = 4.0 Hz, 1H), 4.23 (t, *J* = 8.0 Hz, 1H), 3.76(s, 6H), 3.71 (t, *J* = 8.0 Hz, 1H), 3.36-3.32 (m, 1H), 2.87-2.78 (m, 1H); ^{13}C NMR (101 MHz, $CDCl_3$): δ 175.14, 148.13, 146.99 (2C), 146.35, 133.96, 132.29, 130.82, 130.43, 110.41, 109.14, 107.89 (2C), 101.37, 71.32, 71.24, 67.76, 56.44 (2C), 43.69, 41.29, 38.37, 29.67.

ESI-MS: calc'd for $C_{23}H_{21}N_3O_7S$ $[M+H]^+$: 484.11, found 484.00 $[M+H]^+$.

17.1. ¹H NMR spectrum of compound 3'N

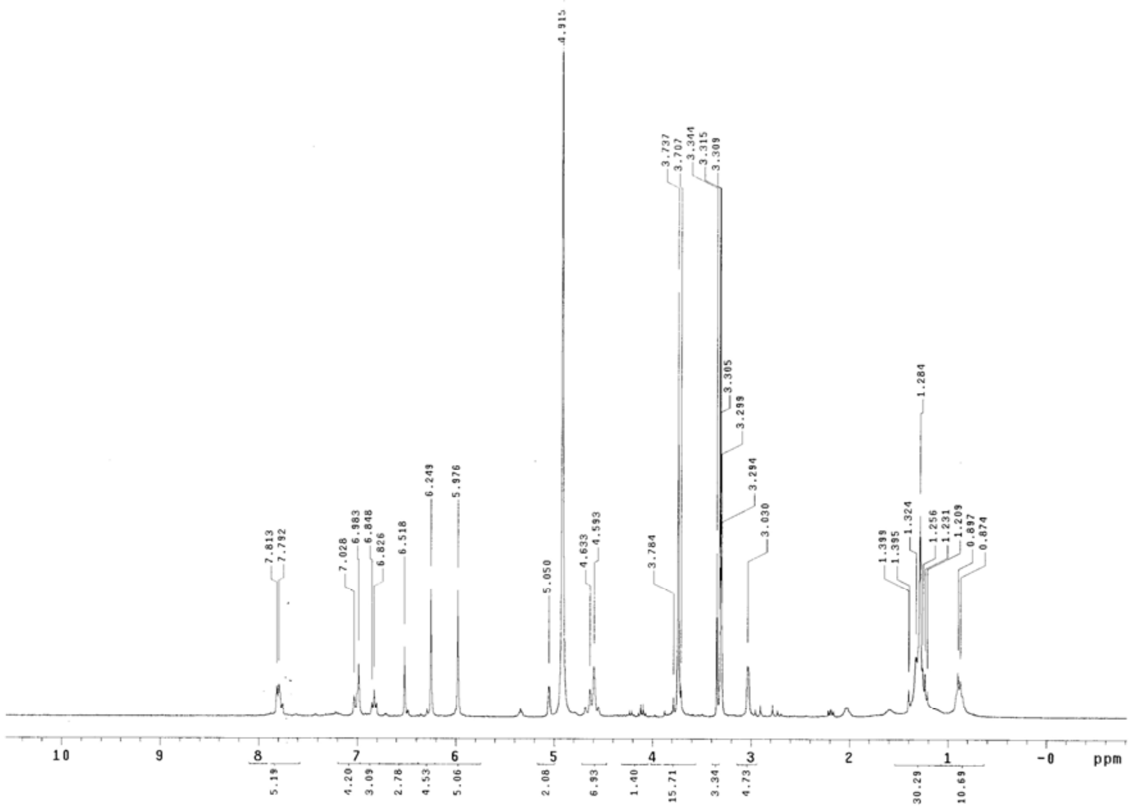

17.2. ¹³C NMR spectrum of compound 3'N

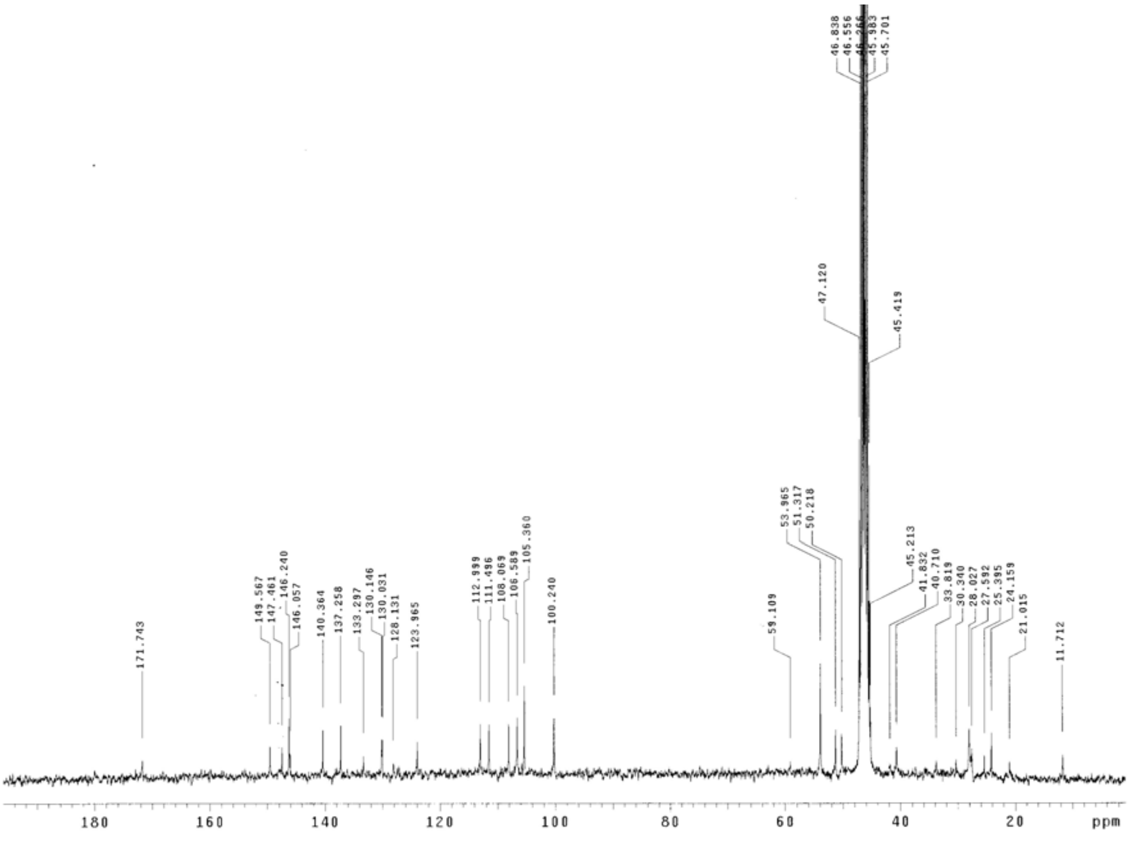

17.3. ^{1}H-^{1}H COSY diagram of compound 3'N

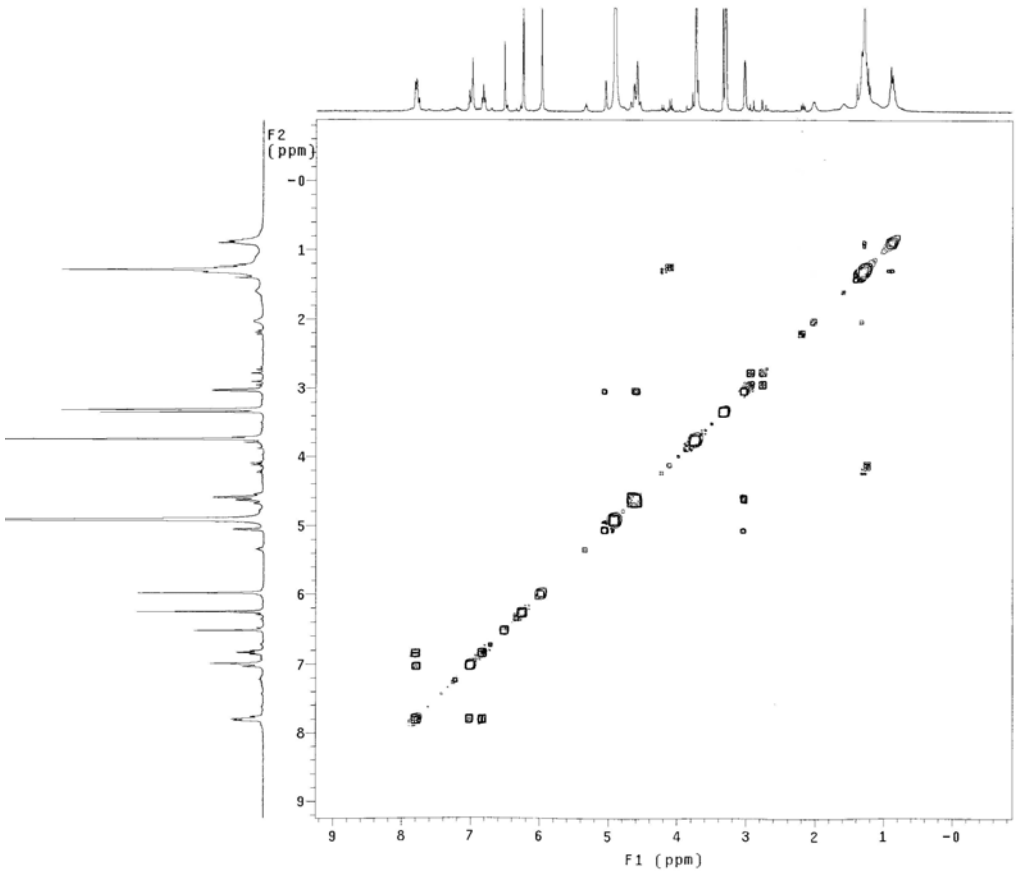

17.4. HMBC diagram of compound 3'N

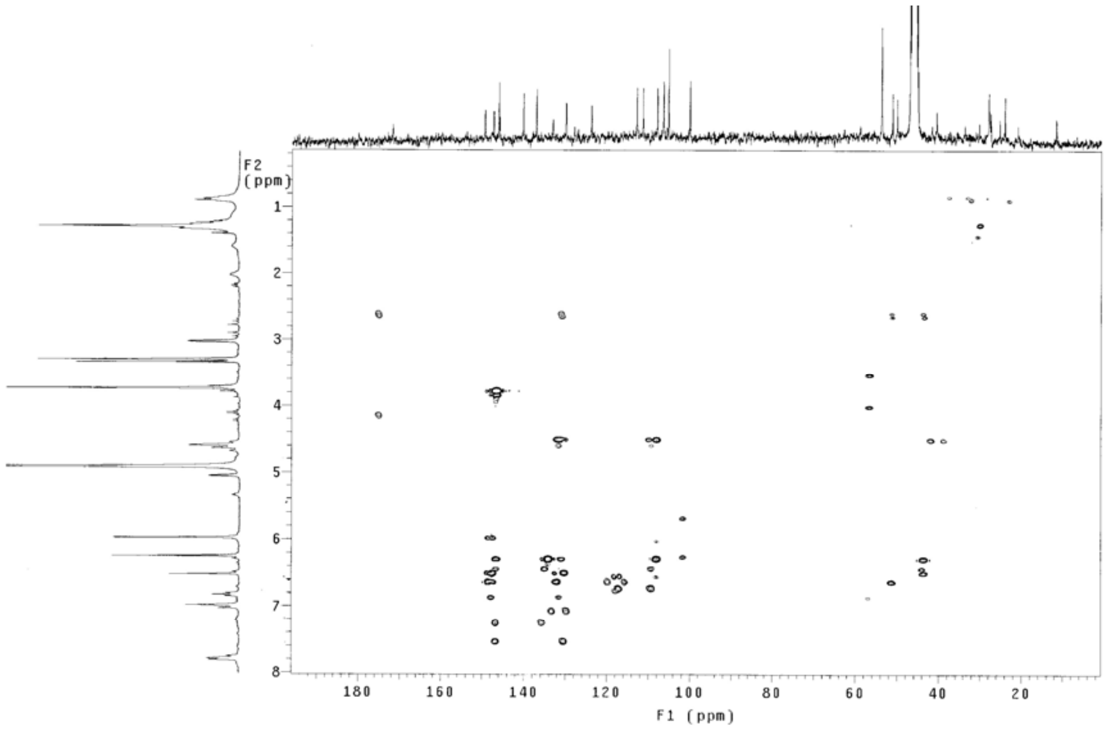

17.5. HSQC diagram of compound 3'N

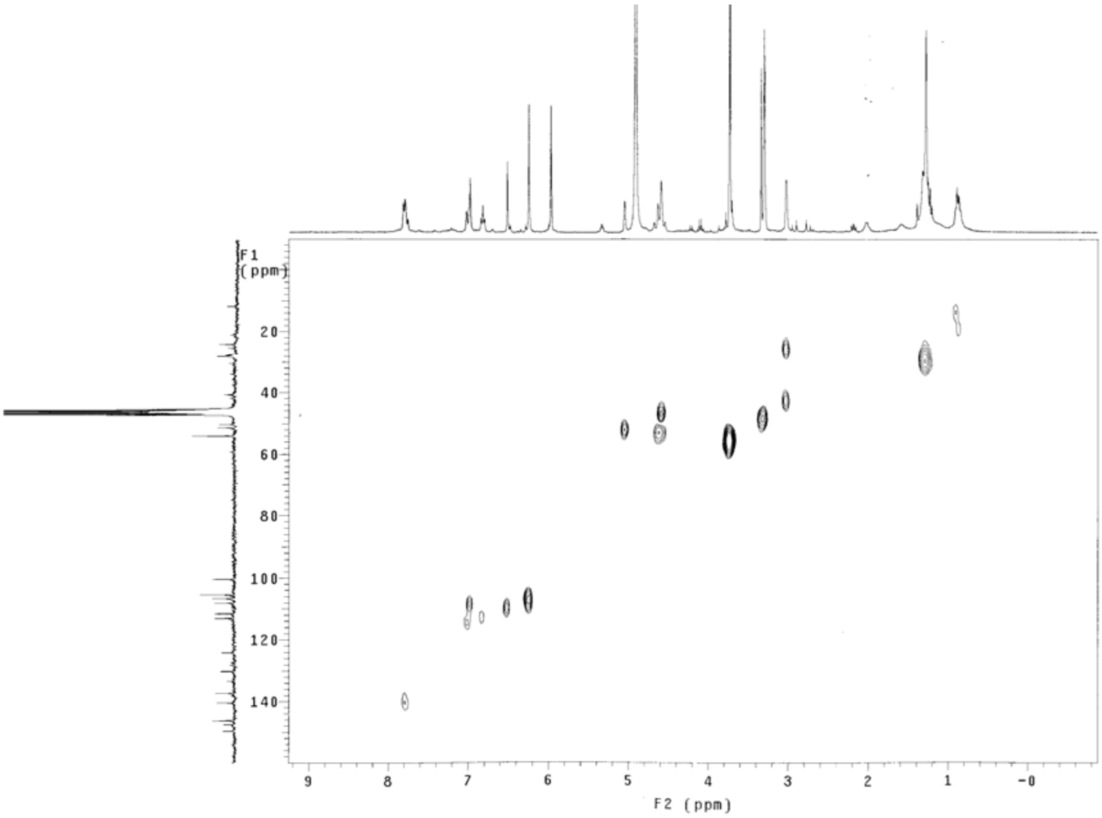

17.6. MS spectrum of compound 3'N

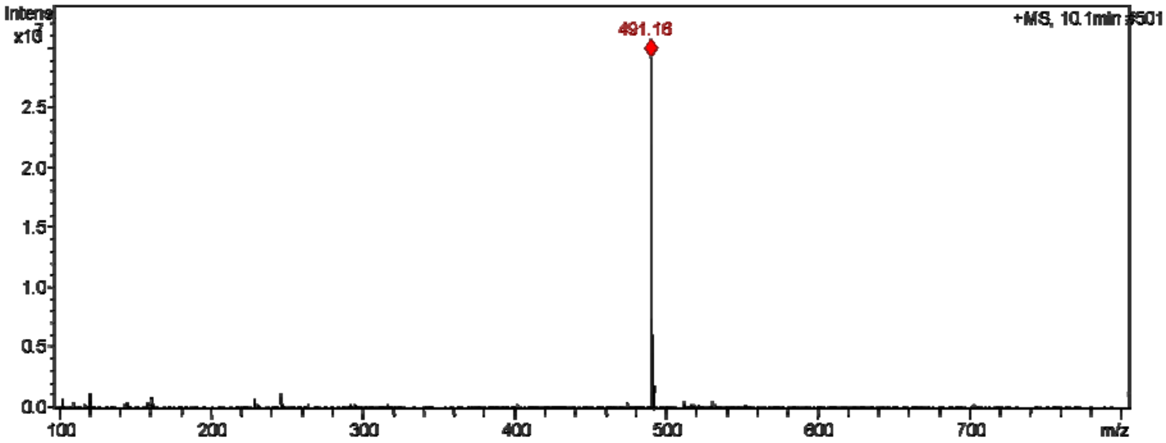

4β-N-(pyridine-2)-4-deoxy-4'-demethyl-podophyllotoxin (3'N)

^{1}H NMR (300 MHz, CD_3OD): δ 7.813 (d, *J* = 6.3 Hz, 2H), 6.983 (s, 2H), 6.826 (t, *J* = 6.6 Hz, 1H), 6.369 (s, 1H), 6.249 (s, 2H), 5.976 (s, 2H), 4.593 (d, *J* = 3.0 Hz, 3H), 3.784 (t, *J* = 9.0 Hz, 1H), 3.737 (s, 6H), 3.030 (s, 2H); ^{13}C NMR (75 MHz, CD_3OD): δ 171.743, 149.567, 147.461, 146.240 (2C), 140.364, 137.258, 133.297, 130.146, 130.031, 128.131, 123.965, 112.999, 111.496, 108.069, 106.589, 105.360 (2C), 100.240, 59.109, 53.965 (2C), 51.317, 50.218, 41.832, 40.710.

ESI-MS: calc'd for $C_{26}H_{24}N_2O_7$ $[M+H]^+$: 491.18, found 491.16 $[M+H]^+$.

18.1.¹H NMR spectrum of compound 4'N

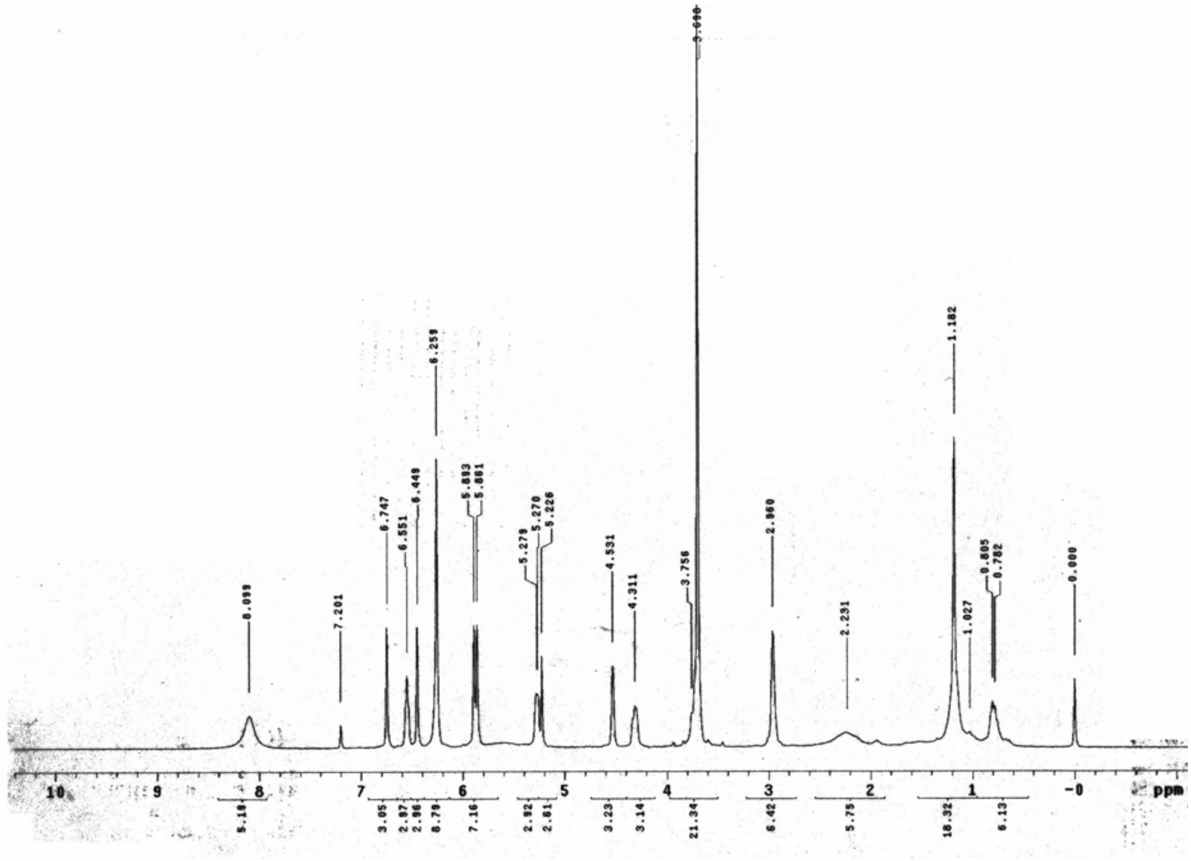

18.2. ^{13}C NMR spectrum of compound 4'N

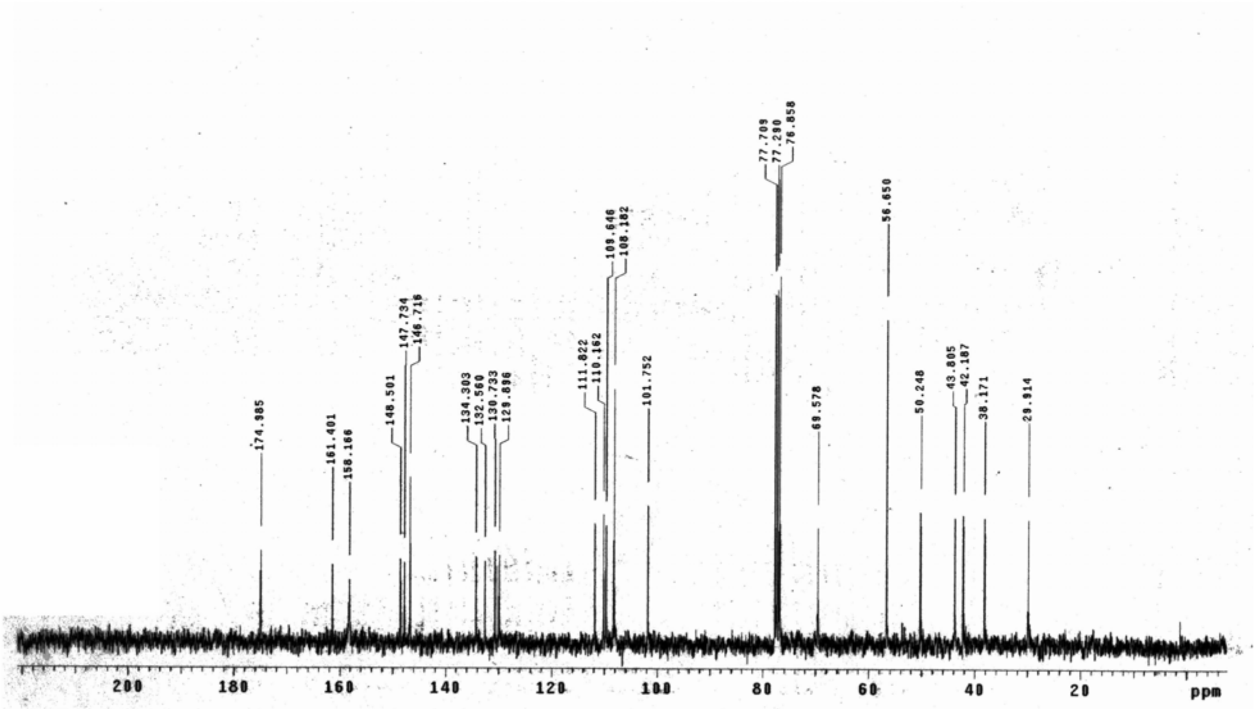

18.3. ^{1}H-^{1}H COSY diagram of compound 4'N

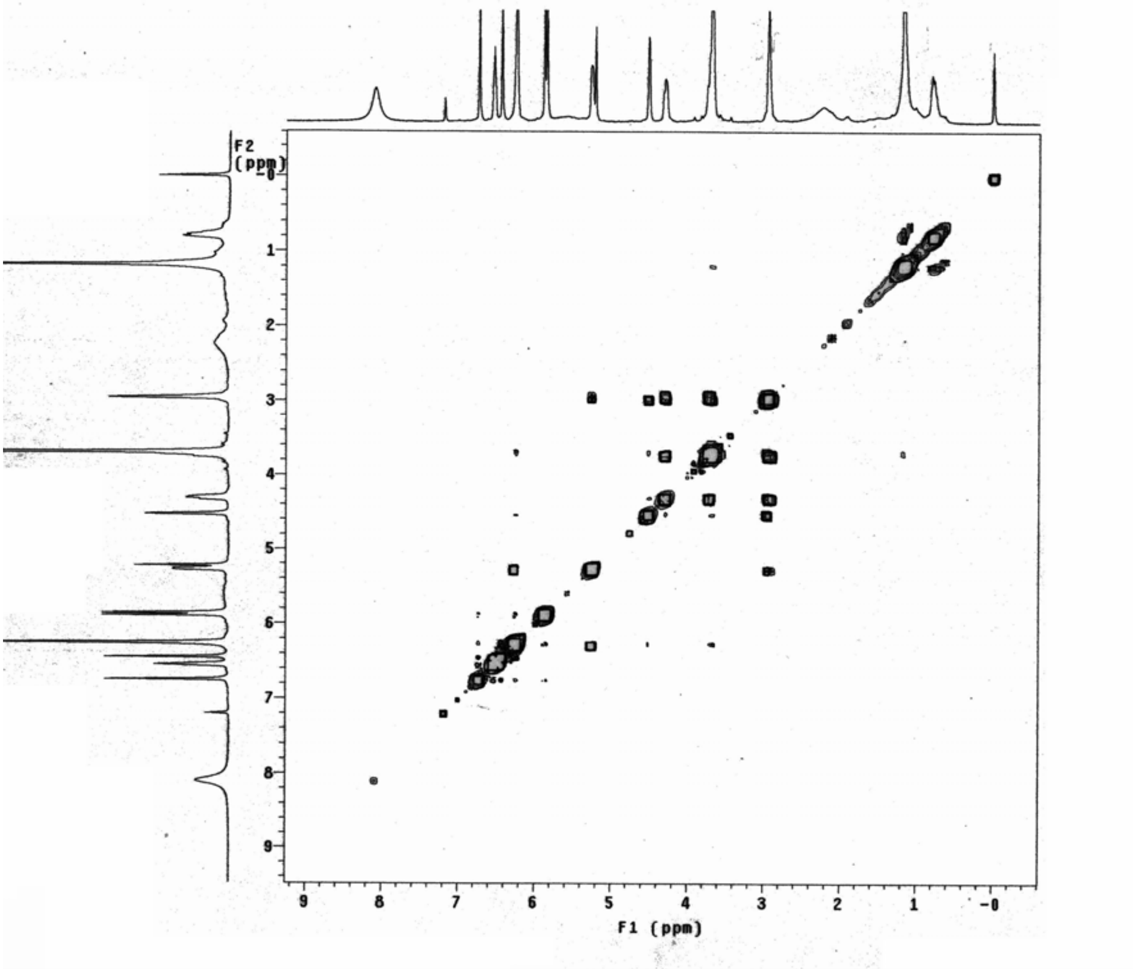

18.4. HMBC diagram of compound 4'N

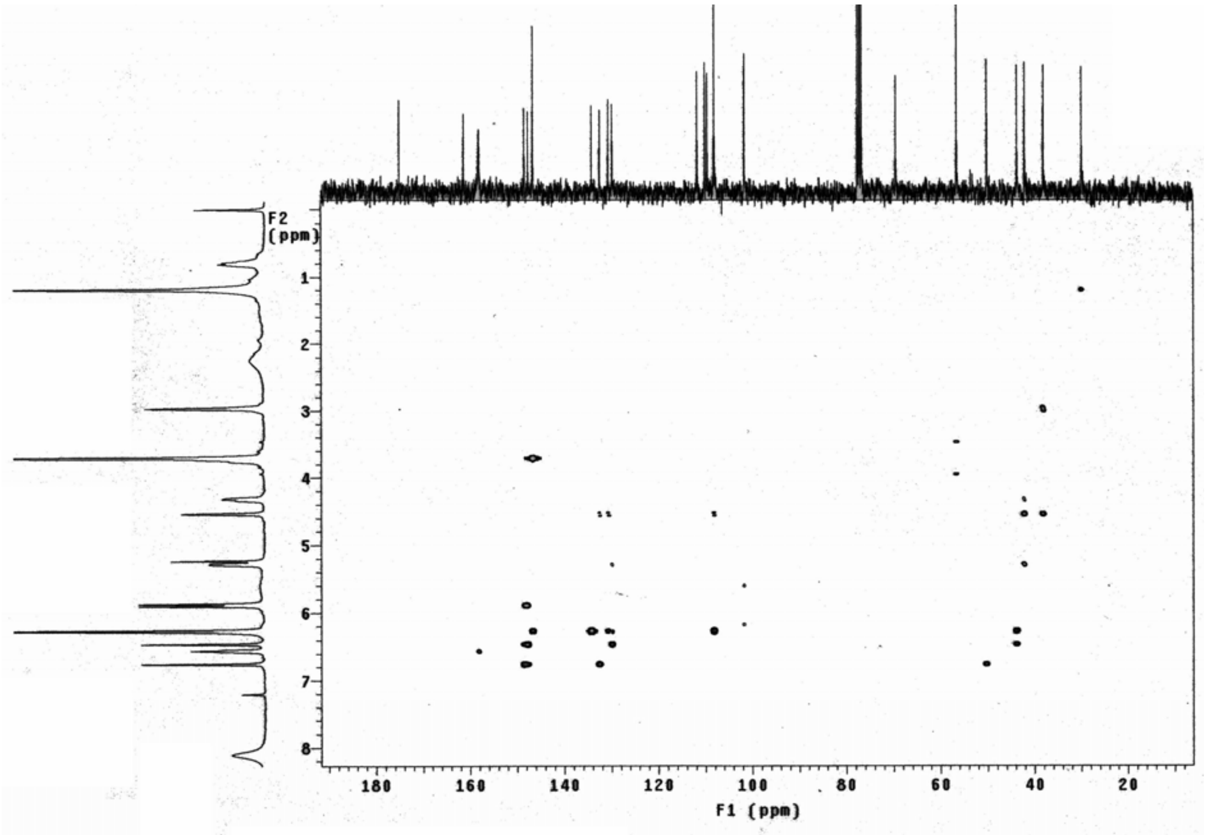

18.5. HSQC spectrums for compound 4'N.

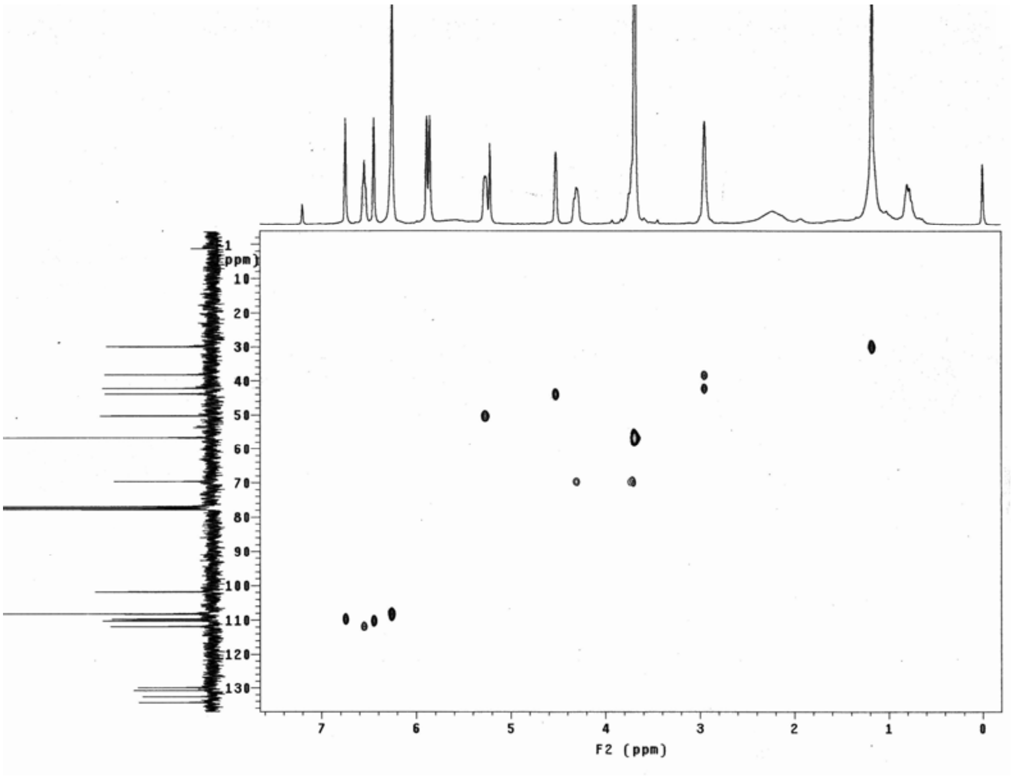

18.6. MS spectrum of compound 4'N

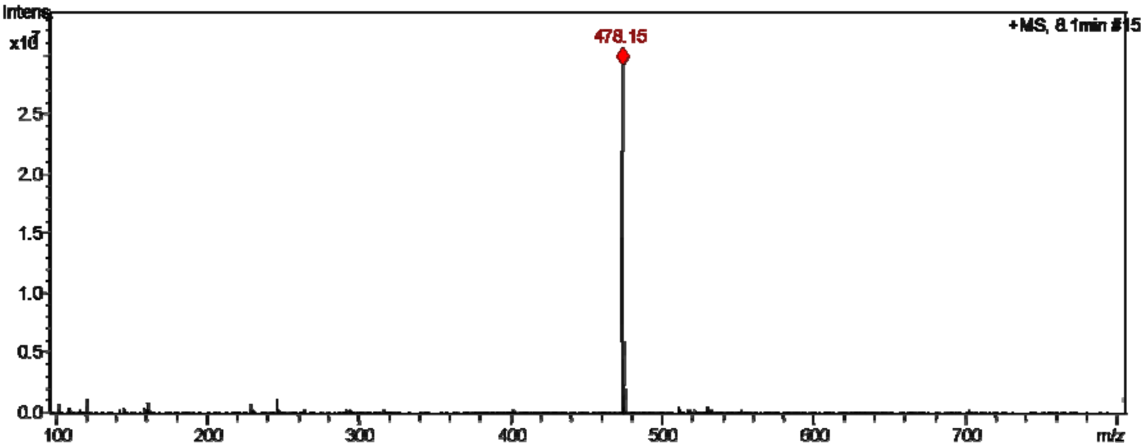

4β-N-(pyrimidine-2)-4-deoxy-4'-demethyl-podophyllotoxin (4'N)

^{1}H NMR (300 MHz, $CDCl_3$): δ 8.099 (s, 2H), 6.747 (s, 1H), 6.551 (s, 1H), 6.449 (s, 1H), 6.259 (s, 2H), 5.893 (d, *J* = 9.6 Hz, 2H), 5.279 (d, *J* = 2.7 Hz, 1H), 5.226 (s, 1H), 4.289 (t, *J* = 7.8 Hz, 1H), 4.531 (s, 1H), 4.311 (s, 1H), 3.756 (t, *J* = 5.4 Hz, 1H), 3.698 (s, 6H), 2.960 (s, 2H); ^{13}C NMR (75 MHz, $CDCl_3$): δ 174.985 , 161.401, 158.166, 148.501, 147.734, 146.716 (2C), 134.303, 132.560, 130.733, 129.896, 111.822, 110.162, 109.646, 108.182 (2C), 101.752, 69.578, 56.650 (2C), 50.248, 43.805, 42.187, 38.171, 29.914.

ESI-MS: calc'd for $C_{25}H_{23}N_3O_7$ $[M+H]^+$: 478.16, found 478.15 $[M+H]^+$.

19.1. ^{1}H NMR spectrum of compound 5'N

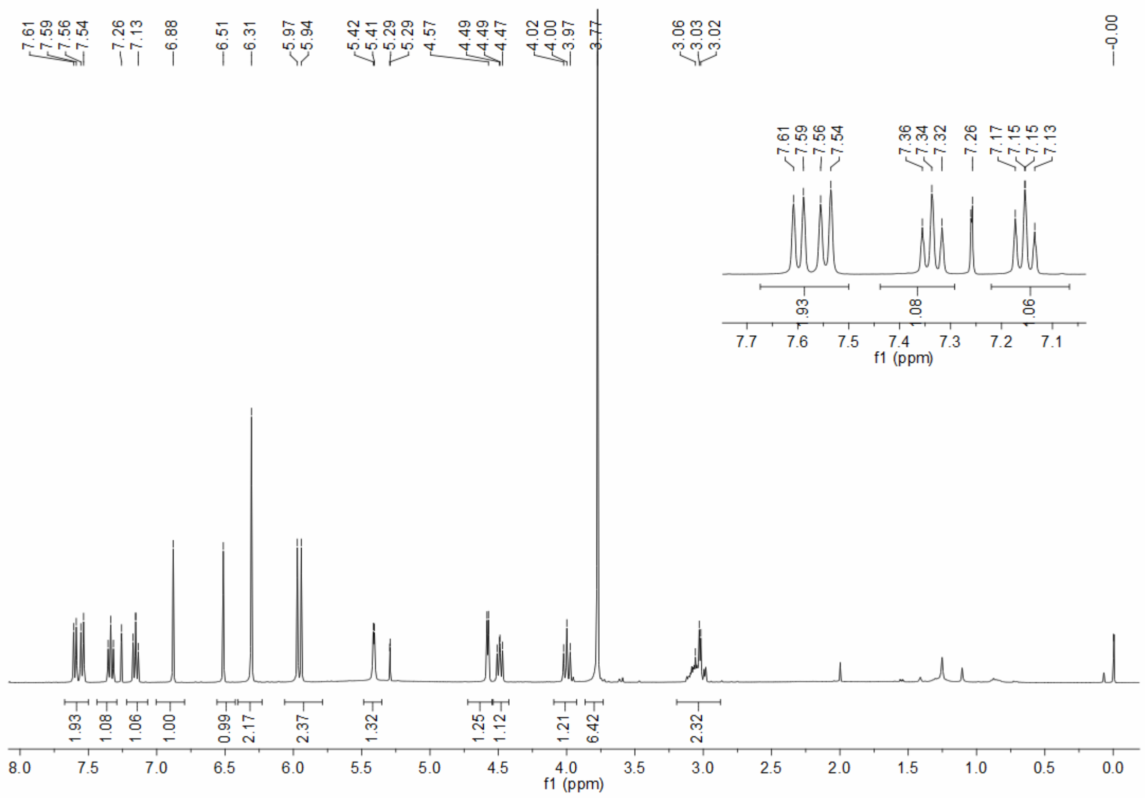

19.2. ^{13}C NMR spectrum of compound 5'N

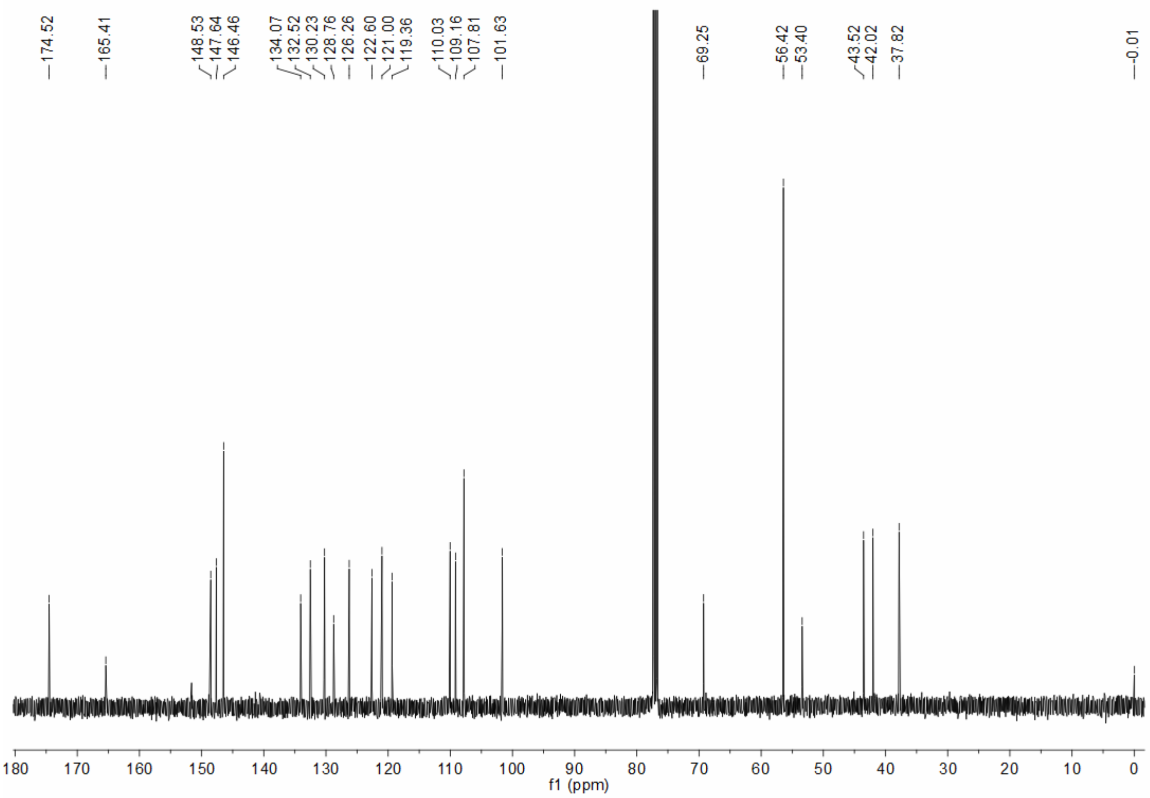

19.3. ^{1}H-^{1}H COSY diagram of compound 5'N

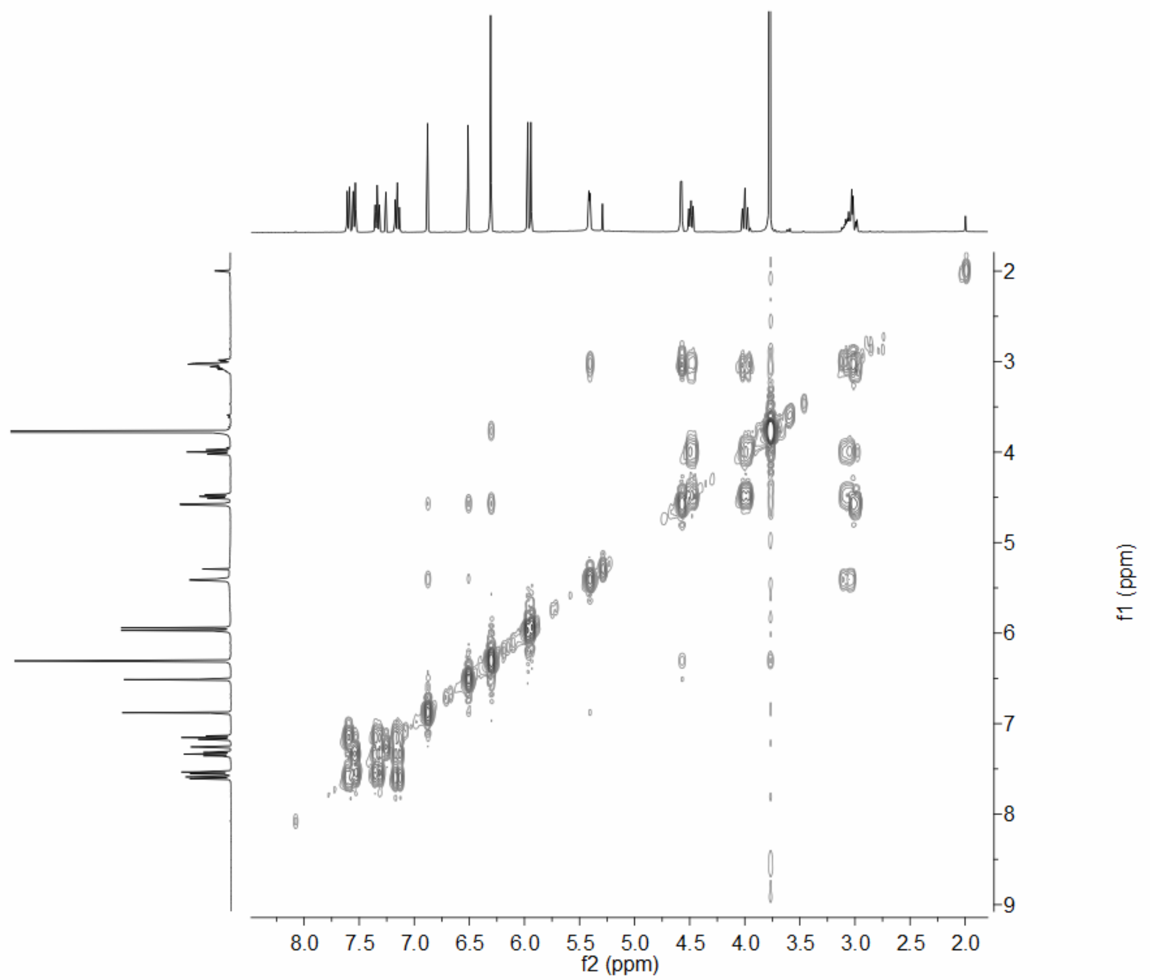

19.4. HMBC diagram of compound 5'N

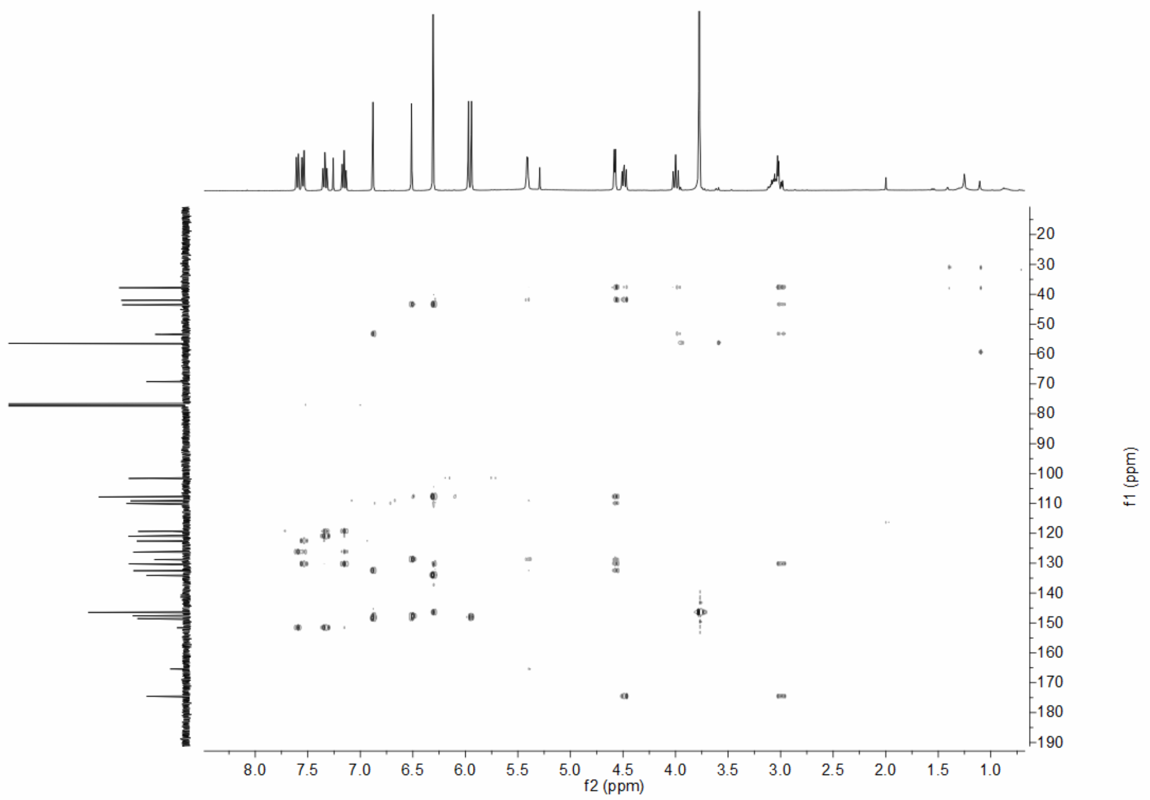

19.5. HSQC diagram of compound 5'N

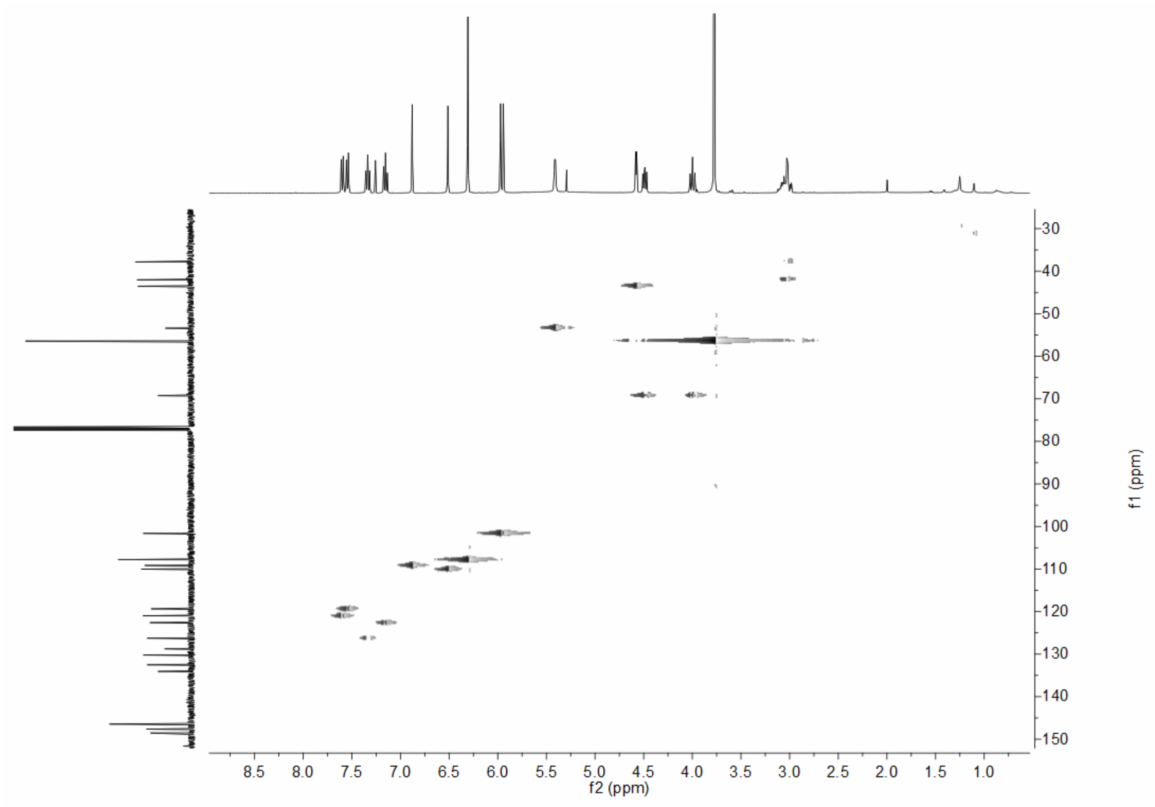

19.6. MS spectrum of compound 5'N

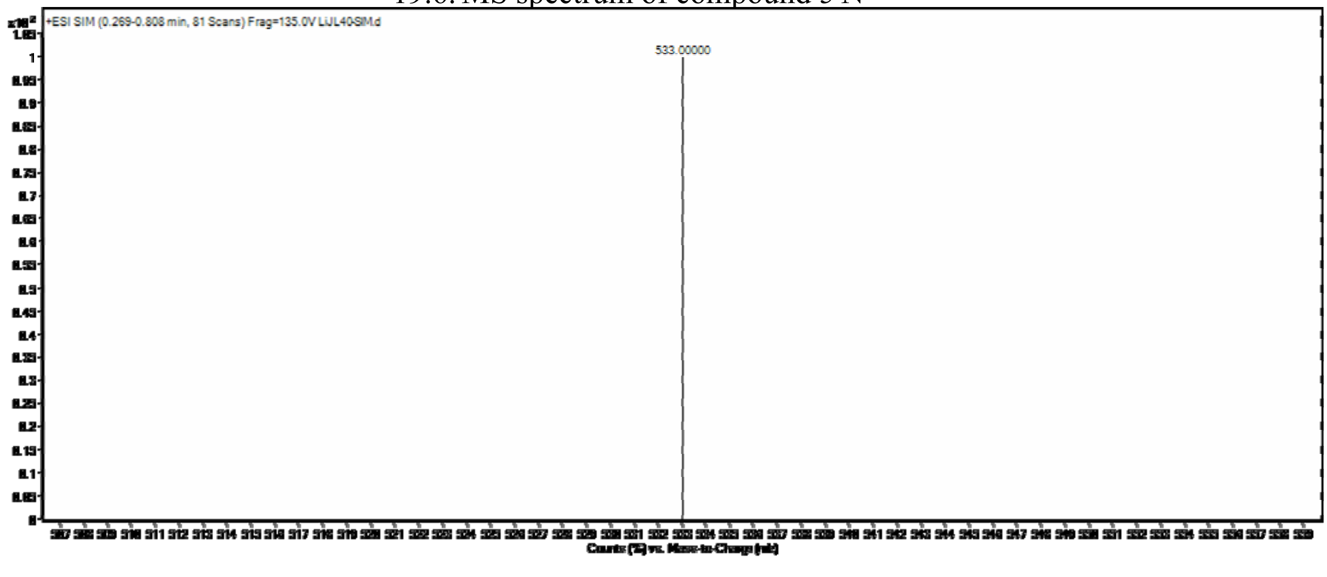

4β-N-(benzothiazole-2)-4-deoxy-4'-demethyl-podophyllotoxin (5'N)

^{1}H NMR (400 MHz, $CDCl_3$): δ 7.61 (d, *J* = 8.0 Hz, 1H), 7.56 (d, *J* = 8.0 Hz, 1H), 7.34 (t, *J* = 8.0 Hz, 1H), 7.15 (t, *J* = 8.0 Hz, 1H), 6.88 (s, 1H), 6.51 (s, 1H), 6.31 (s, 2H), 5.9 7(d, *J* = 12.0 Hz, 2H), 5.42 (d, *J* = 4.0 Hz, 1H), 4.58 (d, *J* = 4.0 Hz, 1H), 4.49 (t, *J* = 8.0 Hz, 1H), 4.00 (t, *J* = 8.0 Hz, 1H), 3.77 (s, 6H), 3.12-2.98 (m, 2H); ^{13}C NMR (101 MHz, $CDCl_3$): δ 174.52 (2C), 165.41, 151.61, 148.53, 147.64, 146.46 (2C), 134.07, 132.52, 130.23, 128.76, 126.26, 122.60 (2C), 121.00, 119.36, 110.03, 109.16, 107.81 (2C), 101.63, 69.25, 56.42 (2C), 53.40, 43.52, 42.02, 37.82.

ESI-MS: calc'd for $C_{28}H_{24}N_2O_7S$ $[M+H]^+$: 533.13, found 533.00 $[M+H]^+$.

20.1. ^{1}H NMR spectrum of compound 6'N.

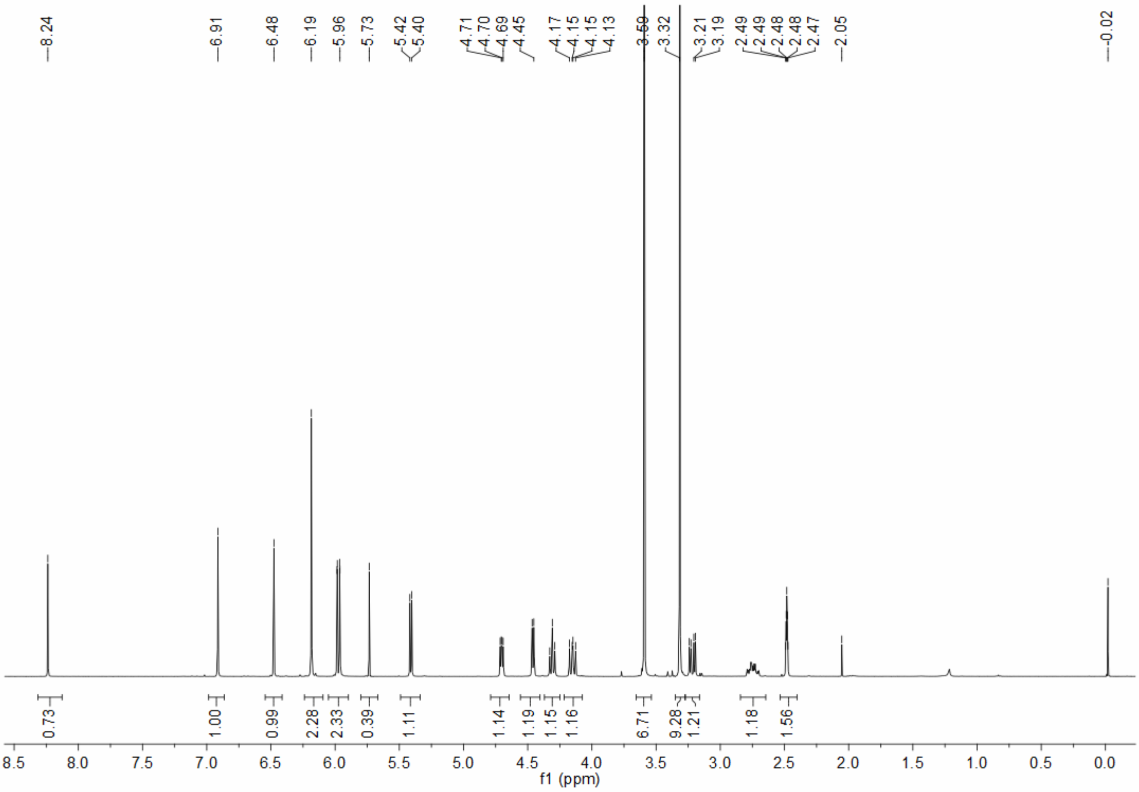

20.2. ^{13}C NMR spectrum of compound 6'N

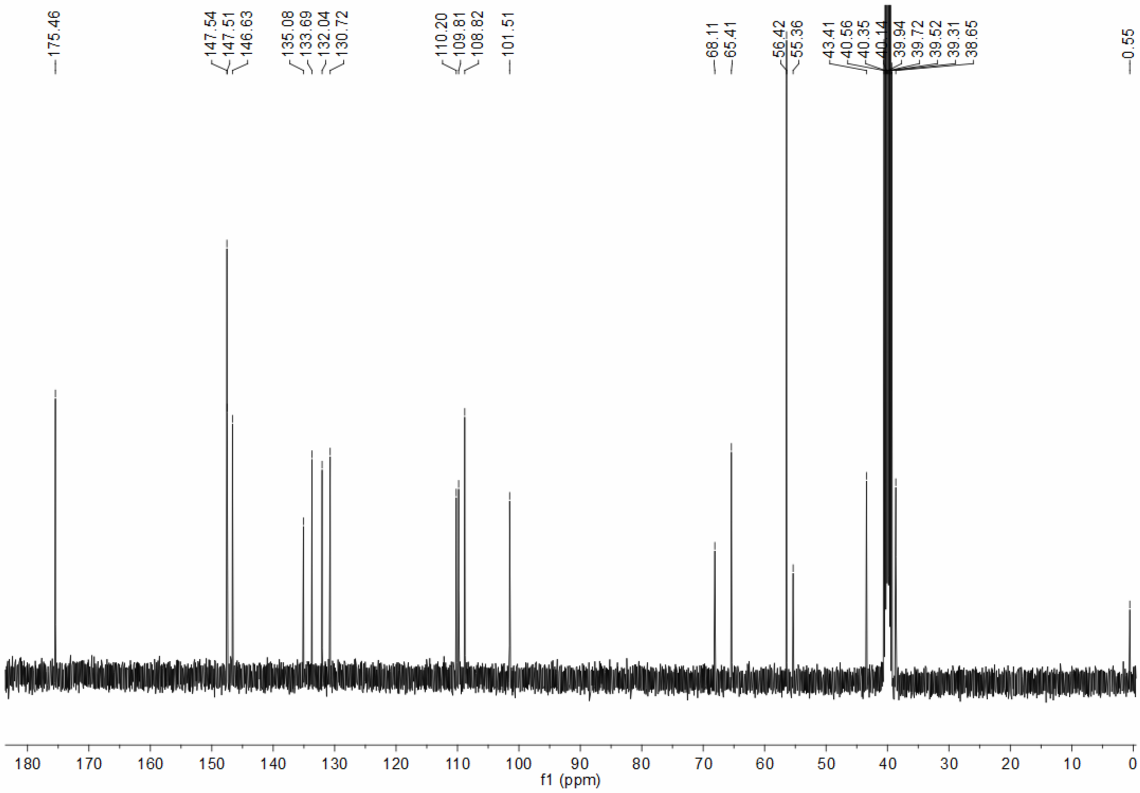

20.3. ^{1}H-^{1}H COSY diagram of compound 6'N

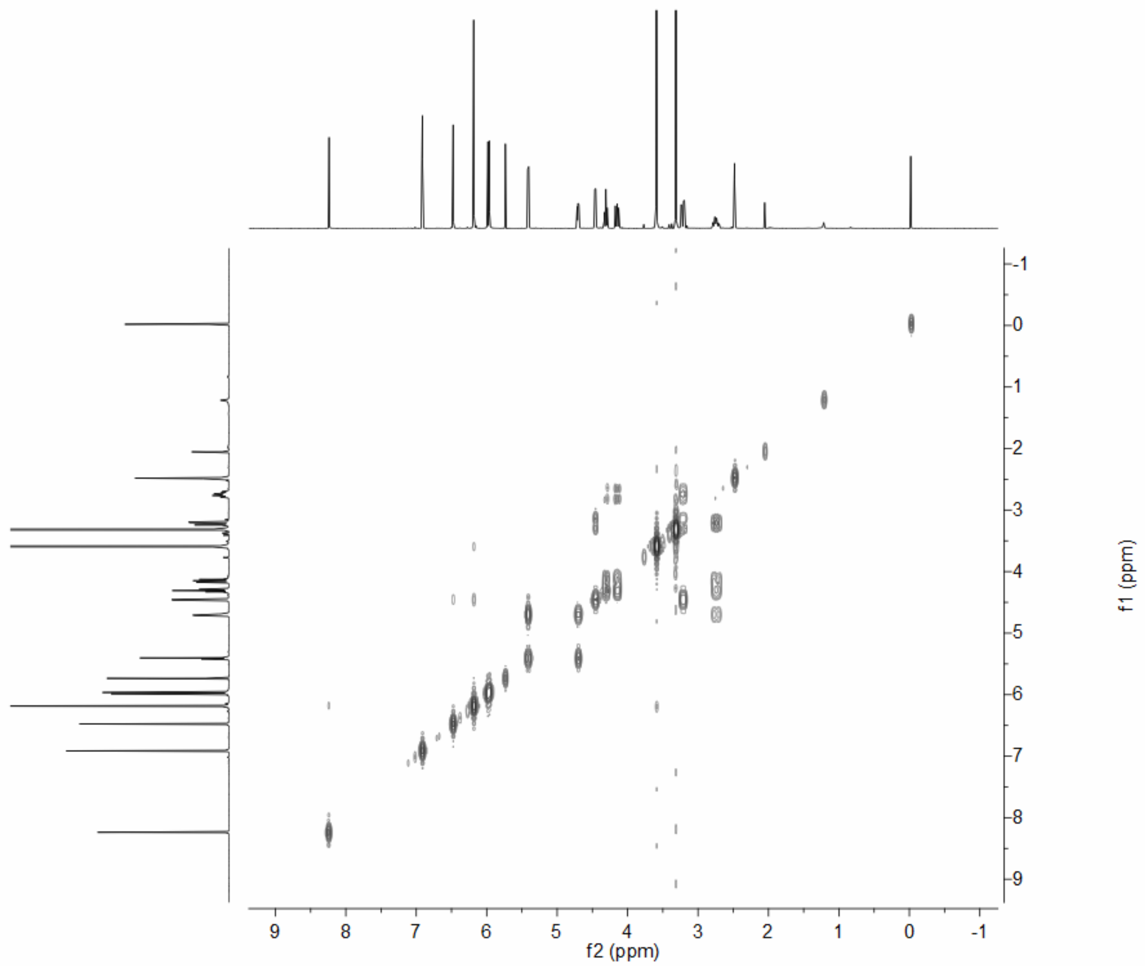

20.4. HMBC diagram of compound 6'N

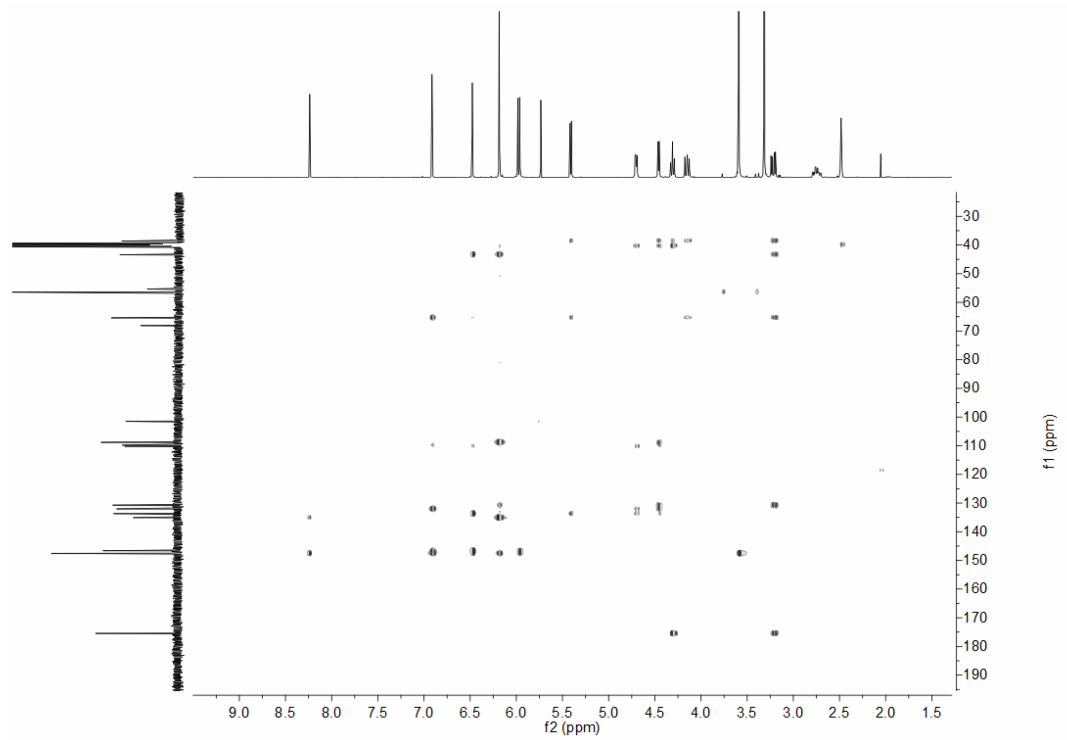

20.5. HSQC diagram of compound 6'N

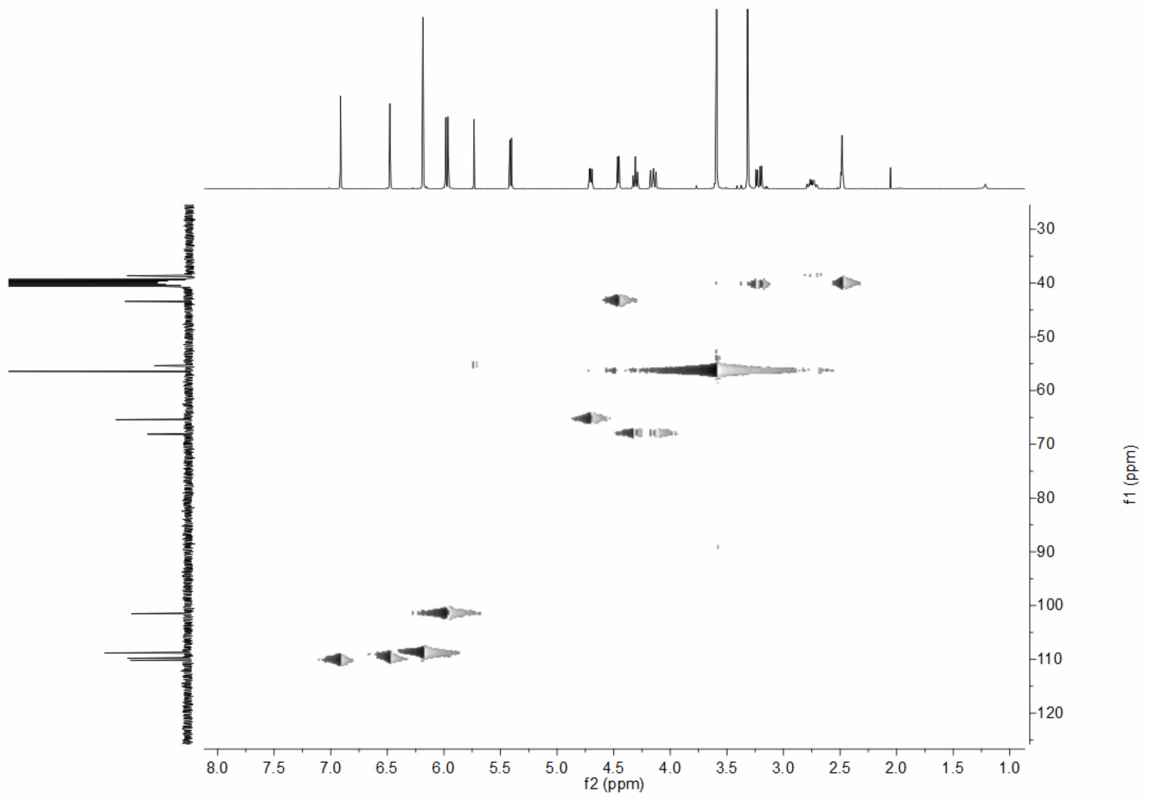

20.6. MS spectrum of compound 6'N

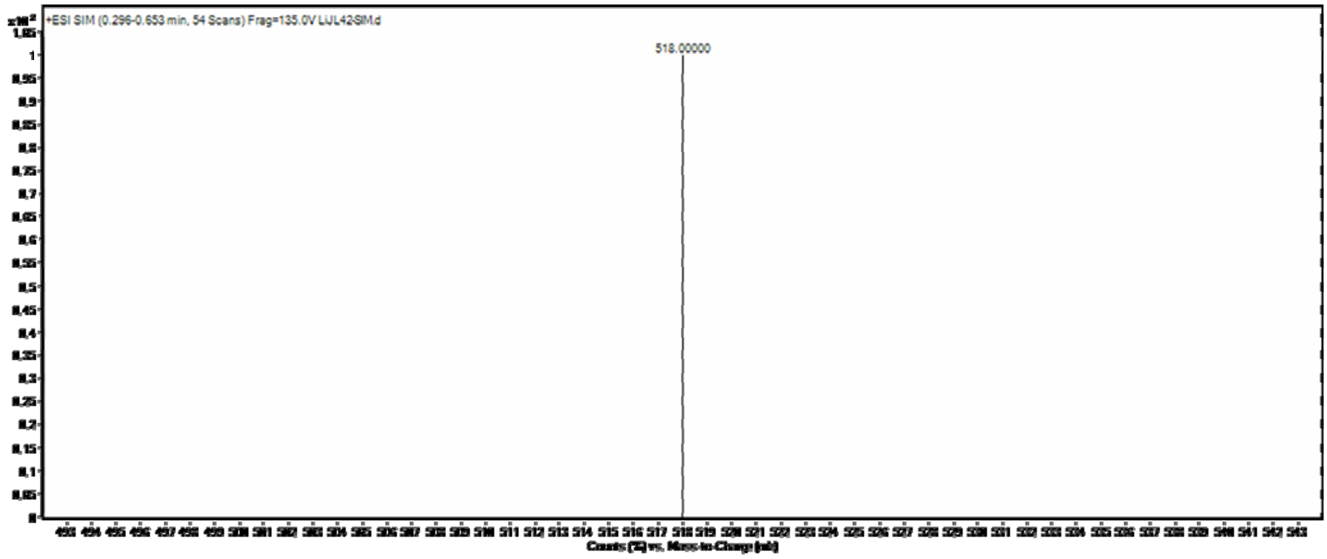

^{1}H NMR (400 MHz, DMSO): δ 8.24 (s, 1H), 6.91 (s, 1H), 6.48 (s, 1H), 6.19 (s, 2H), 5.98 (d, *J* = 8.0 Hz, 2H), 5.42 (d, *J* = 8.0 Hz, 1H), 4.71 (dd, *J* = 4.0 Hz, 1H), 4.47 (d, *J* = 8.0 Hz, 1H), 4.31 (t, J = 8.0 Hz, 1H), 4.15 (t, *J* = 8.0 Hz, 1H), 3.59 (s, 6H), 3.24-3.19 (m, 1H), 2.79 - 2.70 (m, 1H); ^{13}C NMR (101 MHz, DMSO): δ 175.46 (2C), 147.54, 147.51 (2C), 146.63 (2C), 135.08, 133.69 (2C), 132.04, 130.72 (2C), 110.20, 109.81, 108.82 (2C), 101.51, 68.11, 65.41, 56.42 (2C), 55.36, 43.41, 38.65.

ESI-MS: calc'd for $C_{26}H_{23}N_5O_7$ $[M+H]^+$: 518.16, found 518.00 $[M+H]^+$
